# Prevalent Accumulation of Non-Optimal Codons through Somatic Mutations in Human Cancers

**DOI:** 10.1371/journal.pone.0160463

**Published:** 2016-08-11

**Authors:** Xudong Wu, Guohui Li

**Affiliations:** Laboratory of Molecular Modeling and Design, State key Laboratory of Molecular Reaction Dynamics, Dalian Institute of Chemical Physics, Chinese Academy of Sciences, 457 Zhongshan Rd., Dalian 116023, PR China; Eidgenossische Technische Hochschule Zurich, SWITZERLAND

## Abstract

Cancer is characterized by uncontrolled cell growth, and the cause of different cancers is generally attributed to checkpoint dysregulation of cell proliferation and apoptosis. Recent studies have shown that non-optimal codons were preferentially adopted by genes to generate cell cycle-dependent oscillations in protein levels. This raises the intriguing question of how dynamic changes of codon usage modulate the cancer genome to cope with a non-controlled proliferative cell cycle. In this study, we comprehensively analyzed the somatic mutations of codons in human cancers, and found that non-optimal codons tended to be accumulated through both synonymous and non-synonymous mutations compared with other types of genomic substitution. We further demonstrated that non-optimal codons were prevalently accumulated across different types of cancers, amino acids, and chromosomes, and genes with accumulation of non-optimal codons tended to be involved in protein interaction/signaling networks and encoded important enzymes in metabolic networks that played roles in cancer-related pathways. This study provides insights into the dynamics of codons in the cancer genome and demonstrates that accumulation of non-optimal codons may be an adaptive strategy for cancerous cells to win the competition with normal cells. This deeper interpretation of the patterns and the functional characterization of somatic mutations of codons will help to broaden the current understanding of the molecular basis of cancers.

## Introduction

Genetic redundancy refers to multiple copies of the same or similar genetic sequences [1]. The benefit comes from having backups of genes with similar functions by gene duplication or by up-regulating gene products and making more products to drive efficiency. The ‘redundancy’ in the genetic code refers to requiring fewer than 61 tRNAs when 61 codons are translated (isoaccepting codons) [2], especially in cases where the base at the 5’ end of the anticodon is inosine. According to the ‘wobble’ base-pairing rules, the four main wobble base pairs include guanine-uracil (G:U), inosine-uracil (I:U), inosine-adenine (I:A) and inosine-cytosine (I:C) [3]. Codons can be classified as optimal or non-optimal, where non-optimal codons are characterized by wobble-pairing a low concentration of isoaccepting tRNAs with low binding affinities [4].

The biological importance of non-optimal codon usage has been studied for a long time. Kimchi-Sarfaty *et al* revealed that synonymous changes for non-optimal codons had effects on the expression of human genes [5]. Makhoul and Trifonov reported that non-optimal codons played a key role in translation ‘pausing’ between protein domains [6]. Zhou *et al* reported that non-optimal codons regulated protein expression to gain optimal protein structure and function [7]. The frequency (*frq*) gene which has a rhythmic expression pattern that is essential for circadian clock function in *Neurospora*, has been shown to exhibit non-optimal codon usage across its coding region. Optimization of *frq* codon usage resulted in impaired circadian feedback loops and abolished circadian rhythms [7]. Recently, the role of non-optimal codons’ wobble codon—anticodon base pairing in regulating the temporal aspects of protein translation has been recognized. For example, Frenkel-Morgenstern *et al* found that cell cycle regulated genes used non-optimal codons to achieve elongation-limited mRNA translation in eukaryotes as diverse as *Schizosaccharomyces pombe*, *Saccharomyces cerevisiae*, *Arabidopsis thaliana* and *Homo sapiens* [8]. Their simulations indicated that non-optimal codon preferences of cell cycle regulated genes provided opportunities for changes in the tRNA pool to generate cell cycle-dependent oscillations of protein abundance [8].

Cancer is characterized by uncontrolled cell cycle, checkpoint dysregulation of cell differentiation, proliferation, and apoptosis. The application of whole-genome sequencing has contributed to the detection of multiple somatic genetic and epigenetic alterations that occur in cancer cells [9,10]. Somatic mutations caused by carcinogens (environmental factors that increase cancer risk) include point mutations, deletions, gene fusions, gene amplifications and chromosomal rearrangements [11–16]. As a normal part of the aging process, the accumulation of a large number of mutations in a specific group of cells can cause cell division and growth get out of control [17], consequently leading to aggressive malignancy and invasive phenotypes [18–20]. In this study, we analyzed the properties of somatic mutations, and investigated their transformations among optimal and non-optimal codons in several cancers. In our analysis, we focused on two points: (i) whether the non-optimal codons were predominately accumulated; and (ii) what was the cellular function of genes with different patterns of non-optimal codon accumulation.

## Materials and Methods

### Somatic mutations of codons in cancer genomes

The International Cancer Genome Consortium (ICGC) integrated available genomic, transcriptomic and epigenetic data from many different research groups [21]. Somatic mutations were identified by cancer genomics projects, the files with nomenclature like ssm.*.txt.gz, were downloaded from the ICGC data portal (version 11), the source files for each type of cancers were complied in S1 Table. A subset of mutations matching the human genome build 36 was mapped to build 37 with the LiftOver software of the UCSC Genome Browser [22].

In each source file, the ‘Mutation’ column was analyzed. The mutations were displayed like ‘W>M’, where the ‘W’ represented the reference nucleotide acid and the ‘M’ represented the mutant nucleotide acid. The multi-nucleotide substitutions, insertions and deletions were discarded from the datasets.

The genomic coordinates of human genes were retrieved from GENCODE database (version 15) [23], and the hg19 (GRCH 37) human genome was used for analysis. The protein-coding transcripts with complete coding sequence, namely with both start codon and stop codon annotated, were used for mapping the somatic mutations. The mutations were discarded if they created premature stop codons, the remained non-synonymous/synonymous single nucleotide variants (SNVs) were analyzed. Finally, a total of 135760 somatic mutations were complied and referred to as CSM dataset (S2 Table).

### Evolutionary substitutions of codons between close species

The One2One orthologs between *Homo sapiens* and *Pan troglodytes* were retrieved through BioMart [24]. For each gene, the isoform with the longest transcript was used. The Clustalw software was used to align the protein sequences of *Human*-*Chimp* orthologs globally [25], and then the corresponding coding sequences were realigned with the gaps in the alignment trimmed. The ortholog codons with only one difference of nucleotide acid were analyzed. Finally, a total of 180346 nucleotide variation were compiled and referred to as Ortholog-Poly (S3 Table).

### Single nucleotide polymorphism of codons among populations

Single nucleotide polymorphisms (SNPs) were targeted by the HapMap project and have been widely employed in Genome Wide Association Studies for complex traits (GWAS) [26]. The HapMap Phase III SNPs were retrieved from http://hgdownload.cse.ucsc.edu/goldenPath/hg19/database/, including ten populations, CEU, CHB, CHD, GIH, JPT, LWK, MEX, MKK, TSI, YRI [27]. Minor allele frequency (MAF) refers to the frequency at which the less common allele occurs in a given population. The SNPs with MAF of ≥ 5% were mapped onto the coding regions in each of populations. Finally, a total of 35269 nucleotide variants located in protein-coding genes were compiled and referred to as SNP-Poly (S4 Table).

### Translational Optimal codons and Non-optimal codons

According to the studies of Watkins *et al* [4] and Frenkel-Morgenstern *et al* [8], the following codons were characterized by low codon—anticodon affinities and defined as non-optimal codons: *GCA*,*GCT*,*AGA*,*CGA*,*CGT*,*AAT*,*GAT*,*TGT*,*CAA*,*GAA*,*GGA*,*GGT*,*CAT*,*ATA*,*ATT*,*CTA*,*CTT*,*TTA*,*AAA*,*TTT*,*CCA*,*CCT*,*AGT*,*TCA*,*TCT*,*ACA*,*ACT*,*TAT*,*GTA*,*GTT*.

The other codons were defined as optimal codons: *GCC*,*GCG*,*AGG*,*CGC*,*CGG*,*AAC*,*GAC*,*TGC*,*CAG*,*GAG*,*GGC*,*GGG*,*CAC*,*ATC*,*CTC*,*CTG*,*TTG*,*AAG*,*ATG*,*TTC*,*CCC*,*CCG*,*AGC*,*TCC*,*TCG*,*ACC*,*ACG*,*TGG*,*TAC*,*GTC*,*GTG*.

The classification of optimal and non-optimal codons was based on the binding free energy between codons and anticodons at translational stage, the set of optimal codons in chimp was identical in *Homo sapiens* and *Pan troglodytes*.

### Analysis of human cellular signaling network

The human signaling network dataset was downloaded from www.bri.nrc.ca/wang [28]. The nodes with ‘activation’ and ‘inhibition’ regulatory relationships were retrieved. After transforming the gene names to ensembl genes ids, a total of 5405 genes with somatic mutations in CSM were located in the signaling transduction networks. For each gene, the number of regulator genes was used to measure its importance and regulation complexity in the signal transduction network.

### Flux Balance Analysis

Recon 2 contained 7440 reactions and 2626 unique metabolites distributed in eight cellular compartments, it represented the most comprehensive ‘metabolic reconstruction’ of human metabolism [29]. The model of Recon 2 was retrieved from the http://humanmetabolism.org/ (Biomodels model: *1109130000*; SBML format) and loaded with the ‘readCbModel’ in COBRA Toolbox [29]. FBA formalized the system of equations, and described the metabolic network as the dot product of a matrix of the stoichiometric coefficients (the S matrix) and the vector of the unsolved fluxes (V). Linear programming was used to calculate a solution of fluxes corresponding to the steady state by *Cobra* package [30].

The FBA was performed to maximize C^*T*^X, subjected to *SV* = 0 and *lb* ≤ x ≤ *ub*. The *lb* represented the lower-bound, and *ub* represented upper-bound. The *V* was the vector of fluxes to be determined, and *S* was a matrix of coefficients. The maximation of biomass production was set to be the objective function (C^*T*^X in this case). The inequalities lower bound and upper bound established the maximal rates of flux for every reaction (the columns of the *S* matrix). Using the network and the stoichiometry, every possible reaction knockouts were made. The lower-bound and upper-bound of the targeted reaction flux were constrained to 0, and the remainder of the network was re-optimized for maximation of biomass. The maximum flux across all possible conditions was selected for each reaction.

To connect the metabolic reaction with the *ensembl gene ids*, the *gene species ids* and their corresponding *gene symbols* were retrieved from MODEL1109130000.xml, and then *the gene species ids* and their metabolic reaction were linked by the *genes* and *rxnGeneMat* tables of model. After transforming the *gene symbols* to *ensembl gene ids*, the flux values of 3912 reactions catalyzed by 1,623 *ensembl* genes were obtained.

### Analysis of human enzyme-enzyme metabolic network

The model of Recon 2 [29] was used to reconstruct the enzyme-centered metabolic network. The enzymes were represented by nodes, and substrate-product metabolite flux were represented by directional edges. Briefly, the reactions with assigned EC-number were retrieved using the *rxnECNumbers* table of model, the direction of reactions were determined by the *rev* table of model, and then the transformations between metabolites were used to determine the interactions among these enzymes using the *S* matrix of model. For instance, enzyme *EC2*.*7*.*7*.*9* uses alpha-D-glucose-1-phosphate as substrate to produce UDP-glucose, which was then used by enzyme *EC5*.*1*.*3*.*2*, the interaction was defined as EC2.7.7.9 -> EC 5.1.3.2. Because small molecules, *adp*, *amp*, *nad*, *nadh*, *nadp*, *nadph*, *nh4*, *coa*, *o2*, *co2*, *glu*, *pyr*, *h*, *accoa*, *fad*, *fadh2*, *hco3*, *pi*, *ppi*, *h2o*, *na1* and *udp*, are involved in many reactions or are used as carriers for transferring electrons [31], they were excluded from the analysis based on the *mets* and *metFormulas* tables of Recon 2 model.

The enzyme-enzyme metabolic network was constructed with 3,648 directional interactions among 685 enzymes, in which 662 enzymes were included in a large network and the other 23 enzymes in 5 small clusters. The large connected network contained 1826 directional interactions and 899 bi-directional interactions. For directional interaction, the metabolite was the substrate or product of particular enzyme. For the bi-directional interaction, the metabolite was used as substrate as well as product of the same enzyme.

Degree was an important measure of the importance of biological network. For metabolic network with directional interactions, the topological centralities were used to measure the importance of nodes in the control of information transfer. In-degree referred to the number of links forwarded to the considered nodes, out-degree referred to the number of links outwards from the considered nodes, and the nodes with relatively higher degrees are termed as hubs.

### Proto-Oncogenes and Tumor repressors

The proto-oncogenes were retrieved from UniProt (http://www.uniprot.org/uniprot/?query=keyword:KW-0656) and RAS Oncogene Database (http://14.139.245.18/rasond/home.php) [32]. After transforming the *UniProt Entry* and *RefSeq Id* to *ensembl gene id*, the 362 proto-oncogenes with somatic mutations available in CSM were obtained. The tumor repressor genes were downloaded from TSGene database (http://bioinfo.mc.vanderbilt.edu/TSGene/) [33], and 608 tumor repressor genes with somatic mutations available in CSM were obtained.

### Analysis of gene expression profiles

The microarray gene expression profiles of 79 human tissues were extracted from Su et al. [34], and the probe set sequences were assigned by the human coding sequences by BioMart [24]. Two replicates of each tissue were averaged to determine the gene expression intensity in the corresponding tissue. The multiple tissues representing similar areas were grouped and the highest expression level from any tissue in a group were taken as the representative expression intensity for the tissue group (the expression levels in pathogenic tissues were not considered). A gene was identified to be tissue-specific if the expression intensity of the highest tissue group was greater than or equal to twice the expression intensity of the second highest tissue group. For genes with accumulation of non-optimal codons, 6918 genes have microarray expression information and 2208 genes were identified to be tissue-specific. For genes without accumulation of non-optimal codons, 4811 genes have microarray expression information and 1518 genes were identified to be tissue-specific.

Recently, Peng *et al* performed a large-scale *RNA-Seq* transcriptome analysis of cancers and normal tissue controls across 12 cancer types (IlluminaHiSeq_RNASeqV2) [35]. The samples in the clinical category of “primary tumor” or “solid tissue normal” were used for identification of differentially expressed genes in the corresponding cancers. We used the *fdr* smaller than 0.001 as cutoff to retrieve these differentially expressed genes.

### Compilation of codon transformations in COSMIC and GWAS datasets

The COSMIC database (version67) were retrieved from ftp://ftp.sanger.ac.uk/pub/CGP/cosmic/data_export/ [36], and the entries recorded as “confirmed somatic mutations” were analyzed. To avoid the influence of alternative spliced isoforms on the calculation of mutation event, a unique identifier “genomic position—gene—mutation” was counted once. The GWAS dataset were downloaded from EBI GWAS Catalogue (https://www.ebi.ac.uk/gwas/) [26,27], and the *rs* identifiers were used to map the corresponding mutations onto the mRNA. The cancer related GWAS-SNPs were filtered out, and only the disease related GWAS SNPs were analyzed.

### Functional analysis of human genes based on gene ontology

The Gene Ontology (GO) provided three structured controlled vocabularies to describe gene products [37,38]. The human gene association file was downloaded from http://www.geneontology.org/gene-associations/ and compiled by BioMart [24]. For each GO term, the enrichment of annotated genes among the genes with accumulation of non-optimal codons was investigated by the “Functional Annotation” in http://david.abcc.ncifcrf.gov/. The Benjamini corrected p-value with a cutoff of p ≤ 0.001 was used to identify the over-represented GO terms among the genes with accumulation of non-optimal codons.

### Aggregation score and Disorder predictions

The amyloidal aggregation propensities of the 20 naturally occurring amino acids were retrieved from the study of Pawar *et al* [39], which were estimated based on amino acid hydrophobicity, the alpha-helical propensity, the beta-sheet propensity, the hydrophobic patterning and the charge.

Protein disorder was predicted by IUPRED [40] on full length wild-type (WT) and mutated protein sequences, which was generated by changing only one residue at a time. The effects of a mutation were investigated by comparing the predicted score between a residue to be mutated in the WT protein and after the mutation. For one mutation located in codons of different transcripts, all of the transcripts were analyzed.

### Computational environment

The project was started and completed in Dalian Institute of chemical Physics. Computations were performed on a Linux cluster with 50 nodes (Intel 5130, 2.0 GHz CPU, 4G memory, Laboratory of Molecular Modeling and Design, Dalian Institute of Chemical Physics, Chinese Academy of Sciences).

## Results

### Preferential accumulation of non-optimal codons in cancer genomes

We obtained the cancer somatic mutations from the International Cancer Genome Consortium [21], and mapped them onto the coordinates of the ensembl genes to investigate their impact on codon transformations (CSM, see Methods). We also compiled datasets of human genome-wide natural codon variations and used them as the background for comparisons: codon variations of ortholog genes between human and chimp (Ortholog-Poly, S3 Table), and codon variations in the population polymorphisms [41] (SNP-Poly, S4 Table).

According to the different outcomes, the effects of mutations were classified as O->O (optimal to optimal) and N->N (non-optimal to non-optimal) transformations when the optimal and non-optimal assignments did not change; and as O->N (optimal to non-optimal) and N->O (non-optimal to optimal) transformations when optimal and non-optimal assignments switched.

The mutations were classified as synonymous (no amino acid change) and non-synonymous (amino acid change) [42,43], and then the dynamics of the optimal and non-optimal codons were investigated separately. As shown in Table 1, about 8.50% of the cancer non-synonymous mutations in optimal codons resulted in non-optimal codons, while this percentage decreased to only 4.15% and 4.08% in the SNP-Poly and Ortholog-Poly datasets (p = 9.32e-52 and 4.57e-180, *Chi-square*, *two-tail test*). About 3.88% of the cancer non-synonymous mutations of non-optimal codons result in optimal codons, and the percentage increased to 4.50% and 5.75% in the SNP-Poly and Ortholog-Poly datasets (p = 7.19e-3 and 2.16e-31, *Chi-square*, *two-tail test*). A similar tendency was observed for the cancer synonymous mutations (p ≤ 5.70e-15 for four comparisons, *Chi-square*, *two-tail test*). Therefore, cancer mutations contained significantly higher frequencies of O->N transformations and lower frequencies of N->O transformations. Although synonymous and non-synonymous mutations have different intrinsic propensities for non-optimal/optimal codons transformation, the O->N transformations were favored and the N->O transformations were disfavored in cancers.

**Table 1:** The frequencies of O->N and N->O transformations. The p-values were estimated by *Chi-square*, *two-tail test*.

	Dataset	O->O	O->N	%	p-value	N->N	N->O	%	p-value
**Non-Synonymous mutations**	**CSM**	53845	**5003**	8.50	-	38803	**1567**	3.88	-
	**SNP-Poly**	9846	**426**	4.15	9.32E-52	7166	**341**	4.50	*0*.*007193*
	**Ortholog-Poly**	43674	**1856**	4.08	*4*.*57E-180*	28749	**1754**	5.75	*2*.*16E-31*
**Synonymous mutations**	**CSM**	2872	**24774**	89.61	-	1599	**7297**	82.02	-
	**SNP-Poly**	1397	**9186**	86.80	5.70E-15	473	**6434**	93.15	6.96E-94
	**Ortholog-Poly**	10398	**45212**	81.30	*5*.*80E-209*	3576	**45127**	92.66	*4*.*02E-228*

Intuitively, the preferential transformation from optimal to non-optimal codons would be expected to contribute to the widespread accumulation of non-optimal codons in cancers. We estimated this by subtracting the number of N->O from the number of O->N transformations, for 135760 cancer mutations that occurred in codons, a total of 20913 non-optimal codons (5003–1567+24774–7297) were accumulated, which corresponded to 15.40% by 20913/135760. This percentage was significantly higher than that observed in the SNP-Poly (20913/135760 *vs*. 2837/35269, p<0.001, *Chi-square*, *two-tail test*) and Ortholog-Poly (20913/135760 *vs*. 187/180346, p<0.001, *Chi-square*, *two-tail test*) datasets, respectively.

### Prevalent accumulation of non-optimal codons across different cancers, amino acids, and chromosomes

Because the dataset of cancer mutations was integrated from several types of cancers, amino acids, and chromosomes, it is possible that the observed of preferential accumulation of non-optimal codons is attributable to only a few types of cancers, amino acids, or chromosomes. To control these potential biases, we investigated the optimal/non-optimal codon transformations separately in each type of cancer, each amino acids, and each chromosome.

**Fig 1:**
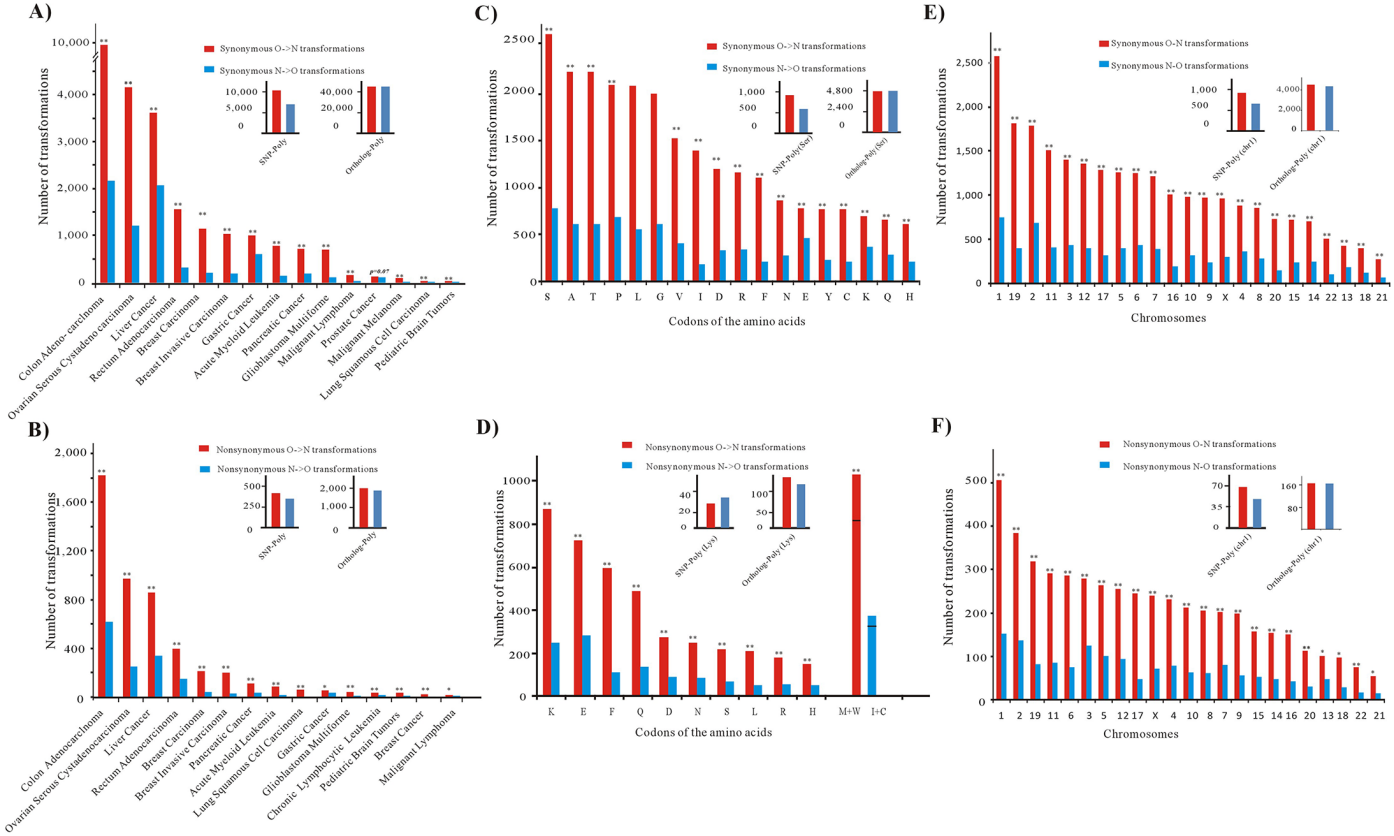
O->N transformations enriched in different cancers, amino acids and chromosomes. (**a**) Synonymous mutations in different types of cancers. (**b**) Non-synonymous mutations in different types of cancers. (**c**) Synonymous mutation for each amino acid. (**d**) Non-synonymous mutations for each amino acid. (**e**) Synonymous mutations in each human chromosomes. (**f**) Non-synonymous mutations in each human chromosome. The p-values were estimated by the comparison between CSM and Ortholog-Poly (*Chi-square*, *two-tail test*), ** p-values ≤ 0.01; * p-values between 0.01 and 0.05.

For each type of cancers, we counted the O->N and N->O transformations. The fold was calculated by dividing the number of O->N by the number of N->O transformations, and then comparing it with the fold observed in the Ortholog-Poly and SNP-Poly datasets. As shown in Fig 1a for synonymous mutations, 45212 optimal codons were transformed to non-optimal codons and 45127 non-optimal codons were transformed to optimal codons in Ortholgy-Poly, and in the 15 cancers with available data for statistical analysis, 14 cancers clearly showed significantly higher numbers of O->N transformations (see the detailed number and the p-values in S5 Table); As shown in the Fig 1b for non-synonymous mutations, 1856 optimal codons were transformed to non-optimal codons and 1754 non-optimal codons were transformed to optimal codons in Ortholgy-Poly, and in the 15 cancers with available data for statistical analysis, each of the cancers clearly showed significantly higher number of O->N transformations (S6 Table).

Similarly, for each amino acid and chromosome, we calculated the number of O->N and N->O transformations, and compared the fold with that observed for related transformations in Ortholog-Poly and SNP-Poly. The results demonstrated that the accumulation of non-optimal codons did not depend on the types of amino acids (Fig 1c and S7 Table for statistical analysis for synonymous mutations of each amino acid, Fig 1d and S8 Table for statistical analysis for non-synonymous mutations of each amino acid) or the location of chromosomes (Fig 1e and S9 Table for statistical analysis for synonymous mutations of each chromosome, Fig 1f and S10 Table for statistical analysis for non-synonymous mutations of each chromosome). Therefore, the preferential accumulation of non-optimal codons may implicate biological processes that are significant in cancers.

### Genes encoding hubs of protein interaction and signaling network tend to accumulate non-optimal codons

For the 17966 genes with somatic mutations that were identified in this study, we investigated the dynamics of optimal/non-optimal codons by comparing the number of N->O transformations with the number of O->N transformations. In accordance with the genome-wide accumulation of optimal codons, 7615 genes had not acquired non-optimal codons (O->N less than N->O) whereas 10351 genes had acquired one or more non-optimal codons (O->N more than N->O) (S11 Table).

**Fig 2:**
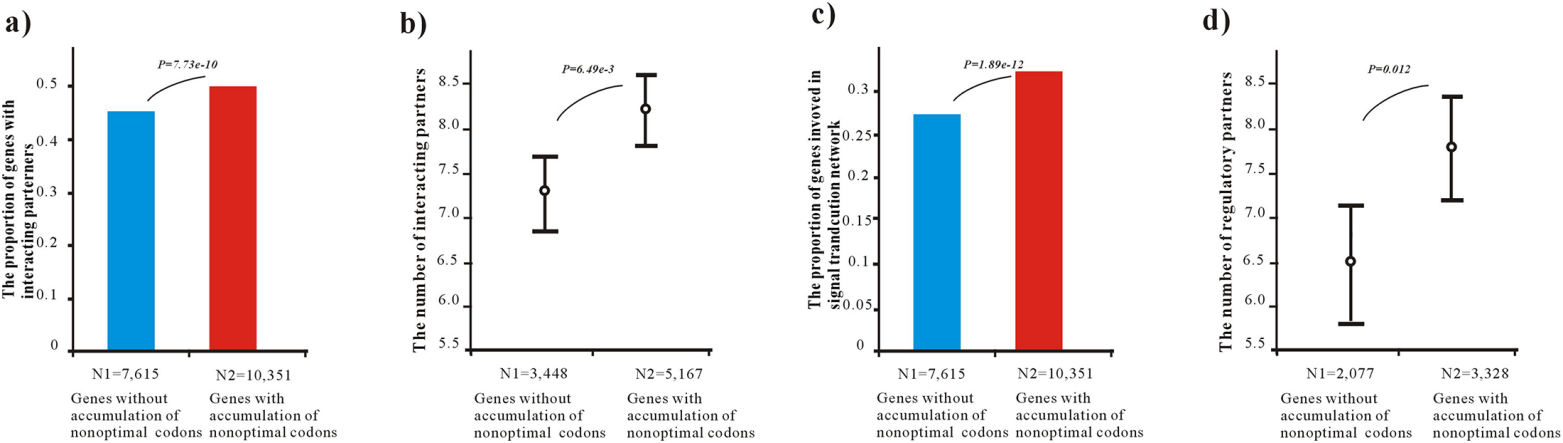
Genes with accumulation of non-optimal codons tend to be involved in protein interaction networks. (**a**) The comparison in the percentage of genes with protein interacting partners. The p-values were estimated by *Chi-square*, *two-tail test*. The N1 represents the number of genes without accumulation of non-optimal codons, and the N2 represents the number of genes with accumulation of non-optimal codons. (**b**) The comparison in the number of protein interacting partners of genes. The average degree was represented and the p-values were estimated by *Mann–Whitney U*, *two-tail test*. The N1 represents the number of genes without accumulation of non-optimal codons in the protein interaction networks, and the N2 represents the number of genes with accumulation of non-optimal codons in the protein interaction networks. (**c**) The comparison in the percentage of genes involved in cellular signal transduction network The p-values were estimated by *Chi-square*, *two-tail test*. The N1 represents the number of genes without accumulation of non-optimal codons, and the N2 represents the number of genes with accumulation of non-optimal codons. (**d**) The comparison in the number of regulatory partners. The average number was represented and the p-values were estimated by *Mann–Whitney U*, *two-tail test*. The N1 represents the number of genes without accumulation of non-optimal codons in the signal transduction networks, and the N2 represents the number of genes with accumulation of non-optimal codons in signal transduction networks.

We first studied how the genes that had accumulated non-optimal codons were distributed in the protein interaction network [44]. By transforming the ref protein ids to ensembl gene ids, interacting partners for the 8615 of 17966 genes were obtained from the Human Protein Reference Database [45]. We found that genes with accumulation of non-optimal codons tended to be involved in protein interaction networks. About 45.29% of genes without accumulation of non-optimal codons had interacting partners, while 49.91% for genes with accumulation of non-optimal codons had interacting partners (p = 7.73e-10, *Chi-square*, *two-tail test*) (Fig 2a). We then investigated the number of interacting partners (also referred to as degree) and found that genes with accumulation of non-optimal codons tended to have significantly higher numbers of interacting partners. As shown in Fig 2b, the average degree for genes without accumulation of non-optimal codons was 7.25, and the average degree increased to 8.1 for genes with accumulation of non-optimal codons (p = 6.49e-3, *Mann–Whitney U*, *two-tail test*).

Because different genes have distinct proportions of optimal codons in their transcripts, it is likely that genes encoding hubs of protein interaction networks may have significantly higher proportions of optimal codons and may, therefore, be more likely to accumulate non-optimal codons through cancer somatic mutations. To control this potential bias, the percentages of optimal codons in these genes were sorted from small to large, and then sampled sequentially into a new dataset until the average proportion of optimal codons of the sampled dataset equaled that of the genes without accumulation of non-optimal codons (S11 Table). Using this sampled dataset of genes that accumulated non-optimal codons and had the similar average proportions of optimal codons, the comparison also showed that the genes with accumulation of non-optimal codons had a higher proportion of genes involved in protein interaction networks (p = 1.21e-7, *Chi-square*, *two-tail test*) and a higher average number of interacting partners (p = 4.09e-3, *Mann–Whitney U*, *two-tail test*) (S1 Fig).

We further studied how the genes with accumulation of non-optimal codons were distributed in the signaling network. We obtained 5405 genes with CSM somatic mutations in the signaling transduction networks, which included 2077 genes without accumulation of non-optimal codons, and 3328 genes with accumulation of non-optimal codons. As shown in Fig 2c, about 32.15% of the genes with accumulation of non-optimal codons were involved in signaling networks, compared with 27.27% of the genes without accumulation of non-optimal codons (p = 1.89e-12, *Chi-square*, *two-tail test*). Furthermore, the number of regulatory partners for the genes with accumulation of non-optimal codons was also significantly higher than for the genes without accumulation of non-optimal codons (p = 0.012, *Mann–Whitney U*, *two-tail test*, Fig 2d). Using the sampled dataset of genes, similar tendencies were also observed; i.e., genes with accumulation of non-optimal codons had a higher proportion of genes involved in signal transduction networks (p = 8.69e-9, *Chi-square*, *two-tail test*) and a higher average number of regulatory partners (p = 0.015, *Mann–Whitney U*, *two-tail test*) (S1 Fig).

### Genes catalyzing the high flux reactions of metabolic network tend to accumulate non-optimal codons

**Fig 3:**
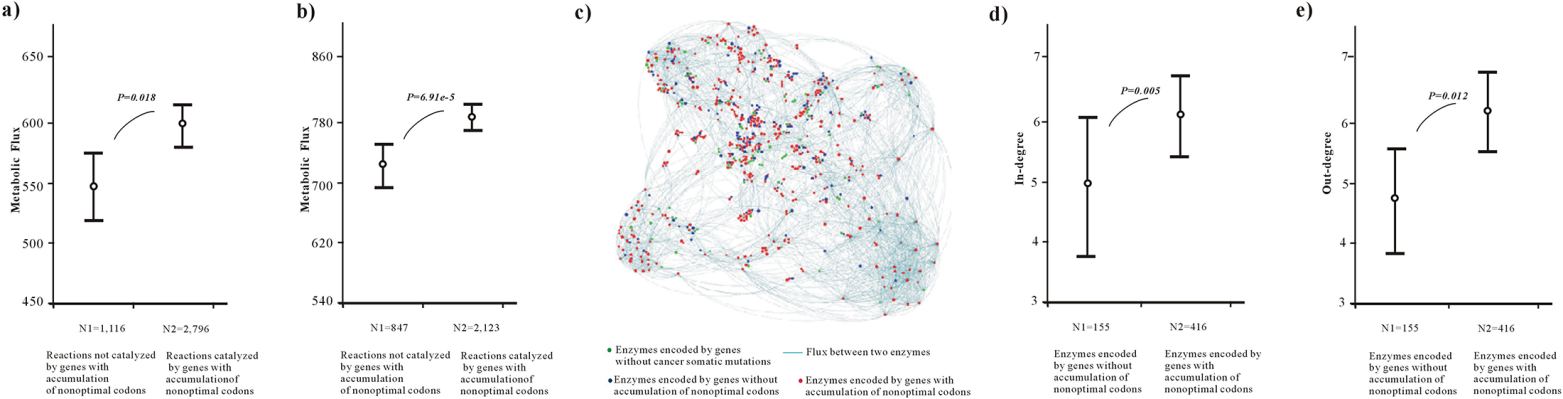
Genes with accumulation of non-optimal codons tend to be involved in high flux reactions in metabolic network. (**a**) Comparison of metabolic flux. N1 represents the number of reactions not catalyzed by genes with accumulation of non-optimal codons in Recon 2, and the N2 represents the number of reactions catalyzed by genes with accumulation of non-optimal codons in Recon 2. (**b**) Comparison of metabolic flux after filtering out null-flux. N1 represents the number of reactions not catalyzed by genes with accumulation of non-optimal codons in Recon 2 after filtering out null-flux, and the N2 represents the number of reactions catalyzed by genes with accumulation of non-optimal codons in Recon 2 after filtering out null-flux. (**c**) The largest sub-network of human enzyme-enzyme metabolic networks Red nodes represent the enzymes encoded by genes with accumulation of non-optimal codons. (**d**) Comparison of in-degree. N1 represents the number of enzymes encoded by genes without accumulation of non-optimal codons in enzyme-enzyme metabolic networks, and the N2 represents the number of enzymes encoded by genes with accumulation of non-optimal codons in enzyme-enzyme metabolic networks. (**e**) Comparison of out-degree. N1 represents the number of enzymes encoded by genes without accumulation of non-optimal codons in enzyme-enzyme metabolic networks, and the N2 represents the number of enzymes encoded by genes with accumulation of non-optimal codons in enzyme-enzyme metabolic networks. The average flux value and in/out-degree were represented. The p-values were estimated by comparisons between the genes without accumulation of non-optimal codons and the genes with accumulation of non-optimal codons (*Mann–Whitney U*, *two-tail test*).

We used the recently updated Recon 2 [29] to explore the reactions catalyzed by the genes with accumulation of non-optimal codons. Using the COBRA Toolbox [30], we performed a flux balance analysis of Recon 2 model and obtained flux values for 3912 metabolic reactions that were catalyzed by 1623 *ensembl* genes (see Methods). Correlation analysis showed a positive relationship between the flux values of reactions and the proportion of genes with accumulation of non-optimal codons for their enzyme encoding genes (rho = 0.106, p = 1.00e-6, *Spearman analysis*, *two-tail test*, n = 3912). This comparison confirmed that the genes with accumulation of non-optimal codons had significantly higher values of flux in the metabolic network (p = 0.018, *Mann–Whitney U*, *two-tail test*) (Fig 3a). The same tendency was also observed after excluding the null fluxes (correlation: rho = 0.109, p = 1.00e-6, *Spearman analysis*, *two-tail test*, n = 2970; comparison: p = 6.91e-5, *Mann–Whitney U*, *two-tail test*, Fig 3b). Obviously, the genes involved in the high-flux reactions tended to accumulate non-optimal codons in cancers.

Next, we studied the importance of genes with accumulation of non-optimal codons from the metabolic network point of view. A human enzyme-enzyme metabolic network was constructed using the Recon 2 model [29] (see Methods). In the large connected network, 571 enzymes were encoded by genes with somatic mutations, including 155 enzymes that were encoded by genes without accumulation of non-optimal codons and 416 enzymes which were encoded by genes with accumulation of non-optimal codons (Fig 3c). We used the topological centralities to measure the importance of the enzymes in the metabolic network (see Methods). As shown in Fig 3d and 3e, the enzymes encoded by genes with accumulation of non-optimal codons had significantly higher in-degree and out-degree values (p = 0.005, p = 0.012 respectively, *Mann–Whitney U*, *two-tail test*), indicating that they preferentially acted as hub enzymes in the metabolic network.

Using the sampled dataset of genes (S11 Table), the comparison also showed that genes with accumulation of non-optimal codons tended to catalyze the reactions with significantly higher flux values (p ≤ 6.37e-7, *Mann–Whitney U*, *two-tail test*) and encoded the hub enzymes in the metabolic network (p ≤ 0.004, *Mann–Whitney U*, *two-tail test*) (S2 Fig).

### Genes with accumulation of non-optimal codons tend to participate in cancer-related pathways

**Fig 4:**
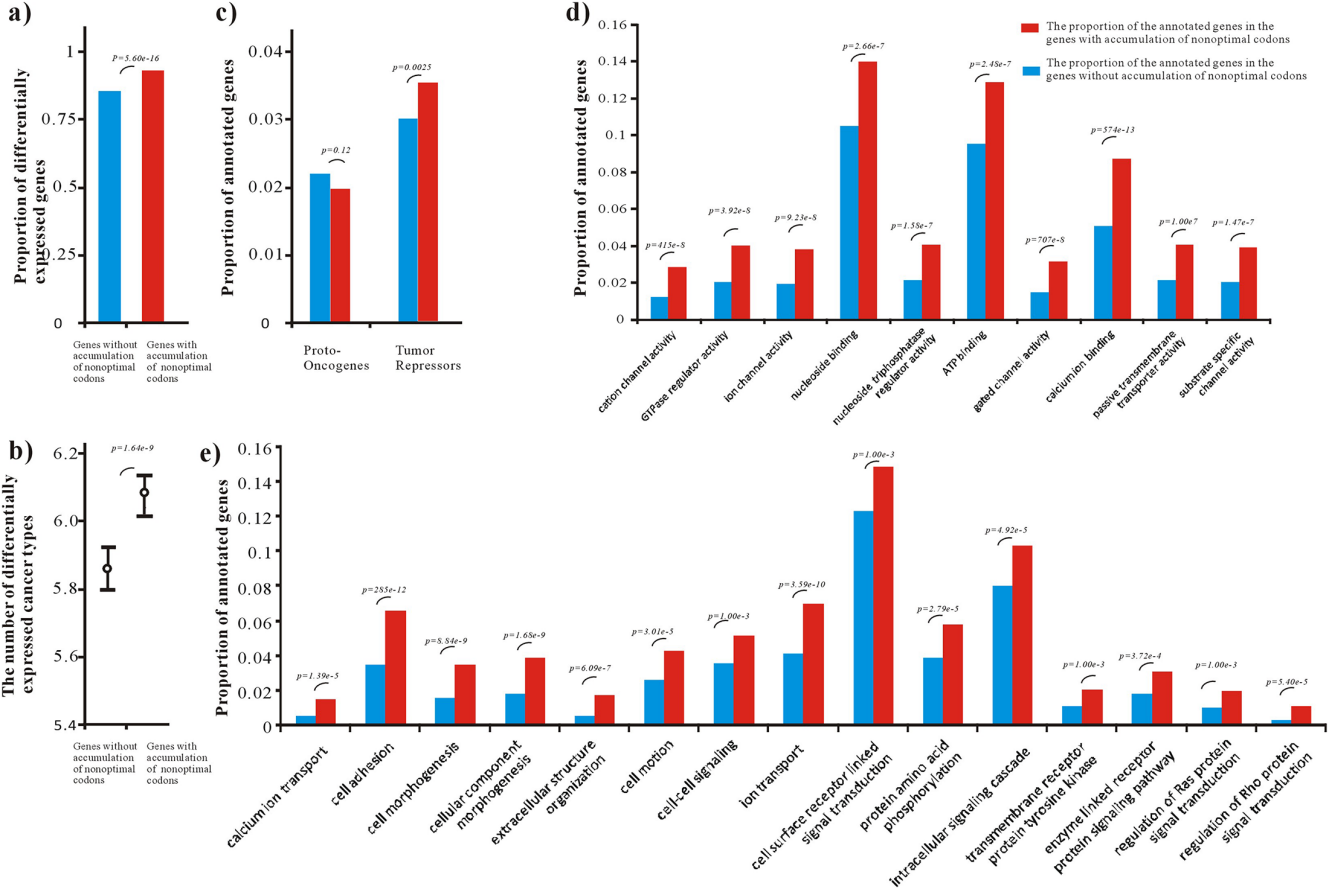
Function analyses of genes with and without accumulation of non-optimal codons. **(a)** Proportion of differentially expressed genes in cancers. The red box represents the proportion of differentially expressed genes in the genes with accumulation of non-optimal codons, the blue box represents the proportion of the proportion of differentially expressed genes in genes without accumulation of non-optimal codons. The p-values were estimated by *Chi-square*, *two-tail test*. **(b)** Number of cancer types for differentially expressed genes. The red box represents the number of differentially expressed cancer types for the genes with accumulation of non-optimal codons. The p-values were estimated by *Mann–Whitney U*, *two-tail test*. **(c)** Proportion of proto-oncogenes and tumor-repressors in the genes without accumulation of non-optimal codons, and the genes with accumulation of non-optimal codons. The red box represents the proportion of annotated genes in the genes with accumulation of non-optimal codons, the blue box represents the proportion of the proportion of annotated genes in genes without accumulation of non-optimal codons. The p-values were estimated by *Chi-square*, *two-tail test*. (d) Functional enrichment analysis of genes with and without accumulation of non-optimal codons annotated with GO terms under molecular function. The red box represents the proportion of annotated genes in the genes with accumulation of non-optimal codons, the blue box represents the proportion of the proportion of annotated genes in genes without accumulation of non-optimal codons. The p-values were estimated by *Hypergeometric test* and *Benjamini* corrected. (e) Functional enrichment analysis of genes with and without accumulation of non-optimal codons annotated with GO terms under biological process. The red box represents the proportion of annotated genes in the genes with accumulation of non-optimal codons, the blue box represents the proportion of the proportion of annotated genes in genes without accumulation of non-optimal codons. The p-values were estimated by *Hypergeometric test* and *Benjamini* corrected.

Generally, cancer somatic substitutions are identified by sequencing genes from healthy and tumor tissues of the same individuals. The variable substitution sites would be present at relatively high frequencies in the tumor. Based on the available microarray profiles of normal tissues (see Methods), a similar proportion of tissue-specific genes were observed in the genes with accumulation of non-optimal codons and the genes without accumulation of non-optimal codons (1518/4811 vs. 2208/6918, p = 0.68, *Chi-square*, *two-tail test*). We further explored the available differentially expressed transcripts in cancer RNA-Seq datasets (see Methods**)**, and found that genes with accumulation of non-optimal codons tended to be differentially expressed in cancers (p = 5.60e-16, *Chi-square*, *two-tail test*) (Fig 4a), and had a significantly higher average number of differential expressed tissues (p = 1.64e-9, *Mann–Whitney U*, *two-tail test*) (Fig 4b).

We then studied the codon dynamics in two major groups of protein-coding genes, proto-oncogenes and tumor repressor genes. For proto-oncogenes, gain of function activated by point mutations can stimulate cell proliferation and promote cell survival by interfering with apoptosis [46]. For tumor suppressor genes, loss of function can contribute to the development of cancer [47] (see Methods and S12 Table). As shown in Fig 4c, the tumor repressor genes tended to have a significantly higher percentage in the genes with accumulation of the non-optimal codons (p = 0.0025, *Chi-square*, *two-tail test*), while the proto-oncogenes tended to have a similar proportion of genes with accumulation of the non-optimal codons and the genes without accumulation of non-optimal codons (p = 0.12, *Chi-square*, *two-tail test*).

We used the Gene Ontology (GO) to explore the functional pathways that the genes with accumulation of non-optimal codons were involved in. Of the 10351 genes with accumulation of optimal codons, 7502 genes were annotated with GO terms under the biological process category; and 7076 genes were annotated with GO terms under the molecular function category. We performed a GO functional analysis to determine whether the genes with accumulation of non-optimal codons encoded proteins that were enriched with specific molecular functions or particular biological processes. (see Methods). As shown in Fig 4d and 4e, genes with accumulation of non-optimal codons were enriched in cell adhesion, cell motility, cell-cell signaling, anatomical structure morphogenesis, cell surface receptor linked signal transduction, angiogenesis, protein amino acid phosphorylation, extracellular transport. These processes were generally considered to be environment-oriented and well-known to be cancer-related. For instances, reduced intercellular adhesiveness made it possible for cancer cells to disobey the social order, and lead to destruction of histological structure [48] Up-regulation of the motility machine pathways contributed to tumor cells’ invasion of neighboring extracellular matrix tissue and the lymphatic system [49], Ion channels regulate cell cycle and differentiation by controlling membrane potential and interaction between the extracellular matrix and cytoskeleton [50]. Although the group of oncogenes were not found to accumulate the non-optimal codons or optimal codons, their regulatory genes tend to accumulate the non-optimal codons (GO:0046578, regulation of Ras protein signal transduction). Therefore, the genes with accumulation of non-optimal codons may participate in dysfunctional transduction of a large variety of external signals in response to a wide range of cellular responses.

### Accumulation of non-optimal codons tends to favor amino acids with higher aggregation and lower disorder properties

The proper three-dimensional structures were usually pre-requested to form the protein interaction interfaces and catalytic cavities. In the normal cell, protein folded into stable globular conformations and competed with aggregation into non-functional insoluble structures, because the biophysical properties of folding also favored intermolecular contacts [51,52]. Recent research indicated cancer as an aggregation disease, the destabilized p53 mutant induced misfolding and co-aggregation of wild-type p53, p63 and p73 into cellular inclusions, and lead to inefficiency of target genes that control cell growth [53,54]. Another important prosperity of protein structure is the intrinsically disordered region, which widely act as flexible linkers connecting two domains and served as switches in transforming to ordered conformation [55,56]. Recent study reported that disease mutations often destroyed the intrinsic disorder regions of human proteins in the etiology of diseases [57].

We studied the potential effects of N->O and O->N non-synonymous mutations on protein structures at the level of aggregation disorder propensity (see Methods). We found that 65.78% of O->N transformations would result in amino acids with higher aggregation propensity, which was significantly higher than the 53.22% obtained for N->O non-synonymous transformations (p = 1.99e-19, Chi-square, two-tail test). Similarly, 47.00% of the O->N transformations would lead to amino acids with lower disorder scores, which was significantly higher than the 31.70% observed for N->O non-synonymous transformations (p = 1.22e-26, *Chi-square*, *two-tail test*) (S13 Table).

### Codon dynamics in COSMIC and GWAS datasets

The accumulation of non-optimal codons in cancer genomes was confirmed by examining the data in the COSMIC database (ftp://ftp.sanger.ac.uk/pub/CGP/cosmic/data_export/, version67), freely available resource of associations between somatic mutations and cancers [36]. For the 299028 recorded ‘confirmed somatic mutations’ that occurred in codons, cancer mutations contained significantly higher frequencies of O->N transformations and lower frequencies of N->O transformations (p ≤ 2.19e-9 for non-synonymous mutations and p ≤ 2.43e-17 for synonymous mutations, *Chi-square*, *two-tail test*, S14 Table). A total of 43959 non-optimal codons (13272–3211+43791–9893) were accumulated in cancer genomes, which corresponded to 14.70% (43959/299028) of the ‘confirmed somatic mutations’, and is similar to the proportion observed in CSM datasets. For a specific group of 1105 COSMIC “recurrent” mutations that were implicated as drivers in the tumorigenesis process, 513, 197, 42, and 353 were observed for the O->O, O->N, N->O and N->N codon transformations, respectively, with a proportion of 14% ((197–42)/1105) accumulation of non-optimal codons.

We used the GWAS diseases-SNPs data to investigate single nucleotide polymorphisms (SNPs) that occurred in protein-coding regions to gain further insights into their codon dynamics. We found 6310 non-cancer-related GWAS-SNPs that were located in the gene regions (including 5’UTR, Coding Region, 3’UTR and introns); 402 of them were located in coding regions that exhibited codon dynamics and only 23 non-optimal codons were accumulated (O->O,175; O->N, 66; N->O, 43; N->N, 118) and corresponded to a proportion as 5.70% by 23/402, which was significantly lower than the proportion observed in the CSM datasets (23/402 *vs*. 20913/135760, *p<0*.*01*, *Chi-square*, *two-tail test*). Thus we found that the accumulation of non-optimal codon was not significant in the GWAS coding SNPs.

## Discussion

In this study, we showed that non-optimal codons were preferentially accumulated through somatic mutations in human cancers. The pattern [58,59] and the functional impact [60] of somatic mutations have been investigated extensively in the past decade; however, the transformations of codons themselves are far less studied. Synonymous mutations were often ignored by traditional studies because the same amino acids were conserved. In an early study, a likelihood ratio test (the classical Ka/Ks test) was developed to estimate the fixation of the mutations in cancer progenitor cells [61]. In this study, we used the codon—anticodon binding affinities-based classification of codons. This classification schema has two advantages. One advantage is that the partition of optimal and non-optimal codons is based on the binding free energy between codons and anticodons at the translational stage, and the set of optimal codons is independent on the species or cell status. The other advantage is that codons with low codon anticodon binding affinities (non-optimal codons) were recently found to be related to the ability genes to control the cell cycle [8], which is closely related to tumors. We used this classification to comprehensively investigate the dynamics of codons in cancers, and demonstrated that the majority of genes accumulated non-optimal codons with both synonymous and non-synonymous mutations. We also showed that genes with accumulation of non-optimal codons tended to participate in biological pathways associated with cell-cell communication and cell motility, the dysfunction of which was frequently associated with carcinogenesis.

It is interesting that the accumulation of non-optimal codons seemed to be favored in cancer genomes where the balance between proliferation and cell death is generally upset [62,63]. Non-optimal codons were found to provide their resident genes with more opportunities to change in the tRNA pool and generate cell cycle-dependent oscillations of protein abundance [8]. Our study indicated that the accumulation of non-optimal codons may be an adaptive strategy for cancer cellular competition for survival. Rapid progress in the understanding of human genetic variations has indicated that tumorigenesis can be studied within a cellular “mutation vs. fitness and evolution vs. selection” framework [18]. In normal tissues, the immune system exerts pressures on cells, and tissue compartmentalization constrains cells from abnormal proliferation [19–21]. Exposure to external genotoxic stress or environmental chemicals [64,65] can cause the accumulation of non-optimal codons, which may enable an individual cell to evade selective pressures and gain cellular fitness over normal cells, and provided positive selectiveness for these cells.

The pattern of somatic mutations was also investigated in oncogenes and tumor repressor genes. The results indicated that the accumulation of non-optimal codons mainly had an adaptive role in the non-controlled cycle of tumor cells with the trade-off being loss of some important functions, but did not provide the original driving force in “gain of function” for tumor occurrence. Recently, Ostrow *et al* identified positively selected genes and suggested that cancer evolution was related with positive selection on globally expressed genes [66]. Our result may complement the pattern of cancer genetic codons; that is, while some driver genes (generally oncogenes) can gain new functions by positive selection, a majority of genes with accumulation of non-optimal codons tended be to differentially expressed in cancers, and became more adaptive to the non-controlled cell cycle in tumors [67–69].

**Fig 5:**
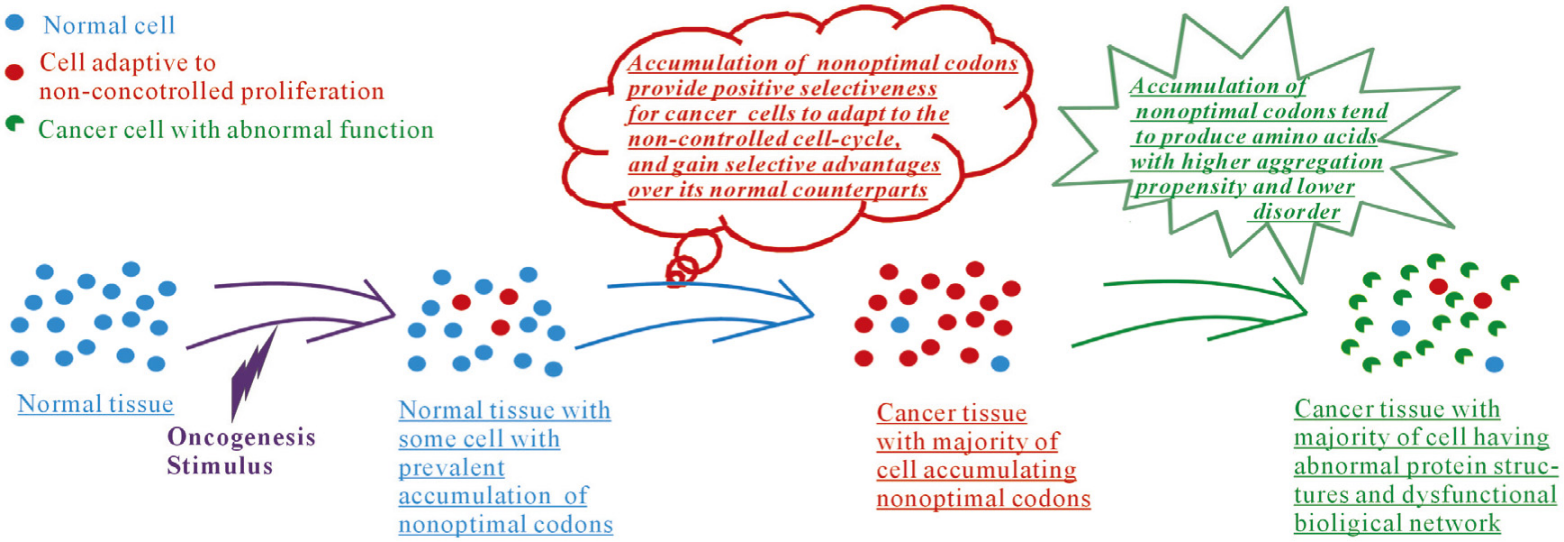
Schematic representation of the dual roles of O->N transformations during the tumorigenesis.

Based on our analyses, we propose that the preference of O->N codon transformations may play dual roles in cancers (Fig 5). During tumorigenesis, this is like an evolutionary dynamics of normal cells and cancer cells with the phenotypic variability. The accumulation of non-optimal codons promotes ability of the tumor cell to adapt to non-controlled proliferative cell cycle, and leads to modification of the original biological networks and consequently stimulates the occurrence of dysfunctional modules. Therefore, we consider that in future anti-cancer studies more attention should be given to the mechanisms that affect the transformation of codons. This is a genome-wide integrative analysis of cancer mutations within the framework of optimal/non-optimal codon transformations. A better understanding of the roles of non-optimal codons will be valuable for studying the impact of mutations on human health.

## Supporting Information

S1 Fig Genes with accumulation of non-optimal codons tend to be involved in protein interaction and signaling network. (**a**) The comparison in the percentage of genes with protein interacting partners, the p-values were estimated by *Chi-square*, *two-tail test*. The N1 represents the number of genes without accumulation of non-optimal codons, and the N2 represents the number of genes with accumulation of non-optimal codons. (**b**) The comparison in the number of protein interacting partners of genes, the average degree was represented and the p-values were estimated by *Mann–Whitney U*, *two-tail test*. The N1 represents the number of genes without accumulation of non-optimal codons in the protein interaction networks, and the N2 represents the number of genes with accumulation of non-optimal codons in the protein interaction networks. (c) The comparison in the percentage of genes involved in cellular signal transduction network, the p-values were estimated by *Chi-square*, *two-tail test*. The N1 represents the number of genes without accumulation of non-optimal codons, and the N2 represents the number of genes with accumulation of non-optimal codons. (d) The comparison in the number of regulatory partners, the average number was represented and the p-values were estimated by *Mann–Whitney U*, *two-tail test*. The N1 represents the number of genes without accumulation of non-optimal codons in the signal transduction networks, and the N2 represents the number of genes with accumulation of non-optimal codons in the signal transduction networks. The genes with accumulation of non-optimal codons were sampled to have a similar average proportion of optimal codons with the genes without accumulation of non-optimal codons.
(TIF)

S2 Fig Genes with accumulation of non-optimal codons tend to be involved in high flux reactions in metabolic network. (**a**) Comparison of metabolic flux. N1 represents the number of reactions not catalyzed by genes with accumulation of non-optimal codons in Recon 2, and the N2 represents the number of reactions catalyzed by genes with accumulation of non-optimal codons in Recon 2. (**b**) Comparison of metabolic flux after filtering out null-flux. N1 represents the number of reactions not catalyzed by genes with accumulation of non-optimal codons in Recon 2 after filtering out null-flux, and the N2 represents the number of reactions catalyzed by genes with accumulation of non-optimal codons in Recon 2 after filtering out null-flux. (**c**) Comparison of in-degree. N1 represents the number of enzymes encoded by genes without accumulation of non-optimal codons in enzyme-enzyme metabolic networks, and the N2 represents the number of enzymes encoded by genes with accumulation of non-optimal codons in enzyme-enzyme metabolic networks. (**d**) Comparison of out-degrees. N1 represents the number of enzymes encoded by genes without accumulation of non-optimal codons in enzyme-enzyme metabolic networks, and the N2 represents the number of enzymes encoded by genes with accumulation of non-optimal codons in enzyme-enzyme metabolic networks. The average flux value, in-degree and out-degree were represented, and the p-values were estimated by *Mann–Whitney U*, *two-tail test*. The genes with accumulation of non-optimal codons were sampled to have a similar average proportion of optimal codons with the genes without accumulation of non-optimal codons.
(TIF)

S1 Table The source files for each type of cancers. (PDF)

S2 Table Somatic mutations of codons in cancers. (XLSX)

S3 Table Variation of codons between Chimp-Human orthologes. (XLSX)

S4 Table Variation of codons among human populations. (XLSX)

S5 Table Synonymous O->N transformations are significantly enriched in each type of cancers. The p-values were estimated by *Chi-square*, *two-tail test*.
(PDF)

S6 Table Non-synonymous O->N transformations are significantly enriched in each type of cancers. The p-values were estimated by *Chi-square*, *two-tail test*.
(PDF)

S7 Table Synonymous O->N transformations are significantly enriched in each type of amino acids. The p-values were estimated by *Chi-square*, *two-tail test*.
(PDF)

S8 Table Non-synonymous O->N transformations are significantly enriched in each type of amino acids. The p-values were estimated by *Chi-square*, *two-tail test*.
(PDF)

S9 Table Synonymous O->N transformations are significantly enriched in each type of chromosomes. The p-values were estimated by *Chi-square*, *two-tail test*.
(PDF)

S10 Table Non-synonymous O->N transformations are significantly enriched in each type of chromosomes. The p-values were estimated by *Chi-square*, *two-tail test*.
(PDF)

S11 Table The list of the genes with accumulation of optimal codons, the genes with accumulation of non-optimal codons, the sampled genes with accumulation of non-optimal codons. As the genes with accumulation of non-optimal codons have a significantly higher average proportion of optimal codons than the genes without accumulation of non-optimal codons, a subset of genes with accumulation of non-optimal codons were the sampled to have a similar average proportion of optimal codons with the genes without accumulation of non-optimal codons.
(PDF)

S12 Table The list of 362 proto-oncogenes and 608 tumor repressors with somatic mutations identified in CSM. (PDF)

S13 Table The variation of aggregation and disorder scores for non-synonymous O->N and N->O transformations. The calculation of aggregation and disorder were based on gene unit, the p-values were estimated by *Chi-square*, *two-tail test*.
(PDF)

S14 Table The frequencies of O->N and N->O transformations in COSMIC v67 somatic mutations. The p-values were estimated by *Chi-square*, *two-tail test*.
(PDF)
