# Supplementary figures and images for: Prevalent Accumulation of Non-Optimal Codons through Somatic Mutations in Human Cancers
Source: PLoS One. 2016 Aug 11;11(8):e0160463. doi: 10.1371/journal.pone.0160463 (PMC4981346; doi:10.1371/journal.pone.0160463)

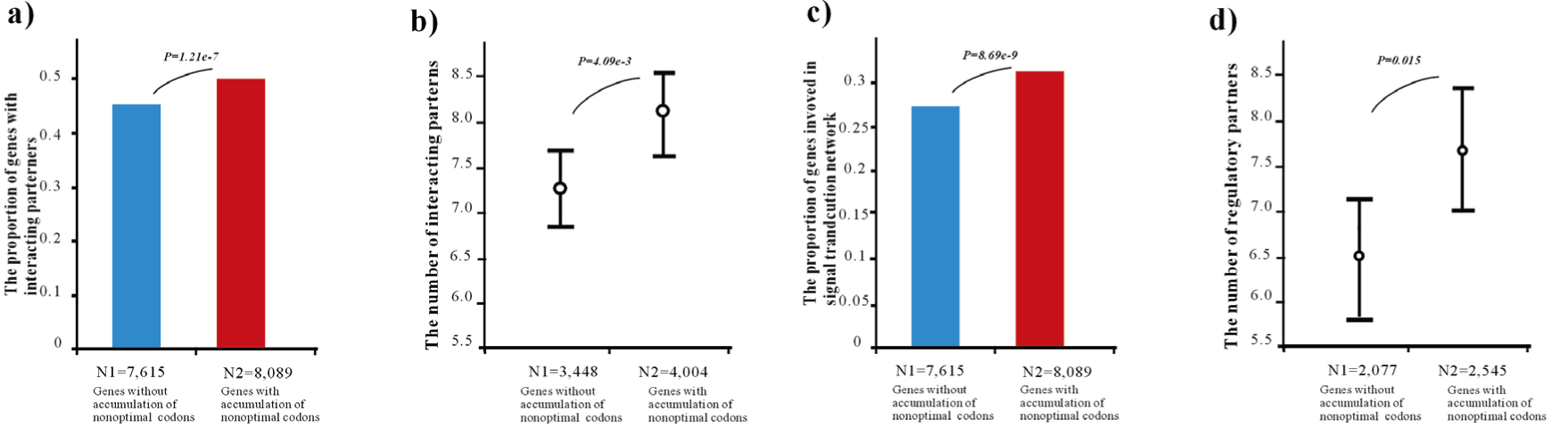

Supplement: S1 Fig — (a) The comparison in the percentage of genes with protein interacting partners, the p-values were estimated by Chi-square, two-tail test. The N1 represents the number of genes without accumulation of non-optimal codons, and the N2 represents the number of genes with accumulation of non-optimal codons. (b) The comparison in the number of protein interacting partners of genes, the average degree was represented and the p-values were estimated by Mann–Whitney U, two-tail test. The N1 represents the number of genes without accumulation of non-optimal codons in the protein interaction networks, and the N2 represents the number of genes with accumulation of non-optimal codons in the protein interaction networks. (c) The comparison in the percentage of genes involved in cellular signal transduction network, the p-values were estimated by Chi-square, two-tail test. The N1 represents the number of genes without accumulation of non-optimal codons, and the N2 represents the number of genes with accumulation of non-optimal codons. (d) The comparison in the number of regulatory partners, the average number was represented and the p-values were estimated by Mann–Whitney U, two-tail test. The N1 represents the number of genes without accumulation of non-optimal codons in the signal transduction networks, and the N2 represents the number of genes with accumulation of non-optimal codons in the signal transduction networks. The genes with accumulation of non-optimal codons were sampled to have a similar average proportion of optimal codons with the genes without accumulation of non-optimal codons. (TIF) [file pone.0160463.s001.tif]

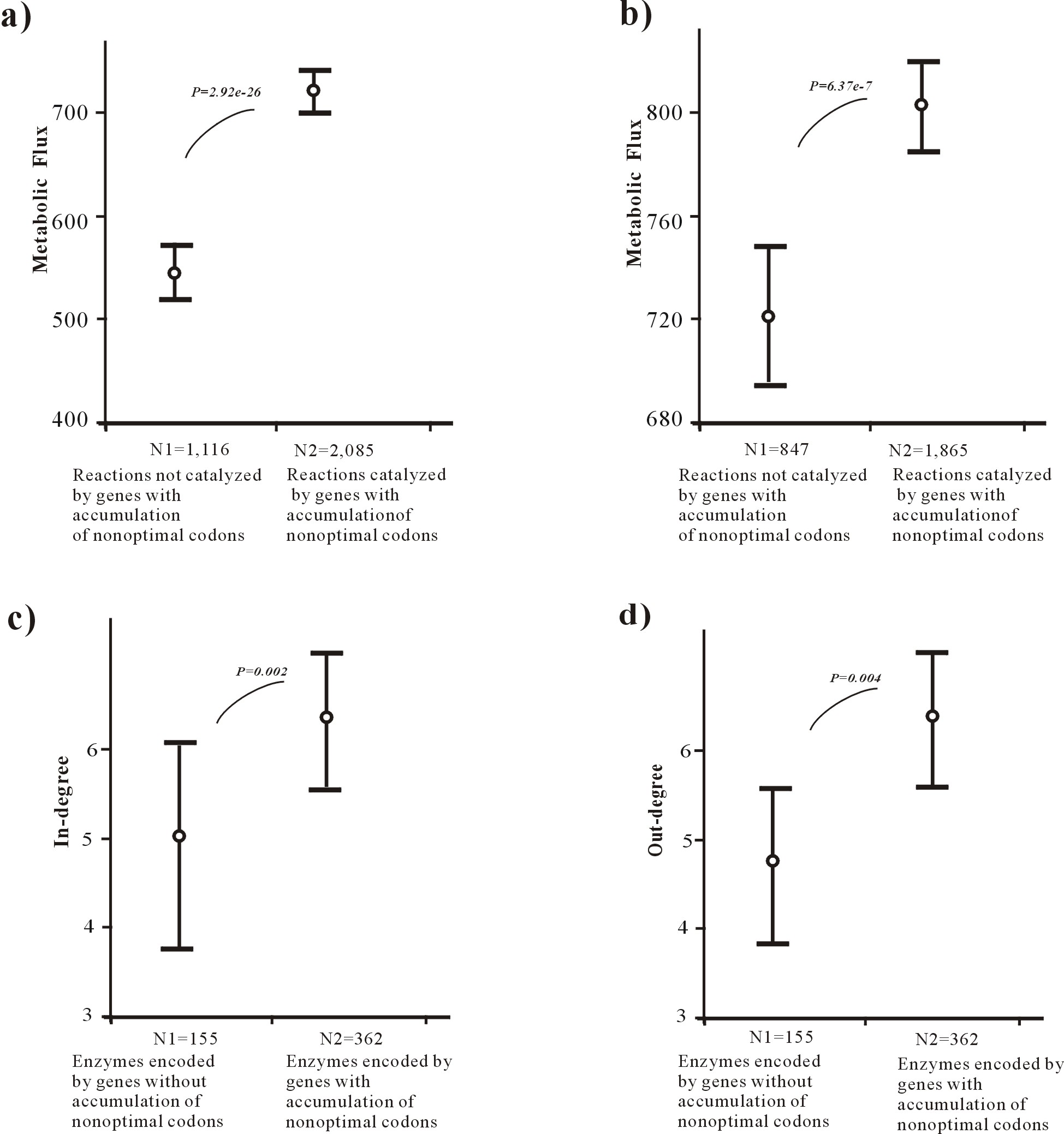

Supplement: S2 Fig — (a) Comparison of metabolic flux. N1 represents the number of reactions not catalyzed by genes with accumulation of non-optimal codons in Recon 2, and the N2 represents the number of reactions catalyzed by genes with accumulation of non-optimal codons in Recon 2. (b) Comparison of metabolic flux after filtering out null-flux. N1 represents the number of reactions not catalyzed by genes with accumulation of non-optimal codons in Recon 2 after filtering out null-flux, and the N2 represents the number of reactions catalyzed by genes with accumulation of non-optimal codons in Recon 2 after filtering out null-flux. (c) Comparison of in-degree. N1 represents the number of enzymes encoded by genes without accumulation of non-optimal codons in enzyme-enzyme metabolic networks, and the N2 represents the number of enzymes encoded by genes with accumulation of non-optimal codons in enzyme-enzyme metabolic networks. (d) Comparison of out-degrees. N1 represents the number of enzymes encoded by genes without accumulation of non-optimal codons in enzyme-enzyme metabolic networks, and the N2 represents the number of enzymes encoded by genes with accumulation of non-optimal codons in enzyme-enzyme metabolic networks. The average flux value, in-degree and out-degree were represented, and the p-values were estimated by Mann–Whitney U, two-tail test. The genes with accumulation of non-optimal codons were sampled to have a similar average proportion of optimal codons with the genes without accumulation of non-optimal codons. (TIF) [file pone.0160463.s002.tif]
