# Supplementary material for: Prevalent Accumulation of Non-Optimal Codons through Somatic Mutations in Human Cancers
Source: PLoS One. 2016 Aug 11;11(8):e0160463. doi: 10.1371/journal.pone.0160463 (PMC4981346; doi:10.1371/journal.pone.0160463)
Supplement: S1 Table — (PDF) [file pone.0160463.s003.pdf]

Cancers	Source Files
Acute Myeloid Leukemia	ssm.tcgaLAML.txt
Breast Cancer	ssm.hopkinsBreast.txt
Breast Carcinoma	ssm.sangerBreast.txt
Breast Invasive Carcinoma	ssm.tcgaBRCA.txt
Chronic Lymphocytic Leukemia	ssm.esCLL.txt
Colon Adenocarcinoma	ssm.tcgaCOAD.txt
Colorectal Cancer	ssm.hopkinsColon.txt
Gastric Cancer	ssm.ccgcGastric.txt
Glioblastoma Multiformet;	ssm.hopkinsGBM.txt; ssm.tcgaGBM.tx
Liver Cancer	ssm.frLiver.txt; ssm.jpNCCLiver.txt; ssm.jpRikenLiver.txt
Lung Adenocarcinoma	ssm.tspLung.txt
Lung Squamous Cell Carcinoma	ssm.tcgaLUSC.txt;
Malignant Lymphoma	ssm.deMMML.txt
Malignant Melanoma	ssm.sangerMelanoma.txt
Myeloproliferative Disorders	ssm.sangerMDS.txt
Ovarian Serous Cystadenocarcinoma	ssm.tcgaOV.txt
Pancreatic Cancer	ssm.hopkinsPanc.txt; ssm.oicrPanc.txt; ssm.QCMGPancreas.txt
Pediatric Brain Tumors	ssm.dePedbrain.txt
Prostate Cancer	ssm.caProstate.txt
Rectum Adenocarcinoma	ssm.tcgaREAD.txt
Small Cell Lung Carcinoma	ssm.sangerLung.txt
