# Supplementary material for: Prevalent Accumulation of Non-Optimal Codons through Somatic Mutations in Human Cancers
Source: PLoS One. 2016 Aug 11;11(8):e0160463. doi: 10.1371/journal.pone.0160463 (PMC4981346; doi:10.1371/journal.pone.0160463)
Supplement: S5 Table — The p-values were estimated by Chi-square, two-tail test. (PDF) [file pone.0160463.s007.pdf]

	Datasets	O->N	N->O	Fold	p-values [#1]	p-values [#2]
Back-ground	Ortholog-Poly	45,212	45,127	1.00		
	SNP-Poly	9,186	6,434	1.43		
CSM	Colon_Adenocarcinoma	9,831	2,163	4.55	*0*	*0*
	Ovarian_Serous_Cystadenocarcinoma	4,157	1,204	3.45	*0*	*1.4E-133*
	Liver_Cancer	3,613	2,067	1.75	*1.5957E-87*	*2.52E-10*
	Rectum_Adenocarcinoma	1,562	317	4.93	*2.695E-177*	*4.79E-93*
	Breast_Carcinoma	1,148	196	5.86	*3.734E-146*	*5.13E-82*
	Breast_Invasive_Carcinoma	1,031	177	5.82	*2.983E-131*	*5.57E-74*
	Gastric_Cancer	990	604	1.64	*1.337E-21*	*0.010716*
	Acute_Myeloid_Leukemia	768	137	5.61	*1.7515E-96*	*1.17E-54*
	Pancreatic_Cancer	705	177	3.98	*7.7851E-70*	*1.32E-35*
	Glioblastoma_Multiforme	697	98	7.11	*4.622E-99*	*3.69E-59*
	Malignant_Lymphoma	146	32	4.56	*1.5449E-17*	*3.78E-10*
	Prostate_Cancer	125	98	1.28	*0.07316593*	*0.406541*
	Malignant_Melanoma	81	6	13.50	*9.8901E-16*	*8.83E-11*
	Lung_Squamous_Cell_Carcinoma	31	9	3.44	*0.000517*	*0.016434*
	Pediatric_Brain_Tumors	29	9	3.22	*0.00120392*	*0.0285*
	Small_Cell_Lung_Carcinoma	23	6	-	-	-
	Chronic_Lymphocytic_Leukemia	11	12	-	-	-
	Myeloproliferative_Disorders	10	2	-	-	-
	Breast_Cancer	3	0	-	-	-
	Colorectal_Cancer	3	2	-	-	-
	Small_Cell_Lung_Carcinoma	23	6	-	-	-

The p-values[#1] were obtained from the comparison in folds of O->N/N->O between the CSM and Ortholog-Poly, the p-values[#2] were obtained from the comparison in folds of O->N/N->O between the CSM and SNP-Poly. The datasets with a total number of O->N and N->O larger than 30 were analyzed, the p-values $\leq$ 0.05 were represented by red color and indicate significant higher number of O->N than N->O in CSM considering the distribution from the control datasets.
