# Supplementary material for: Prevalent Accumulation of Non-Optimal Codons through Somatic Mutations in Human Cancers
Source: PLoS One. 2016 Aug 11;11(8):e0160463. doi: 10.1371/journal.pone.0160463 (PMC4981346; doi:10.1371/journal.pone.0160463)
Supplement: S6 Table — The p-values were estimated by Chi-square, two-tail test. (PDF) [file pone.0160463.s008.pdf]

	Datasets	O->N	N->O	Fold	p-values [#1]	p-values [#2]
**Back-ground**	Ortholog-Poly	1,856	1,754	1.06		
	SNP-Poly	426	341	1.25		
**CSM**	Colon_Adenocarcinoma	1,819	617	2.95	*9.16E-74*	*6.031E-24*
	Ovarian_Serous_Cystadenocarcinoma	973	247	3.94	*1.31E-67*	*1.156E-30*
	Liver_Cancer	859	336	2.56	*3.69E-35*	*1.087E-13*
	Rectum_Adenocarcinoma	398	148	2.69	*6.00E-21*	*1.453E-10*
	Breast_Carcinoma	216	39	5.54	*6.82E-25*	*6.931E-17*
	Breast_Invasive_Carcinoma	203	31	6.55	*8.25E-26*	*5.224E-18*
	Pancreatic_Cancer	112	33	3.39	*1.02E-09*	*1.104E-06*
	Acute_Myeloid_Leukemia	90	15	6.00	*3.99E-12*	*3.644E-09*
	Lung_Adenocarcinoma	66	3	22.00	*3.15E-13*	*8.857E-11*
	Gastric_Cancer	57	35	1.63	*0.045*	*0.241*
	Glioblastoma_Multiforme	42	8	5.25	*4.64E-06*	*8.093E-05*
	Chronic_Lymphocytic_Leukemia	40	15	2.67	*0.0016922*	*0.0129654*
	Pediatric_Brain_Tumors	37	8	4.63	*3.94E-05*	*0.0004417*
	Breast_Cancer	26	5	5.20	*0.000317*	*0.0018057*
	Malignant_Lymphoma	22	10	2.20	*0.050*	*0.140*
	Colorectal_Cancer	16	9	-	-	-
	Prostate_Cancer	16	8	-	-	-
	Small_Cell_Lung_Carcinoma	11	1	-	-	-
	Malignant_Melanoma	7	2	-	-	-
	Lung_Squamous_Cell_Carcinoma	5	3	-	-	-
	Myeloproliferative_Disorders	2	0	-	-	-

The p-values[#1] were obtained from the comparison in folds of O->N/N->O between the CSM and Ortholog-Poly, the p-values[#2] were obtained from the comparison in folds of O->N/N->O between the CSM and SNP-Poly. The datasets with a total number of O->N and N->O larger than 30 were analyzed, the p-values $\leq$ 0.05 were represented by red color and indicate significant higher number of O->N than N->O in CSM considering the distribution from the control datasets.
