# Supplementary material for: Prevalent Accumulation of Non-Optimal Codons through Somatic Mutations in Human Cancers
Source: PLoS One. 2016 Aug 11;11(8):e0160463. doi: 10.1371/journal.pone.0160463 (PMC4981346; doi:10.1371/journal.pone.0160463)
Supplement: S7 Table — The p-values were estimated by Chi-square, two-tail test. (PDF) [file pone.0160463.s009.pdf]

Amino Acids	Datasets	Optimal to Non-ptimal	Non-optimal to Optimal	Fold	p-values [#1]	p-values [#2]
S	**Ortholog-Poly**	4,634	4,675	0.99		
	**SNP-Poly**	969	645	1.50		
	**CSM**	2,633	775	3.40	*2.64E-169*	*1.03E-36*
A	**Ortholog-Poly**	4,800	4,932	0.97		
	**SNP-Poly**	944	657	1.44		
	**CSM**	2,238	605	3.70	*8.06E-170*	*1.15E-44*
T	**Ortholog-Poly**	3,936	4,084	0.96		
	**SNP-Poly**	899	602	1.49		
	**CSM**	2,236	606	3.69	*5.56E-165*	*2.00E-39*
P	**Ortholog-Poly**	5,019	5,074	0.99		
	**SNP-Poly**	932	637	1.46		
	**CSM**	2,092	679	3.08	*5.13E-129*	*1.52E-28*
L	**Ortholog-Poly**	3,438	3,443	1.00		
	**SNP-Poly**	690	465	1.48		
	**CSM**	2,086	550	3.79	*7.50E-147*	*2.22E-35*
G	**Ortholog-Poly**	2,898	3,047	0.95		
	**SNP-Poly**	484	361	1.34		
	**CSM**	2,003	612	3.27	*3.46E-127*	*1.81E-27*
V	**Ortholog-Poly**	2,003	2,024	0.99		
	**SNP-Poly**	438	301	1.46		
	**CSM**	1,523	404	3.77	*9.80E-103*	*3.86E-25*
I	**Ortholog-Poly**	1,548	1,445	1.07		
	**SNP-Poly**	381	207	1.84		
	**CSM**	1,396	182	7.67	*2.29E-134*	*2.63E-37*
D	**Ortholog-Poly**	2,603	2,490	1.05		
	**SNP-Poly**	561	377	1.49		
	**CSM**	1,196	324	3.69	*3.56E-81*	*7.58E-24*
R	**Ortholog-Poly**	1,888	1,898	0.99		
	**SNP-Poly**	350	253	1.38		
	**CSM**	1,155	341	3.39	*2.42E-73*	*1.15E-18*
F	**Ortholog-Poly**	1,318	1,259	1.05		
	**SNP-Poly**	292	193	1.51		
	**CSM**	1,099	209	5.26	*9.46E-89*	*6.54E-27*
N	**Ortholog-Poly**	1,859	1,736	1.07		
	**SNP-Poly**	429	317	1.35		
	**CSM**	858	268	3.20	*1.08E-47*	*1.31E-17*
E	**Ortholog-Poly**	2,031	1,985	1.02		
	**SNP-Poly**	348	267	1.30		
	**CSM**	773	459	1.68	*6.80E-14*	*1.07E-02*
Y	**Ortholog-Poly**	1,737	1,589	1.09		
	**SNP-Poly**	387	275	1.41		

	**CSM**	771	225	3.43	*2.54E-45*	*1.80E-16*
	**Ortholog-Poly**	986	959	1.03		
**C**	**SNP-Poly**	192	121	1.59		
	**CSM**	768	205	3.75	*7.99E-49*	*4.90E-10*
	**Ortholog-Poly**	1,421	1,418	1.00		
**K**	**SNP-Poly**	266	215	1.24		
	**CSM**	687	367	1.87	*3.86E-17*	*2.15E-04*
	**Ortholog-Poly**	1,541	1,457	1.06		
**Q**	**SNP-Poly**	282	295	0.96		
	**CSM**	657	282	2.33	*1.56E-23*	*2.15E-16*
	**Ortholog-Poly**	1,552	1,612	0.96		
**H**	**SNP-Poly**	342	246	1.39		
	**CSM**	603	204	2.96	*5.16E-39*	*6.46E-11*

The p-values[#1] were obtained from the comparison in folds of O->N/N->O between the CSM and Ortholog-Poly, the p-values[#2] were obtained from the comparison in folds of O->N/N->O between the CSM and SNP-Poly. The datasets with a total number of O->N and N->O larger than 30 were analyzed, the p-values $\leq$ 0.05 were represented by red color and indicate significant higher number of O->N than N->O in CSM considering the distribution from the control datasets.
