# Supplementary material for: Prevalent Accumulation of Non-Optimal Codons through Somatic Mutations in Human Cancers
Source: PLoS One. 2016 Aug 11;11(8):e0160463. doi: 10.1371/journal.pone.0160463 (PMC4981346; doi:10.1371/journal.pone.0160463)
Supplement: S8 Table — The p-values were estimated by Chi-square, two-tail test. (PDF) [file pone.0160463.s010.pdf]

Amino Acids	Datasets	O->N	N->O	Fold	p-values [#1]	p-values [#2]
K	**Ortholog-Poly**	137	107	1.28		
	**SNP-Poly**	26	30	0.87		
	**CSM**	874	250	3.50	*3.23E-12*	*7.51E-08*
E	**Ortholog-Poly**	258	259	1.00		
	**SNP-Poly**	46	43	1.07		
	**CSM**	729	283	2.58	*1.14E-17*	*5.54E-05*
F	**Ortholog-Poly**	73	111	0.66		
	**SNP-Poly**	15	18	0.83		
	**CSM**	597	114	5.24	*5.25E-35*	*1.50E-08*
Q	**Ortholog-Poly**	178	120	1.48		
	**SNP-Poly**	40	21	1.90		
	**CSM**	490	139	3.53	*8.50E-09*	*2.94E-02*
D	**Ortholog-Poly**	243	241	1.01		
	**SNP-Poly**	46	41	1.12		
	**CSM**	275	89	3.09	*6.78E-14*	*2.73E-05*
N	**Ortholog-Poly**	133	114	1.17		
	**SNP-Poly**	37	19	1.95		
	**CSM**	248	85	2.92	*2.29E-07*	*0.188*
S	**Ortholog-Poly**	111	71	1.56		
	**SNP-Poly**	23	10	2.30		
	**CSM**	218	69	3.16	*5.56E-04*	*0.429*
L	**Ortholog-Poly**	106	79	1.34		
	**SNP-Poly**	26	18	1.44		
	**CSM**	213	51	4.18	*7.56E-08*	*1.47E-03*
R	**Ortholog-Poly**	81	88	0.92		
	**SNP-Poly**	21	22	0.95		
	**CSM**	179	55	3.25	*3.33E-09*	*1.98E-04*
H	**Ortholog-Poly**	103	143	0.72		
	**SNP-Poly**	26	20	1.30		
	**CSM**	151	53	2.85	*7.52E-12*	*1.84E-02*
M	**Ortholog-Poly**	382	0	-		
	**SNP-Poly**	111	0	-		
	**CSM**	811	0	-	-	-
W	**Ortholog-Poly**	51	0	-		
	**SNP-Poly**	6	0	-		
	**CSM**	218	0	-	-	-
I	**Ortholog-Poly**	0	377	-		
	**SNP-Poly**	0	85	-		
	**CSM**	0	324	-	-	-
C	**Ortholog-Poly**	0	44	-		
	**SNP-Poly**	0	9	-		

	**CSM**	0	55	-	-	-
	**Ortholog-Poly**	0	0	-		
**Y**	**SNP-Poly**	1	0	-		
	**CSM**	0	0	-	-	-
	**Ortholog-Poly**	0	0	-		
**V**	**SNP-Poly**	0	1	-		
	**CSM**	0	0	-	-	-
	**Ortholog-Poly**	0	0	-		
**T**	**SNP-Poly**	1	1	-		
	**CSM**	0	0	-	-	-
	**Ortholog-Poly**	0	0	-		
**P**	**SNP-Poly**	1	2	-		
	**CSM**	0	0	-	-	-
	**Ortholog-Poly**	0	0	-		
**A**	**SNP-Poly**	0	1	-		
	**CSM**	0	0	-	-	-
	**Ortholog-Poly**	0	0	-		
**G**	**SNP-Poly**	0	0	-		
	**CSM**	0	0	-	-	-

The p-values[#1] were obtained from the comparison in folds of O->N/N->O between the CSM and Ortholog-Poly, the p-values[#2] were obtained from the comparison in folds of O->N/N->O between the CSM and SNP-Poly. The datasets with a total number of O->N and N->O larger than 30 were analyzed, the p-values ≤ 0.05 were represented by red color and indicate significant higher number of O->N than N->O in CSM considering the distribution from the control datasets. The codon transitions of amino acid M, W, I, C were combined for analysis, the codon transitions of Y, V,T,P,A,G were too little to do statistical analysis.
