# Supplementary material for: Prevalent Accumulation of Non-Optimal Codons through Somatic Mutations in Human Cancers
Source: PLoS One. 2016 Aug 11;11(8):e0160463. doi: 10.1371/journal.pone.0160463 (PMC4981346; doi:10.1371/journal.pone.0160463)
Supplement: S9 Table — The p-values were estimated by Chi-square, two-tail test. (PDF) [file pone.0160463.s011.pdf]

Chromosomes	Datasets	O->N	N->O	Fold	p-values#1	p-values #2
1	**Ortholog-Poly**	4, 517	4, 364	1. 04		
	**SNP-Poly**	901	637	1. 41		
	**CSM**	2, 568	742	3. 46	*7.53E-156*	*2.09E-42*
19	**Ortholog-Poly**	3, 745	3, 716	1. 01		
	**SNP-Poly**	667	418	1. 60		
	**CSM**	1, 808	393	4. 60	*2.16E-156*	*3.29E-38*
2	**Ortholog-Poly**	3, 081	3, 235	0. 95		
	**SNP-Poly**	621	498	1. 25		
	**CSM**	1, 781	677	2. 63	*2.65E-89*	*1.32E-23*
11	**Ortholog-Poly**	2, 712	2, 578	1. 05		
	**SNP-Poly**	562	385	1. 46		
	**CSM**	1, 506	405	3. 72	*1.95E-97*	*6.62E-28*
3	**Ortholog-Poly**	2, 402	2, 389	1. 01		
	**SNP-Poly**	531	365	1. 45		
	**CSM**	1, 400	433	3. 23	*3.47E-83*	*2.71E-20*
12	**Ortholog-Poly**	2, 291	2, 268	1. 01		
	**SNP-Poly**	511	326	1. 57		
	**CSM**	1, 352	390	3. 47	*4.03E-86*	*1.46E-18*
17	**Ortholog-Poly**	2, 733	2, 728	1. 00		
	**SNP-Poly**	507	331	1. 53		
	**CSM**	1, 281	311	4. 12	*3.68E-103*	*2.70E-26*
5	**Ortholog-Poly**	1, 877	1, 993	0. 94		
	**SNP-Poly**	430	324	1. 33		
	**CSM**	1, 258	392	3. 21	*6.95E-81*	*1.20E-21*
6	**Ortholog-Poly**	2, 270	2, 367	0. 96		
	**SNP-Poly**	527	378	1. 39		
	**CSM**	1, 246	426	2. 92	*8.72E-73*	*1.60E-17*
7	**Ortholog-Poly**	2, 106	2, 154	0. 98		
	**SNP-Poly**	424	275	1. 54		
	**CSM**	1, 211	387	3. 13	*2.15E-73*	*1.79E-13*
16	**Ortholog-Poly**	2, 204	2, 089	1. 06		
	**SNP-Poly**	396	250	1. 58		
	**CSM**	1, 007	191	5. 27	*8.54E-92*	*8.37E-28*
10	**Ortholog-Poly**	1, 711	1, 795	0. 95		
	**SNP-Poly**	380	272	1. 40		
	**CSM**	974	316	3. 08	*2.71E-61*	*6.17E-15*
9	**Ortholog-Poly**	1, 874	1, 816	1. 03		
	**SNP-Poly**	398	283	1. 41		
	**CSM**	967	231	4. 19	*2.26E-74*	*2.19E-25*
X	**Ortholog-Poly**	975	974	1. 00		
	**SNP-Poly**	190	139	1. 37		

	**CSM**	958	296	3. 24	*3.77E-50*	*1.56E-11*
	**Ortholog-Poly**	1, 793	1, 796	1. 00		
**4**	**SNP-Poly**	342	283	1. 21		
	**CSM**	878	354	2. 48	*1.54E-38*	*1.27E-12*
	**Ortholog-Poly**	1, 408	1, 501	0. 94		
**8**	**SNP-Poly**	305	245	1. 24		
	**CSM**	850	279	3. 05	*8.27E-54*	*1.84E-16*
	**Ortholog-Poly**	1, 215	1, 198	1. 01		
**20**	**SNP-Poly**	264	162	1. 63		
	**CSM**	724	143	5. 06	*4.78E-65*	*1.01E-17*
	**Ortholog-Poly**	1, 588	1, 541	1. 03		
**15**	**SNP-Poly**	293	194	1. 51		
	**CSM**	717	231	3. 10	*9.18E-42*	*1.22E-09*
	**Ortholog-Poly**	1, 396	1, 333	1. 05		
**14**	**SNP-Poly**	290	213	1. 36		
	**CSM**	701	239	2. 93	*6.24E-36*	*4.01E-11*
	**Ortholog-Poly**	1, 001	1, 026	0. 98		
**22**	**SNP-Poly**	235	126	1. 87		
	**CSM**	499	99	5. 04	*1.69E-49*	*8.24E-11*
	**Ortholog-Poly**	801	840	0. 95		
**13**	**SNP-Poly**	154	146	1. 05		
	**CSM**	420	181	2. 32	*7.03E-19*	*4.83E-08*
	**Ortholog-Poly**	815	750	1. 09		
**18**	**SNP-Poly**	154	114	1. 35		
	**CSM**	396	119	3. 33	*3.96E-23*	*1.68E-08*
	**Ortholog-Poly**	642	621	1. 03		
**21**	**SNP-Poly**	104	70	1. 49		
	**CSM**	271	59	4. 59	*1.42E-24*	*4.58E-08*

The p-values[#1] were obtained from the comparison in folds of O->N/N->O between the CSM and Ortholog-Poly, the p-values[#2] were obtained from the comparison in folds of O->N/N->O between the CSM and SNP-Poly. The datasets with a total number of O->N and N->O larger than 30 were analyzed, the p-values $\leq$ 0.05 were represented by red color and indicate significant higher number of O->N than N->O in CSM considering the distribution from the control datasets.
