# Supplementary material for: Prevalent Accumulation of Non-Optimal Codons through Somatic Mutations in Human Cancers
Source: PLoS One. 2016 Aug 11;11(8):e0160463. doi: 10.1371/journal.pone.0160463 (PMC4981346; doi:10.1371/journal.pone.0160463)
Supplement: S10 Table — The p-values were estimated by Chi-square, two-tail test. (PDF) [file pone.0160463.s012.pdf]

Amino Acids	Datasets	O->N	N->O	Fold	p-values[#1]	p-values[#2]
1	**Ortholog-Poly**	169	168	1.01		
	**SNP-Poly**	61	38	1.61		
	**CSM**	506	151	3.35	*8.71E-18*	*9.7E-04*
2	**Ortholog-Poly**	136	154	0.88		
	**SNP-Poly**	29	20	1.45		
	**CSM**	384	135	2.84	*1.24E-14*	*0.0261*
19	**Ortholog-Poly**	157	131	1.20		
	**SNP-Poly**	23	22	1.05		
	**CSM**	318	81	3.93	*1.77E-12*	*1.65E-05*
11	**Ortholog-Poly**	135	115	1.17		
	**SNP-Poly**	22	19	1.16		
	**CSM**	291	85	3.42	*7.85E-10*	*8.52E-04*
6	**Ortholog-Poly**	97	95	1.02		
	**SNP-Poly**	32	20	1.60		
	**CSM**	285	74	3.85	*2.53E-12*	*4.18E-03*
3	**Ortholog-Poly**	94	78	1.21		
	**SNP-Poly**	21	19	1.11		
	**CSM**	279	124	2.25	*7.99E-04*	*0.031*
5	**Ortholog-Poly**	82	94	0.87		
	**SNP-Poly**	21	18	1.17		
	**CSM**	263	99	2.66	*3.36E-09*	*0.0141*
12	**Ortholog-Poly**	92	95	0.97		
	**SNP-Poly**	25	12	2.08		
	**CSM**	254	92	2.76	*2.27E-08*	*0.447537285*
17	**Ortholog-Poly**	106	86	1.23		
	**SNP-Poly**	20	20	1.00		
	**CSM**	244	47	5.19	*5.35E-12*	*5.86E-07*
X	**Ortholog-Poly**	67	61	1.10		
	**SNP-Poly**	12	7	-		
	**CSM**	239	71	3.37	*2.83E-07*	-
4	**Ortholog-Poly**	81	84	0.96		
	**SNP-Poly**	16	15	1.07		
	**CSM**	231	78	2.96	*2.00E-08*	*5.85E-03*
10	**Ortholog-Poly**	77	69	1.12		
	**SNP-Poly**	15	11	-		
	**CSM**	211	62	3.40	*2.40E-07*	-
8	**Ortholog-Poly**	55	54	1.02		
	**SNP-Poly**	11	18	-		
	**CSM**	204	61	3.34	*4.39E-07*	-
7	**Ortholog-Poly**	65	69	0.94		
	**SNP-Poly**	15	14	-		

	**CSM**	202	79	2.56	*3.33E-06*	-
	**Ortholog-Poly**	63	64	0.98		
**9**	**SNP-Poly**	14	21	0.67		
	**CSM**	198	55	3.60	*1.34E-08*	*1.48E-06*
	**Ortholog-Poly**	60	57	1.05		
**15**	**SNP-Poly**	16	10	-		
	**CSM**	157	52	3.02	*1.21E-05*	-
	**Ortholog-Poly**	74	61	1.21		
**14**	**SNP-Poly**	11	9	-		
	**CSM**	153	46	3.33	*2.22E-05*	-
	**Ortholog-Poly**	69	74	0.93		
**16**	**SNP-Poly**	14	14	-		
	**CSM**	150	42	3.57	*1.31E-08*	-
	**Ortholog-Poly**	51	40	1.28		
**20**	**SNP-Poly**	13	7	-		
	**CSM**	111	30	3.70	*2.38E-04*	-
	**Ortholog-Poly**	28	28	1.00		
**13**	**SNP-Poly**	8	5	-		
	**CSM**	99	46	2.15	*1.60E-02*	-
	**Ortholog-Poly**	39	23	1.70		
**18**	**SNP-Poly**	11	6	-		
	**CSM**	97	27	3.59	*2.63E-02*	-
	**Ortholog-Poly**	38	34	1.12		
**22**	**SNP-Poly**	13	10	-		
	**CSM**	74	16	4.63	*5.54E-05*	-
	**Ortholog-Poly**	15	13	1.15		
**21**	**SNP-Poly**	3	6	-		
	**CSM**	53	14	3.79	*1.19E-02*	-

The p-values#1 were obtained from the comparison in folds of O->N/N->O between the CSM and Ortholog-Poly, the p-values#2 were obtained from the comparison in folds of O->N/N->O between the CSM and SNP-Poly. The datasets with a total number of O->N and N->O larger than 30 were analyzed, the p-values $\leq$ 0.05 were represented by red color and indicate significant higher number of O->N than N->O in CSM considering the distribution from the control datasets.
