# Supplementary material for: Prevalent Accumulation of Non-Optimal Codons through Somatic Mutations in Human Cancers
Source: PLoS One. 2016 Aug 11;11(8):e0160463. doi: 10.1371/journal.pone.0160463 (PMC4981346; doi:10.1371/journal.pone.0160463)
Supplement: S11 Table — As the genes with accumulation of non-optimal codons have a significantly higher average proportion of optimal codons than the genes without accumulation of non-optimal codons, a subset of genes with accumulation of non-optimal codons were the sampled to have a similar average proportion of optimal codons with the genes without accumulation of non-optimal codons. (PDF) [file pone.0160463.s013.pdf]

Genes with accumulation of optimal codons	Genes with accumulation of nonoptimal codons	The sampled genes with accumulation of nonoptimal codons
ENSG00000038427	ENSG00000127720	ENSG00000127720
ENSG00000116062	ENSG00000165240	ENSG00000165240
ENSG00000068878	ENSG00000180739	ENSG00000172020
ENSG00000005483	ENSG00000172020	ENSG00000131724
ENSG00000196455	ENSG00000131724	ENSG00000128595
ENSG00000123607	ENSG00000128595	ENSG00000100211
ENSG00000004766	ENSG00000100211	ENSG00000186479
ENSG00000164037	ENSG00000186479	ENSG00000156282
ENSG00000124279	ENSG00000156282	ENSG00000100652
ENSG00000131747	ENSG00000100652	ENSG00000258472
ENSG00000168813	ENSG00000258472	ENSG00000167536
ENSG00000135968	ENSG00000167536	ENSG00000090539
ENSG00000004897	ENSG00000090539	ENSG00000102805
ENSG00000108510	ENSG00000102805	ENSG00000119314
ENSG00000172139	ENSG00000119314	ENSG00000159259
ENSG00000128915	ENSG00000159259	ENSG00000179776
ENSG00000135315	ENSG00000179776	ENSG00000125266
ENSG00000188107	ENSG00000125266	ENSG00000196220
ENSG00000136014	ENSG00000165985	ENSG00000155792
ENSG00000166004	ENSG00000196220	ENSG00000133665
ENSG00000189056	ENSG00000155792	ENSG00000181852
ENSG00000115760	ENSG00000160285	ENSG00000213341
ENSG00000145087	ENSG00000133665	ENSG00000047315
ENSG00000079156	ENSG00000181852	ENSG00000126785
ENSG00000115970	ENSG00000076344	ENSG00000115159
ENSG00000133703	ENSG00000142279	ENSG00000162636
ENSG00000138398	ENSG00000213341	ENSG00000249581
ENSG00000135249	ENSG00000047315	ENSG00000100196
ENSG00000123200	ENSG00000126785	ENSG00000110987
ENSG00000164418	ENSG00000115159	ENSG00000132612
ENSG00000146587	ENSG00000162636	ENSG00000163481
ENSG00000005810	ENSG00000130700	ENSG00000132185
ENSG00000176222	ENSG00000249581	ENSG00000145022
ENSG00000117523	ENSG00000100196	ENSG00000179841
ENSG00000164190	ENSG00000110987	ENSG00000171840
ENSG00000153827	ENSG00000130270	ENSG00000074356
ENSG00000071794	ENSG00000132612	ENSG00000186051
ENSG00000188641	ENSG00000163481	ENSG00000256683
ENSG00000204262	ENSG00000132185	ENSG00000081985
ENSG00000057468	ENSG00000178585	ENSG00000137842
ENSG00000138035	ENSG00000162931	ENSG00000188811
ENSG00000181450	ENSG00000145022	ENSG00000197213
ENSG00000102595	ENSG00000196169	ENSG00000119688
ENSG00000075945	ENSG00000179841	ENSG00000163823
ENSG00000138778	ENSG00000204160	ENSG00000108381
ENSG00000144451	ENSG00000171840	ENSG00000064042
ENSG00000033867	ENSG00000010319	ENSG00000187187
ENSG00000248905	ENSG00000074356	ENSG00000141371
ENSG00000166783	ENSG00000186051	ENSG00000197756
ENSG00000107651	ENSG00000256683	ENSG00000104442
ENSG00000127995	ENSG00000081985	ENSG00000204913
ENSG00000162437	ENSG00000137842	ENSG00000139908
ENSG00000136546	ENSG00000188811	ENSG00000258518
ENSG00000182890	ENSG00000197213	ENSG00000152904
ENSG00000144619	ENSG00000119688	ENSG00000129595
ENSG00000091009	ENSG00000163823	ENSG00000112319
ENSG00000184881	ENSG00000170989	ENSG00000197181
ENSG00000123106	ENSG00000108381	ENSG00000134897
ENSG00000005022	ENSG00000173302	ENSG00000235863
ENSG00000178075	ENSG00000064042	ENSG00000152763

ENSG00000145388	ENSG00000198113	ENSG00000131381
ENSG00000151779	ENSG00000187187	ENSG00000153237
ENSG00000186113	ENSG00000141371	ENSG00000137054
ENSG00000143520	ENSG00000197756	ENSG00000004864
ENSG00000172888	ENSG00000104442	ENSG00000176402
ENSG00000148516	ENSG00000204913	ENSG00000181396
ENSG00000139354	ENSG00000139908	ENSG00000151846
ENSG00000083097	ENSG00000258518	ENSG00000206075
ENSG00000144445	ENSG00000152904	ENSG00000051825
ENSG00000155744	ENSG00000129595	ENSG00000183783
ENSG00000198836	ENSG00000112319	ENSG00000048740
ENSG00000042317	ENSG00000197181	ENSG00000186297
ENSG00000117262	ENSG00000134897	ENSG00000080493
ENSG00000111700	ENSG00000235863	ENSG00000131378
ENSG00000134744	ENSG00000152763	ENSG00000163521
ENSG00000121316	ENSG00000131381	ENSG00000091831
ENSG00000120526	ENSG00000153237	ENSG00000116151
ENSG00000065060	ENSG00000137054	ENSG00000158352
ENSG00000196502	ENSG00000004864	ENSG00000162191
ENSG00000162779	ENSG00000176402	ENSG00000102900
ENSG00000186714	ENSG00000181396	ENSG00000213934
ENSG00000196110	ENSG00000151846	ENSG00000179195
ENSG00000125885	ENSG00000206075	ENSG00000124588
ENSG00000166928	ENSG00000051825	ENSG00000076258
ENSG00000169508	ENSG00000144031	ENSG00000188316
ENSG00000082898	ENSG00000183783	ENSG00000242247
ENSG00000070367	ENSG00000048740	ENSG00000112312
ENSG00000069122	ENSG00000137857	ENSG00000144824
ENSG00000180777	ENSG00000186297	ENSG00000126107
ENSG00000121989	ENSG00000080493	ENSG00000172575
ENSG00000170017	ENSG00000131378	ENSG00000168746
ENSG00000114302	ENSG00000131187	ENSG00000180611
ENSG00000171970	ENSG00000116525	ENSG00000129472
ENSG00000145332	ENSG00000163521	ENSG00000123472
ENSG00000170471	ENSG00000198049	ENSG00000161526
ENSG00000131023	ENSG00000091831	ENSG00000173699
ENSG00000120802	ENSG00000116151	ENSG00000132872
ENSG00000167978	ENSG00000158352	ENSG00000078618
ENSG00000134982	ENSG00000197079	ENSG00000122584
ENSG00000100592	ENSG00000162191	ENSG00000183960
ENSG00000149218	ENSG00000102900	ENSG00000089060
ENSG00000006712	ENSG00000213934	ENSG00000148136
ENSG00000177683	ENSG00000179195	ENSG00000198715
ENSG00000180257	ENSG00000172531	ENSG00000154451
ENSG00000152270	ENSG00000124588	ENSG00000104823
ENSG00000104375	ENSG00000076258	ENSG00000125744
ENSG00000256771	ENSG00000188316	ENSG00000165792
ENSG00000129315	ENSG00000242247	ENSG00000204311
ENSG00000164099	ENSG00000112312	ENSG00000010610
ENSG00000164675	ENSG00000144824	ENSG00000072401
ENSG00000188153	ENSG00000126107	ENSG00000108523
ENSG00000136542	ENSG00000172575	ENSG00000129317
ENSG00000166263	ENSG00000168746	ENSG00000105679
ENSG00000174780	ENSG00000180611	ENSG00000132383
ENSG00000115504	ENSG00000129472	ENSG00000248713
ENSG00000102144	ENSG00000140675	ENSG00000118271
ENSG00000102189	ENSG00000123472	ENSG00000166896
ENSG00000085840	ENSG00000161526	ENSG00000153064
ENSG00000110318	ENSG00000173699	ENSG00000078328
ENSG00000113810	ENSG00000187531	ENSG00000111845
ENSG00000026103	ENSG00000132872	ENSG00000101464
ENSG00000165392	ENSG00000204612	ENSG00000198657
ENSG00000257046	ENSG00000078618	ENSG00000171067
ENSG00000127914	ENSG00000122584	ENSG00000196578

ENSG00000104093	ENSG00000183960	ENSG00000066455
ENSG00000109689	ENSG00000125531	ENSG00000171055
ENSG00000159086	ENSG00000089060	ENSG00000163221
ENSG00000080345	ENSG00000148136	ENSG00000242180
ENSG00000170759	ENSG00000198715	ENSG00000118217
ENSG00000160352	ENSG00000154451	ENSG00000184056
ENSG00000186088	ENSG00000164438	ENSG00000138757
ENSG00000122484	ENSG00000163497	ENSG00000147679
ENSG00000162624	ENSG00000104823	ENSG00000182545
ENSG00000267360	ENSG00000125744	ENSG00000102054
ENSG00000066084	ENSG00000165792	ENSG00000112096
ENSG00000205413	ENSG00000204311	ENSG00000141642
ENSG00000198185	ENSG00000007541	ENSG00000172724
ENSG00000161057	ENSG00000010610	ENSG00000179057
ENSG00000180376	ENSG00000072401	ENSG00000182318
ENSG00000175066	ENSG00000108523	ENSG00000147488
ENSG00000154710	ENSG00000129317	ENSG00000187601
ENSG00000104320	ENSG00000105679	ENSG00000185100
ENSG00000110841	ENSG00000132383	ENSG00000135763
ENSG00000240403	ENSG00000248713	ENSG00000112029
ENSG00000047188	ENSG00000186844	ENSG00000213619
ENSG00000107165	ENSG00000118271	ENSG00000158220
ENSG00000186310	ENSG00000166896	ENSG00000179097
ENSG00000150753	ENSG00000153064	ENSG00000004455
ENSG00000185792	ENSG00000078328	ENSG00000213420
ENSG00000155085	ENSG00000111845	ENSG00000151466
ENSG00000134121	ENSG00000101464	ENSG00000170091
ENSG00000129071	ENSG00000198657	ENSG00000156983
ENSG00000119912	ENSG00000171067	ENSG00000228083
ENSG00000183814	ENSG00000196578	ENSG00000181562
ENSG00000087253	ENSG00000066455	ENSG00000158079
ENSG00000167377	ENSG00000197530	ENSG00000184012
ENSG00000198843	ENSG00000171055	ENSG00000053372
ENSG00000109101	ENSG00000163221	ENSG00000077063
ENSG00000111371	ENSG00000242180	ENSG00000183813
ENSG00000014123	ENSG00000118217	ENSG00000158578
ENSG00000105856	ENSG00000184056	ENSG00000155890
ENSG00000101310	ENSG00000138757	ENSG00000259224
ENSG00000101955	ENSG00000011304	ENSG00000005238
ENSG00000147592	ENSG00000147679	ENSG00000100122
ENSG00000117010	ENSG00000182545	ENSG00000164603
ENSG00000101161	ENSG00000102054	ENSG00000175544
ENSG00000184575	ENSG00000112096	ENSG00000170540
ENSG00000139324	ENSG00000141642	ENSG00000177688
ENSG00000198961	ENSG00000188051	ENSG00000197142
ENSG00000189045	ENSG00000172724	ENSG00000189221
ENSG00000221944	ENSG00000179057	ENSG00000129988
ENSG00000132466	ENSG00000182318	ENSG00000160785
ENSG00000171033	ENSG00000147488	ENSG00000198728
ENSG00000109911	ENSG00000187601	ENSG00000196236
ENSG00000196083	ENSG00000185100	ENSG00000168958
ENSG00000146872	ENSG00000135763	ENSG00000167779
ENSG00000119787	ENSG00000161395	ENSG00000175274
ENSG00000172123	ENSG00000112029	ENSG00000006634
ENSG00000138653	ENSG00000213619	ENSG00000143450
ENSG00000102032	ENSG00000158220	ENSG00000165071
ENSG00000136770	ENSG00000179097	ENSG00000141655
ENSG00000006747	ENSG00000004455	ENSG00000198805
ENSG00000107854	ENSG00000213420	ENSG00000108055
ENSG00000071205	ENSG00000151466	ENSG00000234068
ENSG00000092201	ENSG00000170091	ENSG00000174792
ENSG00000171557	ENSG00000156983	ENSG00000115641
ENSG00000140598	ENSG00000228083	ENSG00000056097
ENSG00000204644	ENSG00000181562	ENSG00000106853

ENSG00000112851	ENSG00000158079	ENSG00000113273
ENSG00000182053	ENSG00000184012	ENSG00000147168
ENSG00000104427	ENSG00000053372	ENSG00000186231
ENSG00000073712	ENSG00000077063	ENSG00000111276
ENSG00000163939	ENSG00000183813	ENSG00000182793
ENSG00000066777	ENSG00000145990	ENSG00000183850
ENSG00000198160	ENSG00000131368	ENSG00000109220
ENSG00000214827	ENSG00000158578	ENSG00000117174
ENSG00000112297	ENSG00000155890	ENSG00000165102
ENSG00000186814	ENSG00000259224	ENSG00000093009
ENSG00000168137	ENSG00000182162	ENSG00000147234
ENSG00000118873	ENSG00000005238	ENSG00000085831
ENSG00000204296	ENSG00000100122	ENSG00000153531
ENSG00000030066	ENSG00000164603	ENSG00000119673
ENSG00000188171	ENSG00000175544	ENSG00000087077
ENSG00000072041	ENSG00000171791	ENSG00000183571
ENSG00000127125	ENSG00000170540	ENSG00000083844
ENSG00000248333	ENSG00000037280	ENSG00000135436
ENSG00000137845	ENSG00000177688	ENSG00000092850
ENSG00000211456	ENSG00000173918	ENSG00000136052
ENSG00000109686	ENSG00000197142	ENSG00000120087
ENSG00000257103	ENSG00000072163	ENSG00000139719
ENSG00000239474	ENSG00000189221	ENSG00000121318
ENSG00000109320	ENSG00000129988	ENSG00000154065
ENSG00000083093	ENSG00000160785	ENSG00000185842
ENSG00000109189	ENSG00000198728	ENSG00000159593
ENSG00000115758	ENSG00000196236	ENSG00000079482
ENSG00000135250	ENSG00000168958	ENSG00000117682
ENSG00000169018	ENSG00000167779	ENSG00000119950
ENSG00000083535	ENSG00000175274	ENSG00000170180
ENSG00000168619	ENSG00000006634	ENSG00000188373
ENSG00000107443	ENSG00000143450	ENSG00000094804
ENSG00000172766	ENSG00000165071	ENSG00000174516
ENSG00000213759	ENSG00000141655	ENSG00000121552
ENSG00000145390	ENSG00000198805	ENSG00000197448
ENSG00000048405	ENSG00000108055	ENSG00000150244
ENSG00000116095	ENSG00000101444	ENSG00000174640
ENSG00000109861	ENSG00000234068	ENSG00000168026
ENSG00000126016	ENSG00000174792	ENSG00000136732
ENSG00000170456	ENSG00000115641	ENSG00000226979
ENSG00000115825	ENSG00000056097	ENSG00000113648
ENSG00000140367	ENSG00000106853	ENSG00000261359
ENSG00000172748	ENSG00000113273	ENSG00000152402
ENSG00000148935	ENSG00000147168	ENSG00000125726
ENSG00000138738	ENSG00000186231	ENSG00000186818
ENSG00000177613	ENSG00000111276	ENSG00000172640
ENSG00000197714	ENSG00000092067	ENSG00000198924
ENSG00000171848	ENSG00000182793	ENSG00000117507
ENSG00000168917	ENSG00000183850	ENSG00000178404
ENSG00000184984	ENSG00000109220	ENSG00000154265
ENSG00000182010	ENSG00000117174	ENSG00000152578
ENSG00000097033	ENSG00000165102	ENSG00000174227
ENSG00000165775	ENSG00000093009	ENSG00000110079
ENSG00000196376	ENSG00000147234	ENSG00000151065
ENSG00000151923	ENSG00000085831	ENSG00000130177
ENSG00000119535	ENSG00000153531	ENSG00000175746
ENSG00000162924	ENSG00000119673	ENSG00000126254
ENSG00000262209	ENSG00000087077	ENSG00000156313
ENSG00000113595	ENSG00000183571	ENSG00000143157
ENSG00000171160	ENSG00000187730	ENSG00000108684
ENSG00000151773	ENSG00000083844	ENSG00000159214
ENSG00000085224	ENSG00000125841	ENSG00000114904
ENSG00000165813	ENSG00000135436	ENSG00000137462
ENSG00000135205	ENSG00000092850	ENSG00000126231

ENSG00000180872	ENSG00000136052	ENSG00000004779
ENSG00000112972	ENSG00000120087	ENSG00000170962
ENSG00000105750	ENSG00000139719	ENSG00000121022
ENSG00000182070	ENSG00000121318	ENSG00000164707
ENSG00000113649	ENSG00000154065	ENSG00000167721
ENSG00000011114	ENSG00000185842	ENSG00000204498
ENSG00000156076	ENSG00000145911	ENSG00000156858
ENSG00000159917	ENSG00000159593	ENSG00000175556
ENSG00000047346	ENSG00000183682	ENSG00000259220
ENSG00000005189	ENSG00000079482	ENSG00000106809
ENSG00000137822	ENSG00000162975	ENSG00000136518
ENSG00000160588	ENSG00000117682	ENSG00000182986
ENSG00000142621	ENSG00000119950	ENSG00000122705
ENSG00000137145	ENSG00000170180	ENSG00000112655
ENSG00000176105	ENSG00000112619	ENSG00000249437
ENSG00000101890	ENSG00000188373	ENSG00000213085
ENSG00000168542	ENSG00000094804	ENSG00000128656
ENSG00000008517	ENSG00000188505	ENSG00000137601
ENSG00000251569	ENSG00000174516	ENSG00000101150
ENSG00000226761	ENSG00000121552	ENSG00000173614
ENSG00000170802	ENSG00000197448	ENSG00000140287
ENSG00000240303	ENSG00000095370	ENSG00000154330
ENSG00000163285	ENSG00000150244	ENSG00000170776
ENSG00000085382	ENSG00000174640	ENSG00000165609
ENSG00000139679	ENSG00000168026	ENSG00000113532
ENSG00000160688	ENSG00000152784	ENSG00000167065
ENSG00000172939	ENSG00000136732	ENSG00000117868
ENSG00000198721	ENSG00000226979	ENSG00000122299
ENSG00000138399	ENSG00000113648	ENSG00000181074
ENSG00000188175	ENSG00000261359	ENSG00000172188
ENSG00000173875	ENSG00000152402	ENSG00000143951
ENSG00000169684	ENSG00000181234	ENSG00000139684
ENSG00000139155	ENSG00000125726	ENSG00000139579
ENSG00000068885	ENSG00000186818	ENSG00000117479
ENSG00000141480	ENSG00000158792	ENSG00000128254
ENSG00000106144	ENSG00000172640	ENSG00000140400
ENSG00000155313	ENSG00000181830	ENSG00000065970
ENSG00000213020	ENSG00000198924	ENSG00000185414
ENSG00000163093	ENSG00000117507	ENSG00000187033
ENSG00000158161	ENSG00000178404	ENSG00000168000
ENSG00000018869	ENSG00000154265	ENSG00000079337
ENSG00000165028	ENSG00000136856	ENSG00000169402
ENSG00000173376	ENSG00000152578	ENSG00000125753
ENSG00000215853	ENSG00000174227	ENSG00000167588
ENSG00000174839	ENSG00000176894	ENSG00000114279
ENSG00000177201	ENSG00000172935	ENSG00000157851
ENSG00000105373	ENSG00000110079	ENSG00000153944
ENSG00000204713	ENSG00000151065	ENSG00000134871
ENSG00000205189	ENSG00000130177	ENSG00000132305
ENSG00000198440	ENSG00000175746	ENSG00000241468
ENSG00000174840	ENSG00000126254	ENSG00000244122
ENSG00000061676	ENSG00000156313	ENSG00000185385
ENSG00000188393	ENSG00000143157	ENSG00000015285
ENSG00000090534	ENSG00000108684	ENSG00000118596
ENSG00000120616	ENSG00000159214	ENSG00000115902
ENSG00000221874	ENSG00000114904	ENSG00000119471
ENSG00000197323	ENSG00000137462	ENSG00000186075
ENSG00000163535	ENSG00000126231	ENSG00000174206
ENSG00000113249	ENSG00000004779	ENSG00000204386
ENSG00000009694	ENSG00000170962	ENSG00000022355
ENSG00000137642	ENSG00000121022	ENSG00000119383
ENSG00000198477	ENSG00000120875	ENSG00000244405
ENSG00000134815	ENSG00000164707	ENSG00000163444
ENSG00000176896	ENSG00000167721	ENSG00000215251

ENSG00000167447	ENSG00000204498	ENSG00000106436
ENSG00000135218	ENSG00000156858	ENSG00000197880
ENSG00000123737	ENSG00000175556	ENSG00000213760
ENSG00000214193	ENSG00000126803	ENSG00000114503
ENSG00000153015	ENSG00000259220	ENSG00000126773
ENSG00000094975	ENSG00000106809	ENSG00000121749
ENSG00000158315	ENSG00000136518	ENSG00000250127
ENSG00000166167	ENSG00000182986	ENSG00000133142
ENSG00000115904	ENSG00000180264	ENSG00000171180
ENSG00000234511	ENSG00000122705	ENSG00000214706
ENSG00000155189	ENSG00000112655	ENSG00000166682
ENSG00000173480	ENSG00000249437	ENSG00000139055
ENSG00000080823	ENSG00000213085	ENSG00000125149
ENSG00000251192	ENSG00000174903	ENSG00000167461
ENSG00000138668	ENSG00000128656	ENSG00000140320
ENSG00000172421	ENSG00000137601	ENSG00000133063
ENSG00000180917	ENSG00000101150	ENSG00000181885
ENSG00000150681	ENSG00000173614	ENSG00000212900
ENSG00000145495	ENSG00000109956	ENSG00000154920
ENSG00000127993	ENSG00000140287	ENSG00000167311
ENSG00000104205	ENSG00000154330	ENSG00000110367
ENSG00000255501	ENSG00000185000	ENSG00000204138
ENSG00000165929	ENSG00000170776	ENSG00000104549
ENSG00000166960	ENSG00000165609	ENSG00000152556
ENSG00000165694	ENSG00000141873	ENSG00000196071
ENSG00000079557	ENSG00000113532	ENSG00000172667
ENSG00000196782	ENSG00000167065	ENSG00000172459
ENSG00000142731	ENSG00000117868	ENSG00000166398
ENSG00000143595	ENSG00000122299	ENSG00000149346
ENSG00000196240	ENSG00000181074	ENSG00000097096
ENSG00000212643	ENSG00000172188	ENSG00000240021
ENSG00000143374	ENSG00000143951	ENSG00000179542
ENSG00000198250	ENSG00000139684	ENSG00000204569
ENSG00000155850	ENSG00000139579	ENSG00000131944
ENSG00000122779	ENSG00000117479	ENSG00000103168
ENSG00000183735	ENSG00000128254	ENSG00000099399
ENSG00000116198	ENSG00000140400	ENSG00000146067
ENSG00000170954	ENSG00000065970	ENSG00000196396
ENSG00000139505	ENSG00000185414	ENSG00000198265
ENSG00000069956	ENSG00000187033	ENSG00000171763
ENSG00000186871	ENSG00000168000	ENSG00000188984
ENSG00000127083	ENSG00000079337	ENSG00000171847
ENSG00000239779	ENSG00000169402	ENSG00000057935
ENSG00000185947	ENSG00000125753	ENSG00000150627
ENSG00000197013	ENSG00000167588	ENSG00000244414
ENSG00000165156	ENSG00000114279	ENSG00000108474
ENSG00000164754	ENSG00000157851	ENSG00000050344
ENSG00000047230	ENSG00000153944	ENSG00000117593
ENSG00000163946	ENSG00000130304	ENSG00000254986
ENSG00000106384	ENSG00000134871	ENSG00000023228
ENSG00000125686	ENSG00000132305	ENSG00000175489
ENSG00000119969	ENSG00000241468	ENSG00000105928
ENSG00000256436	ENSG00000244122	ENSG00000136653
ENSG00000135299	ENSG00000185385	ENSG00000213689
ENSG00000137478	ENSG00000174586	ENSG00000088727
ENSG00000171121	ENSG00000148926	ENSG00000189023
ENSG00000170801	ENSG00000015285	ENSG00000179335
ENSG00000156671	ENSG00000118596	ENSG00000136754
ENSG00000100100	ENSG00000115902	ENSG00000181904
ENSG00000146063	ENSG00000135604	ENSG00000128346
ENSG00000164129	ENSG00000119471	ENSG00000172809
ENSG00000137571	ENSG00000186075	ENSG00000111581
ENSG00000163491	ENSG00000175766	ENSG00000197768
ENSG00000164070	ENSG00000174206	ENSG00000057019

ENSG00000092148	ENSG00000204386	ENSG00000184022
ENSG00000236699	ENSG00000022355	ENSG00000090060
ENSG00000083520	ENSG00000119383	ENSG00000130707
ENSG00000180336	ENSG00000119280	ENSG00000183044
ENSG00000163749	ENSG00000177291	ENSG00000268194
ENSG00000134762	ENSG00000244405	ENSG00000120727
ENSG00000089876	ENSG00000163444	ENSG00000065371
ENSG00000008118	ENSG00000215251	ENSG00000174370
ENSG00000116984	ENSG00000115461	ENSG00000151498
ENSG00000135077	ENSG00000106436	ENSG00000197134
ENSG00000156162	ENSG00000197880	ENSG00000109016
ENSG00000163728	ENSG00000264813	ENSG00000141627
ENSG00000182903	ENSG00000213760	ENSG00000186407
ENSG00000001631	ENSG00000050820	ENSG00000204520
ENSG00000184451	ENSG00000114503	ENSG00000169213
ENSG00000134255	ENSG00000128709	ENSG00000151092
ENSG00000011009	ENSG00000126773	ENSG00000171408
ENSG00000139910	ENSG00000120738	ENSG00000124939
ENSG00000165916	ENSG00000121749	ENSG00000140543
ENSG00000109163	ENSG00000250127	ENSG00000222028
ENSG00000100888	ENSG00000133142	ENSG00000099194
ENSG00000177565	ENSG00000171180	ENSG00000085415
ENSG00000130826	ENSG00000214706	ENSG00000255804
ENSG00000101126	ENSG00000166682	ENSG00000140577
ENSG00000143032	ENSG00000139055	ENSG00000065491
ENSG00000077380	ENSG00000128294	ENSG00000038002
ENSG00000148702	ENSG00000197241	ENSG00000152413
ENSG00000147162	ENSG00000125149	ENSG00000000457
ENSG00000084754	ENSG00000167461	ENSG00000186523
ENSG00000114648	ENSG00000140320	ENSG00000092964
ENSG00000196504	ENSG00000180155	ENSG00000165283
ENSG00000103429	ENSG00000168763	ENSG00000170476
ENSG00000112053	ENSG00000133063	ENSG00000132792
ENSG00000198142	ENSG00000181885	ENSG00000175318
ENSG00000119125	ENSG00000212900	ENSG00000117395
ENSG00000198515	ENSG00000154920	ENSG00000189013
ENSG00000121350	ENSG00000167311	ENSG00000101003
ENSG00000100852	ENSG00000110367	ENSG00000186335
ENSG00000139318	ENSG00000204138	ENSG00000164188
ENSG00000101639	ENSG00000104549	ENSG00000146267
ENSG00000165084	ENSG00000152556	ENSG00000118434
ENSG00000137337	ENSG00000104901	ENSG00000183475
ENSG00000162775	ENSG00000196071	ENSG00000030110
ENSG00000143355	ENSG00000172667	ENSG00000152234
ENSG00000089048	ENSG00000172459	ENSG00000139278
ENSG00000023516	ENSG00000166398	ENSG00000197620
ENSG00000217128	ENSG00000091073	ENSG00000090857
ENSG00000198554	ENSG00000149346	ENSG00000104388
ENSG00000113407	ENSG00000097096	ENSG00000106477
ENSG00000111206	ENSG00000240021	ENSG00000155530
ENSG00000138777	ENSG00000179542	ENSG00000164053
ENSG00000119820	ENSG00000204569	ENSG00000138750
ENSG00000144320	ENSG00000131944	ENSG00000177462
ENSG00000171262	ENSG00000177202	ENSG00000179695
ENSG00000154415	ENSG00000103168	ENSG00000100109
ENSG00000164944	ENSG00000099399	ENSG00000157456
ENSG00000143952	ENSG00000146067	ENSG00000219492
ENSG00000218823	ENSG00000196396	ENSG00000062650
ENSG00000058272	ENSG00000198265	ENSG00000167670
ENSG00000162402	ENSG00000171763	ENSG00000107551
ENSG00000177189	ENSG00000188984	ENSG00000103415
ENSG00000172456	ENSG00000171847	ENSG00000090924
ENSG00000196867	ENSG00000057935	ENSG00000138081
ENSG00000137558	ENSG00000150627	ENSG00000196911

ENSG00000155970	ENSG00000244414	ENSG00000196616
ENSG00000173432	ENSG00000108474	ENSG00000117069
ENSG00000145107	ENSG00000050344	ENSG00000120729
ENSG00000148225	ENSG00000117593	ENSG00000203907
ENSG00000182973	ENSG00000254986	ENSG00000179476
ENSG00000198677	ENSG00000023228	ENSG00000108375
ENSG00000221961	ENSG00000175489	ENSG00000138346
ENSG00000076053	ENSG00000105928	ENSG00000113070
ENSG00000197275	ENSG00000136653	ENSG00000110074
ENSG00000102309	ENSG00000213689	ENSG00000187630
ENSG00000132170	ENSG00000088727	ENSG00000162631
ENSG00000164506	ENSG00000189023	ENSG00000198483
ENSG00000175575	ENSG00000179335	ENSG00000197629
ENSG00000241058	ENSG00000136754	ENSG00000254535
ENSG00000221843	ENSG00000181904	ENSG00000165555
ENSG00000128918	ENSG00000101246	ENSG00000170545
ENSG00000229676	ENSG00000128346	ENSG00000067182
ENSG00000159784	ENSG00000172809	ENSG00000170364
ENSG00000175054	ENSG00000111581	ENSG00000180999
ENSG00000255423	ENSG00000197768	ENSG00000103353
ENSG00000196233	ENSG00000057019	ENSG00000186265
ENSG00000196776	ENSG00000197723	ENSG00000185104
ENSG00000204514	ENSG00000127054	ENSG00000147613
ENSG00000100941	ENSG00000184022	ENSG00000188133
ENSG00000187950	ENSG00000090060	ENSG00000153006
ENSG00000171806	ENSG00000130707	ENSG00000107447
ENSG00000223443	ENSG00000183044	ENSG00000168300
ENSG00000158615	ENSG00000268194	ENSG00000182712
ENSG00000076067	ENSG00000145945	ENSG00000182180
ENSG00000130224	ENSG00000103264	ENSG00000135045
ENSG00000070444	ENSG00000120727	ENSG00000114062
ENSG00000108239	ENSG00000065371	ENSG00000021461
ENSG00000020922	ENSG00000174370	ENSG00000106367
ENSG00000108021	ENSG00000151498	ENSG00000198270
ENSG00000185630	ENSG00000197134	ENSG00000181896
ENSG00000176624	ENSG00000109016	ENSG00000168556
ENSG00000147274	ENSG00000141627	ENSG00000188931
ENSG00000132294	ENSG00000186407	ENSG00000144589
ENSG00000168288	ENSG00000204520	ENSG00000111405
ENSG00000100227	ENSG00000169213	ENSG00000196700
ENSG00000166368	ENSG00000151092	ENSG00000225683
ENSG00000172244	ENSG00000171408	ENSG00000152433
ENSG00000029639	ENSG00000124939	ENSG00000139617
ENSG00000130349	ENSG00000197561	ENSG00000188677
ENSG00000182333	ENSG00000140543	ENSG00000101049
ENSG00000088451	ENSG00000222028	ENSG00000170581
ENSG00000154822	ENSG00000099194	ENSG00000183549
ENSG00000170632	ENSG00000085415	ENSG00000163607
ENSG00000143457	ENSG00000188976	ENSG00000009307
ENSG00000125633	ENSG00000255804	ENSG00000052841
ENSG00000187550	ENSG00000140577	ENSG00000110811
ENSG00000186063	ENSG00000065491	ENSG00000027644
ENSG00000144026	ENSG00000038002	ENSG00000042445
ENSG00000148229	ENSG00000152413	ENSG00000149305
ENSG00000162739	ENSG00000000457	ENSG00000259075
ENSG00000124406	ENSG00000186523	ENSG00000103126
ENSG00000175305	ENSG00000129347	ENSG00000100142
ENSG00000147138	ENSG00000092964	ENSG00000127564
ENSG00000106278	ENSG00000165283	ENSG00000006459
ENSG00000113391	ENSG00000170476	ENSG00000148719
ENSG00000164035	ENSG00000132792	ENSG00000130023
ENSG00000173890	ENSG00000167770	ENSG00000125676
ENSG00000136100	ENSG00000175318	ENSG00000125999
ENSG00000163104	ENSG00000117395	ENSG00000055955

ENSG00000048392	ENSG00000175093	ENSG00000205464
ENSG00000006128	ENSG00000189013	ENSG00000143622
ENSG00000176986	ENSG00000101003	ENSG00000116521
ENSG00000197329	ENSG00000186335	ENSG00000112761
ENSG00000086288	ENSG00000164188	ENSG00000187116
ENSG00000135845	ENSG00000146267	ENSG00000154493
ENSG00000165209	ENSG00000175077	ENSG00000072080
ENSG00000156860	ENSG00000118434	ENSG00000125740
ENSG00000196611	ENSG00000183475	ENSG00000021488
ENSG00000081026	ENSG00000030110	ENSG00000132911
ENSG00000187546	ENSG00000152234	ENSG00000161277
ENSG00000160888	ENSG00000139278	ENSG00000196839
ENSG00000136522	ENSG00000197620	ENSG00000104755
ENSG00000129003	ENSG00000167861	ENSG00000112130
ENSG00000197822	ENSG00000090857	ENSG00000081181
ENSG00000214248	ENSG00000104388	ENSG00000185697
ENSG00000189252	ENSG00000145217	ENSG00000168530
ENSG00000135951	ENSG00000106477	ENSG00000071539
ENSG00000142867	ENSG00000196787	ENSG00000174145
ENSG00000156471	ENSG00000155530	ENSG00000101474
ENSG00000135698	ENSG00000164053	ENSG00000197302
ENSG00000135318	ENSG00000138750	ENSG00000011523
ENSG00000181036	ENSG00000177462	ENSG00000101752
ENSG00000147140	ENSG00000179695	ENSG00000019485
ENSG00000138050	ENSG00000213445	ENSG00000100226
ENSG00000172967	ENSG00000100109	ENSG00000185156
ENSG00000105136	ENSG00000157456	ENSG00000137343
ENSG00000170209	ENSG00000219492	ENSG00000161905
ENSG00000214046	ENSG00000062650	ENSG00000221972
ENSG00000214978	ENSG00000167670	ENSG00000258890
ENSG00000167393	ENSG00000107551	ENSG00000159685
ENSG00000107147	ENSG00000103415	ENSG00000151491
ENSG00000134107	ENSG00000090924	ENSG00000171236
ENSG00000184983	ENSG00000138081	ENSG00000186795
ENSG00000239306	ENSG00000196911	ENSG00000197938
ENSG00000138286	ENSG00000196616	ENSG00000053501
ENSG00000173153	ENSG00000117069	ENSG00000138246
ENSG00000176692	ENSG00000120729	ENSG00000197548
ENSG00000125482	ENSG00000203907	ENSG00000204513
ENSG00000118017	ENSG00000179476	ENSG00000128606
ENSG00000198478	ENSG00000108375	ENSG00000117448
ENSG00000179454	ENSG00000138346	ENSG00000106483
ENSG00000175325	ENSG00000113070	ENSG00000119862
ENSG00000253309	ENSG00000228594	ENSG00000214290
ENSG00000170264	ENSG00000133247	ENSG00000183172
ENSG00000197557	ENSG00000186766	ENSG00000072422
ENSG00000198818	ENSG00000102871	ENSG00000100916
ENSG00000182993	ENSG00000110074	ENSG00000176988
ENSG00000240344	ENSG00000136213	ENSG00000176209
ENSG00000213927	ENSG00000187630	ENSG00000136367
ENSG00000117500	ENSG00000243284	ENSG00000116604
ENSG00000155906	ENSG00000115085	ENSG00000258817
ENSG00000138175	ENSG00000162631	ENSG00000162869
ENSG00000105173	ENSG00000154035	ENSG00000115165
ENSG00000206199	ENSG00000198483	ENSG00000120440
ENSG00000168615	ENSG00000197629	ENSG00000183155
ENSG00000196935	ENSG00000254535	ENSG00000141556
ENSG00000157450	ENSG00000165555	ENSG00000205856
ENSG00000151838	ENSG00000183729	ENSG00000095739
ENSG00000267618	ENSG00000170545	ENSG00000137269
ENSG00000145428	ENSG00000067182	ENSG00000198064
ENSG00000108256	ENSG00000170364	ENSG00000182372
ENSG00000100129	ENSG00000180999	ENSG00000161202
ENSG00000168944	ENSG00000159189	ENSG00000204788

ENSG00000177150	ENSG00000103353	ENSG00000107562
ENSG00000071677	ENSG00000186265	ENSG00000123130
ENSG00000172340	ENSG00000185104	ENSG00000114742
ENSG00000171295	ENSG00000147613	ENSG00000100442
ENSG00000071189	ENSG00000188133	ENSG00000172845
ENSG00000188895	ENSG00000153006	ENSG00000086205
ENSG00000184363	ENSG00000107447	ENSG00000239887
ENSG00000159322	ENSG00000168300	ENSG00000073969
ENSG00000151414	ENSG00000182712	ENSG00000157999
ENSG00000108242	ENSG00000182180	ENSG00000135413
ENSG00000170222	ENSG00000004139	ENSG00000125637
ENSG00000144161	ENSG00000135045	ENSG00000154174
ENSG00000119685	ENSG00000114062	ENSG00000214097
ENSG00000150459	ENSG00000173013	ENSG00000148154
ENSG00000188610	ENSG00000127948	ENSG00000174194
ENSG00000146733	ENSG00000021461	ENSG00000175581
ENSG00000175899	ENSG00000175229	ENSG00000124214
ENSG00000130340	ENSG00000106367	ENSG00000141699
ENSG00000137414	ENSG00000198270	ENSG00000114026
ENSG00000063322	ENSG00000181896	ENSG00000178537
ENSG00000149100	ENSG00000168556	ENSG00000079332
ENSG00000204178	ENSG00000188931	ENSG00000175143
ENSG00000101544	ENSG00000144589	ENSG00000186451
ENSG00000152503	ENSG00000111405	ENSG00000145241
ENSG00000197779	ENSG00000196700	ENSG00000114982
ENSG00000203815	ENSG00000119630	ENSG00000149591
ENSG00000073921	ENSG00000225683	ENSG00000111725
ENSG00000143363	ENSG00000152433	ENSG00000110906
ENSG00000135451	ENSG00000139617	ENSG00000128655
ENSG00000197953	ENSG00000188677	ENSG00000077514
ENSG00000138660	ENSG00000101049	ENSG00000100949
ENSG00000257218	ENSG00000170581	ENSG00000181689
ENSG00000047457	ENSG00000183549	ENSG00000143153
ENSG00000112394	ENSG00000163607	ENSG00000136628
ENSG00000196850	ENSG00000009307	ENSG00000164331
ENSG00000198814	ENSG00000162494	ENSG00000176204
ENSG00000103067	ENSG00000130764	ENSG00000110002
ENSG00000124693	ENSG00000182177	ENSG00000179165
ENSG00000133477	ENSG00000052841	ENSG00000169903
ENSG00000100028	ENSG00000110811	ENSG00000242866
ENSG00000166037	ENSG00000027644	ENSG00000108599
ENSG00000205174	ENSG00000042445	ENSG00000188501
ENSG00000198839	ENSG00000149305	ENSG00000268500
ENSG00000100580	ENSG00000104957	ENSG00000143971
ENSG00000166887	ENSG00000259075	ENSG00000172464
ENSG00000163584	ENSG00000103126	ENSG00000114656
ENSG00000243989	ENSG00000100142	ENSG00000143149
ENSG00000204839	ENSG00000158863	ENSG00000120952
ENSG00000125388	ENSG00000127564	ENSG00000035499
ENSG00000075290	ENSG00000006459	ENSG00000062598
ENSG00000162618	ENSG00000140990	ENSG00000087074
ENSG00000178750	ENSG00000148719	ENSG00000170260
ENSG00000175352	ENSG00000186895	ENSG00000172264
ENSG00000085760	ENSG00000130023	ENSG00000256394
ENSG00000198342	ENSG00000140854	ENSG00000104918
ENSG00000151835	ENSG00000125676	ENSG00000064115
ENSG00000179134	ENSG00000142765	ENSG00000197124
ENSG00000116221	ENSG00000122592	ENSG00000115539
ENSG00000077809	ENSG00000125999	ENSG00000215099
ENSG00000167702	ENSG00000064547	ENSG00000267281
ENSG00000165288	ENSG00000055955	ENSG00000145975
ENSG00000106638	ENSG00000205464	ENSG00000126890
ENSG00000042813	ENSG00000143622	ENSG00000000005
ENSG00000196387	ENSG00000116521	ENSG00000166596

ENSG00000198060	ENSG00000112761	ENSG00000102069
ENSG00000253327	ENSG00000140807	ENSG00000174453
ENSG00000176542	ENSG00000187116	ENSG00000153879
ENSG00000101811	ENSG00000154493	ENSG00000112699
ENSG00000130159	ENSG00000072080	ENSG00000112640
ENSG00000104177	ENSG00000125740	ENSG00000179532
ENSG00000129657	ENSG00000021488	ENSG00000198951
ENSG00000153898	ENSG00000132911	ENSG00000263563
ENSG00000172322	ENSG00000161277	ENSG00000104731
ENSG00000196131	ENSG00000196839	ENSG00000096063
ENSG00000118733	ENSG00000104755	ENSG00000183644
ENSG00000141570	ENSG00000139266	ENSG00000112531
ENSG00000182768	ENSG00000112130	ENSG00000171951
ENSG00000124593	ENSG00000184945	ENSG00000204020
ENSG00000175104	ENSG00000081181	ENSG00000170956
ENSG00000131263	ENSG00000185697	ENSG00000068308
ENSG00000134352	ENSG00000168530	ENSG00000166391
ENSG00000143476	ENSG00000184076	ENSG00000146373
ENSG00000165066	ENSG00000071539	ENSG00000083312
ENSG00000186684	ENSG00000174145	ENSG00000137161
ENSG00000196597	ENSG00000101474	ENSG00000143434
ENSG00000154479	ENSG00000197302	ENSG00000005187
ENSG00000138294	ENSG00000086504	ENSG00000118894
ENSG00000073417	ENSG00000011523	ENSG00000254093
ENSG00000255212	ENSG00000101752	ENSG00000114541
ENSG00000183283	ENSG00000197879	ENSG00000173838
ENSG00000086589	ENSG00000042286	ENSG00000137070
ENSG00000213171	ENSG00000019485	ENSG00000092208
ENSG00000179528	ENSG00000100226	ENSG00000152894
ENSG00000118849	ENSG00000185156	ENSG00000117472
ENSG00000144802	ENSG00000137343	ENSG00000005020
ENSG00000138795	ENSG00000161905	ENSG00000159873
ENSG00000110060	ENSG00000221972	ENSG00000120686
ENSG00000243710	ENSG00000129946	ENSG00000135040
ENSG00000122126	ENSG00000258890	ENSG00000108231
ENSG00000140297	ENSG00000159685	ENSG00000120647
ENSG00000268790	ENSG00000151491	ENSG00000160305
ENSG00000001561	ENSG00000171236	ENSG00000186838
ENSG00000073605	ENSG00000186795	ENSG00000108344
ENSG00000124831	ENSG00000197938	ENSG00000122692
ENSG00000196126	ENSG00000053501	ENSG00000075884
ENSG00000111707	ENSG00000138246	ENSG00000138101
ENSG00000091164	ENSG00000197548	ENSG00000171928
ENSG00000089335	ENSG00000204513	ENSG00000056661
ENSG00000121486	ENSG00000128606	ENSG00000231171
ENSG00000125246	ENSG00000117448	ENSG00000100324
ENSG00000134058	ENSG00000145506	ENSG00000126351
ENSG00000123095	ENSG00000106483	ENSG00000149201
ENSG00000100285	ENSG00000119862	ENSG00000197935
ENSG00000173230	ENSG00000214290	ENSG00000180532
ENSG00000136487	ENSG00000183172	ENSG00000151692
ENSG00000112759	ENSG00000072422	ENSG00000236032
ENSG00000133606	ENSG00000100916	ENSG00000196188
ENSG00000113441	ENSG00000176988	ENSG00000125780
ENSG00000118298	ENSG00000176209	ENSG00000179615
ENSG00000163563	ENSG00000136367	ENSG00000178685
ENSG00000132646	ENSG00000116604	ENSG00000143393
ENSG00000176840	ENSG00000258817	ENSG00000125351
ENSG00000135801	ENSG00000125652	ENSG00000091181
ENSG00000205108	ENSG00000162869	ENSG00000126581
ENSG00000173542	ENSG00000115165	ENSG00000169085
ENSG00000137815	ENSG00000120440	ENSG00000118985
ENSG00000176253	ENSG00000090238	ENSG00000173467
ENSG00000168439	ENSG00000183155	ENSG00000180708

ENSG00000150455	ENSG00000176058	ENSG00000163114
ENSG00000138071	ENSG00000250479	ENSG00000164746
ENSG00000132357	ENSG00000141556	ENSG00000205669
ENSG00000158941	ENSG00000161847	ENSG00000166483
ENSG00000105472	ENSG00000205856	ENSG00000163597
ENSG00000188659	ENSG00000095739	ENSG00000170312
ENSG00000139618	ENSG00000137269	ENSG00000182168
ENSG00000112893	ENSG00000198064	ENSG00000163950
ENSG00000197857	ENSG00000182372	ENSG00000170558
ENSG00000196184	ENSG00000183655	ENSG00000149651
ENSG00000183605	ENSG00000161202	ENSG00000095574
ENSG00000170584	ENSG00000204788	ENSG00000206530
ENSG00000257242	ENSG00000107562	ENSG00000112425
ENSG00000128578	ENSG00000123130	ENSG00000198169
ENSG00000136929	ENSG00000114742	ENSG00000222014
ENSG00000197888	ENSG00000100442	ENSG00000058804
ENSG00000182541	ENSG00000172845	ENSG00000003989
ENSG00000140740	ENSG00000116649	ENSG00000145907
ENSG00000122679	ENSG00000086205	ENSG00000105698
ENSG00000143575	ENSG00000239887	ENSG00000173641
ENSG00000050130	ENSG00000073969	ENSG00000160917
ENSG00000124523	ENSG00000157999	ENSG00000104081
ENSG00000188629	ENSG00000135413	ENSG00000185945
ENSG00000206560	ENSG00000105370	ENSG00000135392
ENSG00000166401	ENSG00000125637	ENSG00000171931
ENSG00000248710	ENSG00000154174	ENSG00000173908
ENSG00000002745	ENSG00000214097	ENSG00000131089
ENSG00000131748	ENSG00000254470	ENSG00000113790
ENSG00000262314	ENSG00000148154	ENSG00000222018
ENSG00000163251	ENSG00000174194	ENSG00000139780
ENSG00000226974	ENSG00000175581	ENSG00000175445
ENSG00000134020	ENSG00000124214	ENSG00000145777
ENSG00000215372	ENSG00000141699	ENSG00000116761
ENSG00000084090	ENSG00000114026	ENSG00000119508
ENSG00000109452	ENSG00000144152	ENSG00000180198
ENSG00000134323	ENSG00000099985	ENSG00000150593
ENSG00000170448	ENSG00000178537	ENSG00000117335
ENSG00000234444	ENSG00000161653	ENSG00000197635
ENSG00000237441	ENSG00000079332	ENSG00000102316
ENSG00000204174	ENSG00000108379	ENSG00000235109
ENSG00000166926	ENSG00000175143	ENSG00000147124
ENSG00000170835	ENSG00000186451	ENSG00000101138
ENSG00000198920	ENSG00000145241	ENSG00000197050
ENSG00000140379	ENSG00000171060	ENSG00000118482
ENSG00000155749	ENSG00000114982	ENSG00000123561
ENSG00000103502	ENSG00000149591	ENSG00000156140
ENSG00000180035	ENSG00000111725	ENSG00000231192
ENSG00000181458	ENSG00000110906	ENSG00000198382
ENSG00000072657	ENSG00000128655	ENSG00000144355
ENSG00000176102	ENSG00000077514	ENSG00000197037
ENSG00000182700	ENSG00000100949	ENSG00000105321
ENSG00000162782	ENSG00000181689	ENSG00000155229
ENSG00000182004	ENSG00000143153	ENSG00000185332
ENSG00000154359	ENSG00000163286	ENSG00000160325
ENSG00000206559	ENSG00000136628	ENSG00000160323
ENSG00000155008	ENSG00000164331	ENSG00000198453
ENSG00000143196	ENSG00000176204	ENSG00000001167
ENSG00000221977	ENSG00000110002	ENSG00000110429
ENSG00000013523	ENSG00000179165	ENSG00000155249
ENSG00000123405	ENSG00000169903	ENSG00000177981
ENSG00000112739	ENSG00000242866	ENSG00000061918
ENSG00000027001	ENSG00000108599	ENSG00000171467
ENSG00000111832	ENSG00000188501	ENSG00000174939
ENSG00000205495	ENSG00000268500	ENSG00000164304

ENSG00000169230	ENSG00000143971	ENSG00000171241
ENSG00000130165	ENSG00000172464	ENSG00000164089
ENSG00000150401	ENSG00000114656	ENSG00000143614
ENSG00000140259	ENSG00000143149	ENSG00000243130
ENSG00000189319	ENSG00000120952	ENSG00000185291
ENSG00000177943	ENSG00000035499	ENSG00000166800
ENSG00000164404	ENSG00000062598	ENSG00000181929
ENSG00000144591	ENSG00000087074	ENSG00000065802
ENSG00000196781	ENSG00000170260	ENSG00000106799
ENSG00000156502	ENSG00000172264	ENSG00000116791
ENSG00000185053	ENSG00000256394	ENSG00000169908
ENSG00000172687	ENSG00000104918	ENSG00000102271
ENSG00000240720	ENSG00000064115	ENSG00000136696
ENSG00000024526	ENSG00000197124	ENSG00000129226
ENSG00000103494	ENSG00000115539	ENSG00000171448
ENSG00000008869	ENSG00000215099	ENSG00000158769
ENSG00000110934	ENSG00000267281	ENSG00000176024
ENSG00000164938	ENSG00000145975	ENSG00000103707
ENSG00000157193	ENSG00000126890	ENSG00000183742
ENSG00000023445	ENSG00000000005	ENSG00000111843
ENSG00000134597	ENSG00000166596	ENSG00000179104
ENSG00000064933	ENSG00000102069	ENSG00000128928
ENSG00000159840	ENSG00000197891	ENSG00000256525
ENSG00000125629	ENSG00000174453	ENSG00000108578
ENSG00000101346	ENSG00000153879	ENSG00000135097
ENSG00000134698	ENSG00000112699	ENSG00000145375
ENSG00000002919	ENSG00000112640	ENSG00000100003
ENSG00000174600	ENSG00000117984	ENSG00000064309
ENSG00000170871	ENSG00000205220	ENSG00000102524
ENSG00000180287	ENSG00000179532	ENSG00000204852
ENSG00000035115	ENSG00000129437	ENSG00000134779
ENSG00000203827	ENSG00000214717	ENSG00000145632
ENSG00000143515	ENSG00000198951	ENSG00000150433
ENSG00000105793	ENSG00000263563	ENSG00000185418
ENSG00000182362	ENSG00000104731	ENSG00000183067
ENSG00000141469	ENSG00000096063	ENSG00000116690
ENSG00000163536	ENSG00000183644	ENSG00000148735
ENSG00000114786	ENSG00000112531	ENSG00000161921
ENSG00000119684	ENSG00000171951	ENSG00000141429
ENSG00000151575	ENSG00000204020	ENSG00000186625
ENSG00000111786	ENSG00000162728	ENSG00000113303
ENSG00000113594	ENSG00000109736	ENSG00000103507
ENSG00000168961	ENSG00000115756	ENSG00000165525
ENSG00000196655	ENSG00000170956	ENSG00000128519
ENSG00000237765	ENSG00000068308	ENSG00000162892
ENSG00000166619	ENSG00000125656	ENSG00000136872
ENSG00000168273	ENSG00000166391	ENSG00000146857
ENSG00000154548	ENSG00000146373	ENSG00000011007
ENSG00000141367	ENSG00000083312	ENSG00000168124
ENSG00000104059	ENSG00000137161	ENSG00000136867
ENSG00000213901	ENSG00000143434	ENSG00000100351
ENSG00000163131	ENSG00000106683	ENSG00000188921
ENSG00000011260	ENSG00000005187	ENSG00000152661
ENSG00000107581	ENSG00000118894	ENSG00000267629
ENSG00000159335	ENSG00000254093	ENSG00000080986
ENSG00000167900	ENSG00000114541	ENSG00000107897
ENSG00000237693	ENSG00000173838	ENSG00000188690
ENSG00000007312	ENSG00000137070	ENSG00000136152
ENSG00000124103	ENSG00000092208	ENSG00000206418
ENSG00000136436	ENSG00000152894	ENSG00000151623
ENSG00000071626	ENSG00000117472	ENSG00000196431
ENSG00000115419	ENSG00000005020	ENSG00000254415
ENSG00000100565	ENSG00000159873	ENSG00000143319
ENSG00000244462	ENSG00000120686	ENSG00000204427

ENSG00000187815	ENSG00000135040	ENSG00000108515
ENSG00000151116	ENSG00000100726	ENSG00000112796
ENSG00000144648	ENSG00000108231	ENSG00000164393
ENSG00000196975	ENSG00000120647	ENSG00000159079
ENSG00000110700	ENSG00000182324	ENSG00000180537
ENSG00000163935	ENSG00000160305	ENSG00000090621
ENSG00000084764	ENSG00000186838	ENSG00000141030
ENSG00000170949	ENSG00000108344	ENSG00000183098
ENSG00000112592	ENSG00000074855	ENSG00000196826
ENSG00000116754	ENSG00000122692	ENSG00000212657
ENSG00000112305	ENSG00000075884	ENSG00000065427
ENSG00000082438	ENSG00000100417	ENSG00000196605
ENSG00000256229	ENSG00000182557	ENSG00000147251
ENSG00000019995	ENSG00000138101	ENSG00000154889
ENSG00000126249	ENSG00000215455	ENSG00000132640
ENSG00000134057	ENSG00000228835	ENSG00000109132
ENSG00000185899	ENSG00000171928	ENSG00000188761
ENSG00000179152	ENSG00000056661	ENSG00000204365
ENSG00000137473	ENSG00000231171	ENSG00000205784
ENSG00000101558	ENSG00000100324	ENSG00000103995
ENSG00000178935	ENSG00000126351	ENSG00000075142
ENSG00000105778	ENSG00000149201	ENSG00000081148
ENSG00000144357	ENSG00000197935	ENSG00000127530
ENSG00000198846	ENSG00000180532	ENSG00000177873
ENSG00000100802	ENSG00000151692	ENSG00000035720
ENSG00000166860	ENSG00000236032	ENSG00000166884
ENSG00000179344	ENSG00000196188	ENSG00000145725
ENSG00000169251	ENSG00000125780	ENSG00000089356
ENSG00000018510	ENSG00000179615	ENSG00000178462
ENSG00000143862	ENSG00000178685	ENSG00000148290
ENSG00000187514	ENSG00000130711	ENSG00000154001
ENSG00000138347	ENSG00000143393	ENSG00000155754
ENSG00000116874	ENSG00000125351	ENSG00000113396
ENSG00000119888	ENSG00000091181	ENSG00000102007
ENSG00000174442	ENSG00000167757	ENSG00000095970
ENSG00000172399	ENSG00000126581	ENSG00000197837
ENSG00000109919	ENSG00000169085	ENSG00000116668
ENSG00000204873	ENSG00000118985	ENSG00000203784
ENSG00000009950	ENSG00000173467	ENSG00000075131
ENSG00000155761	ENSG00000106100	ENSG00000178952
ENSG00000170100	ENSG00000145882	ENSG00000120910
ENSG00000237649	ENSG00000180708	ENSG00000146700
ENSG00000167977	ENSG00000163114	ENSG00000185624
ENSG00000104218	ENSG00000198083	ENSG00000124641
ENSG00000186352	ENSG00000007255	ENSG00000059145
ENSG00000078304	ENSG00000164746	ENSG00000250021
ENSG00000078246	ENSG00000205669	ENSG00000110435
ENSG00000167720	ENSG00000166483	ENSG00000117632
ENSG00000120451	ENSG00000163597	ENSG00000138641
ENSG00000172014	ENSG00000170312	ENSG00000122729
ENSG00000108187	ENSG00000182168	ENSG00000184032
ENSG00000117133	ENSG00000163950	ENSG00000129244
ENSG00000127311	ENSG00000170558	ENSG00000176476
ENSG00000180263	ENSG00000149651	ENSG00000080644
ENSG00000176182	ENSG00000095574	ENSG00000169764
ENSG00000165802	ENSG00000206530	ENSG00000124787
ENSG00000196632	ENSG00000112425	ENSG00000163710
ENSG00000257198	ENSG00000198169	ENSG00000176879
ENSG00000163806	ENSG00000222014	ENSG00000171561
ENSG00000136197	ENSG00000160789	ENSG00000172469
ENSG00000198015	ENSG00000058804	ENSG00000237149
ENSG00000156976	ENSG00000180044	ENSG00000165457
ENSG00000134962	ENSG00000003989	ENSG00000188487
ENSG00000166855	ENSG00000145907	ENSG00000185888

ENSG00000131480	ENSG00000171302	ENSG00000227839
ENSG00000128191	ENSG00000105698	ENSG00000198081
ENSG00000117620	ENSG00000173641	ENSG00000133636
ENSG00000176208	ENSG00000101412	ENSG00000187806
ENSG00000120509	ENSG00000160917	ENSG00000196074
ENSG00000136603	ENSG00000104081	ENSG00000099917
ENSG00000186275	ENSG00000185945	ENSG00000175390
ENSG00000205809	ENSG00000184348	ENSG00000164251
ENSG00000176294	ENSG00000135392	ENSG00000146457
ENSG00000113068	ENSG00000162426	ENSG00000145734
ENSG00000144840	ENSG00000171931	ENSG00000118762
ENSG00000136108	ENSG00000003137	ENSG00000204661
ENSG00000184502	ENSG00000173908	ENSG00000105609
ENSG00000067900	ENSG00000131089	ENSG00000145425
ENSG00000213416	ENSG00000113790	ENSG00000104679
ENSG00000082458	ENSG00000222018	ENSG00000088325
ENSG00000165660	ENSG00000123689	ENSG00000125454
ENSG00000184650	ENSG00000139780	ENSG00000118193
ENSG00000122958	ENSG00000175445	ENSG00000157895
ENSG00000251503	ENSG00000145777	ENSG00000133195
ENSG00000129235	ENSG00000116761	ENSG00000213658
ENSG00000109111	ENSG00000169840	ENSG00000205560
ENSG00000086232	ENSG00000121413	ENSG00000118855
ENSG00000166432	ENSG00000167617	ENSG00000104154
ENSG00000109445	ENSG00000119508	ENSG00000015592
ENSG00000153107	ENSG00000180198	ENSG00000144224
ENSG00000198876	ENSG00000150593	ENSG00000154143
ENSG00000169116	ENSG00000117335	ENSG00000196329
ENSG00000114030	ENSG00000197635	ENSG00000089682
ENSG00000124541	ENSG00000101203	ENSG00000174132
ENSG00000142528	ENSG00000102316	ENSG00000172794
ENSG00000188321	ENSG00000235109	ENSG00000069998
ENSG00000131171	ENSG00000147124	ENSG00000171509
ENSG00000188611	ENSG00000101138	ENSG00000161956
ENSG00000181035	ENSG00000197050	ENSG00000166105
ENSG00000106823	ENSG00000118482	ENSG00000116213
ENSG00000182327	ENSG00000123561	ENSG00000128245
ENSG00000152726	ENSG00000156140	ENSG00000170903
ENSG00000150636	ENSG00000231192	ENSG00000131148
ENSG00000165181	ENSG00000198382	ENSG00000177570
ENSG00000113569	ENSG00000144355	ENSG00000253910
ENSG00000164256	ENSG00000197037	ENSG00000168802
ENSG00000196099	ENSG00000105321	ENSG00000244754
ENSG00000137193	ENSG00000155229	ENSG00000184905
ENSG00000119640	ENSG00000185332	ENSG00000158477
ENSG00000157107	ENSG00000160325	ENSG00000124145
ENSG00000138640	ENSG00000160323	ENSG00000149557
ENSG00000196139	ENSG00000198453	ENSG00000112210
ENSG00000129493	ENSG00000001167	ENSG00000214595
ENSG00000115977	ENSG00000110429	ENSG00000122224
ENSG00000172660	ENSG00000155249	ENSG00000125384
ENSG00000164961	ENSG00000177981	ENSG00000241644
ENSG00000083720	ENSG00000061918	ENSG00000163818
ENSG00000171649	ENSG00000171467	ENSG00000189233
ENSG00000112874	ENSG00000174939	ENSG00000135362
ENSG00000151657	ENSG00000164304	ENSG00000257727
ENSG00000184434	ENSG00000171241	ENSG00000186197
ENSG00000155542	ENSG00000164089	ENSG00000137513
ENSG00000089234	ENSG00000170075	ENSG00000134940
ENSG00000177311	ENSG00000143614	ENSG00000122497
ENSG00000001084	ENSG00000243130	ENSG00000172146
ENSG00000100485	ENSG00000185291	ENSG00000169189
ENSG00000173818	ENSG00000166800	ENSG00000125630
ENSG00000108733	ENSG00000107021	ENSG00000185220

ENSG00000023287	ENSG00000181929	ENSG00000106635
ENSG00000198283	ENSG00000065802	ENSG00000233828
ENSG00000180574	ENSG00000007866	ENSG00000129195
ENSG00000101577	ENSG00000106799	ENSG00000222046
ENSG00000151014	ENSG00000116791	ENSG00000120699
ENSG00000221947	ENSG00000239886	ENSG00000155957
ENSG00000188100	ENSG00000169908	ENSG00000187736
ENSG00000196507	ENSG00000154319	ENSG00000131504
ENSG00000163527	ENSG00000102271	ENSG00000081307
ENSG00000197121	ENSG00000136696	ENSG00000101849
ENSG00000163239	ENSG00000129226	ENSG00000111404
ENSG00000185480	ENSG00000113722	ENSG00000133134
ENSG00000104765	ENSG00000104969	ENSG00000159267
ENSG00000157181	ENSG00000171448	ENSG00000016402
ENSG00000055208	ENSG00000158769	ENSG00000132429
ENSG00000162735	ENSG00000176024	ENSG00000152464
ENSG00000115232	ENSG00000103707	ENSG00000049245
ENSG00000065833	ENSG00000183742	ENSG00000124688
ENSG00000120289	ENSG00000007216	ENSG00000109743
ENSG00000136143	ENSG00000111843	ENSG00000071282
ENSG00000101158	ENSG00000179104	ENSG00000159579
ENSG00000268107	ENSG00000128928	ENSG00000153560
ENSG00000172243	ENSG00000176490	ENSG00000161011
ENSG00000175161	ENSG00000256525	ENSG00000241370
ENSG00000135241	ENSG00000101470	ENSG00000170296
ENSG00000137463	ENSG00000106327	ENSG00000184007
ENSG00000178741	ENSG00000108578	ENSG00000122378
ENSG00000221845	ENSG00000135097	ENSG00000242221
ENSG00000198765	ENSG00000145375	ENSG00000122335
ENSG00000172007	ENSG00000100003	ENSG00000257726
ENSG00000164292	ENSG00000064309	ENSG00000184611
ENSG00000123171	ENSG00000102524	ENSG00000175348
ENSG00000091157	ENSG00000204852	ENSG00000236499
ENSG00000240065	ENSG00000187840	ENSG00000100296
ENSG00000151233	ENSG00000134779	ENSG00000110619
ENSG00000075568	ENSG00000145632	ENSG00000096996
ENSG00000213121	ENSG00000150433	ENSG00000255561
ENSG00000167618	ENSG00000185418	ENSG00000117226
ENSG00000113312	ENSG00000183067	ENSG00000139323
ENSG00000173207	ENSG00000116690	ENSG00000172208
ENSG00000171490	ENSG00000183048	ENSG00000225781
ENSG00000267918	ENSG00000148735	ENSG00000106025
ENSG00000102172	ENSG00000161921	ENSG00000158805
ENSG00000107201	ENSG00000141429	ENSG00000118513
ENSG00000130303	ENSG00000186625	ENSG00000134461
ENSG00000082269	ENSG00000113303	ENSG00000160193
ENSG00000188227	ENSG00000103507	ENSG00000180138
ENSG00000125812	ENSG00000165525	ENSG00000167778
ENSG00000126215	ENSG00000128519	ENSG00000109272
ENSG00000188707	ENSG00000162892	ENSG00000179636
ENSG00000234284	ENSG00000136872	ENSG00000082126
ENSG00000090061	ENSG00000196372	ENSG00000256053
ENSG00000114374	ENSG00000146857	ENSG00000267179
ENSG00000133835	ENSG00000011007	ENSG00000130768
ENSG00000069275	ENSG00000168124	ENSG00000204393
ENSG00000178055	ENSG00000136867	ENSG00000101084
ENSG00000115415	ENSG00000154768	ENSG00000105364
ENSG00000117016	ENSG00000100351	ENSG00000185962
ENSG00000196890	ENSG00000188921	ENSG00000214872
ENSG00000108849	ENSG00000185340	ENSG00000162599
ENSG00000197299	ENSG00000152661	ENSG00000163209
ENSG00000138079	ENSG00000267629	ENSG00000102218
ENSG00000173335	ENSG00000080986	ENSG00000168329
ENSG00000072042	ENSG00000099624	ENSG00000159792

ENSG00000268467	ENSG00000107897	ENSG00000061987
ENSG00000175393	ENSG00000160994	ENSG00000131495
ENSG00000171657	ENSG00000188690	ENSG00000197182
ENSG00000077616	ENSG00000136152	ENSG00000140403
ENSG00000119953	ENSG00000206418	ENSG00000262628
ENSG00000170323	ENSG00000151623	ENSG00000173409
ENSG00000129170	ENSG00000196431	ENSG00000108406
ENSG00000020426	ENSG00000254415	ENSG00000164096
ENSG00000138430	ENSG00000143319	ENSG00000034971
ENSG00000089157	ENSG00000204427	ENSG00000083845
ENSG00000163098	ENSG00000267534	ENSG00000198601
ENSG00000113522	ENSG00000162344	ENSG00000087338
ENSG00000137814	ENSG00000108515	ENSG00000105967
ENSG00000187871	ENSG00000112796	ENSG00000100997
ENSG00000143870	ENSG00000164393	ENSG00000162543
ENSG00000138496	ENSG00000159079	ENSG00000197309
ENSG00000119318	ENSG00000180537	ENSG00000198346
ENSG00000166348	ENSG00000090621	ENSG00000121377
ENSG00000257743	ENSG00000141030	ENSG00000178035
ENSG00000266118	ENSG00000183098	ENSG00000104907
ENSG00000124784	ENSG00000196826	ENSG00000087263
ENSG00000112276	ENSG00000212657	ENSG00000152292
ENSG00000249459	ENSG00000065427	ENSG00000139304
ENSG00000150556	ENSG00000196605	ENSG00000169740
ENSG00000227124	ENSG00000147251	ENSG00000148386
ENSG00000198682	ENSG00000100302	ENSG00000111305
ENSG00000197020	ENSG00000154889	ENSG00000078369
ENSG00000131507	ENSG00000132640	ENSG00000172059
ENSG00000163795	ENSG00000109132	ENSG00000172199
ENSG00000158882	ENSG00000188761	ENSG00000115761
ENSG00000139734	ENSG00000204365	ENSG00000152520
ENSG00000172071	ENSG00000205784	ENSG00000166780
ENSG00000137218	ENSG00000103995	ENSG00000125779
ENSG00000104044	ENSG00000075142	ENSG00000078579
ENSG00000158545	ENSG00000130822	ENSG00000228368
ENSG00000109511	ENSG00000081148	ENSG00000074219
ENSG00000106392	ENSG00000127530	ENSG00000114999
ENSG00000164118	ENSG00000177873	ENSG00000186652
ENSG00000103037	ENSG00000035720	ENSG00000180801
ENSG00000187741	ENSG00000171532	ENSG00000085832
ENSG00000133302	ENSG00000166884	ENSG00000076706
ENSG00000122970	ENSG00000180767	ENSG00000170515
ENSG00000133983	ENSG00000145725	ENSG00000204590
ENSG00000197780	ENSG00000146094	ENSG00000143753
ENSG00000182307	ENSG00000089356	ENSG00000173214
ENSG00000108384	ENSG00000159640	ENSG00000145808
ENSG00000111615	ENSG00000178462	ENSG00000072756
ENSG00000079263	ENSG00000148290	ENSG00000140691
ENSG00000135437	ENSG00000154001	ENSG00000158828
ENSG00000104953	ENSG00000151365	ENSG00000101361
ENSG00000130787	ENSG00000155754	ENSG00000107968
ENSG00000138303	ENSG00000113396	ENSG00000164645
ENSG00000135486	ENSG00000102007	ENSG00000204695
ENSG00000105202	ENSG00000095970	ENSG00000183474
ENSG00000138459	ENSG00000197837	ENSG00000143771
ENSG00000120438	ENSG00000116668	ENSG00000129187
ENSG00000124205	ENSG00000203784	ENSG00000154309
ENSG00000184508	ENSG00000075131	ENSG00000103023
ENSG00000197753	ENSG00000178952	ENSG00000151704
ENSG00000164346	ENSG00000120910	ENSG00000172269
ENSG00000170917	ENSG00000146700	ENSG00000125037
ENSG00000256043	ENSG00000185624	ENSG00000165474
ENSG00000143933	ENSG00000138030	ENSG00000196934
ENSG00000165637	ENSG00000124641	ENSG00000180008

ENSG00000135655	ENSG00000059145	ENSG00000111445
ENSG00000182831	ENSG00000250021	ENSG00000198925
ENSG00000163689	ENSG00000110435	ENSG00000154832
ENSG00000148660	ENSG00000117632	ENSG00000137496
ENSG00000215186	ENSG00000138641	ENSG00000196865
ENSG00000136521	ENSG00000122729	ENSG00000131899
ENSG00000204655	ENSG00000184032	ENSG00000095981
ENSG00000150776	ENSG00000129244	ENSG00000132517
ENSG00000104290	ENSG00000176476	ENSG00000267680
ENSG00000111713	ENSG00000080644	ENSG00000136869
ENSG00000115361	ENSG00000169764	ENSG00000149084
ENSG00000187239	ENSG00000124787	ENSG00000135900
ENSG00000177888	ENSG00000163710	ENSG00000188817
ENSG00000156738	ENSG00000176879	ENSG00000151617
ENSG00000104497	ENSG00000171561	ENSG00000203747
ENSG00000131845	ENSG00000198914	ENSG00000214107
ENSG00000136155	ENSG00000172469	ENSG00000100024
ENSG00000115604	ENSG00000102924	ENSG00000104231
ENSG00000162813	ENSG00000237149	ENSG00000159884
ENSG00000183036	ENSG00000177666	ENSG00000230891
ENSG00000046889	ENSG00000165457	ENSG00000111834
ENSG00000179562	ENSG00000160439	ENSG00000101182
ENSG00000084444	ENSG00000188487	ENSG00000119929
ENSG00000013441	ENSG00000185888	ENSG00000125977
ENSG00000143549	ENSG00000227839	ENSG00000112599
ENSG00000212127	ENSG00000198081	ENSG00000068615
ENSG00000197008	ENSG00000133636	ENSG00000122877
ENSG00000116857	ENSG00000187806	ENSG00000125954
ENSG00000121335	ENSG00000196074	ENSG00000188001
ENSG00000117569	ENSG00000099917	ENSG00000168275
ENSG00000114023	ENSG00000115257	ENSG00000197568
ENSG00000152127	ENSG00000175390	ENSG00000106263
ENSG00000136243	ENSG00000164251	ENSG00000136156
ENSG00000158292	ENSG00000146457	ENSG00000176160
ENSG00000078699	ENSG00000145734	ENSG00000198363
ENSG00000183248	ENSG00000118762	ENSG00000188848
ENSG00000109805	ENSG00000204661	ENSG00000132405
ENSG00000166181	ENSG00000105609	ENSG00000144771
ENSG00000160714	ENSG00000145425	ENSG00000206073
ENSG00000141452	ENSG00000246705	ENSG00000104435
ENSG00000167555	ENSG00000124302	ENSG00000103111
ENSG00000148700	ENSG00000104679	ENSG00000155016
ENSG00000213281	ENSG00000088325	ENSG00000137494
ENSG00000004799	ENSG00000125454	ENSG00000129151
ENSG00000125257	ENSG00000118193	ENSG00000137500
ENSG00000101844	ENSG00000157895	ENSG00000155380
ENSG00000103061	ENSG00000170498	ENSG00000163586
ENSG00000186212	ENSG00000133195	ENSG00000158301
ENSG00000113889	ENSG00000213658	ENSG00000138092
ENSG00000068078	ENSG00000205560	ENSG00000142186
ENSG00000127870	ENSG00000143761	ENSG00000186074
ENSG00000152291	ENSG00000070404	ENSG00000196141
ENSG00000115145	ENSG00000118855	ENSG00000186184
ENSG00000166912	ENSG00000239732	ENSG00000196166
ENSG00000154589	ENSG00000104154	ENSG00000215203
ENSG00000133640	ENSG00000015592	ENSG00000184385
ENSG00000152229	ENSG00000144224	ENSG00000188888
ENSG00000232119	ENSG00000126903	ENSG00000131459
ENSG00000224659	ENSG00000154143	ENSG00000144134
ENSG00000213625	ENSG00000196329	ENSG00000198390
ENSG00000189134	ENSG00000089682	ENSG00000198324
ENSG00000174738	ENSG00000174132	ENSG00000143543
ENSG00000159961	ENSG00000172794	ENSG00000175121
ENSG00000137869	ENSG00000069998	ENSG00000105379

ENSG00000197838	ENSG00000187821	ENSG00000137819
ENSG00000205678	ENSG00000171509	ENSG00000127824
ENSG00000130363	ENSG00000161956	ENSG00000177335
ENSG00000166261	ENSG00000188778	ENSG00000111058
ENSG00000106868	ENSG00000166105	ENSG00000152061
ENSG00000136147	ENSG00000116213	ENSG00000133800
ENSG00000181273	ENSG00000128245	ENSG00000155876
ENSG00000144120	ENSG00000170903	ENSG00000175182
ENSG00000158406	ENSG00000130487	ENSG00000182504
ENSG00000176407	ENSG00000131148	ENSG00000177575
ENSG00000105954	ENSG00000177570	ENSG00000171150
ENSG00000139133	ENSG00000253910	ENSG00000244045
ENSG00000135363	ENSG00000168802	ENSG00000100842
ENSG00000174844	ENSG00000244754	ENSG00000186648
ENSG00000109943	ENSG00000184905	ENSG00000099901
ENSG00000111536	ENSG00000158477	ENSG00000072274
ENSG00000159197	ENSG00000124145	ENSG00000155324
ENSG00000087502	ENSG00000149557	ENSG00000116698
ENSG00000197771	ENSG00000112210	ENSG00000134508
ENSG00000165819	ENSG00000214595	ENSG00000233224
ENSG00000076242	ENSG00000122224	ENSG00000257446
ENSG00000215114	ENSG00000142552	ENSG00000188452
ENSG00000156876	ENSG00000125384	ENSG00000116701
ENSG00000106992	ENSG00000241644	ENSG00000196345
ENSG00000159239	ENSG00000163818	ENSG00000048540
ENSG00000165410	ENSG00000189233	ENSG00000174951
ENSG00000075239	ENSG00000124104	ENSG00000074416
ENSG00000134077	ENSG00000135362	ENSG00000129277
ENSG00000143382	ENSG00000257727	ENSG00000164587
ENSG00000049249	ENSG00000186197	ENSG00000198755
ENSG00000067596	ENSG00000137513	ENSG00000152689
ENSG00000101109	ENSG00000134940	ENSG00000090013
ENSG00000100478	ENSG00000101986	ENSG00000137693
ENSG00000104371	ENSG00000122497	ENSG00000039600
ENSG00000204022	ENSG00000172146	ENSG00000168066
ENSG00000125304	ENSG00000169189	ENSG00000147889
ENSG00000176884	ENSG00000125630	ENSG00000029993
ENSG00000116985	ENSG00000007376	ENSG00000184564
ENSG00000130935	ENSG00000160877	ENSG00000171564
ENSG00000067646	ENSG00000185220	ENSG00000167625
ENSG00000138363	ENSG00000106635	ENSG00000198739
ENSG00000164209	ENSG00000105997	ENSG00000116991
ENSG00000173200	ENSG00000233828	ENSG00000118707
ENSG00000106723	ENSG00000129195	ENSG00000149212
ENSG00000070761	ENSG00000222046	ENSG00000136933
ENSG00000181092	ENSG00000120699	ENSG00000179914
ENSG00000174740	ENSG00000155957	ENSG00000187510
ENSG00000125879	ENSG00000131398	ENSG00000082512
ENSG00000181610	ENSG00000187736	ENSG00000131969
ENSG00000122550	ENSG00000131504	ENSG00000077157
ENSG00000203880	ENSG00000081307	ENSG00000163661
ENSG00000178809	ENSG00000160013	ENSG00000163424
ENSG00000100207	ENSG00000101849	ENSG00000102471
ENSG00000130348	ENSG00000111404	ENSG00000134490
ENSG00000052723	ENSG00000167244	ENSG00000100439
ENSG00000169067	ENSG00000184497	ENSG00000143479
ENSG00000233404	ENSG00000133134	ENSG00000121381
ENSG00000168297	ENSG00000183196	ENSG00000068781
ENSG00000147027	ENSG00000159267	ENSG00000129596
ENSG00000105852	ENSG00000016402	ENSG00000148688
ENSG00000144048	ENSG00000205362	ENSG00000092295
ENSG00000171488	ENSG00000132429	ENSG00000156219
ENSG00000168003	ENSG00000152464	ENSG00000133627
ENSG00000114771	ENSG00000049245	ENSG00000008294

ENSG00000065559	ENSG00000124688	ENSG00000187857
ENSG00000197070	ENSG00000109743	ENSG00000149582
ENSG00000178105	ENSG00000071282	ENSG00000100523
ENSG00000255713	ENSG00000159579	ENSG00000076554
ENSG00000161929	ENSG00000153560	ENSG00000226397
ENSG00000175309	ENSG00000161011	ENSG00000162627
ENSG00000153993	ENSG00000241370	ENSG00000180483
ENSG00000170950	ENSG00000053371	ENSG00000111300
ENSG00000249861	ENSG00000170296	ENSG00000196417
ENSG00000101266	ENSG00000184007	ENSG00000186440
ENSG00000077713	ENSG00000122378	ENSG00000170035
ENSG00000206106	ENSG00000180438	ENSG00000017621
ENSG00000173421	ENSG00000262660	ENSG00000188352
ENSG00000243244	ENSG00000242221	ENSG00000042429
ENSG00000050165	ENSG00000127084	ENSG00000119778
ENSG00000196409	ENSG00000122335	ENSG00000140992
ENSG00000142871	ENSG00000125449	ENSG00000243667
ENSG00000123594	ENSG00000257726	ENSG00000007306
ENSG00000233412	ENSG00000184611	ENSG00000183431
ENSG00000081019	ENSG00000175348	ENSG00000213347
ENSG00000176046	ENSG00000236499	ENSG00000145335
ENSG00000160199	ENSG00000100296	ENSG00000125821
ENSG00000100056	ENSG00000110619	ENSG00000109182
ENSG00000134873	ENSG00000096996	ENSG00000196943
ENSG00000134215	ENSG00000255561	ENSG00000124380
ENSG00000196363	ENSG00000117226	ENSG00000165409
ENSG00000188419	ENSG00000139323	ENSG00000257017
ENSG00000078124	ENSG00000177508	ENSG00000061656
ENSG00000138600	ENSG00000172208	ENSG00000156136
ENSG00000100557	ENSG00000225781	ENSG00000081386
ENSG00000143207	ENSG00000106025	ENSG00000140830
ENSG00000204604	ENSG00000158805	ENSG00000109381
ENSG00000167264	ENSG00000105374	ENSG00000126091
ENSG00000069345	ENSG00000118513	ENSG00000177752
ENSG00000141255	ENSG00000134461	ENSG00000138744
ENSG00000177301	ENSG00000160193	ENSG00000181577
ENSG00000091490	ENSG00000180138	ENSG00000119682
ENSG00000206052	ENSG00000107807	ENSG00000157500
ENSG00000063176	ENSG00000167778	ENSG00000173917
ENSG00000116212	ENSG00000109272	ENSG00000175334
ENSG00000135824	ENSG00000179636	ENSG00000117592
ENSG00000073150	ENSG00000082126	ENSG00000132623
ENSG00000134595	ENSG00000256053	ENSG00000213380
ENSG00000005059	ENSG00000235098	ENSG00000118094
ENSG00000214921	ENSG00000267179	ENSG00000198690
ENSG00000172594	ENSG00000130768	ENSG00000141425
ENSG00000134460	ENSG00000204393	ENSG00000163565
ENSG00000196284	ENSG00000115738	ENSG00000092841
ENSG00000137948	ENSG00000101084	ENSG00000174886
ENSG00000136883	ENSG00000105364	ENSG00000133104
ENSG00000164106	ENSG00000185962	ENSG00000213215
ENSG00000009724	ENSG00000214872	ENSG00000053524
ENSG00000092931	ENSG00000162599	ENSG00000091972
ENSG00000204704	ENSG00000205730	ENSG00000134216
ENSG00000169302	ENSG00000163209	ENSG00000124370
ENSG00000180543	ENSG00000102218	ENSG00000167230
ENSG00000255470	ENSG00000172201	ENSG00000124564
ENSG00000188266	ENSG00000168329	ENSG00000158639
ENSG00000152266	ENSG00000159792	ENSG00000056277
ENSG00000138386	ENSG00000185650	ENSG00000204671
ENSG00000174243	ENSG00000197405	ENSG00000163626
ENSG00000132522	ENSG00000124191	ENSG00000066427
ENSG00000215644	ENSG00000061987	ENSG00000172209
ENSG00000184863	ENSG00000136295	ENSG00000196189

ENSG00000164924	ENSG00000131495	ENSG00000056736
ENSG00000146085	ENSG00000197182	ENSG00000166326
ENSG00000149554	ENSG00000140403	ENSG00000182934
ENSG00000118507	ENSG00000262628	ENSG00000159398
ENSG00000048028	ENSG00000182393	ENSG00000135837
ENSG00000111266	ENSG00000173409	ENSG00000171163
ENSG00000168143	ENSG00000108406	ENSG00000056291
ENSG00000196944	ENSG00000164096	ENSG00000109083
ENSG00000092871	ENSG00000034971	ENSG00000144713
ENSG00000105926	ENSG00000083845	ENSG00000255833
ENSG00000151790	ENSG00000198601	ENSG00000033030
ENSG00000172733	ENSG00000087338	ENSG00000136003
ENSG00000137976	ENSG00000146192	ENSG00000147874
ENSG00000170381	ENSG00000105967	ENSG00000085998
ENSG00000135778	ENSG00000184260	ENSG00000126804
ENSG00000108679	ENSG00000100997	ENSG00000111729
ENSG00000171405	ENSG00000162543	ENSG00000079101
ENSG00000108700	ENSG00000197309	ENSG00000169208
ENSG00000244005	ENSG00000065717	ENSG00000213949
ENSG00000104140	ENSG00000198346	ENSG00000137075
ENSG00000180613	ENSG00000121377	ENSG00000022267
ENSG00000188878	ENSG00000178035	ENSG00000101391
ENSG00000157045	ENSG00000104907	ENSG00000162959
ENSG00000128482	ENSG00000087263	ENSG00000177535
ENSG00000124467	ENSG00000152292	ENSG00000150687
ENSG00000196711	ENSG00000139304	ENSG00000129007
ENSG00000167323	ENSG00000169740	ENSG00000149289
ENSG00000197498	ENSG00000148386	ENSG00000138032
ENSG00000101417	ENSG00000111305	ENSG00000100139
ENSG00000180667	ENSG00000078369	ENSG00000146350
ENSG00000204548	ENSG00000172059	ENSG00000166200
ENSG00000140105	ENSG00000172199	ENSG00000159256
ENSG00000179008	ENSG00000115761	ENSG00000163328
ENSG00000151725	ENSG00000152520	ENSG00000177494
ENSG00000132436	ENSG00000166780	ENSG00000147160
ENSG00000213719	ENSG00000125779	ENSG00000065413
ENSG00000130702	ENSG00000078579	ENSG00000123388
ENSG00000144744	ENSG00000105254	ENSG00000151612
ENSG00000188636	ENSG00000228368	ENSG00000161542
ENSG00000154040	ENSG00000137507	ENSG00000133398
ENSG00000198001	ENSG00000074219	ENSG00000140968
ENSG00000187097	ENSG00000114999	ENSG00000073861
ENSG00000117152	ENSG00000186652	ENSG00000087053
ENSG00000134153	ENSG00000180801	ENSG00000198000
ENSG00000167552	ENSG00000085832	ENSG00000165948
ENSG00000113161	ENSG00000076706	ENSG00000250254
ENSG00000162604	ENSG00000105223	ENSG00000133393
ENSG00000168890	ENSG00000170515	ENSG00000127947
ENSG00000204930	ENSG00000105656	ENSG00000046774
ENSG00000122068	ENSG00000204590	ENSG00000165325
ENSG00000169255	ENSG00000143753	ENSG00000264668
ENSG00000164494	ENSG00000169105	ENSG00000085978
ENSG00000148655	ENSG00000173214	ENSG00000144677
ENSG00000132855	ENSG00000259384	ENSG00000153187
ENSG00000127463	ENSG00000145808	ENSG00000178358
ENSG00000124657	ENSG00000072756	ENSG00000165416
ENSG00000140463	ENSG00000140691	ENSG00000166033
ENSG00000122417	ENSG00000158828	ENSG00000150456
ENSG00000134317	ENSG00000101361	ENSG00000174236
ENSG00000184500	ENSG00000107968	ENSG00000228768
ENSG00000161940	ENSG00000164645	ENSG00000203857
ENSG00000102098	ENSG00000204695	ENSG00000172164
ENSG00000081665	ENSG00000183474	ENSG00000112077
ENSG00000079387	ENSG00000143771	ENSG00000077942

ENSG00000100979	ENSG00000129187	ENSG00000141068
ENSG00000178385	ENSG00000174791	ENSG00000113525
ENSG00000095110	ENSG00000102879	ENSG00000170860
ENSG00000115942	ENSG00000154309	ENSG00000138376
ENSG00000130921	ENSG00000103023	ENSG00000152439
ENSG00000111647	ENSG00000151704	ENSG00000138606
ENSG00000006756	ENSG00000172269	ENSG00000184162
ENSG00000128283	ENSG00000160401	ENSG00000239382
ENSG00000197563	ENSG00000125037	ENSG00000204371
ENSG00000138400	ENSG00000165474	ENSG00000136937
ENSG00000115368	ENSG00000196934	ENSG00000172680
ENSG00000108654	ENSG00000180008	ENSG00000168538
ENSG00000115289	ENSG00000111445	ENSG00000077800
ENSG00000164111	ENSG00000198925	ENSG00000136842
ENSG00000164327	ENSG00000139549	ENSG00000174576
ENSG00000183281	ENSG00000154832	ENSG00000010219
ENSG00000130032	ENSG00000172830	ENSG00000083799
ENSG00000124224	ENSG00000137496	ENSG00000138231
ENSG00000144199	ENSG00000196865	ENSG00000140479
ENSG00000181004	ENSG00000131899	ENSG00000082175
ENSG00000120068	ENSG00000095981	ENSG00000148482
ENSG00000132740	ENSG00000132517	ENSG00000167280
ENSG00000036054	ENSG00000218891	ENSG00000067715
ENSG00000163608	ENSG00000267680	ENSG00000243696
ENSG00000169359	ENSG00000185519	ENSG00000129675
ENSG00000170606	ENSG00000136869	ENSG00000061455
ENSG00000160613	ENSG00000149084	ENSG00000197619
ENSG00000114127	ENSG00000135900	ENSG00000058866
ENSG00000100722	ENSG00000188817	ENSG00000152766
ENSG00000139496	ENSG00000151617	ENSG00000012983
ENSG00000141401	ENSG00000203747	ENSG00000163632
ENSG00000166788	ENSG00000214107	ENSG00000182264
ENSG00000163218	ENSG00000182378	ENSG00000169393
ENSG00000255508	ENSG00000100024	ENSG00000158055
ENSG00000185158	ENSG00000104231	ENSG00000151665
ENSG00000128000	ENSG00000159884	ENSG00000140718
ENSG00000106013	ENSG00000130701	ENSG00000187605
ENSG00000258804	ENSG00000230891	ENSG00000255223
ENSG00000143398	ENSG00000111834	ENSG00000170234
ENSG00000123552	ENSG00000101182	ENSG00000174156
ENSG00000112419	ENSG00000119929	ENSG00000181638
ENSG00000108666	ENSG00000125977	ENSG00000105875
ENSG00000197647	ENSG00000112599	ENSG00000230301
ENSG00000198816	ENSG00000068615	ENSG00000147852
ENSG00000137831	ENSG00000122877	ENSG00000118816
ENSG00000141002	ENSG00000188026	ENSG00000121207
ENSG00000043514	ENSG00000125954	ENSG00000126456
ENSG00000213931	ENSG00000188001	ENSG00000010072
ENSG00000173157	ENSG00000168275	ENSG00000126733
ENSG00000133997	ENSG00000197568	ENSG00000182521
ENSG00000166352	ENSG00000106263	ENSG00000183166
ENSG00000168811	ENSG00000136156	ENSG00000197757
ENSG00000170571	ENSG00000176160	ENSG00000064102
ENSG00000175193	ENSG00000189401	ENSG00000134326
ENSG00000115648	ENSG00000198363	ENSG00000173391
ENSG00000166548	ENSG00000188848	ENSG00000197415
ENSG00000049860	ENSG00000132405	ENSG00000130561
ENSG00000167850	ENSG00000205090	ENSG00000178252
ENSG00000183559	ENSG00000144771	ENSG00000135446
ENSG00000206043	ENSG00000206073	ENSG00000226887
ENSG00000182150	ENSG00000104435	ENSG00000152700
ENSG00000164220	ENSG00000103111	ENSG00000196821
ENSG00000143278	ENSG00000155016	ENSG00000196171
ENSG00000171987	ENSG00000137494	ENSG00000143373

ENSG00000164237	ENSG00000129151	ENSG00000250799
ENSG00000132356	ENSG00000137500	ENSG00000104872
ENSG00000184887	ENSG00000155380	ENSG00000176915
ENSG00000113924	ENSG00000105700	ENSG00000164520
ENSG00000117859	ENSG00000163586	ENSG00000136463
ENSG00000143369	ENSG00000158301	ENSG00000143954
ENSG00000184371	ENSG00000101335	ENSG00000165490
ENSG00000184164	ENSG00000138092	ENSG00000104824
ENSG00000169245	ENSG00000142186	ENSG00000171723
ENSG00000189283	ENSG00000186074	ENSG00000128923
ENSG00000115084	ENSG00000196141	ENSG00000204130
ENSG00000101019	ENSG00000170616	ENSG00000255168
ENSG00000096872	ENSG00000186184	ENSG00000226023
ENSG00000010017	ENSG00000196166	ENSG00000165138
ENSG00000143106	ENSG00000215203	ENSG00000136694
ENSG00000186522	ENSG00000184385	ENSG00000116273
ENSG00000185989	ENSG00000188888	ENSG00000169429
ENSG00000122043	ENSG00000131459	ENSG00000134285
ENSG00000160124	ENSG00000144134	ENSG00000171960
ENSG00000205409	ENSG00000198390	ENSG00000105810
ENSG00000162747	ENSG00000198324	ENSG00000183607
ENSG00000144395	ENSG00000143543	ENSG00000119457
ENSG00000183137	ENSG00000175121	ENSG00000115966
ENSG00000105185	ENSG00000197483	ENSG00000187969
ENSG00000183977	ENSG00000105379	ENSG00000173706
ENSG00000145839	ENSG00000137819	ENSG00000198742
ENSG00000189180	ENSG00000127824	ENSG00000100342
ENSG00000196172	ENSG00000177335	ENSG00000167747
ENSG00000185009	ENSG00000111058	ENSG00000117877
ENSG00000145283	ENSG00000152061	ENSG00000135093
ENSG00000172345	ENSG00000133800	ENSG00000174498
ENSG00000109738	ENSG00000155876	ENSG00000187151
ENSG00000155592	ENSG00000175182	ENSG00000116199
ENSG00000198648	ENSG00000182504	ENSG00000165816
ENSG00000213066	ENSG00000177575	ENSG00000123297
ENSG00000108094	ENSG00000198858	ENSG00000078401
ENSG00000028839	ENSG00000171150	ENSG00000066382
ENSG00000005175	ENSG00000244045	ENSG00000163349
ENSG00000186417	ENSG00000100842	ENSG00000181481
ENSG00000100612	ENSG00000186648	ENSG00000241595
ENSG00000163412	ENSG00000171443	ENSG00000188694
ENSG00000116191	ENSG00000099901	ENSG00000116031
ENSG00000158636	ENSG00000072274	ENSG00000110484
ENSG00000267106	ENSG00000155324	ENSG00000152137
ENSG00000149573	ENSG00000116698	ENSG00000102978
ENSG00000148671	ENSG00000134508	ENSG00000138293
ENSG00000153790	ENSG00000233224	ENSG00000150471
ENSG00000070731	ENSG00000257446	ENSG00000160194
ENSG00000120837	ENSG00000188452	ENSG00000167315
ENSG00000183476	ENSG00000116701	ENSG00000086065
ENSG00000164542	ENSG00000196345	ENSG00000122741
ENSG00000139645	ENSG00000048540	ENSG00000116514
ENSG00000099385	ENSG00000174951	ENSG00000173928
ENSG00000153037	ENSG00000074416	ENSG00000006007
ENSG00000125848	ENSG00000129277	ENSG00000151715
ENSG00000137185	ENSG00000164587	ENSG00000243566
ENSG00000171471	ENSG00000198755	ENSG00000069206
ENSG00000178172	ENSG00000152689	ENSG00000008394
ENSG00000266733	ENSG00000132702	ENSG00000103942
ENSG00000170627	ENSG00000090013	ENSG00000177125
ENSG00000139160	ENSG00000168010	ENSG00000005981
ENSG00000152936	ENSG00000137693	ENSG00000178188
ENSG00000196091	ENSG00000039600	ENSG00000165076
ENSG00000141380	ENSG00000168066	ENSG00000000003

ENSG00000156500	ENSG00000072954	ENSG00000196305
ENSG00000188010	ENSG00000069812	ENSG00000157077
ENSG00000126878	ENSG00000147889	ENSG00000137270
ENSG00000198874	ENSG00000029993	ENSG00000164325
ENSG00000081870	ENSG00000184564	ENSG00000187105
ENSG00000074201	ENSG00000171564	ENSG00000185250
ENSG00000188033	ENSG00000204659	ENSG00000143390
ENSG00000170365	ENSG00000167625	ENSG00000205496
ENSG00000236669	ENSG00000198739	ENSG00000100906
ENSG00000186907	ENSG00000171522	ENSG00000166024
ENSG00000163605	ENSG00000116991	ENSG00000164458
ENSG00000163785	ENSG00000118707	ENSG00000134882
ENSG00000204475	ENSG00000149212	ENSG00000198558
ENSG00000105141	ENSG00000136933	ENSG00000166747
ENSG00000078674	ENSG00000179914	ENSG00000132485
ENSG00000077458	ENSG00000187510	ENSG00000213240
ENSG00000155393	ENSG00000082512	ENSG00000178028
ENSG00000119729	ENSG00000131969	ENSG00000135547
ENSG00000170390	ENSG00000077157	ENSG00000197535
ENSG00000169826	ENSG00000163661	ENSG00000164631
ENSG00000170892	ENSG00000163424	ENSG00000070540
ENSG00000083750	ENSG00000102471	ENSG00000131233
ENSG00000169857	ENSG00000134490	ENSG00000110696
ENSG00000121073	ENSG00000100439	ENSG00000091651
ENSG00000256812	ENSG00000075073	ENSG00000134294
ENSG00000183530	ENSG00000143479	ENSG00000075089
ENSG00000009780	ENSG00000121381	ENSG00000141574
ENSG00000213516	ENSG00000182533	ENSG00000215301
ENSG00000113742	ENSG00000068781	ENSG00000124557
ENSG00000110944	ENSG00000129596	ENSG00000187918
ENSG00000268059	ENSG00000148688	ENSG00000168779
ENSG00000184560	ENSG00000092295	ENSG00000112530
ENSG00000185798	ENSG00000179058	ENSG00000186930
ENSG00000158092	ENSG00000198246	ENSG00000162927
ENSG00000149308	ENSG00000156219	ENSG00000078070
ENSG00000170266	ENSG00000133627	ENSG00000108506
ENSG00000069712	ENSG00000008294	ENSG00000162396
ENSG00000062485	ENSG00000187857	ENSG00000131471
ENSG00000106701	ENSG00000149582	ENSG00000170325
ENSG00000140488	ENSG00000100523	ENSG00000115274
ENSG00000197701	ENSG00000135973	ENSG00000173464
ENSG00000161849	ENSG00000076554	ENSG00000147853
ENSG00000154079	ENSG00000226397	ENSG00000196774
ENSG00000114744	ENSG00000162627	ENSG00000269259
ENSG00000204539	ENSG00000180483	ENSG00000117616
ENSG00000114790	ENSG00000111300	ENSG00000127920
ENSG00000168175	ENSG00000129514	ENSG00000079691
ENSG00000198894	ENSG00000196417	ENSG00000148337
ENSG00000163069	ENSG00000186440	ENSG00000136270
ENSG00000089775	ENSG00000170035	ENSG00000006744
ENSG00000178163	ENSG00000142273	ENSG00000172586
ENSG00000163440	ENSG00000167749	ENSG00000243729
ENSG00000186575	ENSG00000176749	ENSG00000242372
ENSG00000089505	ENSG00000017621	ENSG00000176087
ENSG00000144566	ENSG00000188352	ENSG00000129083
ENSG00000117289	ENSG00000042429	ENSG00000176302
ENSG00000165259	ENSG00000119778	ENSG00000167914
ENSG00000145416	ENSG00000140992	ENSG00000160345
ENSG00000186306	ENSG00000243667	ENSG00000180878
ENSG00000197372	ENSG00000007306	ENSG00000163191
ENSG00000170881	ENSG00000183431	ENSG00000149054
ENSG00000086544	ENSG00000213347	ENSG00000162888
ENSG00000091127	ENSG00000145335	ENSG00000172115
ENSG00000111554	ENSG00000125821	ENSG00000021852

ENSG00000163833	ENSG00000109182	ENSG00000196860
ENSG00000177414	ENSG00000196943	ENSG00000198556
ENSG00000165120	ENSG00000124380	ENSG00000163430
ENSG00000150656	ENSG00000165409	ENSG00000163145
ENSG00000163288	ENSG00000257017	ENSG00000197863
ENSG00000111727	ENSG00000061656	ENSG00000165684
ENSG00000196646	ENSG00000156136	ENSG00000179071
ENSG00000176658	ENSG00000081386	ENSG00000164411
ENSG00000100664	ENSG00000140830	ENSG00000180938
ENSG00000100526	ENSG00000109381	ENSG00000156097
ENSG00000169607	ENSG00000126091	ENSG00000128563
ENSG00000185658	ENSG00000177752	ENSG00000213967
ENSG00000117155	ENSG00000138744	ENSG00000009790
ENSG00000164654	ENSG00000181577	ENSG00000011198
ENSG00000115525	ENSG00000119682	ENSG00000204435
ENSG00000108448	ENSG00000157500	ENSG00000168038
ENSG00000082213	ENSG00000173917	ENSG00000189139
ENSG00000109606	ENSG00000175334	ENSG00000139193
ENSG00000100154	ENSG00000117592	ENSG00000185681
ENSG00000166527	ENSG00000129221	ENSG00000103888
ENSG00000099974	ENSG00000132623	ENSG00000173914
ENSG00000196331	ENSG00000213380	ENSG00000204256
ENSG00000048649	ENSG00000118094	ENSG00000110395
ENSG00000197937	ENSG00000198690	ENSG00000219073
ENSG00000225932	ENSG00000141425	ENSG00000176697
ENSG00000099937	ENSG00000163565	ENSG00000007038
ENSG00000139737	ENSG00000092841	ENSG00000180113
ENSG00000144559	ENSG00000174886	ENSG00000183826
ENSG00000137955	ENSG00000172725	ENSG00000170185
ENSG00000180660	ENSG00000133104	ENSG00000168994
ENSG00000149050	ENSG00000213215	ENSG00000104835
ENSG00000164062	ENSG00000053524	ENSG00000107105
ENSG00000047056	ENSG00000091972	ENSG00000014164
ENSG00000213918	ENSG00000134216	ENSG00000166153
ENSG00000003509	ENSG00000124370	ENSG00000176170
ENSG00000107290	ENSG00000167230	ENSG00000267508
ENSG00000074054	ENSG00000124564	ENSG00000138166
ENSG00000167232	ENSG00000158639	ENSG00000125931
ENSG00000114650	ENSG00000167895	ENSG00000174448
ENSG00000188770	ENSG00000056277	ENSG00000169490
ENSG00000182149	ENSG00000204671	ENSG00000131050
ENSG00000135269	ENSG00000163626	ENSG00000204700
ENSG00000198046	ENSG00000066427	ENSG00000217930
ENSG00000182263	ENSG00000172209	ENSG00000168062
ENSG00000205754	ENSG00000196189	ENSG00000104613
ENSG00000166157	ENSG00000056736	ENSG00000124116
ENSG00000269402	ENSG00000166326	ENSG00000178662
ENSG00000130741	ENSG00000182934	ENSG00000101871
ENSG00000205927	ENSG00000159398	ENSG00000198730
ENSG00000144228	ENSG00000135837	ENSG00000198826
ENSG00000211455	ENSG00000171163	ENSG00000172497
ENSG00000007168	ENSG00000056291	ENSG00000171853
ENSG00000166363	ENSG00000109083	ENSG00000073734
ENSG00000185278	ENSG00000136286	ENSG00000162408
ENSG00000164609	ENSG00000144713	ENSG00000174093
ENSG00000163781	ENSG00000255833	ENSG00000142676
ENSG00000165195	ENSG00000033030	ENSG00000197808
ENSG00000169814	ENSG00000108479	ENSG00000058799
ENSG00000211448	ENSG00000136003	ENSG00000166069
ENSG00000258664	ENSG00000147874	ENSG00000241553
ENSG00000136381	ENSG00000085998	ENSG00000184719
ENSG00000164736	ENSG00000126804	ENSG00000239665
ENSG00000188649	ENSG00000111729	ENSG00000203933
ENSG00000164808	ENSG00000175040	ENSG00000114473

ENSG00000186354	ENSG00000079101	ENSG00000000460
ENSG00000197429	ENSG00000169208	ENSG00000163156
ENSG00000163554	ENSG00000213949	ENSG00000255093
ENSG00000142892	ENSG00000137075	ENSG00000262633
ENSG00000184613	ENSG00000022267	ENSG00000196341
ENSG00000120915	ENSG00000101391	ENSG00000150630
ENSG00000186001	ENSG00000167671	ENSG00000215021
ENSG00000165923	ENSG00000162959	ENSG00000148429
ENSG00000149609	ENSG00000177535	ENSG00000115590
ENSG00000197024	ENSG00000150687	ENSG00000185721
ENSG00000168515	ENSG00000129007	ENSG00000140931
ENSG00000127951	ENSG00000204314	ENSG00000115884
ENSG00000141542	ENSG00000149289	ENSG00000161634
ENSG00000176956	ENSG00000138032	ENSG00000120708
ENSG00000186812	ENSG00000167703	ENSG00000167754
ENSG00000197511	ENSG00000072110	ENSG00000167858
ENSG00000172171	ENSG00000100139	ENSG00000156253
ENSG00000175395	ENSG00000151651	ENSG00000165495
ENSG00000137776	ENSG00000146350	ENSG00000115241
ENSG00000162976	ENSG00000162496	ENSG00000158528
ENSG00000015153	ENSG00000159871	ENSG00000250091
ENSG00000164294	ENSG00000166200	ENSG00000169224
ENSG00000214960	ENSG00000159256	ENSG00000106336
ENSG00000134769	ENSG00000163328	ENSG00000198053
ENSG00000165164	ENSG00000177494	ENSG00000049449
ENSG00000170236	ENSG00000147160	ENSG00000178999
ENSG00000205838	ENSG00000065413	ENSG00000166526
ENSG00000123096	ENSG00000123388	ENSG00000092445
ENSG00000187987	ENSG00000151612	ENSG00000183696
ENSG00000174720	ENSG00000161542	ENSG00000177673
ENSG00000106049	ENSG00000133398	ENSG00000113658
ENSG00000152990	ENSG00000140968	ENSG00000123415
ENSG00000164815	ENSG00000073861	ENSG00000161298
ENSG00000100811	ENSG00000087053	ENSG00000150712
ENSG00000159445	ENSG00000198000	ENSG00000146729
ENSG00000135960	ENSG00000183741	ENSG00000118600
ENSG00000164484	ENSG00000165948	ENSG00000109133
ENSG00000103194	ENSG00000250254	ENSG00000162365
ENSG00000161013	ENSG00000133393	ENSG00000101190
ENSG00000162669	ENSG00000254806	ENSG00000187990
ENSG00000132952	ENSG00000127947	ENSG00000186073
ENSG00000204228	ENSG00000046774	ENSG00000132780
ENSG00000151743	ENSG00000165325	ENSG00000149929
ENSG00000164169	ENSG00000264668	ENSG00000143140
ENSG00000104974	ENSG00000161996	ENSG00000118156
ENSG00000049759	ENSG00000085978	ENSG00000106829
ENSG00000178965	ENSG00000144677	ENSG00000105497
ENSG00000131174	ENSG00000153187	ENSG00000140254
ENSG00000198130	ENSG00000178358	ENSG00000248487
ENSG00000105341	ENSG00000165416	ENSG00000141664
ENSG00000196437	ENSG00000166033	ENSG00000197375
ENSG00000126883	ENSG00000126603	ENSG00000157800
ENSG00000143384	ENSG00000150456	ENSG00000204843
ENSG00000088179	ENSG00000174236	ENSG00000159461
ENSG00000173418	ENSG00000228768	ENSG00000080561
ENSG00000167740	ENSG00000244115	ENSG00000176390
ENSG00000128342	ENSG00000203857	ENSG00000137033
ENSG00000181718	ENSG00000166148	ENSG00000086696
ENSG00000173068	ENSG00000172164	ENSG00000254959
ENSG00000111846	ENSG00000112077	ENSG00000205864
ENSG00000102753	ENSG00000077942	ENSG00000112742
ENSG00000087448	ENSG00000186765	ENSG00000127837
ENSG00000120278	ENSG00000141068	ENSG00000103852
ENSG00000176125	ENSG00000113525	ENSG00000205649

ENSG00000006530	ENSG00000170860	ENSG00000197054
ENSG00000269307	ENSG00000138376	ENSG00000127884
ENSG00000006042	ENSG00000152439	ENSG00000196247
ENSG00000235194	ENSG00000138606	ENSG00000146858
ENSG00000198542	ENSG00000160256	ENSG00000226232
ENSG00000134028	ENSG00000184162	ENSG00000156110
ENSG00000183751	ENSG00000239382	ENSG00000160392
ENSG00000111275	ENSG00000215440	ENSG00000066136
ENSG00000129636	ENSG00000204371	ENSG00000151422
ENSG00000037897	ENSG00000136937	ENSG00000168685
ENSG00000162545	ENSG00000172680	ENSG00000124160
ENSG00000167604	ENSG00000168538	ENSG00000249087
ENSG00000229972	ENSG00000077800	ENSG00000186416
ENSG00000111450	ENSG00000136842	ENSG00000100593
ENSG00000179253	ENSG00000174576	ENSG00000172197
ENSG00000135404	ENSG00000010219	ENSG00000157259
ENSG00000153233	ENSG00000083799	ENSG00000180066
ENSG00000101350	ENSG00000138231	ENSG00000137504
ENSG00000182584	ENSG00000140479	ENSG00000074935
ENSG00000135046	ENSG00000082175	ENSG00000160563
ENSG00000111196	ENSG00000148482	ENSG00000010256
ENSG00000126247	ENSG00000167280	ENSG00000197125
ENSG00000171174	ENSG00000171303	ENSG00000203785
ENSG00000060339	ENSG00000067715	ENSG00000112081
ENSG00000221910	ENSG00000243696	ENSG00000157343
ENSG00000163002	ENSG00000129675	ENSG00000151576
ENSG00000159377	ENSG00000061455	ENSG00000181935
ENSG00000131781	ENSG00000197619	ENSG00000110237
ENSG00000164074	ENSG00000058866	ENSG00000214194
ENSG00000182791	ENSG00000152766	ENSG00000175711
ENSG00000126214	ENSG00000012983	ENSG00000158764
ENSG00000134371	ENSG00000119715	ENSG00000175592
ENSG00000204936	ENSG00000163632	ENSG00000197233
ENSG00000152683	ENSG00000182264	ENSG00000164134
ENSG00000115993	ENSG00000169393	ENSG00000073792
ENSG00000127922	ENSG00000158055	ENSG00000109762
ENSG00000172650	ENSG00000151665	ENSG00000165480
ENSG00000074706	ENSG00000140718	ENSG00000165943
ENSG00000188467	ENSG00000187605	ENSG00000114796
ENSG00000011454	ENSG00000255223	ENSG00000113597
ENSG00000108592	ENSG00000170234	ENSG00000149970
ENSG00000254004	ENSG00000174156	ENSG00000136689
ENSG00000082068	ENSG00000242173	ENSG00000159289
ENSG00000109814	ENSG00000181638	ENSG00000143224
ENSG00000168906	ENSG00000105875	ENSG00000175874
ENSG00000188674	ENSG00000230301	ENSG00000039523
ENSG00000184459	ENSG00000147852	ENSG00000183723
ENSG00000196109	ENSG00000118816	ENSG00000187753
ENSG00000074582	ENSG00000121207	ENSG00000087494
ENSG00000230344	ENSG00000126456	ENSG00000117114
ENSG00000102539	ENSG00000010072	ENSG00000177938
ENSG00000147439	ENSG00000126733	ENSG00000137200
ENSG00000120832	ENSG00000182521	ENSG00000100427
ENSG00000203926	ENSG00000183166	ENSG00000161692
ENSG00000113638	ENSG00000108511	ENSG00000188517
ENSG00000153179	ENSG00000197757	ENSG00000213928
ENSG00000188428	ENSG00000064102	ENSG00000214556
ENSG00000205030	ENSG00000134326	ENSG00000139971
ENSG00000169385	ENSG00000188909	ENSG00000157224
ENSG00000168032	ENSG00000173391	ENSG00000134086
ENSG00000105866	ENSG00000197415	ENSG00000137440
ENSG00000060138	ENSG00000130561	ENSG00000111885
ENSG00000115109	ENSG00000178252	ENSG00000141580
ENSG00000122882	ENSG00000135446	ENSG00000132142

ENSG00000138433	ENSG00000226887	ENSG00000188152
ENSG00000140682	ENSG00000152700	ENSG00000102743
ENSG00000139219	ENSG00000196821	ENSG00000067533
ENSG00000161960	ENSG00000196171	ENSG00000158488
ENSG00000100567	ENSG00000143373	ENSG00000256632
ENSG00000151304	ENSG00000250799	ENSG00000126602
ENSG00000144909	ENSG00000104872	ENSG00000185499
ENSG00000196715	ENSG00000176915	ENSG00000171989
ENSG00000121310	ENSG00000164520	ENSG00000130684
ENSG00000197728	ENSG00000136463	ENSG00000169031
ENSG00000146574	ENSG00000166558	ENSG00000197019
ENSG00000170613	ENSG00000143954	ENSG00000134242
ENSG00000146856	ENSG00000165490	ENSG00000188647
ENSG00000169891	ENSG00000104824	ENSG00000115750
ENSG00000080572	ENSG00000171723	ENSG00000244025
ENSG00000078795	ENSG00000128923	ENSG00000104325
ENSG00000170293	ENSG00000204130	ENSG00000175538
ENSG00000112818	ENSG00000255168	ENSG00000198353
ENSG00000175449	ENSG00000226023	ENSG00000029725
ENSG00000128708	ENSG00000165138	ENSG00000038274
ENSG00000121964	ENSG00000125522	ENSG00000178096
ENSG00000197321	ENSG00000136694	ENSG00000111880
ENSG00000131051	ENSG00000116273	ENSG00000054983
ENSG00000165304	ENSG00000169429	ENSG00000251537
ENSG00000162928	ENSG00000134285	ENSG00000183831
ENSG00000054267	ENSG00000171960	ENSG00000263002
ENSG00000173559	ENSG00000105810	ENSG00000143556
ENSG00000120158	ENSG00000183607	ENSG00000186496
ENSG00000011258	ENSG00000125735	ENSG00000140396
ENSG00000156384	ENSG00000119457	ENSG00000213973
ENSG00000138801	ENSG00000148985	ENSG00000180098
ENSG00000213578	ENSG00000115966	ENSG00000132768
ENSG00000166068	ENSG00000187969	ENSG00000108039
ENSG00000128607	ENSG00000173706	ENSG00000255641
ENSG00000132016	ENSG00000198742	ENSG00000109680
ENSG00000187833	ENSG00000100342	ENSG00000019102
ENSG00000155111	ENSG00000197457	ENSG00000006194
ENSG00000082212	ENSG00000205336	ENSG00000185087
ENSG00000116906	ENSG00000129354	ENSG00000111490
ENSG00000104450	ENSG00000167747	ENSG00000109832
ENSG00000162994	ENSG00000117877	ENSG00000118194
ENSG00000163393	ENSG00000135093	ENSG00000120820
ENSG00000128272	ENSG00000174498	ENSG00000100271
ENSG00000167705	ENSG00000167113	ENSG00000003436
ENSG00000244067	ENSG00000187151	ENSG00000139372
ENSG00000131127	ENSG00000186897	ENSG00000089163
ENSG00000171533	ENSG00000116199	ENSG00000153029
ENSG00000074603	ENSG00000165816	ENSG00000149485
ENSG00000137960	ENSG00000123297	ENSG00000076201
ENSG00000170899	ENSG00000078401	ENSG00000101413
ENSG00000165828	ENSG00000066382	ENSG00000132693
ENSG00000121104	ENSG00000007516	ENSG00000162433
ENSG00000136891	ENSG00000163349	ENSG00000258447
ENSG00000196584	ENSG00000181481	ENSG00000174456
ENSG00000053900	ENSG00000241595	ENSG00000165591
ENSG00000005893	ENSG00000188694	ENSG00000116750
ENSG00000108946	ENSG00000116031	ENSG00000127483
ENSG00000089818	ENSG00000110484	ENSG00000189159
ENSG00000119004	ENSG00000152137	ENSG00000232382
ENSG00000155307	ENSG00000102978	ENSG00000188368
ENSG00000168658	ENSG00000138293	ENSG00000171659
ENSG00000177042	ENSG00000150471	ENSG00000128039
ENSG00000196946	ENSG00000160194	ENSG00000169519
ENSG00000120685	ENSG00000167315	ENSG00000196670

ENSG00000171492	ENSG00000086065	ENSG00000177138
ENSG00000143036	ENSG00000122741	ENSG00000100312
ENSG00000167359	ENSG00000116514	ENSG00000119689
ENSG00000120798	ENSG00000173928	ENSG00000163751
ENSG00000203908	ENSG00000006007	ENSG00000158793
ENSG00000140471	ENSG00000151715	ENSG00000106331
ENSG00000064995	ENSG00000243566	ENSG00000165584
ENSG00000110080	ENSG00000069206	ENSG00000105732
ENSG00000169758	ENSG00000008394	ENSG00000168785
ENSG00000079931	ENSG00000188290	ENSG00000179133
ENSG00000248801	ENSG00000103942	ENSG00000125459
ENSG00000198019	ENSG00000177125	ENSG00000186143
ENSG00000166225	ENSG00000005981	ENSG00000230031
ENSG00000132680	ENSG00000178188	ENSG00000103528
ENSG00000196118	ENSG00000165076	ENSG00000126267
ENSG00000156265	ENSG00000000003	ENSG00000080802
ENSG00000109181	ENSG00000196305	ENSG00000103351
ENSG00000113593	ENSG00000157077	ENSG00000144354
ENSG00000135473	ENSG00000137270	ENSG00000164683
ENSG00000158019	ENSG00000164325	ENSG00000134709
ENSG00000157106	ENSG00000187105	ENSG00000103047
ENSG00000182117	ENSG00000185250	ENSG00000109193
ENSG00000205330	ENSG00000143390	ENSG00000119574
ENSG00000162642	ENSG00000205496	ENSG00000124702
ENSG00000078747	ENSG00000178695	ENSG00000099260
ENSG00000166900	ENSG00000100906	ENSG00000175573
ENSG00000105771	ENSG00000186188	ENSG00000165061
ENSG00000109458	ENSG00000166024	ENSG00000166262
ENSG00000169375	ENSG00000164458	ENSG00000123427
ENSG00000173209	ENSG00000134882	ENSG00000104142
ENSG00000104879	ENSG00000198558	ENSG00000132958
ENSG00000100320	ENSG00000166747	ENSG00000138336
ENSG00000157551	ENSG00000132485	ENSG00000122642
ENSG00000100380	ENSG00000213240	ENSG00000143839
ENSG00000138709	ENSG00000178028	ENSG00000187010
ENSG00000084463	ENSG00000135547	ENSG00000130307
ENSG00000185670	ENSG00000197535	ENSG00000113615
ENSG00000140948	ENSG00000164631	ENSG00000240386
ENSG00000268528	ENSG00000070540	ENSG00000182508
ENSG00000198610	ENSG00000131233	ENSG00000144021
ENSG00000179919	ENSG00000101665	ENSG00000091409
ENSG00000126860	ENSG00000179348	ENSG00000182253
ENSG00000183665	ENSG00000110696	ENSG00000115194
ENSG00000134575	ENSG00000189337	ENSG00000125970
ENSG00000133739	ENSG00000091651	ENSG00000075826
ENSG00000102384	ENSG00000134294	ENSG00000156574
ENSG00000203867	ENSG00000075089	ENSG00000110218
ENSG00000170445	ENSG00000141574	ENSG00000157093
ENSG00000241697	ENSG00000134830	ENSG00000233816
ENSG00000104112	ENSG00000215301	ENSG00000164902
ENSG00000165629	ENSG00000124557	ENSG00000103064
ENSG00000142875	ENSG00000198915	ENSG00000204814
ENSG00000175697	ENSG00000187918	ENSG00000221882
ENSG00000197472	ENSG00000168779	ENSG00000269690
ENSG00000043093	ENSG00000139567	ENSG00000108953
ENSG00000135925	ENSG00000112530	ENSG00000180228
ENSG00000101966	ENSG00000167962	ENSG00000221932
ENSG00000151458	ENSG00000186930	ENSG00000164303
ENSG00000265817	ENSG00000162927	ENSG00000166801
ENSG00000176155	ENSG00000078070	ENSG00000102401
ENSG00000185305	ENSG00000108506	ENSG00000187079
ENSG00000180974	ENSG00000162396	ENSG00000019144
ENSG00000144747	ENSG00000148291	ENSG00000175970
ENSG00000185862	ENSG00000131471	ENSG00000166343

ENSG00000121152	ENSG00000170325	ENSG00000128694
ENSG00000006451	ENSG00000167302	ENSG00000165458
ENSG00000221954	ENSG00000115274	ENSG00000119711
ENSG00000130545	ENSG00000173464	ENSG00000152056
ENSG00000100461	ENSG00000147853	ENSG00000007392
ENSG00000077232	ENSG00000196774	ENSG00000095209
ENSG00000109270	ENSG00000269259	ENSG00000113460
ENSG00000181690	ENSG00000117616	ENSG00000147596
ENSG00000160214	ENSG00000127920	ENSG00000166569
ENSG00000197894	ENSG00000079691	ENSG00000006831
ENSG00000154511	ENSG00000148337	ENSG00000162222
ENSG00000106546	ENSG00000136270	ENSG00000133422
ENSG00000152749	ENSG00000006744	ENSG00000181026
ENSG00000145569	ENSG00000172586	ENSG00000123496
ENSG00000253598	ENSG00000103485	ENSG00000137101
ENSG00000071889	ENSG00000243729	ENSG00000160201
ENSG00000143977	ENSG00000242372	ENSG00000164949
ENSG00000158623	ENSG00000176087	ENSG00000142494
ENSG00000171552	ENSG00000129083	ENSG00000078061
ENSG00000196862	ENSG00000176302	ENSG00000163820
ENSG00000141232	ENSG00000167914	ENSG00000075790
ENSG00000164332	ENSG00000160345	ENSG00000015475
ENSG00000187735	ENSG00000234224	ENSG00000119638
ENSG00000170275	ENSG00000188897	ENSG00000138813
ENSG00000198825	ENSG00000180878	ENSG00000089248
ENSG00000205011	ENSG00000163191	ENSG00000198398
ENSG00000172273	ENSG00000149054	ENSG00000153914
ENSG00000198182	ENSG00000168505	ENSG00000204304
ENSG00000124529	ENSG00000162888	ENSG00000186960
ENSG00000083838	ENSG00000172115	ENSG00000108798
ENSG00000237515	ENSG00000021852	ENSG00000182568
ENSG00000040275	ENSG00000196860	ENSG00000221867
ENSG00000136709	ENSG00000198556	ENSG00000204516
ENSG00000170385	ENSG00000182512	ENSG00000204116
ENSG00000141741	ENSG00000163430	ENSG00000172377
ENSG00000163468	ENSG00000160472	ENSG00000257594
ENSG00000172795	ENSG00000163145	ENSG00000166670
ENSG00000115392	ENSG00000197863	ENSG00000186767
ENSG00000127081	ENSG00000165684	ENSG00000173473
ENSG00000204366	ENSG00000179071	ENSG00000196792
ENSG00000095596	ENSG00000164411	ENSG00000138688
ENSG00000007001	ENSG00000081277	ENSG00000185515
ENSG00000092010	ENSG00000100721	ENSG00000119397
ENSG00000100815	ENSG00000180938	ENSG00000183662
ENSG00000099940	ENSG00000139194	ENSG00000156469
ENSG00000116044	ENSG00000171596	ENSG00000105186
ENSG00000048471	ENSG00000170421	ENSG00000146263
ENSG00000213903	ENSG00000156097	ENSG00000062524
ENSG00000212722	ENSG00000128563	ENSG00000198853
ENSG00000107130	ENSG00000213967	ENSG00000177426
ENSG00000170734	ENSG00000009790	ENSG00000181827
ENSG00000187790	ENSG00000241123	ENSG00000103148
ENSG00000092094	ENSG00000011198	ENSG00000115540
ENSG00000148218	ENSG00000204435	ENSG00000164405
ENSG00000167881	ENSG00000135480	ENSG00000162757
ENSG00000105393	ENSG00000110446	ENSG00000064199
ENSG00000170099	ENSG00000168038	ENSG00000063180
ENSG00000203963	ENSG00000104884	ENSG00000003402
ENSG00000100281	ENSG00000189139	ENSG00000163590
ENSG00000165895	ENSG00000162595	ENSG00000255374
ENSG00000096401	ENSG00000139193	ENSG00000172016
ENSG00000135316	ENSG00000215475	ENSG00000169271
ENSG00000115457	ENSG00000185681	ENSG00000111602
ENSG00000136935	ENSG00000099875	ENSG00000100376

ENSG00000177181	ENSG00000103888	ENSG00000105865
ENSG00000196391	ENSG00000173914	ENSG00000174970
ENSG00000171497	ENSG00000163931	ENSG00000087884
ENSG00000249773	ENSG00000204256	ENSG00000138443
ENSG00000165171	ENSG00000110395	ENSG00000173769
ENSG00000115947	ENSG00000219073	ENSG00000124151
ENSG00000131375	ENSG00000090554	ENSG00000127329
ENSG00000221994	ENSG00000176697	ENSG00000100450
ENSG00000102245	ENSG00000007038	ENSG00000062282
ENSG00000090263	ENSG00000180113	ENSG00000006468
ENSG00000138041	ENSG00000183826	ENSG00000100479
ENSG00000154957	ENSG00000170185	ENSG00000117477
ENSG00000164236	ENSG00000168994	ENSG00000120029
ENSG00000215915	ENSG00000104835	ENSG00000226479
ENSG00000254466	ENSG00000107105	ENSG00000153922
ENSG00000075420	ENSG00000014164	ENSG00000114405
ENSG00000122008	ENSG00000166153	ENSG00000137628
ENSG00000101557	ENSG00000176170	ENSG00000184661
ENSG00000174197	ENSG00000141965	ENSG00000213822
ENSG00000112238	ENSG00000267508	ENSG00000166211
ENSG00000135723	ENSG00000183396	ENSG00000241127
ENSG00000077454	ENSG00000138166	ENSG00000152582
ENSG00000117139	ENSG00000125931	ENSG00000155846
ENSG00000181392	ENSG00000174448	ENSG00000168274
ENSG00000115091	ENSG00000069399	ENSG00000110721
ENSG00000107758	ENSG00000169490	ENSG00000080709
ENSG00000120690	ENSG00000131050	ENSG00000165512
ENSG00000172318	ENSG00000204700	ENSG00000138109
ENSG00000237787	ENSG00000217930	ENSG00000043462
ENSG00000051596	ENSG00000168062	ENSG00000090376
ENSG00000189241	ENSG00000104613	ENSG00000231213
ENSG00000182511	ENSG00000124116	ENSG00000120242
ENSG00000135541	ENSG00000143793	ENSG00000197416
ENSG00000172172	ENSG00000104903	ENSG00000180592
ENSG00000100218	ENSG00000071246	ENSG00000173213
ENSG00000163870	ENSG00000178662	ENSG00000214367
ENSG00000132604	ENSG00000149418	ENSG00000156050
ENSG00000162676	ENSG00000101871	ENSG00000006114
ENSG00000149483	ENSG00000198730	ENSG00000146109
ENSG00000183617	ENSG00000136942	ENSG00000215570
ENSG00000175220	ENSG00000198826	ENSG00000138944
ENSG00000168283	ENSG00000172497	ENSG00000144791
ENSG00000204165	ENSG00000171853	ENSG00000183060
ENSG00000178934	ENSG00000073734	ENSG00000117595
ENSG00000162621	ENSG00000162408	ENSG00000259399
ENSG00000131652	ENSG00000174093	ENSG00000176142
ENSG00000107185	ENSG00000142676	ENSG00000116785
ENSG00000196652	ENSG00000072958	ENSG00000236320
ENSG00000008283	ENSG00000197808	ENSG00000145012
ENSG00000108468	ENSG00000058799	ENSG00000115112
ENSG00000164114	ENSG00000166069	ENSG00000187238
ENSG00000169313	ENSG00000241553	ENSG00000114378
ENSG00000184220	ENSG00000184719	ENSG00000107669
ENSG00000105829	ENSG00000239665	ENSG00000176386
ENSG00000244537	ENSG00000203933	ENSG00000101773
ENSG00000169026	ENSG00000114473	ENSG00000221978
ENSG00000131238	ENSG00000000460	ENSG00000112299
ENSG00000178804	ENSG00000124635	ENSG00000188257
ENSG00000156049	ENSG00000015532	ENSG00000258405
ENSG00000099330	ENSG00000163156	ENSG00000135677
ENSG00000101146	ENSG00000255093	ENSG00000151332
ENSG00000131153	ENSG00000262633	ENSG00000145386
ENSG00000121671	ENSG00000196341	ENSG00000066813
ENSG00000100577	ENSG00000150630	ENSG00000196374

ENSG00000177045	ENSG00000215021	ENSG00000170873
ENSG00000165905	ENSG00000148429	ENSG00000150540
ENSG00000181938	ENSG00000115590	ENSG00000138768
ENSG00000186577	ENSG00000185721	ENSG00000198707
ENSG00000154222	ENSG00000105355	ENSG00000172785
ENSG00000171824	ENSG00000140931	ENSG00000197471
ENSG00000186281	ENSG00000115884	ENSG00000024048
ENSG00000051341	ENSG00000130829	ENSG00000204231
ENSG00000106714	ENSG00000161634	ENSG00000148481
ENSG00000088766	ENSG00000120708	ENSG00000203970
ENSG00000214226	ENSG00000088899	ENSG00000146830
ENSG00000070669	ENSG00000167754	ENSG00000069667
ENSG00000164483	ENSG00000103335	ENSG00000113558
ENSG00000077312	ENSG00000167858	ENSG00000143536
ENSG00000261857	ENSG00000156253	ENSG00000152253
ENSG00000119669	ENSG00000165495	ENSG00000168411
ENSG00000260527	ENSG00000115241	ENSG00000145191
ENSG00000242515	ENSG00000084207	ENSG00000167182
ENSG00000101336	ENSG00000158528	ENSG00000172238
ENSG00000129559	ENSG00000250091	ENSG00000119943
ENSG00000175518	ENSG00000161036	ENSG00000189037
ENSG00000006715	ENSG00000169224	ENSG00000064726
ENSG00000041353	ENSG00000106336	ENSG00000081059
ENSG00000185264	ENSG00000130731	ENSG00000138413
ENSG00000176219	ENSG00000198053	ENSG00000177483
ENSG00000143545	ENSG00000049449	ENSG00000116106
ENSG00000092470	ENSG00000178999	ENSG00000166035
ENSG00000100814	ENSG00000166526	ENSG00000154217
ENSG00000182118	ENSG00000092445	ENSG00000136932
ENSG00000139168	ENSG00000183696	ENSG00000177951
ENSG00000065054	ENSG00000177673	ENSG00000168389
ENSG00000164733	ENSG00000113658	ENSG00000167766
ENSG00000124164	ENSG00000214309	ENSG00000163644
ENSG00000198887	ENSG00000123415	ENSG00000137274
ENSG00000166974	ENSG00000173457	ENSG00000078549
ENSG00000184787	ENSG00000197774	ENSG00000007908
ENSG00000162704	ENSG00000161298	ENSG00000139714
ENSG00000180694	ENSG00000150712	ENSG00000146247
ENSG00000115474	ENSG00000146729	ENSG00000102678
ENSG00000171396	ENSG00000161671	ENSG00000187147
ENSG00000136982	ENSG00000169093	ENSG00000168077
ENSG00000145919	ENSG00000231852	ENSG00000139631
ENSG00000163125	ENSG00000118600	ENSG00000198967
ENSG00000065613	ENSG00000109133	ENSG00000167759
ENSG00000178665	ENSG00000162365	ENSG00000186566
ENSG00000014914	ENSG00000101190	ENSG00000138073
ENSG00000146223	ENSG00000187990	ENSG00000126464
ENSG00000076321	ENSG00000186073	ENSG00000175213
ENSG00000128713	ENSG00000132780	ENSG00000111011
ENSG00000152078	ENSG00000149929	ENSG00000204572
ENSG00000108771	ENSG00000116685	ENSG00000158985
ENSG00000141569	ENSG00000152669	ENSG00000136895
ENSG00000198380	ENSG00000143140	ENSG00000086666
ENSG00000115762	ENSG00000118156	ENSG00000180346
ENSG00000249709	ENSG00000179364	ENSG00000144407
ENSG00000151876	ENSG00000106829	ENSG00000171936
ENSG00000176716	ENSG00000172478	ENSG00000196979
ENSG00000136098	ENSG00000258674	ENSG00000184492
ENSG00000116957	ENSG00000267149	ENSG00000102030
ENSG00000148426	ENSG00000105497	ENSG00000172955
ENSG00000121851	ENSG00000140254	ENSG00000168765
ENSG00000115944	ENSG00000248487	ENSG00000125998
ENSG00000164896	ENSG00000181085	ENSG00000118496
ENSG00000158022	ENSG00000141664	ENSG00000181733

ENSG00000139613	ENSG00000187091	ENSG00000168743
ENSG00000100425	ENSG00000197375	ENSG00000186603
ENSG00000092421	ENSG00000125492	ENSG00000164904
ENSG00000187266	ENSG00000157800	ENSG00000123560
ENSG00000158864	ENSG00000204843	ENSG00000173065
ENSG00000168268	ENSG00000159461	ENSG00000134686
ENSG00000063761	ENSG00000080189	ENSG00000167004
ENSG00000093134	ENSG00000080561	ENSG00000178821
ENSG00000226174	ENSG00000110057	ENSG00000047932
ENSG00000236624	ENSG00000176390	ENSG00000181433
ENSG00000231925	ENSG00000137033	ENSG00000187720
ENSG00000133740	ENSG00000086696	ENSG00000072736
ENSG00000188511	ENSG00000254959	ENSG00000174851
ENSG00000165655	ENSG00000205864	ENSG00000143469
ENSG00000121716	ENSG00000101331	ENSG00000175792
ENSG00000125875	ENSG00000112742	ENSG00000163697
ENSG00000148795	ENSG00000127837	ENSG00000104361
ENSG00000183146	ENSG00000103852	ENSG00000132010
ENSG00000168828	ENSG00000156711	ENSG00000138663
ENSG00000181781	ENSG00000115255	ENSG00000160908
ENSG00000164932	ENSG00000205649	ENSG00000122783
ENSG00000177186	ENSG00000197054	ENSG00000011295
ENSG00000124356	ENSG00000127884	ENSG00000123360
ENSG00000206531	ENSG00000196247	ENSG00000172156
ENSG00000166321	ENSG00000146858	ENSG00000154611
ENSG00000255103	ENSG00000226232	ENSG00000107874
ENSG00000137880	ENSG00000156110	ENSG00000163141
ENSG00000103855	ENSG00000103199	ENSG00000174599
ENSG00000198498	ENSG00000160392	ENSG00000151577
ENSG00000198075	ENSG00000066136	ENSG00000120756
ENSG00000083099	ENSG00000151422	ENSG00000196772
ENSG00000172732	ENSG00000115844	ENSG00000106415
ENSG00000091140	ENSG00000160216	ENSG00000099953
ENSG00000096088	ENSG00000168685	ENSG00000101888
ENSG00000127743	ENSG00000124160	ENSG00000198104
ENSG00000116120	ENSG00000249087	ENSG00000206104
ENSG00000168772	ENSG00000124839	ENSG00000135374
ENSG00000172292	ENSG00000168081	ENSG00000099364
ENSG00000179817	ENSG00000186416	ENSG00000099250
ENSG00000138115	ENSG00000100593	ENSG00000155090
ENSG00000145040	ENSG00000162241	ENSG00000110756
ENSG00000107262	ENSG00000261459	ENSG00000220201
ENSG00000105205	ENSG00000121281	ENSG00000114520
ENSG00000015676	ENSG00000105552	ENSG00000185651
ENSG00000214415	ENSG00000182218	ENSG00000176714
ENSG00000164296	ENSG00000172197	ENSG00000180210
ENSG00000152219	ENSG00000157259	ENSG00000155330
ENSG00000185272	ENSG00000169885	ENSG00000065809
ENSG00000088782	ENSG00000180066	ENSG00000196628
ENSG00000180011	ENSG00000137504	ENSG00000131591
ENSG00000163882	ENSG00000188784	ENSG00000073737
ENSG00000143315	ENSG00000074935	ENSG00000170144
ENSG00000167077	ENSG00000160563	ENSG00000120725
ENSG00000198860	ENSG00000010256	ENSG00000072415
ENSG00000101883	ENSG00000197125	ENSG00000139977
ENSG00000178057	ENSG00000203785	ENSG00000071909
ENSG00000115524	ENSG00000112081	ENSG00000124789
ENSG00000180901	ENSG00000157343	ENSG00000175287
ENSG00000115129	ENSG00000151576	ENSG00000105556
ENSG00000258790	ENSG00000181935	ENSG00000174125
ENSG00000249242	ENSG00000110237	ENSG00000127324
ENSG00000184857	ENSG00000214194	ENSG00000234857
ENSG00000138207	ENSG00000175711	ENSG00000116171
ENSG00000151881	ENSG00000158764	ENSG00000198643

ENSG00000205356	ENSG00000175592	ENSG00000119737
ENSG00000205089	ENSG00000197233	ENSG00000160803
ENSG00000150527	ENSG00000164134	ENSG00000141579
ENSG00000122696	ENSG00000073792	ENSG00000123843
ENSG00000105298	ENSG00000109762	ENSG00000164081
ENSG00000002586	ENSG00000165480	ENSG00000110925
ENSG00000220008	ENSG00000165943	ENSG00000166987
ENSG00000259332	ENSG00000114796	ENSG00000203879
ENSG00000186446	ENSG00000113597	ENSG00000230797
ENSG00000007944	ENSG00000149970	ENSG00000176231
ENSG00000164972	ENSG00000136689	ENSG00000187258
ENSG00000077092	ENSG00000159289	ENSG00000234719
ENSG00000205277	ENSG00000143224	ENSG00000158402
ENSG00000178828	ENSG00000175874	ENSG00000137672
ENSG00000214819	ENSG00000039523	ENSG00000184743
ENSG00000228278	ENSG00000183723	ENSG00000186453
ENSG00000198563	ENSG00000187753	ENSG00000144671
ENSG00000162458	ENSG00000130813	ENSG00000110768
ENSG00000205318	ENSG00000087494	ENSG00000066027
ENSG00000173113	ENSG00000168421	ENSG00000177300
ENSG00000121542	ENSG00000117114	ENSG00000160593
ENSG00000164898	ENSG00000177938	ENSG00000160049
ENSG00000214575	ENSG00000167674	ENSG00000102878
ENSG00000151239	ENSG00000137200	ENSG00000100784
ENSG00000174407	ENSG00000100427	ENSG00000196352
ENSG00000147475	ENSG00000161692	ENSG00000186115
ENSG00000254999	ENSG00000174871	ENSG00000168758
ENSG00000099999	ENSG00000188517	ENSG00000198420
ENSG00000162399	ENSG00000213928	ENSG00000149016
ENSG00000167131	ENSG00000214556	ENSG00000155099
ENSG00000161091	ENSG00000139971	ENSG00000170677
ENSG00000108518	ENSG00000157224	ENSG00000149269
ENSG00000215187	ENSG00000134086	ENSG00000067955
ENSG00000163467	ENSG00000137440	ENSG00000105939
ENSG00000100836	ENSG00000111885	ENSG00000250305
ENSG00000205846	ENSG00000141580	ENSG00000137871
ENSG00000126756	ENSG00000132142	ENSG00000100413
ENSG00000134453	ENSG00000188152	ENSG00000157470
ENSG00000136274	ENSG00000102743	ENSG00000096654
ENSG00000173947	ENSG00000205899	ENSG00000163166
ENSG00000125462	ENSG00000067533	ENSG00000187049
ENSG00000138964	ENSG00000158488	ENSG00000156006
ENSG00000129480	ENSG00000256632	ENSG00000092020
ENSG00000187461	ENSG00000126602	ENSG00000183775
ENSG00000168079	ENSG00000185499	ENSG00000189171
ENSG00000185950	ENSG00000187783	ENSG00000196381
ENSG00000206549	ENSG00000091542	ENSG00000197608
ENSG00000213214	ENSG00000171989	ENSG00000107960
ENSG00000108669	ENSG00000130684	ENSG00000137090
ENSG00000172053	ENSG00000169031	ENSG00000145863
ENSG00000243708	ENSG00000197019	ENSG00000128059
ENSG00000163132	ENSG00000134242	ENSG00000171201
ENSG00000107014	ENSG00000188647	ENSG00000221886
ENSG00000006047	ENSG00000115750	ENSG00000184923
ENSG00000134443	ENSG00000244025	ENSG00000146904
ENSG00000108691	ENSG00000104325	ENSG00000126858
ENSG00000121057	ENSG00000175538	ENSG00000085721
ENSG00000126259	ENSG00000198353	ENSG00000120094
ENSG00000120963	ENSG00000029725	ENSG00000179119
ENSG00000124226	ENSG00000038274	ENSG00000128872
ENSG00000159527	ENSG00000178096	ENSG00000214517
ENSG00000182400	ENSG00000111880	ENSG00000073536
ENSG00000261611	ENSG00000054983	ENSG00000182836
ENSG00000157600	ENSG00000251537	ENSG00000171462

ENSG00000160746	ENSG00000137875	ENSG00000127688
ENSG00000130522	ENSG00000183831	ENSG00000144043
ENSG00000185304	ENSG00000263002	ENSG00000241935
ENSG00000179361	ENSG00000143556	ENSG00000082516
ENSG00000197965	ENSG00000186496	ENSG00000125650
ENSG00000062725	ENSG00000140396	ENSG00000176922
ENSG00000137142	ENSG00000213973	ENSG00000171817
ENSG00000125815	ENSG00000180098	ENSG00000203791
ENSG00000117697	ENSG00000132768	ENSG00000103932
ENSG00000163064	ENSG00000108039	ENSG00000198881
ENSG00000143702	ENSG00000255641	ENSG00000140263
ENSG00000106560	ENSG00000131462	ENSG00000187690
ENSG00000172215	ENSG00000109680	ENSG00000100883
ENSG00000160209	ENSG00000019102	ENSG00000244617
ENSG00000205670	ENSG00000006194	ENSG00000119777
ENSG00000104723	ENSG00000185087	ENSG00000169084
ENSG00000111667	ENSG00000111490	ENSG00000135847
ENSG00000137656	ENSG00000162591	ENSG00000089195
ENSG00000123500	ENSG00000109832	ENSG00000114416
ENSG00000150526	ENSG00000166819	ENSG00000183963
ENSG00000106617	ENSG00000118194	ENSG00000184154
ENSG00000253313	ENSG00000158373	ENSG00000171793
ENSG00000155304	ENSG00000120820	ENSG00000108924
ENSG00000221983	ENSG00000100271	ENSG00000187533
ENSG00000130475	ENSG00000003436	ENSG00000205233
ENSG00000109390	ENSG00000131435	ENSG00000135932
ENSG00000180016	ENSG00000139372	ENSG00000109670
ENSG00000165023	ENSG00000089163	ENSG00000163879
ENSG00000130962	ENSG00000134438	ENSG00000111057
ENSG00000123091	ENSG00000153029	ENSG00000115840
ENSG00000196873	ENSG00000157881	ENSG00000149452
ENSG00000091527	ENSG00000149485	ENSG00000067248
ENSG00000010626	ENSG00000076201	ENSG00000165588
ENSG00000120256	ENSG00000101413	ENSG00000204688
ENSG00000181617	ENSG00000132693	ENSG00000178229
ENSG00000234545	ENSG00000162433	ENSG00000173530
ENSG00000179564	ENSG00000178473	ENSG00000157399
ENSG00000269657	ENSG00000258447	ENSG00000144401
ENSG00000141295	ENSG00000174456	ENSG00000164729
ENSG00000157764	ENSG00000165591	ENSG00000174106
ENSG00000197172	ENSG00000116750	ENSG00000189067
ENSG00000100604	ENSG00000127483	ENSG00000206562
ENSG00000171819	ENSG00000189159	ENSG00000156687
ENSG00000106688	ENSG00000232382	ENSG00000131013
ENSG00000147588	ENSG00000170837	ENSG00000133149
ENSG00000166133	ENSG00000005513	ENSG00000160953
ENSG00000166741	ENSG00000188368	ENSG00000205858
ENSG00000101365	ENSG00000171659	ENSG00000164556
ENSG00000134245	ENSG00000115107	ENSG00000116005
ENSG00000167695	ENSG00000128039	ENSG00000198298
ENSG00000187959	ENSG00000145936	ENSG00000196960
ENSG00000205129	ENSG00000169519	ENSG00000152804
ENSG00000145604	ENSG00000196670	ENSG00000163743
ENSG00000167081	ENSG00000177138	ENSG00000138755
ENSG00000183578	ENSG00000100312	ENSG00000172757
ENSG00000146006	ENSG00000119689	ENSG00000165732
ENSG00000164825	ENSG00000163751	ENSG00000115526
ENSG00000143401	ENSG00000158793	ENSG00000070476
ENSG00000188343	ENSG00000106331	ENSG00000186448
ENSG00000163026	ENSG00000165584	ENSG00000108984
ENSG00000093144	ENSG00000105732	ENSG00000133657
ENSG00000108270	ENSG00000168785	ENSG00000137747
ENSG00000232399	ENSG00000179133	ENSG00000170315
ENSG00000162542	ENSG00000125459	ENSG00000115956

ENSG00000178691	ENSG00000186143	ENSG00000138621
ENSG00000204439	ENSG00000230031	ENSG00000077935
ENSG00000154127	ENSG00000103528	ENSG00000213512
ENSG00000095139	ENSG00000126267	ENSG00000111752
ENSG00000075891	ENSG00000080802	ENSG00000134287
ENSG00000177489	ENSG00000103351	ENSG00000069974
ENSG00000249115	ENSG00000144354	ENSG00000153814
ENSG00000167964	ENSG00000203814	ENSG00000110315
ENSG00000112304	ENSG00000172889	ENSG00000173334
ENSG00000146122	ENSG00000164683	ENSG00000126653
ENSG00000147119	ENSG00000134709	ENSG00000237102
ENSG00000132823	ENSG00000103047	ENSG00000188000
ENSG00000173482	ENSG00000109193	ENSG00000161594
ENSG00000119917	ENSG00000119574	ENSG00000170509
ENSG00000204019	ENSG00000176101	ENSG00000123124
ENSG00000169045	ENSG00000124702	ENSG00000170782
ENSG00000143633	ENSG00000099260	ENSG00000160223
ENSG00000244486	ENSG00000175573	ENSG00000167165
ENSG00000171136	ENSG00000165061	ENSG00000160691
ENSG00000125398	ENSG00000136327	ENSG00000183798
ENSG00000107020	ENSG00000166262	ENSG00000119844
ENSG00000100156	ENSG00000126934	ENSG00000011478
ENSG00000171103	ENSG00000130300	ENSG00000163512
ENSG00000124610	ENSG00000123427	ENSG00000001617
ENSG00000153802	ENSG00000104142	ENSG00000125691
ENSG00000197601	ENSG00000132958	ENSG00000060642
ENSG00000075624	ENSG00000138336	ENSG00000178150
ENSG00000139291	ENSG00000122642	ENSG00000183148
ENSG00000164122	ENSG00000176020	ENSG00000114098
ENSG00000117475	ENSG00000143839	ENSG00000162843
ENSG00000137393	ENSG00000187010	ENSG00000110169
ENSG00000164543	ENSG00000168874	ENSG00000163519
ENSG00000165272	ENSG00000095066	ENSG00000087470
ENSG00000072506	ENSG00000130307	ENSG00000232040
ENSG00000113328	ENSG00000161328	ENSG00000204482
ENSG00000126550	ENSG00000113615	ENSG00000241322
ENSG00000144597	ENSG00000240386	ENSG00000164934
ENSG00000161513	ENSG00000182508	ENSG00000159588
ENSG00000176083	ENSG00000144021	ENSG00000135248
ENSG00000125170	ENSG00000091409	ENSG00000132912
ENSG00000137074	ENSG00000182253	ENSG00000110921
ENSG00000174697	ENSG00000120645	ENSG00000124019
ENSG00000183111	ENSG00000115194	ENSG00000114054
ENSG00000183648	ENSG00000176971	ENSG00000131044
ENSG00000137288	ENSG00000258429	ENSG00000146278
ENSG00000167085	ENSG00000125970	ENSG00000142082
ENSG00000137285	ENSG00000075826	ENSG00000175354
ENSG00000163281	ENSG00000156574	ENSG00000176273
ENSG00000114107	ENSG00000110218	ENSG00000135341
ENSG00000162078	ENSG00000157093	ENSG00000102531
ENSG00000229474	ENSG00000233816	ENSG00000130449
ENSG00000205531	ENSG00000164902	ENSG00000187754
ENSG00000196531	ENSG00000103064	ENSG00000139537
ENSG00000163295	ENSG00000204814	ENSG00000130234
ENSG00000167791	ENSG00000221882	ENSG00000123213
ENSG00000204640	ENSG00000168263	ENSG00000134200
ENSG00000166272	ENSG00000163623	ENSG00000144362
ENSG00000204525	ENSG00000269690	ENSG00000129084
ENSG00000204882	ENSG00000108953	ENSG00000185565
ENSG00000172742	ENSG00000180228	ENSG00000067334
ENSG00000187145	ENSG00000157514	ENSG00000198700
ENSG00000130255	ENSG00000160326	ENSG00000198326
ENSG00000158715	ENSG00000221932	ENSG00000049130
ENSG00000175482	ENSG00000164303	ENSG00000183527

ENSG00000092531	ENSG00000166801	ENSG00000146072
ENSG00000180440	ENSG00000132000	ENSG00000168631
ENSG00000109756	ENSG00000102401	ENSG00000228120
ENSG00000198890	ENSG00000187079	ENSG00000143155
ENSG00000178127	ENSG00000019144	ENSG00000165983
ENSG00000119321	ENSG00000181220	ENSG00000174007
ENSG00000139200	ENSG00000175970	ENSG00000115290
ENSG00000135870	ENSG00000166343	ENSG00000147677
ENSG00000204227	ENSG00000128694	ENSG00000172940
ENSG00000164853	ENSG00000165458	ENSG00000131351
ENSG00000204495	ENSG00000215218	ENSG00000134014
ENSG00000130066	ENSG00000119711	ENSG00000267954
ENSG00000103978	ENSG00000152056	ENSG00000205810
ENSG00000172031	ENSG00000079313	ENSG00000125823
ENSG00000133059	ENSG00000142327	ENSG00000197322
ENSG00000105640	ENSG00000007392	ENSG00000148584
ENSG00000147570	ENSG00000095209	ENSG00000134333
ENSG00000100373	ENSG00000113460	ENSG00000133773
ENSG00000204856	ENSG00000147596	ENSG00000155561
ENSG00000255073	ENSG00000166569	ENSG00000179051
ENSG00000168118	ENSG00000006831	ENSG00000100150
ENSG00000134574	ENSG00000162222	ENSG00000205929
ENSG00000175567	ENSG00000153902	ENSG00000156931
ENSG00000116014	ENSG00000133422	ENSG00000259363
ENSG00000197380	ENSG00000115268	ENSG00000136450
ENSG00000188092	ENSG00000181026	ENSG00000116729
ENSG00000198252	ENSG00000123496	ENSG00000066294
ENSG00000135297	ENSG00000187140	ENSG00000169905
ENSG00000113494	ENSG00000137101	ENSG00000163625
ENSG00000076604	ENSG00000160201	ENSG00000146809
ENSG00000172270	ENSG00000164949	ENSG00000172969
ENSG00000212128	ENSG00000119771	ENSG00000138604
ENSG00000134548	ENSG00000142494	ENSG00000001461
ENSG00000176953	ENSG00000078061	ENSG00000166025
ENSG00000183186	ENSG00000163820	ENSG00000171224
ENSG00000269749	ENSG00000075790	ENSG00000153989
ENSG00000184992	ENSG00000015475	ENSG00000001460
ENSG00000167920	ENSG00000119638	ENSG00000179902
ENSG00000153086	ENSG00000117411	ENSG00000088356
ENSG00000168899	ENSG00000138813	ENSG00000111860
ENSG00000182187	ENSG00000089248	ENSG00000143412
ENSG00000111704	ENSG00000101197	ENSG00000183597
ENSG00000099785	ENSG00000198398	ENSG00000083842
ENSG00000198680	ENSG00000153914	ENSG00000138395
ENSG00000196793	ENSG00000204304	ENSG00000188996
ENSG00000228672	ENSG00000186960	ENSG00000123119
ENSG00000004838	ENSG00000108798	ENSG00000130755
ENSG00000146674	ENSG00000182568	ENSG00000141127
ENSG00000141753	ENSG00000221867	ENSG00000154263
ENSG00000135185	ENSG00000204516	ENSG00000154727
ENSG00000177842	ENSG00000204116	ENSG00000168246
ENSG00000166920	ENSG00000172377	ENSG00000102181
ENSG00000173950	ENSG00000257594	ENSG00000177476
ENSG00000141391	ENSG00000166670	ENSG00000089327
ENSG00000081014	ENSG00000186767	ENSG00000197428
ENSG00000146521	ENSG00000173473	ENSG00000188375
ENSG00000100473	ENSG00000196792	ENSG00000238269
ENSG00000163428	ENSG00000138688	ENSG00000213676
ENSG00000182230	ENSG00000185515	ENSG00000102393
ENSG00000178449	ENSG00000119397	ENSG00000156284
ENSG00000187801	ENSG00000183662	ENSG00000198911
ENSG00000171643	ENSG00000156469	ENSG00000204710
ENSG00000167236	ENSG00000105186	ENSG00000116954
ENSG00000255872	ENSG00000232810	ENSG00000189186

ENSG00000141933	ENSG00000146263	ENSG00000122547
ENSG00000023572	ENSG00000062524	ENSG00000124237
ENSG00000164181	ENSG00000198853	ENSG00000070814
ENSG00000100908	ENSG00000177426	ENSG00000179144
ENSG00000147364	ENSG00000181827	ENSG00000187688
ENSG00000213722	ENSG00000140623	ENSG00000186803
ENSG00000204071	ENSG00000103148	ENSG00000163792
ENSG00000248098	ENSG00000040487	ENSG00000213246
ENSG00000167565	ENSG00000115540	ENSG00000106125
ENSG00000197414	ENSG00000164405	ENSG00000259132
ENSG00000136868	ENSG00000162757	ENSG00000077327
ENSG00000184374	ENSG00000064199	ENSG00000130021
ENSG00000169551	ENSG00000166337	ENSG00000059769
ENSG00000131848	ENSG00000063180	ENSG00000178896
ENSG00000187244	ENSG00000187609	ENSG00000166863
ENSG00000182271	ENSG00000003402	ENSG00000072803
ENSG00000126950	ENSG00000166160	ENSG00000175206
ENSG00000161800	ENSG00000163590	ENSG00000145293
ENSG00000012817	ENSG00000255374	ENSG00000205213
ENSG00000198754	ENSG00000172016	ENSG00000178226
ENSG00000223638	ENSG00000169271	ENSG00000198455
ENSG00000196620	ENSG00000111602	ENSG00000164164
ENSG00000100319	ENSG00000100376	ENSG00000179091
ENSG00000182979	ENSG00000105865	ENSG00000105501
ENSG00000013392	ENSG00000156521	ENSG00000171469
ENSG00000180432	ENSG00000174970	ENSG00000187231
ENSG00000011105	ENSG00000196156	ENSG00000256188
ENSG00000114737	ENSG00000087884	ENSG00000068903
ENSG00000121417	ENSG00000138443	ENSG00000134899
ENSG00000128050	ENSG00000173769	ENSG00000240184
ENSG00000183307	ENSG00000124151	ENSG00000115685
ENSG00000165782	ENSG00000127329	ENSG00000124429
ENSG00000115839	ENSG00000109103	ENSG00000176495
ENSG00000160050	ENSG00000100450	ENSG00000135472
ENSG00000119614	ENSG00000105701	ENSG00000141293
ENSG00000130772	ENSG00000062282	ENSG00000133119
ENSG00000186564	ENSG00000006468	ENSG00000126070
ENSG00000164687	ENSG00000100479	ENSG00000166509
ENSG00000166922	ENSG00000117477	ENSG00000188167
ENSG00000116977	ENSG00000120029	ENSG00000187474
ENSG00000119121	ENSG00000226479	ENSG00000021776
ENSG00000151778	ENSG00000153922	ENSG00000111801
ENSG00000172782	ENSG00000114405	ENSG00000113758
ENSG00000119333	ENSG00000137628	ENSG00000110693
ENSG00000168807	ENSG00000184661	ENSG00000163793
ENSG00000170345	ENSG00000213822	ENSG00000125775
ENSG00000160097	ENSG00000166211	ENSG00000032742
ENSG00000257482	ENSG00000241127	ENSG00000124535
ENSG00000120279	ENSG00000152582	ENSG00000163431
ENSG00000164163	ENSG00000155846	ENSG00000166441
ENSG00000148120	ENSG00000168274	ENSG00000250565
ENSG00000120586	ENSG00000110721	ENSG00000165168
ENSG00000139263	ENSG00000080709	ENSG00000085733
ENSG00000184321	ENSG00000165512	ENSG00000187701
ENSG00000132879	ENSG00000138109	ENSG00000176299
ENSG00000133103	ENSG00000043462	ENSG00000132698
ENSG00000234745	ENSG00000090376	ENSG00000006659
ENSG00000163110	ENSG00000231213	ENSG00000005302
ENSG00000058063	ENSG00000120242	ENSG00000172769
ENSG00000225899	ENSG00000197416	ENSG00000151694
ENSG00000156374	ENSG00000073009	ENSG00000173208
ENSG00000100644	ENSG00000180592	ENSG00000183862
ENSG00000160606	ENSG00000173213	ENSG00000110324
ENSG00000008516	ENSG00000214367	ENSG00000137309

ENSG00000100387	ENSG00000156050	ENSG00000067704
ENSG00000166436	ENSG00000006114	ENSG00000123268
ENSG00000170500	ENSG00000146109	ENSG00000073584
ENSG00000171208	ENSG00000215570	ENSG00000212126
ENSG00000017797	ENSG00000138944	ENSG00000165626
ENSG00000115317	ENSG00000169006	ENSG00000122986
ENSG00000071564	ENSG00000144791	ENSG00000183130
ENSG00000177485	ENSG00000130751	ENSG00000182613
ENSG00000176974	ENSG00000183060	ENSG00000198301
ENSG00000173588	ENSG00000117595	ENSG00000072571
ENSG00000168594	ENSG00000174332	ENSG00000160703
ENSG00000136881	ENSG00000259399	ENSG00000196433
ENSG00000213424	ENSG00000176142	ENSG00000069329
ENSG00000179271	ENSG00000164402	ENSG00000134533
ENSG00000094841	ENSG00000116785	ENSG00000113805
ENSG00000001629	ENSG00000236320	ENSG00000138760
ENSG00000140941	ENSG00000145012	ENSG00000147434
ENSG00000176605	ENSG00000115112	ENSG00000163162
ENSG00000145414	ENSG00000187238	ENSG00000117899
ENSG00000226757	ENSG00000131408	ENSG00000257365
ENSG00000106066	ENSG00000114378	ENSG00000196151
ENSG00000171421	ENSG00000107669	ENSG00000144366
ENSG00000148488	ENSG00000176386	ENSG00000106348
ENSG00000090487	ENSG00000101773	ENSG00000102048
ENSG00000157554	ENSG00000103184	ENSG00000099622
ENSG00000230657	ENSG00000221978	ENSG00000172457
ENSG00000146281	ENSG00000112299	ENSG00000126088
ENSG00000010704	ENSG00000188257	ENSG00000144659
ENSG00000148908	ENSG00000258405	ENSG00000269175
ENSG00000172366	ENSG00000105514	ENSG00000070831
ENSG00000108559	ENSG00000135677	ENSG00000175877
ENSG00000124257	ENSG00000151332	ENSG00000071967
ENSG00000139436	ENSG00000145386	ENSG00000165119
ENSG00000067221	ENSG00000066813	ENSG00000147059
ENSG00000177830	ENSG00000168148	ENSG00000166908
ENSG00000181927	ENSG00000196374	ENSG00000071073
ENSG00000049246	ENSG00000170873	ENSG00000151379
ENSG00000110422	ENSG00000150540	ENSG00000197872
ENSG00000174501	ENSG00000138768	ENSG00000122643
ENSG00000183347	ENSG00000198707	ENSG00000204428
ENSG00000105519	ENSG00000253304	ENSG00000120337
ENSG00000112242	ENSG00000172785	ENSG00000064989
ENSG00000113300	ENSG00000105402	ENSG00000205143
ENSG00000153147	ENSG00000197471	ENSG00000105705
ENSG00000176293	ENSG00000024048	ENSG00000122481
ENSG00000160801	ENSG00000177728	ENSG00000120370
ENSG00000175591	ENSG00000126882	ENSG00000166454
ENSG00000112486	ENSG00000204231	ENSG00000118804
ENSG00000117650	ENSG00000148481	ENSG00000004700
ENSG00000130203	ENSG00000020181	ENSG00000133805
ENSG00000117362	ENSG00000203970	ENSG00000145700
ENSG00000025423	ENSG00000146830	ENSG00000138764
ENSG00000162594	ENSG00000069667	ENSG00000143167
ENSG00000166145	ENSG00000113558	ENSG00000186047
ENSG00000100359	ENSG00000143536	ENSG00000269058
ENSG00000259494	ENSG00000152253	ENSG00000268172
ENSG00000175879	ENSG00000168411	ENSG00000011243
ENSG00000158164	ENSG00000145191	ENSG00000186103
ENSG00000123933	ENSG00000167182	ENSG00000132196
ENSG00000151849	ENSG00000172238	ENSG00000257115
ENSG00000154252	ENSG00000119943	ENSG00000075218
ENSG00000122042	ENSG00000189037	ENSG00000242366
ENSG00000123892	ENSG00000064726	ENSG00000092330
ENSG00000182224	ENSG00000168528	ENSG00000123505

ENSG00000100744	ENSG00000081059	ENSG00000183918
ENSG00000155097	ENSG00000138413	ENSG00000091428
ENSG00000157349	ENSG00000177483	ENSG00000088876
ENSG00000100554	ENSG00000212122	ENSG00000169762
ENSG00000204290	ENSG00000116106	ENSG00000143499
ENSG00000198574	ENSG00000166035	ENSG00000124785
ENSG00000055147	ENSG00000154217	ENSG00000102575
ENSG00000173281	ENSG00000136932	ENSG00000122507
ENSG00000175279	ENSG00000177951	ENSG00000186334
ENSG00000150201	ENSG00000168490	ENSG00000154781
ENSG00000174136	ENSG00000168389	ENSG00000137860
ENSG00000170923	ENSG00000167766	ENSG00000179761
ENSG00000075388	ENSG00000163644	ENSG00000196872
ENSG00000082929	ENSG00000137274	ENSG00000113013
ENSG00000259471	ENSG00000078549	ENSG00000119913
ENSG00000099282	ENSG00000108557	ENSG00000168306
ENSG00000111530	ENSG00000123136	ENSG00000059377
ENSG00000121764	ENSG00000137266	ENSG00000169484
ENSG00000206535	ENSG00000007908	ENSG00000170836
ENSG00000107938	ENSG00000117266	ENSG00000178694
ENSG00000213185	ENSG00000139714	ENSG00000112462
ENSG00000169203	ENSG00000146247	ENSG00000203730
ENSG00000138061	ENSG00000102678	ENSG00000100601
ENSG00000182931	ENSG00000187147	ENSG00000143847
ENSG00000171475	ENSG00000168077	ENSG00000166173
ENSG00000131100	ENSG00000139631	ENSG00000130723
ENSG00000071082	ENSG00000198967	ENSG00000198092
ENSG00000112799	ENSG00000185359	ENSG00000136478
ENSG00000203950	ENSG00000167759	ENSG00000130305
ENSG00000168806	ENSG00000186566	ENSG00000166794
ENSG00000175087	ENSG00000269190	ENSG00000267699
ENSG00000121742	ENSG00000126353	ENSG00000145723
ENSG00000105325	ENSG00000138073	ENSG00000241343
ENSG00000060762	ENSG00000126464	ENSG00000178033
ENSG00000115297	ENSG00000061337	ENSG00000134072
ENSG00000160654	ENSG00000175213	ENSG00000124143
ENSG00000180855	ENSG00000111011	ENSG00000204351
ENSG00000257108	ENSG00000204572	ENSG00000135744
ENSG00000104763	ENSG00000130383	ENSG00000137077
ENSG00000082701	ENSG00000158985	ENSG00000133872
ENSG00000155975	ENSG00000136895	ENSG00000156234
ENSG00000178722	ENSG00000121410	ENSG00000164989
ENSG00000118197	ENSG00000086666	ENSG00000173456
ENSG00000218819	ENSG00000180346	ENSG00000204544
ENSG00000189167	ENSG00000144407	ENSG00000254122
ENSG00000196476	ENSG00000171936	ENSG00000131931
ENSG00000135272	ENSG00000196979	ENSG00000229809
ENSG00000234906	ENSG00000184492	ENSG00000163754
ENSG00000123908	ENSG00000102030	ENSG00000205832
ENSG00000130725	ENSG00000172955	ENSG00000131126
ENSG00000198642	ENSG00000168765	ENSG00000176040
ENSG00000187583	ENSG00000125998	ENSG00000185345
ENSG00000100297	ENSG00000118496	ENSG00000106460
ENSG00000156239	ENSG00000116809	ENSG00000165841
ENSG00000187772	ENSG00000181733	ENSG00000090006
ENSG00000112282	ENSG00000168743	ENSG00000198865
ENSG00000137106	ENSG00000180209	ENSG00000083544
ENSG00000166171	ENSG00000121775	ENSG00000103152
ENSG00000154639	ENSG00000186603	ENSG00000074319
ENSG00000109084	ENSG00000164904	ENSG00000111145
ENSG00000139921	ENSG00000123560	ENSG00000171402
ENSG00000177047	ENSG00000173065	ENSG00000164308
ENSG00000186117	ENSG00000134686	ENSG00000080546
ENSG00000142534	ENSG00000167004	ENSG00000198573

ENSG00000127780	ENSG00000149243	ENSG00000164048
ENSG00000131508	ENSG00000128185	ENSG00000130528
ENSG00000184029	ENSG00000178821	ENSG00000196832
ENSG00000197734	ENSG00000047932	ENSG00000184205
ENSG00000127364	ENSG00000181433	ENSG00000130939
ENSG00000227057	ENSG00000187720	ENSG00000134900
ENSG00000136718	ENSG00000072736	ENSG00000147383
ENSG00000147223	ENSG00000174851	ENSG00000182459
ENSG00000242114	ENSG00000143469	ENSG00000047597
ENSG00000122687	ENSG00000169696	ENSG00000255582
ENSG00000253976	ENSG00000175792	ENSG00000163867
ENSG00000052850	ENSG00000163697	ENSG00000187944
ENSG00000176920	ENSG00000104361	ENSG00000213859
ENSG00000181751	ENSG00000132010	ENSG00000196119
ENSG00000135916	ENSG00000138663	ENSG00000101441
ENSG00000132388	ENSG00000160908	ENSG00000177151
ENSG00000149262	ENSG00000122783	ENSG00000161326
ENSG00000089486	ENSG00000011295	ENSG00000260456
ENSG00000196505	ENSG00000215704	ENSG00000154447
ENSG00000186470	ENSG00000123360	ENSG00000141140
ENSG00000122644	ENSG00000172156	ENSG00000147926
ENSG00000136938	ENSG00000154611	ENSG00000166220
ENSG00000125835	ENSG00000107874	ENSG00000154429
ENSG00000149968	ENSG00000163141	ENSG00000185966
ENSG00000163157	ENSG00000174599	ENSG00000178522
ENSG00000237896	ENSG00000151577	ENSG00000186106
ENSG00000101443	ENSG00000120756	ENSG00000078967
ENSG00000005812	ENSG00000196772	ENSG00000105198
ENSG00000111269	ENSG00000106415	ENSG00000132676
ENSG00000185928	ENSG00000073060	ENSG00000129625
ENSG00000181965	ENSG00000099953	ENSG00000121766
ENSG00000110660	ENSG00000125285	ENSG00000110852
ENSG00000100365	ENSG00000101888	ENSG00000111361
ENSG00000134759	ENSG00000132819	ENSG00000117758
ENSG00000100097	ENSG00000198104	ENSG00000256825
ENSG00000184840	ENSG00000206104	ENSG00000070808
ENSG00000177076	ENSG00000135374	ENSG00000255245
ENSG00000116663	ENSG00000099364	ENSG00000124171
ENSG00000101972	ENSG00000099250	ENSG00000166415
ENSG00000152463	ENSG00000188747	ENSG00000205758
ENSG00000196196	ENSG00000155090	ENSG00000147145
ENSG00000107643	ENSG00000110756	ENSG00000204370
ENSG00000196420	ENSG00000220201	ENSG00000141562
ENSG00000187170	ENSG00000114520	ENSG00000142864
ENSG00000110344	ENSG00000185651	ENSG00000124508
ENSG00000050426	ENSG00000176714	ENSG00000101624
ENSG00000138684	ENSG00000180210	ENSG00000198054
ENSG00000221988	ENSG00000155330	ENSG00000066557
ENSG00000134146	ENSG00000198576	ENSG00000165583
ENSG00000011590	ENSG00000178597	ENSG00000182795
ENSG00000187559	ENSG00000065809	ENSG00000113552
ENSG00000172236	ENSG00000196628	ENSG00000135842
ENSG00000214681	ENSG00000131591	ENSG00000258973
ENSG00000063515	ENSG00000073737	ENSG00000144115
ENSG00000012174	ENSG00000170144	ENSG00000250486
ENSG00000112941	ENSG00000161082	ENSG00000169221
ENSG00000145757	ENSG00000120725	ENSG00000053328
ENSG00000087206	ENSG00000137474	ENSG00000196705
ENSG00000259305	ENSG00000072415	ENSG00000184825
ENSG00000254995	ENSG00000139977	ENSG00000173535
ENSG00000186439	ENSG00000071909	ENSG00000136816
ENSG00000127515	ENSG00000124789	ENSG00000173627
ENSG00000167842	ENSG00000175287	ENSG00000074621
ENSG00000139974	ENSG00000182107	ENSG00000140527

ENSG00000147419	ENSG00000105556	ENSG00000153982
ENSG00000179151	ENSG00000174125	ENSG00000033100
ENSG00000188191	ENSG00000183473	ENSG00000178690
ENSG00000117586	ENSG00000127324	ENSG00000126790
ENSG00000177174	ENSG00000234857	ENSG00000196449
ENSG00000178460	ENSG00000182208	ENSG00000172137
ENSG00000103479	ENSG00000116171	ENSG00000010282
ENSG00000185973	ENSG00000198643	ENSG00000185404
ENSG00000125457	ENSG00000119737	ENSG00000130956
ENSG00000178301	ENSG00000140470	ENSG00000116703
ENSG00000137707	ENSG00000160803	ENSG00000109920
ENSG00000151806	ENSG00000153551	ENSG00000171155
ENSG00000037749	ENSG00000141579	ENSG00000204648
ENSG00000134910	ENSG00000123843	ENSG00000140939
ENSG00000163932	ENSG00000164081	ENSG00000174099
ENSG00000143578	ENSG00000189169	ENSG00000165309
ENSG00000087586	ENSG00000164849	ENSG00000123643
ENSG00000154080	ENSG00000110925	ENSG00000204344
ENSG00000145979	ENSG00000154856	ENSG00000133742
ENSG00000159228	ENSG00000166987	ENSG00000149792
ENSG00000162585	ENSG00000188766	ENSG00000088854
ENSG00000149922	ENSG00000203879	ENSG00000072182
ENSG00000164651	ENSG00000230797	ENSG00000111906
ENSG00000125863	ENSG00000176231	ENSG00000143624
ENSG00000256762	ENSG00000187258	ENSG00000188219
ENSG00000187017	ENSG00000234719	ENSG00000185267
ENSG00000141867	ENSG00000158402	ENSG00000116478
ENSG00000177963	ENSG00000137672	ENSG00000138160
ENSG00000166396	ENSG00000183691	ENSG00000110675
ENSG00000176194	ENSG00000184743	ENSG00000157150
ENSG00000059588	ENSG00000186453	ENSG00000081237
ENSG00000236287	ENSG00000144671	ENSG00000124444
ENSG00000203705	ENSG00000110768	ENSG00000138823
ENSG00000189306	ENSG00000066027	ENSG00000172262
ENSG00000166997	ENSG00000163083	ENSG00000116350
ENSG00000204300	ENSG00000177300	ENSG00000113520
ENSG00000174944	ENSG00000121060	ENSG00000173692
ENSG00000215749	ENSG00000160593	ENSG00000139540
ENSG00000057663	ENSG00000160049	ENSG00000213930
ENSG00000241399	ENSG00000102878	ENSG00000164841
ENSG00000178860	ENSG00000167716	ENSG00000204977
ENSG00000144580	ENSG00000100784	ENSG00000108433
ENSG00000162407	ENSG00000196352	ENSG00000158411
ENSG00000130958	ENSG00000181409	ENSG00000100596
ENSG00000184381	ENSG00000186115	ENSG00000134193
ENSG00000005001	ENSG00000184160	ENSG00000198954
ENSG00000188243	ENSG00000168758	ENSG00000101057
ENSG00000150672	ENSG00000198420	ENSG00000113657
ENSG00000135220	ENSG00000149016	ENSG00000022840
ENSG00000229619	ENSG00000155099	ENSG00000196227
ENSG00000163960	ENSG00000170677	ENSG00000144524
ENSG00000161798	ENSG00000167123	ENSG00000164919
ENSG00000167130	ENSG00000149269	ENSG00000076003
ENSG00000163746	ENSG00000146216	ENSG00000154380
ENSG00000186666	ENSG00000067955	ENSG00000106948
ENSG00000186205	ENSG00000136059	ENSG00000135245
ENSG00000156172	ENSG00000162367	ENSG00000106692
ENSG00000254834	ENSG00000105939	ENSG00000162419
ENSG00000115598	ENSG00000250305	ENSG00000163874
ENSG00000152147	ENSG00000137871	ENSG00000197798
ENSG00000143158	ENSG00000100413	ENSG00000124743
ENSG00000177669	ENSG00000157470	ENSG00000255622
ENSG00000144035	ENSG00000063241	ENSG00000198929
ENSG00000144426	ENSG00000131737	ENSG00000114455

ENSG00000204267	ENSG00000096654	ENSG00000167257
ENSG00000196268	ENSG00000163166	ENSG00000172331
ENSG00000171195	ENSG00000187049	ENSG00000173575
ENSG00000205111	ENSG00000165912	ENSG00000188916
ENSG00000198829	ENSG00000156006	ENSG00000239704
ENSG00000144034	ENSG00000092020	ENSG00000160685
ENSG00000136807	ENSG00000183775	ENSG00000053108
ENSG00000235978	ENSG00000106009	ENSG00000118785
ENSG00000100206	ENSG00000189171	ENSG00000120697
ENSG00000179772	ENSG00000108773	ENSG00000114686
ENSG00000163319	ENSG00000196381	ENSG00000167807
ENSG00000125814	ENSG00000197608	ENSG00000169193
ENSG00000148841	ENSG00000107960	ENSG00000110723
ENSG00000138107	ENSG00000137090	ENSG00000100918
ENSG00000173145	ENSG00000145863	ENSG00000134321
ENSG00000075886	ENSG00000128059	ENSG00000130713
ENSG00000128590	ENSG00000167291	ENSG00000163636
ENSG00000213809	ENSG00000171201	ENSG00000204169
ENSG00000170890	ENSG00000168159	ENSG00000113761
ENSG00000173369	ENSG00000221886	ENSG00000068985
ENSG00000114021	ENSG00000184923	ENSG00000120688
ENSG00000006016	ENSG00000162032	ENSG00000133316
ENSG00000134363	ENSG00000146904	ENSG00000110031
ENSG00000189376	ENSG00000126858	ENSG00000167780
ENSG00000120253	ENSG00000142330	ENSG00000132849
ENSG00000076641	ENSG00000085721	ENSG00000162378
ENSG00000107679	ENSG00000198937	ENSG00000002549
ENSG00000165799	ENSG00000120094	ENSG00000127863
ENSG00000165443	ENSG00000179119	ENSG00000169239
ENSG00000132581	ENSG00000128872	ENSG00000173825
ENSG00000168970	ENSG00000214517	ENSG00000196981
ENSG00000211584	ENSG00000073536	ENSG00000172831
ENSG00000119986	ENSG00000182836	ENSG00000154839
ENSG00000172381	ENSG00000171462	ENSG00000170615
ENSG00000168824	ENSG00000127688	ENSG00000214360
ENSG00000102096	ENSG00000144043	ENSG00000162889
ENSG00000113712	ENSG00000184785	ENSG00000108298
ENSG00000162613	ENSG00000241935	ENSG00000177710
ENSG00000129810	ENSG00000082516	ENSG00000101882
ENSG00000140497	ENSG00000125650	ENSG00000234560
ENSG00000164463	ENSG00000176922	ENSG00000242852
ENSG00000125966	ENSG00000171817	ENSG00000184351
ENSG00000135638	ENSG00000203791	ENSG00000174444
ENSG00000256872	ENSG00000131650	ENSG00000170653
ENSG00000124003	ENSG00000104826	ENSG00000255150
ENSG00000204315	ENSG00000103932	ENSG00000171953
ENSG00000163320	ENSG00000177464	ENSG00000102897
ENSG00000172315	ENSG00000156885	ENSG00000158825
ENSG00000162692	ENSG00000198881	ENSG00000166329
ENSG00000219545	ENSG00000176842	ENSG00000141750
ENSG00000111052	ENSG00000140263	ENSG00000136999
ENSG00000139438	ENSG00000130762	ENSG00000167775
ENSG00000076826	ENSG00000187690	ENSG00000142794
ENSG00000135469	ENSG00000100883	ENSG00000198018
ENSG00000196660	ENSG00000244617	ENSG00000175820
ENSG00000095485	ENSG00000119777	ENSG00000183310
ENSG00000140153	ENSG00000169084	ENSG00000120563
ENSG00000167550	ENSG00000135847	ENSG00000033178
ENSG00000100564	ENSG00000089195	ENSG00000151611
ENSG00000151012	ENSG00000114416	ENSG00000188021
ENSG00000185015	ENSG00000183963	ENSG00000143171
ENSG00000168887	ENSG00000184154	ENSG00000131795
ENSG00000101337	ENSG00000171793	ENSG00000150873
ENSG00000079257	ENSG00000108924	ENSG00000147687

ENSG00000165204	ENSG00000187533	ENSG00000163617
ENSG00000171159	ENSG00000205233	ENSG00000158874
ENSG00000166595	ENSG00000135932	ENSG00000169604
ENSG00000107821	ENSG00000109670	ENSG00000112232
ENSG00000205883	ENSG00000163879	ENSG00000244476
ENSG00000154370	ENSG00000111057	ENSG00000165685
ENSG00000175202	ENSG00000115840	ENSG00000184258
ENSG00000171126	ENSG00000149452	ENSG00000124233
ENSG00000232125	ENSG00000067248	ENSG00000130167
ENSG00000198720	ENSG00000165588	ENSG00000143502
ENSG00000106952	ENSG00000204688	ENSG00000115364
ENSG00000148123	ENSG00000214063	ENSG00000100034
ENSG00000221914	ENSG00000178229	ENSG00000131746
ENSG00000197465	ENSG00000173530	ENSG00000136271
ENSG00000133661	ENSG00000157399	ENSG00000171476
ENSG00000101236	ENSG00000144401	ENSG00000136231
ENSG00000205236	ENSG00000164729	ENSG00000142789
ENSG00000168495	ENSG00000174106	ENSG00000171483
ENSG00000141428	ENSG00000189067	ENSG00000148200
ENSG00000101846	ENSG00000101460	ENSG00000132031
ENSG00000221870	ENSG00000206562	ENSG00000231861
ENSG00000152240	ENSG00000156687	ENSG00000113749
ENSG00000258941	ENSG00000131013	ENSG00000181789
ENSG00000205186	ENSG00000133149	ENSG00000120329
ENSG00000168282	ENSG00000107485	ENSG00000185760
ENSG00000170473	ENSG00000182870	ENSG00000122035
ENSG00000147883	ENSG00000160953	ENSG00000162814
ENSG00000161573	ENSG00000205858	ENSG00000122026
ENSG00000104643	ENSG00000164556	ENSG00000135048
ENSG00000162139	ENSG00000116005	ENSG00000125122
ENSG00000169704	ENSG00000198298	ENSG00000213672
ENSG00000164104	ENSG00000196960	ENSG00000026751
ENSG00000142182	ENSG00000152804	ENSG00000059804
ENSG00000162961	ENSG00000163743	ENSG00000189079
ENSG00000108306	ENSG00000248235	ENSG00000174032
ENSG00000167476	ENSG00000138755	ENSG00000103021
ENSG00000144746	ENSG00000172757	ENSG00000060688
ENSG00000095585	ENSG00000165732	ENSG00000163235
ENSG00000181374	ENSG00000115526	ENSG00000169474
ENSG00000064961	ENSG00000070476	ENSG00000094916
ENSG00000126216	ENSG00000182473	ENSG00000177465
ENSG00000144792	ENSG00000186448	ENSG00000170298
ENSG00000121644	ENSG00000108984	ENSG00000158806
ENSG00000176444	ENSG00000133657	ENSG00000165025
ENSG00000138735	ENSG00000137747	ENSG00000119541
ENSG00000166821	ENSG00000170315	ENSG00000183763
ENSG00000181104	ENSG00000115956	ENSG00000125445
ENSG00000166189	ENSG00000138621	ENSG00000241852
ENSG00000105610	ENSG00000077935	ENSG00000168582
ENSG00000164941	ENSG00000213512	ENSG00000103316
ENSG00000034239	ENSG00000137491	ENSG00000204952
ENSG00000224051	ENSG00000111752	ENSG00000178202
ENSG00000196224	ENSG00000134287	ENSG00000128714
ENSG00000082153	ENSG00000069974	ENSG00000156575
ENSG00000254553	ENSG00000153814	ENSG00000115355
ENSG00000101198	ENSG00000160282	ENSG00000124802
ENSG00000179913	ENSG00000182087	ENSG00000161896
ENSG00000173762	ENSG00000110315	ENSG00000108854
ENSG00000156026	ENSG00000160813	ENSG00000137210
ENSG00000171431	ENSG00000173334	ENSG00000239305
ENSG00000130988	ENSG00000126653	ENSG00000064313
ENSG00000197860	ENSG00000237102	ENSG00000089723
ENSG00000105520	ENSG00000188000	ENSG00000116882
ENSG00000164885	ENSG00000161594	ENSG00000156970

ENSG00000248643	ENSG00000170509	ENSG00000144644
ENSG00000267368	ENSG00000123124	ENSG00000113269
ENSG00000132256	ENSG00000170782	ENSG00000183785
ENSG00000198854	ENSG00000160223	ENSG00000055044
ENSG00000180644	ENSG00000167165	ENSG00000184619
ENSG00000165376	ENSG00000160691	ENSG00000115459
ENSG00000166582	ENSG00000183798	ENSG00000073050
ENSG00000229117	ENSG00000127445	ENSG00000119514
ENSG00000141040	ENSG00000119844	ENSG00000159208
ENSG00000132109	ENSG00000011478	ENSG00000181013
ENSG00000155130	ENSG00000076944	ENSG00000119720
ENSG00000149273	ENSG00000163512	ENSG00000174808
ENSG00000179403	ENSG00000099804	ENSG00000087095
ENSG00000254221	ENSG00000001617	ENSG00000142149
ENSG00000176597	ENSG00000125691	ENSG00000033800
ENSG00000175311	ENSG00000060642	ENSG00000168612
ENSG00000178053	ENSG00000178150	ENSG00000161249
ENSG00000111711	ENSG00000183148	ENSG00000128463
ENSG00000071553	ENSG00000114098	ENSG00000102543
ENSG00000101251	ENSG00000162843	ENSG00000221923
ENSG00000117122	ENSG00000068024	ENSG00000164112
ENSG00000158023	ENSG00000110169	ENSG00000240654
ENSG00000189144	ENSG00000163519	ENSG00000174989
ENSG00000099769	ENSG00000087470	ENSG00000152492
ENSG00000204397	ENSG00000232040	ENSG00000146833
ENSG00000100266	ENSG00000204482	ENSG00000087111
ENSG00000205639	ENSG00000241322	ENSG00000165264
ENSG00000132824	ENSG00000164934	ENSG00000050730
ENSG00000079616	ENSG00000159588	ENSG00000196684
ENSG00000089916	ENSG00000238227	ENSG00000183103
ENSG00000053254	ENSG00000067840	ENSG00000181544
ENSG00000184682	ENSG00000135248	ENSG00000241690
ENSG00000170276	ENSG00000132912	ENSG00000124701
ENSG00000087128	ENSG00000110921	ENSG00000063587
ENSG00000158427	ENSG00000124019	ENSG00000240891
ENSG00000068831	ENSG00000124780	ENSG00000223609
ENSG00000170324	ENSG00000114054	ENSG00000131437
ENSG00000146926	ENSG00000118231	ENSG00000122390
ENSG00000187747	ENSG00000131044	ENSG00000152932
ENSG00000186925	ENSG00000146278	ENSG00000109971
ENSG00000222036	ENSG00000142178	ENSG00000185088
ENSG00000123570	ENSG00000180914	ENSG00000166959
ENSG00000109519	ENSG00000142082	ENSG00000115425
ENSG00000169252	ENSG00000175354	ENSG00000172954
ENSG00000147183	ENSG00000132622	ENSG00000107798
ENSG00000217261	ENSG00000176273	ENSG00000262814
ENSG00000159596	ENSG00000135341	ENSG00000204175
ENSG00000188938	ENSG00000102531	ENSG00000234465
ENSG00000015413	ENSG00000198133	ENSG00000188599
ENSG00000166913	ENSG00000130449	ENSG00000161381
ENSG00000128335	ENSG00000187754	ENSG00000185684
ENSG00000174446	ENSG00000139537	ENSG00000255837
ENSG00000174915	ENSG00000130234	ENSG00000188306
ENSG00000164008	ENSG00000123213	ENSG00000156269
ENSG00000147955	ENSG00000134200	ENSG00000145331
ENSG00000167904	ENSG00000144362	ENSG00000103510
ENSG00000129484	ENSG00000171298	ENSG00000185344
ENSG00000189275	ENSG00000129084	ENSG00000109618
ENSG00000102409	ENSG00000185565	ENSG00000119411
ENSG00000178217	ENSG00000067334	ENSG00000168453
ENSG00000145916	ENSG00000124578	ENSG00000166118
ENSG00000105220	ENSG00000198700	ENSG00000121769
ENSG00000151287	ENSG00000198326	ENSG00000155858
ENSG00000204161	ENSG00000049130	ENSG00000139988

ENSG00000163634	ENSG00000183527	ENSG00000100987
ENSG00000012963	ENSG00000146072	ENSG00000229415
ENSG00000198128	ENSG00000168631	ENSG00000149428
ENSG00000188729	ENSG00000228120	ENSG00000138379
ENSG00000213029	ENSG00000143155	ENSG00000143093
ENSG00000166676	ENSG00000184471	ENSG00000124466
ENSG00000106004	ENSG00000165983	ENSG00000140382
ENSG00000189164	ENSG00000174007	ENSG00000105948
ENSG00000139187	ENSG00000115290	ENSG00000170374
ENSG00000121621	ENSG00000172938	ENSG00000188655
ENSG00000128645	ENSG00000147677	ENSG00000156928
ENSG00000119139	ENSG00000172940	ENSG00000121797
ENSG00000136169	ENSG00000131351	ENSG00000171243
ENSG00000164338	ENSG00000181218	ENSG00000138271
ENSG00000243440	ENSG00000172476	ENSG00000103540
ENSG00000146842	ENSG00000172070	ENSG00000113384
ENSG00000181634	ENSG00000134014	ENSG00000111224
ENSG00000033327	ENSG00000267954	ENSG00000138593
ENSG00000000419	ENSG00000125430	ENSG00000196805
ENSG00000176563	ENSG00000205810	ENSG00000135521
ENSG00000023041	ENSG00000125823	ENSG00000120992
ENSG00000170906	ENSG00000141026	ENSG00000168298
ENSG00000093183	ENSG00000197322	ENSG00000147789
ENSG00000187954	ENSG00000148584	ENSG00000243073
ENSG00000109586	ENSG00000267964	ENSG00000083782
ENSG00000118960	ENSG00000134333	ENSG00000124491
ENSG00000147689	ENSG00000133773	ENSG00000163513
ENSG00000137959	ENSG00000155561	ENSG00000146376
ENSG00000158169	ENSG00000179051	ENSG00000175707
ENSG00000185247	ENSG00000106211	ENSG00000147174
ENSG00000077150	ENSG00000100150	ENSG00000068305
ENSG00000162413	ENSG00000205929	ENSG00000103876
ENSG00000176485	ENSG00000156931	ENSG00000180891
ENSG00000132953	ENSG00000259363	ENSG00000140104
ENSG00000170291	ENSG00000136450	ENSG00000175097
ENSG00000134884	ENSG00000116729	ENSG00000181894
ENSG00000129562	ENSG00000066294	ENSG00000082196
ENSG00000180233	ENSG00000169905	ENSG00000167840
ENSG00000259159	ENSG00000163625	ENSG00000134001
ENSG00000213397	ENSG00000146809	ENSG00000116161
ENSG00000159202	ENSG00000172969	ENSG00000112339
ENSG00000166902	ENSG00000174945	ENSG00000123572
ENSG00000240563	ENSG00000091947	ENSG00000197852
ENSG00000214042	ENSG00000138604	ENSG00000143570
ENSG00000116774	ENSG00000122375	ENSG00000269858
ENSG00000126752	ENSG00000001461	ENSG00000167325
ENSG00000176809	ENSG00000185028	ENSG00000174100
ENSG00000154269	ENSG00000166025	ENSG00000085788
ENSG00000164638	ENSG00000136931	ENSG00000131477
ENSG00000183844	ENSG00000171224	ENSG00000164172
ENSG00000107771	ENSG00000153989	ENSG00000130414
ENSG00000164265	ENSG00000148296	ENSG00000144852
ENSG00000092203	ENSG00000001460	ENSG00000140632
ENSG00000162639	ENSG00000197956	ENSG00000204923
ENSG00000205097	ENSG00000179902	ENSG00000182985
ENSG00000237521	ENSG00000088356	ENSG00000073008
ENSG00000182111	ENSG00000146676	ENSG00000159131
ENSG00000167987	ENSG00000111860	ENSG00000132300
ENSG00000196337	ENSG00000143412	ENSG00000171916
ENSG00000172986	ENSG00000178719	ENSG00000241962
ENSG00000170915	ENSG00000183597	ENSG00000186132
ENSG00000198466	ENSG00000166736	ENSG00000046604
ENSG00000117751	ENSG00000083842	ENSG00000101134
ENSG00000131584	ENSG00000138395	ENSG00000139131

ENSG00000122386	ENSG00000105696	ENSG00000138675
ENSG00000146707	ENSG00000188996	ENSG00000082146
ENSG00000126432	ENSG00000123119	ENSG00000139547
ENSG00000157014	ENSG00000130755	ENSG00000164512
ENSG00000168397	ENSG00000141127	ENSG00000110066
ENSG00000167644	ENSG00000154263	ENSG00000155962
ENSG00000159261	ENSG00000154727	ENSG00000140474
ENSG00000183395	ENSG00000168246	ENSG00000196353
ENSG00000115386	ENSG00000126895	ENSG00000102931
ENSG00000133789	ENSG00000102181	ENSG00000091138
ENSG00000135829	ENSG00000177476	ENSG00000114354
ENSG00000205744	ENSG00000089327	ENSG00000181803
ENSG00000080371	ENSG00000197428	ENSG00000091640
ENSG00000179059	ENSG00000188375	ENSG00000169469
ENSG00000167637	ENSG00000187678	ENSG00000139220
ENSG00000114331	ENSG00000238269	ENSG00000251247
ENSG00000177463	ENSG00000116035	ENSG00000188549
ENSG00000115523	ENSG00000213676	ENSG00000012048
ENSG00000171703	ENSG00000102393	ENSG00000196275
ENSG00000091732	ENSG00000156284	ENSG00000173702
ENSG00000164530	ENSG00000198911	ENSG00000204385
ENSG00000183864	ENSG00000204710	ENSG00000151023
ENSG00000165046	ENSG00000116954	ENSG00000166224
ENSG00000163206	ENSG00000189186	ENSG00000141506
ENSG00000163918	ENSG00000122547	ENSG00000203863
ENSG00000167196	ENSG00000161835	ENSG00000165476
ENSG00000157884	ENSG00000124237	ENSG00000105854
ENSG00000105258	ENSG00000070814	ENSG00000100221
ENSG00000197702	ENSG00000179144	ENSG00000147257
ENSG00000166529	ENSG00000187688	ENSG00000175485
ENSG00000138942	ENSG00000124074	ENSG00000117090
ENSG00000159140	ENSG00000186803	ENSG00000100385
ENSG00000150756	ENSG00000163792	ENSG00000144015
ENSG00000025770	ENSG00000213246	ENSG00000206483
ENSG00000268662	ENSG00000106125	ENSG00000037637
ENSG00000104980	ENSG00000259132	ENSG00000135211
ENSG00000159905	ENSG00000077327	ENSG00000140395
ENSG00000131042	ENSG00000196866	ENSG00000119965
ENSG00000106565	ENSG00000130021	ENSG00000033011
ENSG00000100294	ENSG00000059769	ENSG00000110042
ENSG00000198355	ENSG00000178896	ENSG00000141052
ENSG00000204488	ENSG00000105447	ENSG00000213139
ENSG00000054179	ENSG00000166863	ENSG00000016490
ENSG00000100739	ENSG00000132821	ENSG00000266173
ENSG00000186832	ENSG00000072803	ENSG00000069535
ENSG00000203923	ENSG00000175206	ENSG00000184716
ENSG00000142530	ENSG00000145293	ENSG00000113194
ENSG00000047621	ENSG00000184261	ENSG00000169064
ENSG00000128298	ENSG00000205213	ENSG00000179397
ENSG00000166925	ENSG00000178226	ENSG00000158850
ENSG00000107831	ENSG00000198455	ENSG00000242574
ENSG00000197775	ENSG00000065268	ENSG00000107140
ENSG00000035141	ENSG00000164164	ENSG00000163116
ENSG00000177700	ENSG00000179091	ENSG00000244362
ENSG00000092199	ENSG00000105501	ENSG00000149658
ENSG00000131634	ENSG00000122180	ENSG00000064270
ENSG00000071462	ENSG00000184545	ENSG00000111796
ENSG00000253626	ENSG00000008853	ENSG00000163032
ENSG00000176623	ENSG00000171469	ENSG00000168090
ENSG00000075292	ENSG00000132003	ENSG00000163530
ENSG00000162772	ENSG00000187231	ENSG00000139636
ENSG00000143590	ENSG00000256188	ENSG00000183508
ENSG00000186468	ENSG00000068903	ENSG00000163347
ENSG00000163516	ENSG00000134899	ENSG00000145779

ENSG00000143851	ENSG00000240184	ENSG00000257923
ENSG00000181191	ENSG00000115685	ENSG00000197632
ENSG00000243477	ENSG00000124429	ENSG00000255737
ENSG00000158813	ENSG00000176495	ENSG00000149503
ENSG00000152954	ENSG00000135472	ENSG00000176200
ENSG00000205086	ENSG00000141293	ENSG00000149124
ENSG00000167157	ENSG00000133119	ENSG00000124587
ENSG00000176401	ENSG00000102882	ENSG00000162231
ENSG00000176547	ENSG00000126070	ENSG00000164871
ENSG00000205076	ENSG00000166509	ENSG00000144827
ENSG00000107341	ENSG00000188167	ENSG00000185909
ENSG00000168385	ENSG00000126457	ENSG00000179950
ENSG00000237136	ENSG00000187474	ENSG00000106537
ENSG00000171827	ENSG00000021776	ENSG00000162520
ENSG00000162194	ENSG00000111801	ENSG00000242252
ENSG00000158987	ENSG00000113758	ENSG00000101384
ENSG00000102981	ENSG00000110693	ENSG00000248592
ENSG00000131966	ENSG00000163793	ENSG00000205636
ENSG00000249931	ENSG00000213996	ENSG00000197683
ENSG00000085449	ENSG00000149679	ENSG00000139865
ENSG00000135926	ENSG00000125775	ENSG00000172409
ENSG00000112664	ENSG00000032742	ENSG00000181195
ENSG00000120539	ENSG00000124535	ENSG00000122033
ENSG00000197385	ENSG00000181619	ENSG00000149548
ENSG00000096264	ENSG00000163431	ENSG00000119812
ENSG00000135002	ENSG00000166441	ENSG00000104852
ENSG00000125967	ENSG00000250565	ENSG00000184271
ENSG00000166452	ENSG00000149435	ENSG00000143797
ENSG00000168096	ENSG00000110711	ENSG00000078237
ENSG00000152611	ENSG00000165168	ENSG00000147123
ENSG00000173744	ENSG00000085733	ENSG00000169214
ENSG00000267561	ENSG00000187701	ENSG00000168214
ENSG00000145743	ENSG00000176299	ENSG00000005469
ENSG00000158517	ENSG00000132698	ENSG00000161642
ENSG00000089902	ENSG00000006659	ENSG00000163630
ENSG00000109205	ENSG00000005302	ENSG00000185940
ENSG00000221933	ENSG00000172769	ENSG00000145715
ENSG00000182601	ENSG00000150967	ENSG00000182919
ENSG00000173163	ENSG00000183496	ENSG00000124818
ENSG00000183562	ENSG00000151694	ENSG00000031003
ENSG00000026297	ENSG00000173208	ENSG00000081791
ENSG00000171566	ENSG00000177875	ENSG00000175063
ENSG00000118363	ENSG00000183862	ENSG00000080031
ENSG00000162852	ENSG00000150281	ENSG00000090612
ENSG00000130368	ENSG00000110324	ENSG00000121774
ENSG00000010438	ENSG00000187045	ENSG00000182652
ENSG00000197599	ENSG00000137309	ENSG00000091483
ENSG00000165406	ENSG00000103647	ENSG00000043143
ENSG00000242259	ENSG00000067704	ENSG00000177352
ENSG00000204702	ENSG00000123268	ENSG00000106648
ENSG00000110108	ENSG00000073584	ENSG00000182544
ENSG00000158201	ENSG00000212126	ENSG00000224186
ENSG00000267467	ENSG00000172893	ENSG00000111877
ENSG00000137207	ENSG00000165626	ENSG00000133706
ENSG00000130385	ENSG00000122986	ENSG00000185339
ENSG00000117419	ENSG00000183130	ENSG00000120498
ENSG00000072134	ENSG00000182613	ENSG00000009830
ENSG00000176383	ENSG00000198301	ENSG00000157168
ENSG00000185730	ENSG00000072571	ENSG00000137574
ENSG00000145050	ENSG00000160703	ENSG00000163933
ENSG00000115687	ENSG00000157322	ENSG00000244734
ENSG00000106034	ENSG00000196433	ENSG00000079739
ENSG00000188803	ENSG00000069329	ENSG00000110104
ENSG00000204478	ENSG00000122176	ENSG00000118518

ENSG00000104814	ENSG00000006059	ENSG00000008018
ENSG00000128585	ENSG00000134533	ENSG00000119900
ENSG00000135517	ENSG00000113805	ENSG00000214753
ENSG00000181847	ENSG00000138760	ENSG00000167646
ENSG00000167562	ENSG00000147434	ENSG00000165837
ENSG00000128276	ENSG00000163162	ENSG00000123374
ENSG00000175826	ENSG00000117899	ENSG00000164749
ENSG00000185669	ENSG00000257365	ENSG00000131788
ENSG00000188725	ENSG00000196151	ENSG00000123610
ENSG00000137673	ENSG00000144366	ENSG00000149599
ENSG00000174579	ENSG00000106348	ENSG00000182621
ENSG00000125363	ENSG00000102048	ENSG00000146416
ENSG00000198431	ENSG00000101280	ENSG00000169118
ENSG00000170803	ENSG00000099622	ENSG00000128652
ENSG00000150750	ENSG00000172457	ENSG00000188334
ENSG00000166816	ENSG00000126088	ENSG00000120063
ENSG00000165496	ENSG00000144659	ENSG00000156599
ENSG00000135972	ENSG00000269175	ENSG00000125166
ENSG00000124209	ENSG00000070831	ENSG00000186889
ENSG00000114446	ENSG00000175877	ENSG00000183304
ENSG00000262302	ENSG00000118526	ENSG00000111203
ENSG00000180772	ENSG00000071967	ENSG00000104047
ENSG00000256646	ENSG00000160200	ENSG00000247746
ENSG00000100348	ENSG00000165119	ENSG00000175166
ENSG00000172404	ENSG00000147059	ENSG00000106459
ENSG00000068976	ENSG00000105963	ENSG00000187123
ENSG00000137100	ENSG00000166908	ENSG00000171865
ENSG00000126226	ENSG00000162430	ENSG00000142507
ENSG00000170647	ENSG00000071073	ENSG00000141424
ENSG00000174721	ENSG00000151379	ENSG00000155011
ENSG00000173610	ENSG00000197872	ENSG00000115568
ENSG00000140368	ENSG00000233670	ENSG00000206536
ENSG00000254685	ENSG00000122643	ENSG00000087088
ENSG00000231389	ENSG00000151502	ENSG00000183695
ENSG00000136783	ENSG00000204428	ENSG00000149488
ENSG00000145949	ENSG00000135722	ENSG00000162456
ENSG00000100614	ENSG00000120337	ENSG00000013561
ENSG00000134531	ENSG00000064989	ENSG00000170417
ENSG00000065243	ENSG00000205143	ENSG00000068394
ENSG00000166971	ENSG00000105705	ENSG00000134717
ENSG00000171204	ENSG00000122481	ENSG00000121075
ENSG00000143494	ENSG00000120370	ENSG00000196968
ENSG00000204503	ENSG00000166454	ENSG00000164049
ENSG00000169962	ENSG00000118804	ENSG00000121864
ENSG00000081377	ENSG00000004700	ENSG00000236334
ENSG00000174950	ENSG00000133805	ENSG00000186912
ENSG00000187627	ENSG00000145700	ENSG00000174226
ENSG00000139233	ENSG00000138764	ENSG00000089737
ENSG00000071127	ENSG00000143167	ENSG00000107164
ENSG00000100341	ENSG00000186047	ENSG00000204532
ENSG00000156804	ENSG00000205922	ENSG00000187912
ENSG00000121931	ENSG00000269058	ENSG00000173085
ENSG00000186166	ENSG00000268172	ENSG00000080511
ENSG00000186105	ENSG00000011243	ENSG00000147854
ENSG00000188626	ENSG00000186103	ENSG00000163803
ENSG00000116815	ENSG00000132196	ENSG00000101457
ENSG00000128250	ENSG00000173566	ENSG00000204446
ENSG00000169914	ENSG00000257115	ENSG00000214643
ENSG00000178343	ENSG00000139433	ENSG00000149531
ENSG00000100823	ENSG00000254647	ENSG00000172432
ENSG00000006625	ENSG00000075218	ENSG00000090512
ENSG00000031691	ENSG00000242366	ENSG00000197520
ENSG00000143947	ENSG00000092330	ENSG00000196936
ENSG00000145365	ENSG00000123505	ENSG00000172803

ENSG00000116688	ENSG00000183918	ENSG00000178789
ENSG00000121858	ENSG00000091428	ENSG00000035687
ENSG00000122863	ENSG00000088876	ENSG00000164647
ENSG00000157214	ENSG00000169762	ENSG00000104055
ENSG00000188086	ENSG00000143499	ENSG00000257138
ENSG00000243364	ENSG00000124785	ENSG00000105889
ENSG00000184148	ENSG00000177595	ENSG00000147649
ENSG00000090372	ENSG00000117480	ENSG00000183161
ENSG00000076513	ENSG00000102575	ENSG00000240224
ENSG00000127588	ENSG00000122507	ENSG00000175170
ENSG00000140961	ENSG00000186334	ENSG00000160741
ENSG00000184281	ENSG00000154781	ENSG00000156642
ENSG00000087995	ENSG00000137860	ENSG00000186260
ENSG00000156413	ENSG00000147454	ENSG00000183287
ENSG00000166546	ENSG00000179761	ENSG00000147041
ENSG00000198488	ENSG00000196872	ENSG00000134489
ENSG00000131097	ENSG00000113013	ENSG00000180219
ENSG00000198171	ENSG00000119913	ENSG00000145912
ENSG00000159885	ENSG00000168306	ENSG00000079150
ENSG00000087916	ENSG00000059377	ENSG00000074696
ENSG00000198551	ENSG00000169484	ENSG00000018236
ENSG00000136840	ENSG00000170836	ENSG00000256018
ENSG00000176900	ENSG00000178694	ENSG00000158423
ENSG00000205081	ENSG00000112462	ENSG00000112200
ENSG00000152672	ENSG00000203730	ENSG00000100991
ENSG00000178199	ENSG00000124422	ENSG00000102710
ENSG00000134755	ENSG00000100601	ENSG00000165973
ENSG00000240505	ENSG00000143847	ENSG00000067113
ENSG00000065548	ENSG00000166173	ENSG00000172936
ENSG00000184898	ENSG00000130723	ENSG00000006576
ENSG00000169733	ENSG00000198092	ENSG00000170537
ENSG00000189367	ENSG00000136478	ENSG00000131061
ENSG00000145358	ENSG00000130305	ENSG00000102921
ENSG00000105676	ENSG00000166794	ENSG00000078399
ENSG00000157837	ENSG00000267699	ENSG00000163909
ENSG00000070010	ENSG00000179832	ENSG00000126698
ENSG00000197614	ENSG00000131242	ENSG00000243955
ENSG00000187522	ENSG00000145723	ENSG00000077147
ENSG00000104518	ENSG00000241343	ENSG00000140525
ENSG00000180616	ENSG00000167772	ENSG00000189068
ENSG00000131844	ENSG00000178033	ENSG00000154328
ENSG00000186881	ENSG00000134072	ENSG00000189369
ENSG00000110048	ENSG00000124143	ENSG00000150628
ENSG00000154721	ENSG00000204351	ENSG00000147443
ENSG00000142409	ENSG00000135744	ENSG00000172336
ENSG00000187951	ENSG00000137077	ENSG00000036672
ENSG00000102383	ENSG00000133872	ENSG00000123444
ENSG00000169435	ENSG00000156234	ENSG00000180964
ENSG00000204421	ENSG00000164989	ENSG00000111596
ENSG00000213937	ENSG00000173456	ENSG00000143418
ENSG00000179958	ENSG00000204544	ENSG00000269048
ENSG00000138326	ENSG00000172780	ENSG00000152254
ENSG00000111817	ENSG00000254122	ENSG00000269369
ENSG00000157823	ENSG00000131931	ENSG00000118246
ENSG00000128789	ENSG00000229809	ENSG00000176034
ENSG00000249034	ENSG00000163754	ENSG00000237452
ENSG00000161992	ENSG00000205832	ENSG00000116752
ENSG00000148331	ENSG00000131126	ENSG00000137817
ENSG00000165804	ENSG00000176040	ENSG00000204508
ENSG00000176236	ENSG00000185345	ENSG00000153208
ENSG00000197905	ENSG00000186501	ENSG00000187516
ENSG00000101745	ENSG00000106460	ENSG00000159251
ENSG00000056050	ENSG00000165841	ENSG00000154743
ENSG00000228716	ENSG00000169100	ENSG00000164933

ENSG00000104660	ENSG00000090006	ENSG00000168158
ENSG00000028203	ENSG00000185504	ENSG00000102547
ENSG00000054965	ENSG00000198865	ENSG00000115163
ENSG00000100867	ENSG00000083544	ENSG00000036549
ENSG00000132386	ENSG00000104899	ENSG00000115307
ENSG00000163479	ENSG00000103152	ENSG00000175931
ENSG00000142694	ENSG00000178297	ENSG00000196460
ENSG00000142748	ENSG00000183072	ENSG00000147894
ENSG00000183808	ENSG00000074319	ENSG00000184014
ENSG00000141385	ENSG00000244752	ENSG00000183570
ENSG00000063601	ENSG00000111145	ENSG00000133710
ENSG00000124762	ENSG00000213906	ENSG00000112079
ENSG00000105672	ENSG00000171402	ENSG00000131828
ENSG00000120471	ENSG00000164308	ENSG00000178913
ENSG00000198464	ENSG00000080546	ENSG00000148965
ENSG00000100890	ENSG00000198573	ENSG00000185436
ENSG00000182909	ENSG00000164048	ENSG00000137962
ENSG00000152467	ENSG00000130528	ENSG00000170631
ENSG00000158006	ENSG00000011083	ENSG00000065978
ENSG00000163082	ENSG00000164363	ENSG00000110448
ENSG00000171016	ENSG00000196832	ENSG00000113369
ENSG00000186493	ENSG00000184205	ENSG00000110455
ENSG00000183801	ENSG00000130939	ENSG00000066926
ENSG00000212933	ENSG00000134900	ENSG00000120265
ENSG00000112877	ENSG00000134030	ENSG00000135999
ENSG00000144040	ENSG00000147383	ENSG00000119487
ENSG00000173389	ENSG00000182459	ENSG00000131721
ENSG00000146757	ENSG00000047597	ENSG00000030419
ENSG00000100290	ENSG00000255582	ENSG00000075213
ENSG00000107819	ENSG00000163867	ENSG00000110777
ENSG00000108830	ENSG00000187944	ENSG00000143321
ENSG00000269526	ENSG00000213859	ENSG00000169813
ENSG00000159496	ENSG00000196119	ENSG00000119718
ENSG00000137409	ENSG00000101441	ENSG00000102738
ENSG00000136371	ENSG00000177151	ENSG00000077238
ENSG00000006210	ENSG00000161326	ENSG00000164756
ENSG00000140107	ENSG00000260456	ENSG00000013810
ENSG00000109046	ENSG00000154447	ENSG00000082074
ENSG00000169032	ENSG00000133937	ENSG00000179941
ENSG00000179172	ENSG00000145920	ENSG00000186777
ENSG00000152332	ENSG00000142513	ENSG00000205328
ENSG00000249679	ENSG00000141140	ENSG00000124440
ENSG00000268367	ENSG00000147926	ENSG00000166012
ENSG00000160213	ENSG00000166220	ENSG00000172150
ENSG00000175782	ENSG00000123364	ENSG00000130038
ENSG00000011052	ENSG00000154429	ENSG00000160678
ENSG00000181856	ENSG00000185966	ENSG00000110063
ENSG00000183722	ENSG00000178522	ENSG00000163904
ENSG00000070018	ENSG00000186106	ENSG00000163714
ENSG00000167283	ENSG00000078967	ENSG00000160307
ENSG00000175224	ENSG00000105198	ENSG00000106258
ENSG00000109881	ENSG00000132676	ENSG00000051108
ENSG00000196844	ENSG00000129625	ENSG00000106018
ENSG00000184224	ENSG00000121766	ENSG00000179029
ENSG00000182481	ENSG00000110852	ENSG00000136805
ENSG00000255012	ENSG00000145423	ENSG00000169507
ENSG00000204979	ENSG00000111361	ENSG00000221887
ENSG00000139725	ENSG00000117758	ENSG00000102908
ENSG00000148677	ENSG00000256825	ENSG00000164125
ENSG00000181467	ENSG00000070808	ENSG00000135953
ENSG00000116017	ENSG00000255245	ENSG00000115221
ENSG00000181982	ENSG00000124171	ENSG00000167771
ENSG00000162971	ENSG00000155265	ENSG00000138629
ENSG00000124193	ENSG00000180929	ENSG00000168491

ENSG00000144118	ENSG00000166415	ENSG00000119421
ENSG00000145782	ENSG00000205758	ENSG00000188869
ENSG00000132141	ENSG00000161714	ENSG00000181754
ENSG00000090273	ENSG00000147145	ENSG00000115020
ENSG00000057704	ENSG00000204370	ENSG00000130173
ENSG00000161048	ENSG00000141562	ENSG00000151465
ENSG00000156030	ENSG00000142864	ENSG00000169299
ENSG00000188643	ENSG00000108309	ENSG00000219016
ENSG00000162645	ENSG00000124508	ENSG00000077498
ENSG00000188076	ENSG00000101624	ENSG00000135862
ENSG00000197409	ENSG00000183709	ENSG00000121446
ENSG00000086200	ENSG00000198054	ENSG00000108469
ENSG00000116717	ENSG00000066557	ENSG00000115592
ENSG00000113141	ENSG00000165583	ENSG00000129158
ENSG00000118263	ENSG00000182795	ENSG00000221990
ENSG00000164610	ENSG00000065057	ENSG00000105483
ENSG00000116898	ENSG00000113552	ENSG00000139154
ENSG00000189099	ENSG00000135842	ENSG00000102572
ENSG00000102109	ENSG00000258973	ENSG00000256206
ENSG00000185869	ENSG00000144115	ENSG00000163848
ENSG00000108688	ENSG00000250486	ENSG00000213471
ENSG00000269741	ENSG00000169221	ENSG00000174982
ENSG00000220575	ENSG00000053328	ENSG00000178645
ENSG00000144214	ENSG00000196705	ENSG00000180304
ENSG00000178928	ENSG00000184825	ENSG00000178425
ENSG00000137563	ENSG00000173535	ENSG00000115263
ENSG00000141447	ENSG00000105655	ENSG00000257599
ENSG00000181616	ENSG00000156427	ENSG00000069869
ENSG00000173988	ENSG00000136816	ENSG00000112473
ENSG00000156697	ENSG00000173627	ENSG00000131773
ENSG00000173171	ENSG00000074621	ENSG00000167371
ENSG00000188582	ENSG00000140527	ENSG00000204348
ENSG00000179021	ENSG00000153982	ENSG00000197343
ENSG00000152443	ENSG00000182500	ENSG00000133318
ENSG00000116747	ENSG00000033100	ENSG00000182035
ENSG00000108264	ENSG00000178690	ENSG00000132424
ENSG00000188269	ENSG00000126790	ENSG00000136541
ENSG00000066422	ENSG00000196449	ENSG00000244588
ENSG00000198681	ENSG00000172137	ENSG00000133619
ENSG00000133641	ENSG00000010282	ENSG00000087302
ENSG00000175018	ENSG00000185404	ENSG00000165322
ENSG00000204571	ENSG00000130956	ENSG00000158856
ENSG00000112290	ENSG00000116703	ENSG00000141576
ENSG00000134202	ENSG00000109920	ENSG00000167360
ENSG00000179331	ENSG00000171155	ENSG00000155100
ENSG00000122140	ENSG00000204648	ENSG00000163660
ENSG00000092853	ENSG00000140939	ENSG00000110436
ENSG00000260272	ENSG00000174099	ENSG00000170464
ENSG00000164073	ENSG00000165309	ENSG00000163517
ENSG00000178761	ENSG00000169926	ENSG00000143337
ENSG00000106028	ENSG00000123643	ENSG00000121314
ENSG00000111886	ENSG00000187634	ENSG00000106341
ENSG00000178295	ENSG00000126106	ENSG00000145321
ENSG00000138594	ENSG00000204344	ENSG00000129245
ENSG00000095380	ENSG00000133742	ENSG00000153822
ENSG00000119915	ENSG00000149792	ENSG00000197958
ENSG00000170367	ENSG00000169692	ENSG00000086827
ENSG00000167930	ENSG00000088854	ENSG00000115216
ENSG00000078596	ENSG00000072182	ENSG00000170624
ENSG00000120049	ENSG00000111906	ENSG00000196357
ENSG00000176422	ENSG00000143624	ENSG00000163631
ENSG00000183631	ENSG00000188219	ENSG00000126822
ENSG00000174547	ENSG00000107317	ENSG00000164187
ENSG00000129993	ENSG00000185267	ENSG00000169139

ENSG00000054219	ENSG00000116478	ENSG00000154518
ENSG00000028528	ENSG00000138160	ENSG00000178562
ENSG00000203909	ENSG00000171345	ENSG00000006118
ENSG00000140043	ENSG00000110675	ENSG00000134852
ENSG00000181023	ENSG00000105583	ENSG00000169605
ENSG00000109180	ENSG00000157150	ENSG00000169136
ENSG00000100253	ENSG00000081237	ENSG00000092098
ENSG00000204084	ENSG00000124444	ENSG00000118997
ENSG00000156875	ENSG00000138823	ENSG00000186924
ENSG00000266202	ENSG00000215912	ENSG00000153767
ENSG00000182518	ENSG00000172262	ENSG00000102053
ENSG00000213626	ENSG00000116350	ENSG00000165972
ENSG00000101220	ENSG00000113520	ENSG00000108963
ENSG00000163006	ENSG00000173692	ENSG00000186825
ENSG00000166123	ENSG00000139540	ENSG00000003147
ENSG00000136897	ENSG00000213930	ENSG00000152022
ENSG00000133935	ENSG00000090971	ENSG00000140750
ENSG00000088387	ENSG00000164841	ENSG00000110844
ENSG00000120948	ENSG00000204977	ENSG00000070718
ENSG00000198039	ENSG00000169992	ENSG00000184724
ENSG00000105352	ENSG00000162068	ENSG00000188581
ENSG00000113387	ENSG00000108433	ENSG00000092847
ENSG00000158435	ENSG00000158411	ENSG00000124459
ENSG00000078804	ENSG00000011332	ENSG00000159479
ENSG00000170043	ENSG00000100596	ENSG00000102977
ENSG00000144810	ENSG00000179023	ENSG00000092969
ENSG00000179698	ENSG00000134193	ENSG00000260916
ENSG00000177096	ENSG00000198954	ENSG00000104804
ENSG00000136925	ENSG00000101057	ENSG00000180913
ENSG00000135919	ENSG00000113657	ENSG00000184900
ENSG00000106780	ENSG00000022840	ENSG00000133858
ENSG00000182450	ENSG00000141854	ENSG00000232268
ENSG00000167633	ENSG00000196227	ENSG00000169288
ENSG00000186432	ENSG00000144524	ENSG00000156273
ENSG00000131015	ENSG00000164919	ENSG00000188822
ENSG00000154274	ENSG00000171813	ENSG00000137965
ENSG00000129128	ENSG00000076003	ENSG00000164929
ENSG00000124333	ENSG00000154380	ENSG00000181609
ENSG00000169967	ENSG00000172992	ENSG00000205212
ENSG00000139151	ENSG00000106948	ENSG00000188124
ENSG00000168259	ENSG00000173540	ENSG00000185245
ENSG00000183624	ENSG00000135245	ENSG00000158714
ENSG00000165527	ENSG00000169955	ENSG00000196092
ENSG00000206150	ENSG00000165424	ENSG00000101353
ENSG00000185176	ENSG00000106692	ENSG00000118260
ENSG00000107338	ENSG00000162419	ENSG00000164576
ENSG00000135314	ENSG00000100065	ENSG00000221826
ENSG00000106070	ENSG00000163874	ENSG00000203737
ENSG00000135697	ENSG00000197798	ENSG00000114631
ENSG00000187796	ENSG00000124743	ENSG00000185246
ENSG00000179477	ENSG00000255622	ENSG00000144895
ENSG00000158186	ENSG00000198929	ENSG00000197786
ENSG00000225362	ENSG00000114455	ENSG00000183833
ENSG00000212901	ENSG00000167257	ENSG00000140262
ENSG00000154642	ENSG00000172331	ENSG00000171877
ENSG00000104332	ENSG00000148248	ENSG00000170049
ENSG00000164597	ENSG00000148156	ENSG00000100418
ENSG00000067445	ENSG00000173575	ENSG00000147166
ENSG00000198729	ENSG00000188916	ENSG00000143013
ENSG00000188313	ENSG00000239704	ENSG00000187980
ENSG00000101323	ENSG00000160685	ENSG00000198467
ENSG00000179083	ENSG00000053108	ENSG00000108091
ENSG00000168509	ENSG00000118785	ENSG00000162144
ENSG00000204611	ENSG00000120697	ENSG00000172379

ENSG00000100147	ENSG00000188425	ENSG00000109339
ENSG00000118508	ENSG00000114686	ENSG00000183760
ENSG00000109158	ENSG00000167807	ENSG00000146352
ENSG00000102962	ENSG00000169193	ENSG00000174842
ENSG00000130724	ENSG00000110723	ENSG00000196313
ENSG00000152601	ENSG00000100918	ENSG00000140009
ENSG00000259642	ENSG00000134321	ENSG00000164344
ENSG00000137968	ENSG00000130713	ENSG00000122477
ENSG00000184330	ENSG00000163636	ENSG00000101342
ENSG00000163466	ENSG00000197697	ENSG00000162086
ENSG00000100711	ENSG00000204169	ENSG00000129474
ENSG00000123989	ENSG00000113761	ENSG00000114019
ENSG00000214900	ENSG00000068985	ENSG00000139651
ENSG00000186834	ENSG00000120688	ENSG00000174306
ENSG00000142920	ENSG00000133316	ENSG00000153786
ENSG00000103343	ENSG00000110031	ENSG00000141179
ENSG00000188807	ENSG00000162882	ENSG00000132535
ENSG00000163811	ENSG00000167780	ENSG00000125611
ENSG00000237172	ENSG00000132849	ENSG00000158516
ENSG00000139190	ENSG00000149798	ENSG00000111802
ENSG00000121406	ENSG00000162378	ENSG00000104970
ENSG00000182798	ENSG00000002549	ENSG00000120217
ENSG00000099365	ENSG00000162878	ENSG00000004961
ENSG00000136122	ENSG00000127863	ENSG00000203711
ENSG00000103653	ENSG00000169239	ENSG00000120519
ENSG00000164604	ENSG00000173825	ENSG00000141622
ENSG00000169057	ENSG00000196981	ENSG00000125877
ENSG00000167513	ENSG00000172831	ENSG00000105879
ENSG00000160229	ENSG00000173110	ENSG00000204209
ENSG00000035403	ENSG00000154839	ENSG00000128641
ENSG00000063438	ENSG00000170615	ENSG00000101981
ENSG00000055813	ENSG00000142549	ENSG00000118922
ENSG00000213585	ENSG00000214360	ENSG00000156256
ENSG00000164347	ENSG00000188566	ENSG00000153495
ENSG00000143458	ENSG00000162889	ENSG00000116132
ENSG00000136279	ENSG00000108298	ENSG00000088247
ENSG00000163138	ENSG00000177710	ENSG00000102024
ENSG00000128284	ENSG00000101882	ENSG00000164300
ENSG00000166589	ENSG00000234560	ENSG00000143416
ENSG00000160408	ENSG00000151176	ENSG00000011465
ENSG00000164764	ENSG00000242852	ENSG00000111142
ENSG00000171804	ENSG00000184351	ENSG00000173124
ENSG00000130699	ENSG00000174444	ENSG00000160602
ENSG00000254550	ENSG00000170653	ENSG00000151006
ENSG00000149923	ENSG00000255150	ENSG00000106524
ENSG00000105707	ENSG00000171953	ENSG00000136250
ENSG00000154438	ENSG00000102897	ENSG00000185634
ENSG00000163067	ENSG00000158825	ENSG00000163947
ENSG00000157796	ENSG00000166329	ENSG00000106771
ENSG00000170178	ENSG00000184967	ENSG00000101850
ENSG00000106153	ENSG00000141750	ENSG00000143924
ENSG00000139668	ENSG00000136999	ENSG00000215244
ENSG00000163406	ENSG00000167775	ENSG00000151729
ENSG00000069509	ENSG00000142794	ENSG00000198408
ENSG00000163762	ENSG00000198018	ENSG00000241186
ENSG00000143409	ENSG00000175820	ENSG00000108443
ENSG00000169688	ENSG00000183310	ENSG00000230453
ENSG00000175893	ENSG00000120563	ENSG00000099219
ENSG00000156831	ENSG00000033178	ENSG00000046651
ENSG00000159592	ENSG00000151611	ENSG00000162777
ENSG00000162461	ENSG00000188021	ENSG00000100362
ENSG00000125962	ENSG00000143171	ENSG00000160799
ENSG00000204387	ENSG00000214253	ENSG00000170166
ENSG00000164649	ENSG00000131795	ENSG00000100429

ENSG00000109787	ENSG00000212747	ENSG00000149499
ENSG00000147224	ENSG00000150873	ENSG00000185008
ENSG00000197345	ENSG00000137166	ENSG00000183066
ENSG00000173285	ENSG00000147687	ENSG00000127328
ENSG00000104213	ENSG00000163617	ENSG00000124155
ENSG00000100445	ENSG00000121900	ENSG00000196150
ENSG00000005421	ENSG00000158874	ENSG00000197403
ENSG00000174373	ENSG00000169604	ENSG00000172661
ENSG00000165072	ENSG00000112232	ENSG00000174611
ENSG00000171606	ENSG00000244476	ENSG00000214694
ENSG00000130649	ENSG00000165685	ENSG00000160221
ENSG00000150625	ENSG00000184258	ENSG00000132002
ENSG00000137522	ENSG00000124233	ENSG00000166394
ENSG00000023734	ENSG00000130167	ENSG00000143537
ENSG00000068383	ENSG00000143502	ENSG00000112144
ENSG00000105261	ENSG00000115364	ENSG00000169964
ENSG00000186329	ENSG00000100034	ENSG00000136514
ENSG00000172508	ENSG00000198276	ENSG00000164305
ENSG00000204086	ENSG00000131746	ENSG00000173889
ENSG00000127318	ENSG00000198812	ENSG00000026508
ENSG00000125944	ENSG00000136271	ENSG00000107438
ENSG00000146007	ENSG00000171476	ENSG00000128699
ENSG00000205423	ENSG00000185924	ENSG00000197696
ENSG00000153310	ENSG00000136231	ENSG00000128513
ENSG00000102390	ENSG00000142789	ENSG00000129167
ENSG00000188958	ENSG00000116039	ENSG00000115652
ENSG00000204444	ENSG00000171483	ENSG00000197123
ENSG00000134061	ENSG00000148200	ENSG00000167419
ENSG00000185958	ENSG00000132031	ENSG00000204381
ENSG00000113083	ENSG00000231861	ENSG00000112078
ENSG00000150773	ENSG00000113749	ENSG00000146776
ENSG00000226763	ENSG00000181789	ENSG00000163161
ENSG00000120662	ENSG00000120329	ENSG00000168724
ENSG00000173261	ENSG00000185760	ENSG00000197249
ENSG00000181031	ENSG00000101463	ENSG00000213231
ENSG00000173093	ENSG00000122035	ENSG00000178538
ENSG00000149231	ENSG00000162814	ENSG00000215018
ENSG00000168243	ENSG00000122026	ENSG00000135766
ENSG00000186376	ENSG00000135048	ENSG00000179299
ENSG00000147905	ENSG00000125122	ENSG00000118557
ENSG00000162366	ENSG00000213672	ENSG00000156467
ENSG00000183032	ENSG00000171873	ENSG00000159023
ENSG00000136631	ENSG00000026751	ENSG00000164051
ENSG00000164011	ENSG00000059804	ENSG00000171729
ENSG00000257591	ENSG00000189079	ENSG00000205884
ENSG00000196408	ENSG00000174032	ENSG00000125731
ENSG00000114491	ENSG00000103021	ENSG00000186160
ENSG00000080822	ENSG00000060688	ENSG00000138613
ENSG00000198908	ENSG00000163235	ENSG00000134690
ENSG00000103710	ENSG00000169474	ENSG00000076716
ENSG00000176435	ENSG00000094916	ENSG00000172890
ENSG00000177602	ENSG00000177465	ENSG00000172927
ENSG00000108848	ENSG00000170298	ENSG00000164105
ENSG00000104760	ENSG00000158806	ENSG00000176029
ENSG00000167397	ENSG00000165025	ENSG00000179912
ENSG00000075391	ENSG00000162592	ENSG00000067369
ENSG00000117450	ENSG00000115718	ENSG00000174957
ENSG00000126545	ENSG00000119541	ENSG00000124222
ENSG00000175470	ENSG00000143842	ENSG00000104626
ENSG00000109079	ENSG00000183763	ENSG00000108010
ENSG00000171102	ENSG00000007402	ENSG00000187456
ENSG00000139116	ENSG00000172613	ENSG00000179826
ENSG00000120055	ENSG00000109062	ENSG00000152642
ENSG00000183640	ENSG00000125445	ENSG00000162398

ENSG00000240857	ENSG00000241852	ENSG00000186007
ENSG00000121481	ENSG00000168582	ENSG00000155755
ENSG00000104381	ENSG00000103316	ENSG00000214324
ENSG00000129295	ENSG00000204952	ENSG00000107949
ENSG00000133328	ENSG00000178202	ENSG00000186090
ENSG00000153404	ENSG00000128714	ENSG00000184005
ENSG00000171100	ENSG00000156575	ENSG00000147869
ENSG00000095015	ENSG00000154025	ENSG00000131115
ENSG00000162630	ENSG00000115355	ENSG00000185739
ENSG00000180806	ENSG00000124802	ENSG00000156990
ENSG00000163735	ENSG00000146576	ENSG00000165863
ENSG00000213024	ENSG00000161896	ENSG00000188379
ENSG00000147647	ENSG00000108854	ENSG00000101104
ENSG00000164978	ENSG00000137210	ENSG00000102763
ENSG00000197043	ENSG00000239305	ENSG00000176946
ENSG00000117281	ENSG00000064313	ENSG00000147862
ENSG00000139675	ENSG00000089723	ENSG00000014641
ENSG00000196743	ENSG00000116882	ENSG00000147180
ENSG00000205078	ENSG00000156970	ENSG00000188277
ENSG00000153012	ENSG00000144644	ENSG00000066654
ENSG00000213889	ENSG00000113269	ENSG00000117461
ENSG00000178718	ENSG00000183785	ENSG00000081800
ENSG00000173679	ENSG00000055044	ENSG00000100412
ENSG00000170522	ENSG00000184619	ENSG00000115421
ENSG00000100575	ENSG00000214357	ENSG00000172466
ENSG00000099338	ENSG00000115459	ENSG00000057252
ENSG00000145687	ENSG00000073050	ENSG00000047648
ENSG00000123154	ENSG00000185324	ENSG00000005100
ENSG00000089009	ENSG00000056558	ENSG00000143512
ENSG00000255339	ENSG00000119514	ENSG00000165078
ENSG00000167971	ENSG00000159208	ENSG00000138780
ENSG00000113812	ENSG00000181013	ENSG00000112306
ENSG00000101997	ENSG00000197858	ENSG00000186474
ENSG00000204682	ENSG00000119720	ENSG00000162972
ENSG00000182195	ENSG00000174808	ENSG00000154153
ENSG00000198824	ENSG00000087095	ENSG00000147642
ENSG00000165724	ENSG00000142149	ENSG00000117601
ENSG00000161860	ENSG00000033800	ENSG00000162490
ENSG00000114779	ENSG00000168612	ENSG00000197061
ENSG00000100578	ENSG00000161249	ENSG00000087237
ENSG00000105619	ENSG00000128463	ENSG00000187498
ENSG00000158773	ENSG00000102543	ENSG00000221989
ENSG00000183657	ENSG00000221923	ENSG00000102780
ENSG00000188095	ENSG00000164112	ENSG00000128016
ENSG00000147571	ENSG00000196961	ENSG00000239388
ENSG00000159714	ENSG00000240654	ENSG00000067365
ENSG00000122591	ENSG00000174989	ENSG00000084453
ENSG00000111077	ENSG00000186510	ENSG00000122543
ENSG00000100219	ENSG00000152492	ENSG00000134240
ENSG00000161180	ENSG00000235169	ENSG00000155729
ENSG00000180190	ENSG00000174165	ENSG00000105792
ENSG00000129515	ENSG00000146833	ENSG00000146477
ENSG00000168883	ENSG00000087111	ENSG00000176399
ENSG00000175315	ENSG00000165264	ENSG00000132122
ENSG00000141668	ENSG00000050730	ENSG00000182898
ENSG00000101901	ENSG00000196684	ENSG00000105197
ENSG00000179178	ENSG00000183103	ENSG00000107518
ENSG00000166169	ENSG00000181544	ENSG00000182552
ENSG00000027075	ENSG00000241690	ENSG00000143756
ENSG00000180817	ENSG00000143768	ENSG00000106261
ENSG00000111737	ENSG00000124701	ENSG00000119231
ENSG00000169564	ENSG00000063587	ENSG00000140553
ENSG00000121005	ENSG00000135750	ENSG00000174109
ENSG00000097021	ENSG00000240891	ENSG00000183647

ENSG00000169174	ENSG00000223609	ENSG00000157020
ENSG00000134758	ENSG00000131437	ENSG00000258315
ENSG00000165197	ENSG00000122390	ENSG00000145642
ENSG00000100911	ENSG00000152932	ENSG00000184845
ENSG00000134684	ENSG00000109971	ENSG00000137080
ENSG00000183020	ENSG00000185088	ENSG00000269383
ENSG00000165501	ENSG00000166959	ENSG00000137975
ENSG00000102038	ENSG00000115425	ENSG00000125434
ENSG00000113600	ENSG00000148334	ENSG00000198093
ENSG00000196811	ENSG00000172954	ENSG00000122952
ENSG00000175920	ENSG00000185127	ENSG00000168907
ENSG00000110442	ENSG00000107798	ENSG00000052795
ENSG00000141433	ENSG00000262814	ENSG00000180957
ENSG00000146425	ENSG00000204175	ENSG00000133962
ENSG00000131849	ENSG00000136352	ENSG00000147573
ENSG00000197841	ENSG00000168924	ENSG00000105398
ENSG00000161405	ENSG00000234465	ENSG00000184394
ENSG00000253379	ENSG00000120129	ENSG00000140022
ENSG00000179943	ENSG00000188599	ENSG00000182329
ENSG00000124216	ENSG00000127540	ENSG00000188493
ENSG00000067560	ENSG00000161381	ENSG00000175895
ENSG00000172000	ENSG00000185684	ENSG00000078043
ENSG00000156206	ENSG00000104894	ENSG00000247626
ENSG00000132661	ENSG00000255837	ENSG00000068438
ENSG00000160182	ENSG00000188306	ENSG00000116288
ENSG00000168303	ENSG00000156269	ENSG00000119699
ENSG00000232070	ENSG00000145331	ENSG00000092978
ENSG00000132975	ENSG00000103510	ENSG00000102265
ENSG00000117151	ENSG00000185344	ENSG00000187758
ENSG00000186150	ENSG00000109618	ENSG00000130726
ENSG00000173320	ENSG00000119411	ENSG00000131355
ENSG00000163126	ENSG00000168453	ENSG00000135577
ENSG00000118564	ENSG00000166118	ENSG00000155719
ENSG00000083123	ENSG00000121769	ENSG00000120149
ENSG00000096746	ENSG00000155858	ENSG00000235631
ENSG00000165621	ENSG00000139988	ENSG00000172824
ENSG00000151348	ENSG00000105605	ENSG00000213714
ENSG00000145287	ENSG00000100987	ENSG00000106484
ENSG00000181408	ENSG00000229415	ENSG00000134602
ENSG00000177685	ENSG00000149428	ENSG00000127980
ENSG00000205857	ENSG00000138379	ENSG00000176230
ENSG00000215131	ENSG00000143093	ENSG00000119402
ENSG00000115339	ENSG00000124466	ENSG00000165030
ENSG00000135678	ENSG00000105643	ENSG00000258484
ENSG00000104368	ENSG00000213203	ENSG00000171681
ENSG00000243147	ENSG00000140382	ENSG00000133317
ENSG00000169957	ENSG00000105948	ENSG00000253305
ENSG00000148798	ENSG00000170374	ENSG00000106304
ENSG00000143493	ENSG00000188655	ENSG00000118276
ENSG00000168597	ENSG00000156928	ENSG00000163346
ENSG00000146066	ENSG00000121797	ENSG00000115665
ENSG00000188800	ENSG00000171243	ENSG00000102802
ENSG00000152455	ENSG00000138271	ENSG00000110330
ENSG00000140398	ENSG00000120088	ENSG00000161551
ENSG00000111653	ENSG00000103540	ENSG00000042304
ENSG00000102362	ENSG00000113384	ENSG00000184786
ENSG00000179750	ENSG00000179922	ENSG00000188038
ENSG00000258945	ENSG00000176641	ENSG00000172154
ENSG00000109089	ENSG00000134571	ENSG00000078177
ENSG00000101160	ENSG00000169727	ENSG00000151693
ENSG00000198077	ENSG00000111224	ENSG00000236980
ENSG00000145780	ENSG00000244242	ENSG00000204657
ENSG00000171453	ENSG00000171360	ENSG00000065183
ENSG00000095261	ENSG00000138593	ENSG00000118308

ENSG00000166152	ENSG00000196805	ENSG00000125352
ENSG00000068079	ENSG00000143632	ENSG00000165659
ENSG00000058262	ENSG00000135521	ENSG00000164751
ENSG00000147526	ENSG00000120992	ENSG00000167118
ENSG00000144560	ENSG00000168298	ENSG00000153113
ENSG00000172530	ENSG00000147789	ENSG00000138100
ENSG00000051523	ENSG00000243073	ENSG00000186951
ENSG00000184999	ENSG00000083782	ENSG00000160117
ENSG00000203661	ENSG00000124491	ENSG00000212710
ENSG00000084628	ENSG00000163513	ENSG00000177182
ENSG00000214756	ENSG00000146376	ENSG00000164091
ENSG00000168234	ENSG00000175707	ENSG00000212124
ENSG00000243056	ENSG00000187616	ENSG00000160062
ENSG00000076770	ENSG00000147174	ENSG00000117543
ENSG00000151893	ENSG00000104833	ENSG00000169902
ENSG00000067048	ENSG00000068305	ENSG00000139044
ENSG00000115009	ENSG00000103876	ENSG00000101574
ENSG00000187068	ENSG00000167600	ENSG00000119703
ENSG00000165714	ENSG00000180891	ENSG00000111231
ENSG00000178342	ENSG00000140104	ENSG00000213413
ENSG00000180061	ENSG00000175097	ENSG00000205363
ENSG00000196950	ENSG00000156587	ENSG00000073849
ENSG00000026025	ENSG00000090674	ENSG00000173442
ENSG00000142224	ENSG00000181894	ENSG00000125107
ENSG00000173020	ENSG00000082196	ENSG00000161791
ENSG00000149636	ENSG00000167840	ENSG00000185372
ENSG00000167346	ENSG00000134001	ENSG00000204287
ENSG00000169413	ENSG00000116161	ENSG00000175728
ENSG00000143420	ENSG00000112339	ENSG00000074966
ENSG00000160226	ENSG00000133980	ENSG00000171611
ENSG00000137947	ENSG00000123572	ENSG00000100053
ENSG00000189190	ENSG00000197852	ENSG00000171812
ENSG00000141977	ENSG00000143570	ENSG00000174799
ENSG00000188396	ENSG00000177519	ENSG00000197604
ENSG00000100335	ENSG00000269858	ENSG00000184210
ENSG00000105245	ENSG00000167325	ENSG00000138794
ENSG00000135709	ENSG00000174100	ENSG00000152782
ENSG00000196226	ENSG00000085788	ENSG00000160188
ENSG00000150076	ENSG00000131477	ENSG00000132155
ENSG00000180902	ENSG00000164172	ENSG00000129965
ENSG00000125743	ENSG00000162551	ENSG00000116237
ENSG00000143248	ENSG00000130414	ENSG00000130783
ENSG00000197641	ENSG00000159650	ENSG00000145888
ENSG00000070081	ENSG00000144852	ENSG00000108262
ENSG00000117153	ENSG00000140632	ENSG00000180269
ENSG00000109854	ENSG00000160949	ENSG00000115226
ENSG00000137055	ENSG00000188488	ENSG00000106077
ENSG00000183145	ENSG00000204923	ENSG00000159753
ENSG00000196465	ENSG00000182985	ENSG00000160183
ENSG00000169228	ENSG00000127528	ENSG00000136240
ENSG00000119865	ENSG00000073008	ENSG00000184698
ENSG00000079459	ENSG00000099797	ENSG00000152595
ENSG00000251209	ENSG00000159131	ENSG00000133121
ENSG00000171209	ENSG00000175550	ENSG00000137942
ENSG00000186567	ENSG00000132300	ENSG00000105550
ENSG00000106244	ENSG00000171916	ENSG00000159055
ENSG00000088543	ENSG00000241962	ENSG00000198502
ENSG00000103226	ENSG00000186132	ENSG00000204524
ENSG00000168078	ENSG00000046604	ENSG00000254870
ENSG00000183340	ENSG00000101134	ENSG00000132478
ENSG00000169248	ENSG00000139131	ENSG00000109118
ENSG00000162654	ENSG00000138675	ENSG00000143546
ENSG00000166823	ENSG00000082146	ENSG00000181767
ENSG00000107223	ENSG00000139547	ENSG00000135222

ENSG00000221955	ENSG00000164512	ENSG00000171792
ENSG00000182173	ENSG00000180448	ENSG00000103342
ENSG00000106299	ENSG00000110066	ENSG00000137806
ENSG00000197885	ENSG00000155962	ENSG00000168566
ENSG00000148399	ENSG00000157911	ENSG00000100284
ENSG00000130427	ENSG00000140474	ENSG00000017260
ENSG00000185668	ENSG00000196353	ENSG00000256269
ENSG00000186395	ENSG00000102931	ENSG00000124731
ENSG00000114738	ENSG00000137825	ENSG00000146232
ENSG00000170348	ENSG00000091138	ENSG00000077721
ENSG00000105193	ENSG00000114354	ENSG00000164485
ENSG00000104883	ENSG00000101213	ENSG00000166579
ENSG00000123473	ENSG00000181803	ENSG00000115561
ENSG00000122566	ENSG00000124159	ENSG00000105290
ENSG00000013375	ENSG00000091640	ENSG00000172339
ENSG00000122884	ENSG00000169469	ENSG00000164695
ENSG00000108561	ENSG00000139220	ENSG00000137492
ENSG00000184227	ENSG00000251247	ENSG00000006695
ENSG00000099840	ENSG00000188549	ENSG00000189127
ENSG00000054116	ENSG00000012048	ENSG00000268178
ENSG00000196323	ENSG00000196275	ENSG00000134644
ENSG00000196532	ENSG00000173702	ENSG00000117400
ENSG00000158555	ENSG00000204385	ENSG00000126945
ENSG00000176853	ENSG00000151023	ENSG00000111665
ENSG00000139610	ENSG00000166224	ENSG00000242220
ENSG00000182810	ENSG00000141506	ENSG00000169242
ENSG00000087903	ENSG00000203863	ENSG00000114383
ENSG00000168438	ENSG00000165476	ENSG00000141076
ENSG00000124783	ENSG00000105854	ENSG00000186400
ENSG00000163600	ENSG00000100221	ENSG00000037474
ENSG00000162174	ENSG00000147257	ENSG00000112584
ENSG00000267740	ENSG00000175485	ENSG00000139890
ENSG00000187905	ENSG00000117090	ENSG00000121940
ENSG00000183734	ENSG00000100385	ENSG00000125975
ENSG00000111186	ENSG00000182154	ENSG00000230062
ENSG00000173681	ENSG00000101200	ENSG00000163687
ENSG00000141150	ENSG00000043591	ENSG00000214491
ENSG00000143612	ENSG00000144015	ENSG00000184047
ENSG00000090659	ENSG00000206483	ENSG00000222047
ENSG00000183807	ENSG00000100197	ENSG00000153002
ENSG00000213654	ENSG00000037637	ENSG00000182923
ENSG00000173728	ENSG00000148297	ENSG00000100796
ENSG00000066651	ENSG00000135211	ENSG00000258588
ENSG00000105612	ENSG00000140395	ENSG00000139218
ENSG00000185303	ENSG00000182566	ENSG00000267596
ENSG00000173846	ENSG00000119965	ENSG00000158109
ENSG00000175115	ENSG00000033011	ENSG00000136044
ENSG00000203326	ENSG00000110042	ENSG00000163898
ENSG00000087152	ENSG00000141052	ENSG00000102057
ENSG00000216937	ENSG00000213139	ENSG00000113163
ENSG00000184307	ENSG00000016490	ENSG00000221855
ENSG00000027869	ENSG00000266173	ENSG00000178567
ENSG00000013306	ENSG00000069535	ENSG00000131686
ENSG00000171766	ENSG00000184716	ENSG00000243649
ENSG00000182698	ENSG00000113194	ENSG00000169291
ENSG00000089063	ENSG00000169064	ENSG00000167553
ENSG00000110244	ENSG00000177551	ENSG00000082258
ENSG00000236637	ENSG00000179397	ENSG00000082556
ENSG00000070019	ENSG00000158850	ENSG00000143217
ENSG00000175793	ENSG00000242574	ENSG00000184730
ENSG00000129749	ENSG00000107140	ENSG00000132275
ENSG00000152242	ENSG00000163116	ENSG00000115310
ENSG00000124469	ENSG00000244362	ENSG00000170876
ENSG00000074266	ENSG00000149658	ENSG00000145861

ENSG00000131043	ENSG00000064270	ENSG00000149328
ENSG00000135124	ENSG00000111796	ENSG00000175718
ENSG00000167139	ENSG00000163032	ENSG00000198939
ENSG00000043355	ENSG00000168090	ENSG00000136834
ENSG00000177108	ENSG00000163530	ENSG00000164600
ENSG00000108588	ENSG00000139636	ENSG00000140650
ENSG00000221926	ENSG00000183508	ENSG00000167104
ENSG00000214941	ENSG00000103066	ENSG00000107566
ENSG00000130204	ENSG00000163347	ENSG00000151746
ENSG00000261272	ENSG00000145779	ENSG00000141084
ENSG00000198087	ENSG00000257923	ENSG00000144045
ENSG00000178882	ENSG00000197632	ENSG00000066135
ENSG00000121988	ENSG00000255737	ENSG00000151718
ENSG00000105971	ENSG00000149503	ENSG00000186153
ENSG00000116176	ENSG00000170027	ENSG00000011143
ENSG00000136280	ENSG00000176200	ENSG00000168918
ENSG00000258643	ENSG00000149124	ENSG00000040933
ENSG00000248385	ENSG00000124587	ENSG00000132703
ENSG00000162695	ENSG00000162231	ENSG00000132026
ENSG00000182853	ENSG00000164871	ENSG00000138802
ENSG00000203952	ENSG00000144827	ENSG00000198797
ENSG00000213588	ENSG00000185909	ENSG00000111652
ENSG00000122787	ENSG00000179950	ENSG00000166710
ENSG00000021574	ENSG00000106537	ENSG00000164066
ENSG00000143185	ENSG00000162520	ENSG00000154188
ENSG00000258227	ENSG00000242252	ENSG00000135090
ENSG00000105507	ENSG00000101384	ENSG00000203722
ENSG00000148498	ENSG00000248592	ENSG00000138434
ENSG00000249915	ENSG00000205636	ENSG00000165891
ENSG00000134440	ENSG00000197683	ENSG00000155629
ENSG00000136877	ENSG00000139865	ENSG00000197312
ENSG00000197208	ENSG00000187024	ENSG00000059915
ENSG00000197084	ENSG00000172409	ENSG00000084110
ENSG00000161888	ENSG00000181195	ENSG00000244694
ENSG00000107984	ENSG00000122033	ENSG00000125746
ENSG00000130881	ENSG00000064201	ENSG00000137124
ENSG00000256040	ENSG00000149548	ENSG00000109466
ENSG00000181939	ENSG00000119812	ENSG00000135776
ENSG00000174606	ENSG00000104852	ENSG00000196917
ENSG00000175868	ENSG00000184271	ENSG00000117519
ENSG00000013583	ENSG00000143797	ENSG00000139697
ENSG00000134046	ENSG00000078237	ENSG00000143333
ENSG00000248099	ENSG00000147123	ENSG00000153779
ENSG00000159123	ENSG00000169214	ENSG00000165219
ENSG00000244509	ENSG00000108861	ENSG00000091844
ENSG00000226777	ENSG00000197459	ENSG00000170482
ENSG00000108961	ENSG00000168214	ENSG00000139572
ENSG00000156795	ENSG00000005469	ENSG00000182255
ENSG00000132465	ENSG00000161642	ENSG00000242441
ENSG00000101407	ENSG00000163630	ENSG00000182583
ENSG00000060566	ENSG00000174521	ENSG00000187288
ENSG00000256500	ENSG00000007047	ENSG00000198650
ENSG00000082515	ENSG00000185940	ENSG00000171291
ENSG00000133275	ENSG00000145715	ENSG00000163736
ENSG00000174175	ENSG00000182919	ENSG00000080819
ENSG00000124532	ENSG00000124818	ENSG00000135114
ENSG00000219607	ENSG00000031003	ENSG00000204531
ENSG00000160948	ENSG00000081791	ENSG00000164619
ENSG00000105576	ENSG00000175063	ENSG00000086548
ENSG00000215545	ENSG00000080031	ENSG00000184481
ENSG00000164951	ENSG00000090612	ENSG00000135454
ENSG00000158716	ENSG00000131055	ENSG00000170619
ENSG00000204363	ENSG00000121774	ENSG00000151632
ENSG00000159761	ENSG00000182652	ENSG00000175928

ENSG00000125804	ENSG00000099625	ENSG00000184677
ENSG00000127152	ENSG00000091483	ENSG00000136824
ENSG00000111144	ENSG00000043143	ENSG00000255604
ENSG00000073067	ENSG00000177352	ENSG00000121101
ENSG00000132718	ENSG00000106648	ENSG00000111605
ENSG00000124767	ENSG00000182544	ENSG00000066279
ENSG00000164342	ENSG00000181523	ENSG00000164535
ENSG00000221996	ENSG00000224186	ENSG00000197591
ENSG00000169635	ENSG00000111877	ENSG00000185686
ENSG00000187545	ENSG00000133706	ENSG00000163362
ENSG00000185475	ENSG00000185339	ENSG00000144227
ENSG00000175003	ENSG00000120498	ENSG00000116981
ENSG00000099817	ENSG00000172731	ENSG00000109063
ENSG00000116675	ENSG00000009830	ENSG00000145349
ENSG00000249961	ENSG00000157168	ENSG00000137573
ENSG00000213297	ENSG00000137574	ENSG00000160961
ENSG00000118492	ENSG00000163933	ENSG00000132376
ENSG00000184635	ENSG00000244734	ENSG00000100012
ENSG00000143919	ENSG00000079739	ENSG00000091513
ENSG00000106302	ENSG00000110104	ENSG00000186792
ENSG00000167011	ENSG00000118518	ENSG00000141279
ENSG00000104312	ENSG00000008018	ENSG00000125207
ENSG00000168309	ENSG00000119900	ENSG00000136205
ENSG00000068366	ENSG00000157978	ENSG00000120658
ENSG00000175520	ENSG00000134463	ENSG00000172037
ENSG00000108602	ENSG00000214753	ENSG00000005194
ENSG00000189332	ENSG00000167646	ENSG00000182405
ENSG00000119632	ENSG00000165837	ENSG00000139626
ENSG00000213494	ENSG00000163956	ENSG00000164761
ENSG00000178403	ENSG00000101321	ENSG00000172179
ENSG00000125450	ENSG00000267059	ENSG00000118729
ENSG00000079215	ENSG00000123374	ENSG00000205221
ENSG00000128617	ENSG00000197914	ENSG00000198131
ENSG00000186280	ENSG00000185742	ENSG00000250641
ENSG00000106526	ENSG00000164749	ENSG00000162944
ENSG00000168067	ENSG00000131788	ENSG00000101670
ENSG00000172382	ENSG00000123610	ENSG00000166246
ENSG00000157578	ENSG00000149599	ENSG00000165807
ENSG00000268223	ENSG00000182621	ENSG00000178467
ENSG00000163666	ENSG00000146416	ENSG00000198718
ENSG00000189269	ENSG00000169118	ENSG00000020577
ENSG00000243660	ENSG00000128652	ENSG00000166734
ENSG00000185101	ENSG00000188334	ENSG00000111241
ENSG00000096060	ENSG00000120063	ENSG00000104133
ENSG00000149313	ENSG00000156599	ENSG00000165914
ENSG00000155368	ENSG00000125166	ENSG00000101017
ENSG00000182185	ENSG00000186889	ENSG00000134697
ENSG00000214300	ENSG00000183304	ENSG00000156042
ENSG00000197355	ENSG00000111203	ENSG00000165646
ENSG00000085433	ENSG00000104047	ENSG00000134183
ENSG00000184814	ENSG00000247746	ENSG00000187037
ENSG00000089280	ENSG00000130706	ENSG00000162664
ENSG00000196800	ENSG00000175166	ENSG00000259753
ENSG00000214510	ENSG00000106459	ENSG00000065325
ENSG00000165060	ENSG00000167767	ENSG00000266302
ENSG00000111875	ENSG00000187123	ENSG00000025800
ENSG00000153283	ENSG00000171865	ENSG00000250588
ENSG00000155115	ENSG00000142507	ENSG00000205937
ENSG00000182670	ENSG00000141424	ENSG00000196199
ENSG00000197056	ENSG00000155011	ENSG00000128510
ENSG00000120333	ENSG00000115568	ENSG00000145384
ENSG00000079785	ENSG00000206536	ENSG00000129518
ENSG00000137731	ENSG00000185761	ENSG00000145982
ENSG00000168216	ENSG00000087088	ENSG00000138356

ENSG00000235478	ENSG00000183695	ENSG00000163291
ENSG00000006075	ENSG00000185800	ENSG00000185437
ENSG00000184924	ENSG00000149488	ENSG00000167286
ENSG00000136636	ENSG00000162456	ENSG00000152580
ENSG00000105492	ENSG00000184058	ENSG00000162753
ENSG00000188706	ENSG00000013561	ENSG00000161649
ENSG00000100258	ENSG00000121743	ENSG00000204518
ENSG00000148341	ENSG00000170417	ENSG00000175137
ENSG00000164039	ENSG00000068394	ENSG00000196230
ENSG00000168653	ENSG00000134717	ENSG00000182606
ENSG00000216588	ENSG00000121075	ENSG00000172977
ENSG00000214934	ENSG00000196968	ENSG00000055130
ENSG00000186026	ENSG00000164049	ENSG00000188818
ENSG00000181903	ENSG00000121864	ENSG00000148396
ENSG00000257127	ENSG00000236334	ENSG00000160271
ENSG00000268404	ENSG00000186912	ENSG00000151117
ENSG00000146243	ENSG00000174226	ENSG00000196466
ENSG00000103160	ENSG00000099904	ENSG00000162063
ENSG00000198208	ENSG00000173714	ENSG00000106511
ENSG00000178591	ENSG00000089737	ENSG00000152620
ENSG00000174123	ENSG00000107164	ENSG00000148215
ENSG00000181371	ENSG00000204532	ENSG00000188828
ENSG00000188542	ENSG00000187912	ENSG00000117676
ENSG00000112167	ENSG00000173085	ENSG00000107959
ENSG00000114346	ENSG00000080511	ENSG00000165996
ENSG00000174327	ENSG00000147854	ENSG00000161265
ENSG00000135452	ENSG00000163803	ENSG00000169554
ENSG00000168461	ENSG00000101457	ENSG00000204392
ENSG00000120694	ENSG00000204446	ENSG00000110713
ENSG00000267261	ENSG00000214643	ENSG00000008083
ENSG00000152670	ENSG00000184009	ENSG00000169129
ENSG00000138068	ENSG00000119547	ENSG00000174353
ENSG00000243789	ENSG00000149531	ENSG00000131914
ENSG00000114388	ENSG00000172432	ENSG00000121957
ENSG00000124935	ENSG00000160973	ENSG00000185664
ENSG00000010030	ENSG00000090512	ENSG00000171004
ENSG00000099984	ENSG00000197520	ENSG00000067992
ENSG00000100372	ENSG00000185640	ENSG00000143845
ENSG00000172348	ENSG00000196936	ENSG00000120341
ENSG00000163577	ENSG00000172803	ENSG00000150054
ENSG00000177679	ENSG00000178789	ENSG00000087191
ENSG00000161911	ENSG00000131446	ENSG00000143442
ENSG00000148303	ENSG00000186654	ENSG00000083814
ENSG00000213337	ENSG00000035687	ENSG00000130311
ENSG00000114391	ENSG00000164647	ENSG00000197977
ENSG00000103257	ENSG00000104055	ENSG00000082293
ENSG00000186130	ENSG00000257138	ENSG00000119414
ENSG00000036257	ENSG00000105889	ENSG00000096395
ENSG00000103275	ENSG00000147649	ENSG00000170807
ENSG00000206069	ENSG00000183161	ENSG00000223865
ENSG00000164182	ENSG00000240224	ENSG00000175455
ENSG00000127252	ENSG00000175170	ENSG00000143552
ENSG00000145075	ENSG00000160741	ENSG00000198877
ENSG00000138722	ENSG00000156642	ENSG00000178084
ENSG00000149451	ENSG00000186260	ENSG00000178234
ENSG00000216560	ENSG00000183287	ENSG00000146383
ENSG00000168679	ENSG00000147041	ENSG00000140563
ENSG00000143340	ENSG00000134489	ENSG00000181785
ENSG00000131876	ENSG00000180219	ENSG00000104341
ENSG00000138442	ENSG00000145912	ENSG00000148541
ENSG00000173976	ENSG00000184207	ENSG00000123485
ENSG00000014257	ENSG00000079150	ENSG00000109184
ENSG00000040608	ENSG00000074696	ENSG00000155893
ENSG00000197261	ENSG00000018236	ENSG00000091664

ENSG00000162383	ENSG00000256018	ENSG00000159346
ENSG00000122873	ENSG00000158423	ENSG00000105993
ENSG00000196778	ENSG00000112200	ENSG00000109790
ENSG00000184140	ENSG00000169718	ENSG00000080618
ENSG00000112367	ENSG00000100991	ENSG00000026950
ENSG00000254607	ENSG00000115041	ENSG00000064703
ENSG00000174417	ENSG00000213199	ENSG00000177721
ENSG00000151135	ENSG00000102710	ENSG00000221938
ENSG00000167194	ENSG00000165973	ENSG00000172828
ENSG00000123178	ENSG00000101180	ENSG00000176907
ENSG00000166562	ENSG00000067113	ENSG00000141252
ENSG00000156127	ENSG00000188763	ENSG00000103522
ENSG00000184530	ENSG00000177238	ENSG00000140557
ENSG00000181381	ENSG00000172936	ENSG00000143627
ENSG00000183114	ENSG00000006576	ENSG00000163814
ENSG00000179604	ENSG00000170537	ENSG00000213096
ENSG00000100897	ENSG00000131061	ENSG00000162727
ENSG00000135775	ENSG00000102921	ENSG00000096093
ENSG00000261701	ENSG00000170265	ENSG00000140265
ENSG00000153291	ENSG00000078399	ENSG00000139370
ENSG00000130433	ENSG00000163909	ENSG00000136527
ENSG00000155508	ENSG00000126698	ENSG00000006704
ENSG00000108622	ENSG00000243955	ENSG00000111696
ENSG00000037042	ENSG00000077147	ENSG00000126368
ENSG00000046653	ENSG00000140525	ENSG00000172869
ENSG00000176798	ENSG00000142544	ENSG00000145029
ENSG00000146955	ENSG00000189068	ENSG00000142619
ENSG00000086598	ENSG00000154328	ENSG00000105127
ENSG00000106462	ENSG00000168229	ENSG00000143228
ENSG00000169241	ENSG00000189369	ENSG00000044446
ENSG00000240849	ENSG00000150628	ENSG00000089012
ENSG00000177707	ENSG00000147443	ENSG00000154229
ENSG00000160058	ENSG00000160284	ENSG00000253831
ENSG00000115275	ENSG00000172336	ENSG00000154099
ENSG00000111913	ENSG00000036672	ENSG00000089847
ENSG00000160404	ENSG00000123444	ENSG00000186185
ENSG00000080293	ENSG00000180964	ENSG00000115919
ENSG00000166226	ENSG00000111596	ENSG00000169035
ENSG00000174437	ENSG00000143418	ENSG00000187054
ENSG00000108786	ENSG00000269048	ENSG00000136144
ENSG00000186715	ENSG00000152254	ENSG00000111331
ENSG00000107625	ENSG00000269369	ENSG00000186866
ENSG00000151687	ENSG00000108639	ENSG00000133961
ENSG00000086289	ENSG00000118246	ENSG00000198785
ENSG00000121848	ENSG00000176034	ENSG00000122482
ENSG00000236609	ENSG00000237452	ENSG00000155833
ENSG00000162444	ENSG00000153487	ENSG00000083307
ENSG00000214929	ENSG00000116752	ENSG00000187260
ENSG00000163508	ENSG00000137817	ENSG00000111641
ENSG00000054803	ENSG00000204508	ENSG00000262576
ENSG00000115233	ENSG00000153208	ENSG00000205978
ENSG00000128581	ENSG00000175564	ENSG00000243251
ENSG00000198242	ENSG00000187516	ENSG00000185652
ENSG00000121741	ENSG00000189051	ENSG00000115556
ENSG00000258365	ENSG00000159251	ENSG00000126777
ENSG00000152952	ENSG00000205364	ENSG00000173852
ENSG00000113905	ENSG00000204070	ENSG00000008441
ENSG00000120211	ENSG00000154743	ENSG00000112679
ENSG00000164287	ENSG00000177427	ENSG00000070413
ENSG00000130733	ENSG00000164933	ENSG00000123080
ENSG00000137807	ENSG00000168158	ENSG00000179546
ENSG00000212950	ENSG00000102547	ENSG00000153558
ENSG00000084234	ENSG00000161544	ENSG00000197362
ENSG00000136960	ENSG00000115163	ENSG00000019186

ENSG00000267270	ENSG00000036549	ENSG00000070159
ENSG00000169306	ENSG00000135094	ENSG00000089692
ENSG00000179846	ENSG00000167580	ENSG00000119403
ENSG00000136888	ENSG00000115307	ENSG00000197594
ENSG00000117318	ENSG00000175931	ENSG00000100603
ENSG00000158158	ENSG00000196460	ENSG00000170430
ENSG00000183401	ENSG00000147894	ENSG00000137876
ENSG00000121388	ENSG00000184014	ENSG00000163312
ENSG00000159423	ENSG00000183570	ENSG00000186442
ENSG00000058085	ENSG00000133710	ENSG00000064655
ENSG00000132746	ENSG00000112079	ENSG00000135569
ENSG00000170689	ENSG00000131828	ENSG00000058056
ENSG00000123094	ENSG00000178913	ENSG00000180398
ENSG00000114013	ENSG00000148965	ENSG00000124721
ENSG00000255994	ENSG00000168101	ENSG00000187715
ENSG00000077585	ENSG00000185436	ENSG00000176895
ENSG00000106615	ENSG00000203896	ENSG00000172006
ENSG00000110911	ENSG00000137962	ENSG00000137133
ENSG00000108556	ENSG00000125651	ENSG00000171428
ENSG00000184343	ENSG00000198889	ENSG00000172819
ENSG00000181784	ENSG00000170631	ENSG00000171747
ENSG00000092439	ENSG00000103966	ENSG00000118705
ENSG00000127220	ENSG00000065978	ENSG00000113368
ENSG00000072609	ENSG00000110448	ENSG00000175535
ENSG00000158195	ENSG00000113369	ENSG00000173517
ENSG00000164024	ENSG00000110455	ENSG00000134765
ENSG00000198685	ENSG00000066926	ENSG00000092529
ENSG00000185236	ENSG00000120265	ENSG00000101052
ENSG00000168404	ENSG00000135999	ENSG00000156345
ENSG00000180530	ENSG00000119487	ENSG00000196734
ENSG00000143183	ENSG00000131721	ENSG00000172296
ENSG00000088726	ENSG00000167103	ENSG00000136451
ENSG00000171984	ENSG00000030419	ENSG00000183035
ENSG00000145824	ENSG00000075213	ENSG00000171617
ENSG00000162415	ENSG00000110777	ENSG00000203924
ENSG00000129521	ENSG00000143321	ENSG00000102221
ENSG00000197256	ENSG00000169813	ENSG00000064787
ENSG00000184277	ENSG00000119718	ENSG00000042088
ENSG00000142910	ENSG00000102738	ENSG00000159788
ENSG00000124157	ENSG00000077238	ENSG00000135355
ENSG00000163655	ENSG00000164756	ENSG00000080224
ENSG00000163539	ENSG00000013810	ENSG00000143569
ENSG00000188620	ENSG00000104228	ENSG00000104324
ENSG00000188163	ENSG00000082074	ENSG00000010318
ENSG00000131871	ENSG00000179941	ENSG00000110107
ENSG00000101782	ENSG00000186777	ENSG00000156603
ENSG00000185024	ENSG00000205328	ENSG00000178401
ENSG00000146678	ENSG00000124440	ENSG00000100462
ENSG00000089091	ENSG00000166012	ENSG00000185753
ENSG00000178795	ENSG00000172150	ENSG00000131264
ENSG00000126561	ENSG00000113763	ENSG00000150093
ENSG00000075914	ENSG00000130038	ENSG00000168610
ENSG00000213265	ENSG00000160678	ENSG00000183269
ENSG00000171867	ENSG00000134504	ENSG00000102145
ENSG00000163958	ENSG00000110063	ENSG00000126746
ENSG00000116138	ENSG00000163904	ENSG00000078487
ENSG00000075303	ENSG00000186847	ENSG00000086570
ENSG00000215695	ENSG00000163714	ENSG00000160870
ENSG00000139835	ENSG00000160307	ENSG00000105176
ENSG00000166930	ENSG00000124225	ENSG00000119608
ENSG00000227011	ENSG00000100036	ENSG00000198099
ENSG00000116667	ENSG00000106258	ENSG00000146166
ENSG00000149179	ENSG00000051108	ENSG00000100884
ENSG00000170819	ENSG00000106018	ENSG00000197674

ENSG00000171484	ENSG00000250067	ENSG00000123165
ENSG00000171773	ENSG00000179029	ENSG00000108296
ENSG00000157131	ENSG00000129009	ENSG00000143079
ENSG00000231396	ENSG00000136805	ENSG00000154059
ENSG00000169019	ENSG00000169507	ENSG00000128710
ENSG00000198271	ENSG00000105063	ENSG00000176531
ENSG00000168795	ENSG00000221887	ENSG00000124614
ENSG00000141522	ENSG00000102908	ENSG00000221838
ENSG00000092200	ENSG00000090447	ENSG00000083635
ENSG00000111676	ENSG00000164125	ENSG00000228567
ENSG00000261147	ENSG00000135953	ENSG00000166340
ENSG00000181019	ENSG00000115221	ENSG00000103534
ENSG00000117691	ENSG00000167771	ENSG00000205126
ENSG00000122545	ENSG00000138629	ENSG00000172728
ENSG00000006074	ENSG00000168491	ENSG00000172362
ENSG00000126012	ENSG00000119421	ENSG00000159658
ENSG00000168092	ENSG00000188869	ENSG00000118503
ENSG00000196533	ENSG00000258986	ENSG00000137198
ENSG00000255046	ENSG00000181754	ENSG00000084710
ENSG00000115520	ENSG00000115020	ENSG00000203837
ENSG00000198604	ENSG00000130173	ENSG00000065675
ENSG00000162729	ENSG00000151465	ENSG00000168310
ENSG00000109758	ENSG00000169299	ENSG00000094631
ENSG00000110583	ENSG00000219016	ENSG00000112116
ENSG00000137745	ENSG00000077498	ENSG00000152527
ENSG00000105649	ENSG00000135862	ENSG00000151552
ENSG00000063046	ENSG00000135414	ENSG00000140092
ENSG00000166503	ENSG00000121446	ENSG00000070770
ENSG00000175600	ENSG00000108469	ENSG00000128536
ENSG00000122862	ENSG00000115592	ENSG00000121210
ENSG00000153391	ENSG00000129158	ENSG00000101327
ENSG00000187416	ENSG00000221990	ENSG00000145832
ENSG00000184486	ENSG00000105483	ENSG00000103319
ENSG00000188234	ENSG00000139154	ENSG00000099204
ENSG00000159217	ENSG00000102572	ENSG00000111537
ENSG00000005249	ENSG00000256206	ENSG00000066697
ENSG00000101307	ENSG00000163848	ENSG00000188158
ENSG00000214530	ENSG00000213471	ENSG00000090889
ENSG00000146701	ENSG00000174982	ENSG00000123342
ENSG00000068028	ENSG00000178645	ENSG00000099834
ENSG00000197889	ENSG00000141979	ENSG00000168014
ENSG00000104313	ENSG00000039650	ENSG00000139132
ENSG00000153975	ENSG00000153815	ENSG00000244474
ENSG00000135540	ENSG00000180304	ENSG00000148090
ENSG00000183208	ENSG00000178425	ENSG00000163040
ENSG00000090530	ENSG00000115263	ENSG00000130338
ENSG00000205018	ENSG00000257599	ENSG00000204021
ENSG00000204403	ENSG00000069869	ENSG00000151726
ENSG00000183092	ENSG00000112473	ENSG00000169548
ENSG00000169583	ENSG00000131773	ENSG00000086991
ENSG00000178531	ENSG00000167371	ENSG00000087365
ENSG00000005102	ENSG00000204348	ENSG00000147650
ENSG00000260175	ENSG00000197343	ENSG00000049089
ENSG00000221852	ENSG00000133318	ENSG00000151025
ENSG00000139112	ENSG00000182035	ENSG00000050327
ENSG00000177374	ENSG00000132424	ENSG00000102445
ENSG00000196544	ENSG00000136541	ENSG00000183748
ENSG00000034677	ENSG00000244588	ENSG00000078081
ENSG00000116678	ENSG00000133619	ENSG00000175215
ENSG00000101405	ENSG00000087302	ENSG00000130695
ENSG00000253857	ENSG00000165322	ENSG00000166828
ENSG00000165388	ENSG00000158856	ENSG00000143367
ENSG00000171148	ENSG00000141576	ENSG00000163624
ENSG00000205581	ENSG00000167360	ENSG00000239590

ENSG00000184206	ENSG00000243449	ENSG00000157483
ENSG00000125817	ENSG00000155100	ENSG00000126705
ENSG00000133597	ENSG00000163660	ENSG00000140945
ENSG00000167195	ENSG00000110436	ENSG00000070047
ENSG00000245680	ENSG00000165556	ENSG00000107281
ENSG00000061794	ENSG00000108219	ENSG00000122359
ENSG00000244623	ENSG00000170464	ENSG00000102974
ENSG00000163682	ENSG00000163517	ENSG00000198203
ENSG00000167384	ENSG00000130201	ENSG00000138075
ENSG00000163001	ENSG00000143337	ENSG00000151892
ENSG00000111897	ENSG00000121314	ENSG00000185787
ENSG00000144182	ENSG00000106341	ENSG00000164100
ENSG00000138180	ENSG00000145321	ENSG00000176742
ENSG00000102891	ENSG00000129245	ENSG00000159455
ENSG00000154655	ENSG00000153822	ENSG00000142515
ENSG00000213551	ENSG00000197958	ENSG00000004660
ENSG00000180185	ENSG00000086827	ENSG00000225697
ENSG00000166128	ENSG00000115216	ENSG00000147457
ENSG00000137834	ENSG00000170624	ENSG00000165568
ENSG00000135931	ENSG00000196357	ENSG00000178921
ENSG00000115286	ENSG00000163631	ENSG00000138468
ENSG00000143156	ENSG00000126822	ENSG00000164080
ENSG00000186092	ENSG00000164187	ENSG00000198366
ENSG00000120159	ENSG00000169139	ENSG00000162763
ENSG00000171320	ENSG00000229859	ENSG00000258724
ENSG00000169783	ENSG00000154518	ENSG00000138617
ENSG00000107331	ENSG00000178562	ENSG00000025434
ENSG00000127585	ENSG00000100276	ENSG00000141736
ENSG00000075336	ENSG00000006118	ENSG00000106078
ENSG00000103769	ENSG00000134852	ENSG00000135069
ENSG00000118972	ENSG00000169605	ENSG00000255800
ENSG00000148153	ENSG00000124232	ENSG00000172772
ENSG00000131910	ENSG00000169136	ENSG00000157601
ENSG00000203951	ENSG00000092098	ENSG00000136950
ENSG00000135914	ENSG00000118997	ENSG00000169372
ENSG00000189052	ENSG00000186924	ENSG00000056998
ENSG00000158552	ENSG00000153767	ENSG00000102081
ENSG00000140157	ENSG00000102053	ENSG00000078898
ENSG00000103657	ENSG00000165972	ENSG00000100201
ENSG00000213465	ENSG00000149927	ENSG00000100228
ENSG00000114204	ENSG00000108963	ENSG00000196177
ENSG00000185222	ENSG00000186825	ENSG00000131016
ENSG00000132341	ENSG00000003147	ENSG00000166578
ENSG00000204599	ENSG00000181585	ENSG00000103642
ENSG00000182257	ENSG00000152022	ENSG00000176567
ENSG00000188312	ENSG00000140750	ENSG00000148948
ENSG00000167792	ENSG00000110844	ENSG00000179218
ENSG00000160951	ENSG00000070718	ENSG00000198429
ENSG00000118579	ENSG00000112576	ENSG00000075151
ENSG00000129255	ENSG00000184724	ENSG00000112697
ENSG00000174946	ENSG00000188997	ENSG00000179262
ENSG00000134222	ENSG00000188581	ENSG00000176393
ENSG00000124568	ENSG00000092847	ENSG00000113851
ENSG00000185187	ENSG00000124459	ENSG00000204195
ENSG00000160972	ENSG00000159479	ENSG00000181991
ENSG00000107175	ENSG00000102977	ENSG00000140876
ENSG00000136738	ENSG00000092969	ENSG00000151229
ENSG00000047249	ENSG00000260916	ENSG00000011376
ENSG00000197658	ENSG00000104804	ENSG00000148057
ENSG00000139800	ENSG00000176678	ENSG00000102904
ENSG00000118655	ENSG00000180913	ENSG00000142675
ENSG00000183628	ENSG00000184900	ENSG00000029363
ENSG00000198931	ENSG00000133858	ENSG00000146476
ENSG00000214285	ENSG00000232268	ENSG00000143258

ENSG00000242485	ENSG00000185551	ENSG00000180090
ENSG00000166888	ENSG00000169288	ENSG00000130254
ENSG00000132881	ENSG00000156273	ENSG00000012822
ENSG00000224689	ENSG00000188822	ENSG00000137265
ENSG00000154582	ENSG00000130520	ENSG00000100982
ENSG00000154845	ENSG00000185090	ENSG00000198822
ENSG00000108590	ENSG00000137965	ENSG00000163394
ENSG00000135828	ENSG00000125912	ENSG00000176198
ENSG00000136870	ENSG00000164929	ENSG00000166762
ENSG00000248483	ENSG00000181609	ENSG00000145192
ENSG00000110880	ENSG00000154016	ENSG00000213218
ENSG00000243444	ENSG00000205212	ENSG00000166471
ENSG00000164054	ENSG00000188124	ENSG00000256591
ENSG00000182132	ENSG00000185245	ENSG00000171115
ENSG00000130950	ENSG00000158714	ENSG00000196678
ENSG00000244624	ENSG00000196092	ENSG00000067208
ENSG00000165244	ENSG00000169972	ENSG00000177302
ENSG00000128654	ENSG00000101353	ENSG00000155980
ENSG00000099246	ENSG00000168928	ENSG00000065135
ENSG00000155875	ENSG00000118260	ENSG00000134070
ENSG00000003056	ENSG00000164576	ENSG00000144381
ENSG00000153214	ENSG00000221826	ENSG00000134987
ENSG00000173327	ENSG00000203737	ENSG00000153230
ENSG00000242550	ENSG00000114631	ENSG00000120160
ENSG00000206203	ENSG00000188883	ENSG00000119927
ENSG00000151748	ENSG00000185246	ENSG00000113645
ENSG00000186665	ENSG00000144895	ENSG00000166046
ENSG00000206159	ENSG00000197786	ENSG00000169131
ENSG00000204703	ENSG00000183833	ENSG00000009765
ENSG00000141096	ENSG00000165915	ENSG00000100558
ENSG00000165449	ENSG00000085117	ENSG00000165643
ENSG00000204614	ENSG00000140262	ENSG00000174485
ENSG00000147164	ENSG00000171877	ENSG00000174963
ENSG00000173338	ENSG00000170049	ENSG00000188508
ENSG00000203797	ENSG00000100418	ENSG00000102302
ENSG00000130024	ENSG00000147166	ENSG00000166091
ENSG00000160932	ENSG00000184674	ENSG00000174473
ENSG00000057608	ENSG00000143013	ENSG00000122420
ENSG00000100697	ENSG00000101204	ENSG00000214102
ENSG00000163520	ENSG00000187980	ENSG00000181001
ENSG00000158869	ENSG00000170891	ENSG00000137726
ENSG00000213995	ENSG00000198467	ENSG00000152457
ENSG00000165115	ENSG00000108091	ENSG00000144554
ENSG00000242284	ENSG00000162144	ENSG00000163171
ENSG00000161533	ENSG00000180720	ENSG00000187726
ENSG00000204822	ENSG00000172379	ENSG00000163798
ENSG00000105983	ENSG00000109339	ENSG00000132321
ENSG00000204616	ENSG00000183760	ENSG00000090905
ENSG00000166439	ENSG00000146352	ENSG00000112159
ENSG00000152430	ENSG00000174842	ENSG00000064225
ENSG00000263203	ENSG00000196313	ENSG00000182983
ENSG00000148834	ENSG00000141582	ENSG00000221813
ENSG00000205352	ENSG00000140009	ENSG00000167380
ENSG00000244187	ENSG00000164344	ENSG00000250741
ENSG00000196209	ENSG00000122477	ENSG00000182872
ENSG00000147036	ENSG00000101342	ENSG00000221937
ENSG00000167107	ENSG00000162086	ENSG00000116824
ENSG00000186908	ENSG00000185522	ENSG00000175322
ENSG00000108465	ENSG00000129474	ENSG00000164663
ENSG00000131475	ENSG00000114019	ENSG00000124570
ENSG00000168350	ENSG00000139651	ENSG00000156261
ENSG00000168903	ENSG00000184270	ENSG00000111666
ENSG00000237440	ENSG00000174306	ENSG00000119285
ENSG00000152518	ENSG00000153786	ENSG00000155287

ENSG00000081041	ENSG00000141179	ENSG00000196966
ENSG00000197437	ENSG00000104856	ENSG00000184060
ENSG00000214265	ENSG00000132535	ENSG00000136247
ENSG00000132749	ENSG00000267970	ENSG00000135740
ENSG00000088930	ENSG00000125611	ENSG00000256294
ENSG00000170088	ENSG00000242802	ENSG00000108753
ENSG00000253506	ENSG00000158516	ENSG00000173992
ENSG00000178386	ENSG00000111802	ENSG00000132199
ENSG00000189320	ENSG00000104970	ENSG00000169896
ENSG00000136819	ENSG00000120217	ENSG00000179889
ENSG00000183621	ENSG00000004961	ENSG00000213401
ENSG00000089693	ENSG00000174358	ENSG00000125124
ENSG00000163344	ENSG00000203711	ENSG00000170242
ENSG00000149474	ENSG00000120519	ENSG00000183206
ENSG00000184925	ENSG00000141622	ENSG00000116721
ENSG00000111481	ENSG00000125877	ENSG00000123240
ENSG00000101654	ENSG00000105879	ENSG00000138587
ENSG00000150768	ENSG00000204209	ENSG00000123307
ENSG00000107882	ENSG00000128641	ENSG00000138670
ENSG00000135821	ENSG00000101981	ENSG00000134480
ENSG00000165806	ENSG00000118922	ENSG00000079277
ENSG00000184988	ENSG00000064205	ENSG00000186943
ENSG00000164778	ENSG00000187051	ENSG00000013619
ENSG00000134539	ENSG00000156256	ENSG00000165349
ENSG00000180884	ENSG00000153495	ENSG00000125864
ENSG00000054282	ENSG00000112667	ENSG00000164283
ENSG00000133818	ENSG00000214732	ENSG00000167910
ENSG00000100234	ENSG00000116132	ENSG00000225830
ENSG00000173511	ENSG00000255181	ENSG00000121236
ENSG00000162650	ENSG00000088247	ENSG00000122735
ENSG00000228198	ENSG00000102024	ENSG00000088386
ENSG00000111907	ENSG00000164300	ENSG00000107789
ENSG00000160679	ENSG00000143416	ENSG00000023171
ENSG00000163515	ENSG00000011465	ENSG00000018408
ENSG00000180697	ENSG00000111142	ENSG00000172487
ENSG00000205085	ENSG00000086159	ENSG00000125834
ENSG00000077009	ENSG00000173124	ENSG00000171777
ENSG00000116786	ENSG00000161904	ENSG00000162391
ENSG00000162614	ENSG00000160602	ENSG00000154914
ENSG00000154473	ENSG00000151006	ENSG00000072315
ENSG00000112763	ENSG00000106524	ENSG00000111701
ENSG00000170122	ENSG00000204347	ENSG00000171056
ENSG00000028137	ENSG00000136250	ENSG00000100060
ENSG00000117560	ENSG00000185634	ENSG00000170961
ENSG00000139350	ENSG00000163947	ENSG00000179456
ENSG00000163633	ENSG00000106771	ENSG00000167554
ENSG00000183379	ENSG00000117643	ENSG00000117425
ENSG00000177459	ENSG00000101850	ENSG00000115841
ENSG00000166573	ENSG00000143924	ENSG00000088035
ENSG00000184357	ENSG00000072135	ENSG00000124091
ENSG00000100410	ENSG00000215244	ENSG00000118520
ENSG00000205981	ENSG00000139289	ENSG00000152779
ENSG00000167797	ENSG00000151729	ENSG00000154114
ENSG00000243335	ENSG00000198408	ENSG00000185010
ENSG00000158786	ENSG00000241186	ENSG00000171459
ENSG00000100350	ENSG00000108443	ENSG00000104419
ENSG00000151093	ENSG00000230453	ENSG00000105146
ENSG00000189129	ENSG00000099219	ENSG00000136839
ENSG00000141934	ENSG00000046651	ENSG00000151148
ENSG00000156958	ENSG00000162777	ENSG00000198851
ENSG00000183154	ENSG00000117971	ENSG00000176732
ENSG00000101751	ENSG00000100362	ENSG00000162885
ENSG00000184788	ENSG00000213339	ENSG00000186190
ENSG00000164451	ENSG00000160799	ENSG00000161654

ENSG00000125898	ENSG00000170166	ENSG00000132970
ENSG00000164935	ENSG00000100429	ENSG00000174562
ENSG00000269343	ENSG00000175463	ENSG00000170160
ENSG00000105011	ENSG00000149499	ENSG00000175497
ENSG00000101435	ENSG00000185008	ENSG00000140519
ENSG00000148356	ENSG00000183066	ENSG00000197706
ENSG00000108774	ENSG00000168925	ENSG00000116218
ENSG00000142959	ENSG00000127328	ENSG00000152705
ENSG00000241233	ENSG00000124155	ENSG00000070061
ENSG00000256453	ENSG00000196150	ENSG00000001626
ENSG00000181284	ENSG00000176973	ENSG00000100426
ENSG00000253206	ENSG00000174595	ENSG00000182718
ENSG00000187242	ENSG00000105671	ENSG00000261949
ENSG00000095464	ENSG00000197403	ENSG00000117625
ENSG00000168255	ENSG00000172661	ENSG00000138796
ENSG00000103671	ENSG00000174611	ENSG00000166681
ENSG00000162881	ENSG00000214694	ENSG00000170633
ENSG00000008988	ENSG00000160221	ENSG00000157379
ENSG00000254614	ENSG00000132002	ENSG00000144837
ENSG00000104979	ENSG00000166394	ENSG00000187173
ENSG00000148450	ENSG00000143537	ENSG00000127928
ENSG00000160181	ENSG00000127419	ENSG00000115594
ENSG00000160844	ENSG00000112144	ENSG00000091436
ENSG00000148734	ENSG00000159388	ENSG00000139445
ENSG00000162923	ENSG00000169964	ENSG00000169594
ENSG00000196639	ENSG00000136514	ENSG00000109099
ENSG00000164032	ENSG00000164305	ENSG00000186300
ENSG00000065308	ENSG00000173889	ENSG00000198833
ENSG00000176428	ENSG00000026508	ENSG00000107937
ENSG00000041357	ENSG00000174943	ENSG00000183955
ENSG00000136141	ENSG00000107438	ENSG00000150991
ENSG00000169021	ENSG00000128699	ENSG00000196565
ENSG00000142546	ENSG00000197696	ENSG00000119723
ENSG00000129911	ENSG00000128513	ENSG00000198585
ENSG00000169442	ENSG00000129167	ENSG00000123836
ENSG00000124194	ENSG00000115652	ENSG00000134278
ENSG00000186104	ENSG00000197123	ENSG00000141437
ENSG00000180758	ENSG00000167419	ENSG00000065609
ENSG00000175832	ENSG00000204381	ENSG00000132326
ENSG00000158106	ENSG00000112078	ENSG00000068323
ENSG00000124795	ENSG00000146776	ENSG00000100116
ENSG00000161570	ENSG00000163161	ENSG00000214711
ENSG00000172183	ENSG00000168724	ENSG00000111110
ENSG00000241404	ENSG00000197249	ENSG00000107890
ENSG00000205765	ENSG00000213231	ENSG00000119599
ENSG00000167100	ENSG00000178538	ENSG00000186364
ENSG00000159516	ENSG00000215018	ENSG00000085491
ENSG00000205426	ENSG00000135766	ENSG00000186583
ENSG00000008323	ENSG00000179299	ENSG00000169154
ENSG00000197063	ENSG00000118557	ENSG00000173258
ENSG00000122133	ENSG00000156467	ENSG00000137275
ENSG00000198146	ENSG00000103089	ENSG00000168781
ENSG00000170965	ENSG00000159023	ENSG00000101290
ENSG00000144535	ENSG00000162009	ENSG00000189001
ENSG00000125618	ENSG00000006638	ENSG00000197746
ENSG00000139531	ENSG00000164051	ENSG00000029153
ENSG00000196878	ENSG00000171729	ENSG00000007372
ENSG00000250361	ENSG00000205884	ENSG00000185917
ENSG00000124659	ENSG00000125731	ENSG00000177947
ENSG00000169509	ENSG00000186160	ENSG00000101868
ENSG00000117118	ENSG00000138613	ENSG00000070785
ENSG00000115866	ENSG00000134690	ENSG00000112337
ENSG00000090565	ENSG00000076716	ENSG00000135052
ENSG00000186469	ENSG00000172890	ENSG00000109775

ENSG00000129048	ENSG00000172927	ENSG00000109654
ENSG00000111328	ENSG00000118523	ENSG00000160753
ENSG00000163263	ENSG00000164105	ENSG00000140987
ENSG00000212807	ENSG00000176029	ENSG00000101812
ENSG00000255472	ENSG00000179912	ENSG00000183077
ENSG00000206262	ENSG00000067369	ENSG00000115806
ENSG00000012660	ENSG00000174957	ENSG00000244607
ENSG00000072310	ENSG00000124222	ENSG00000144410
ENSG00000102901	ENSG00000104626	ENSG00000163297
ENSG00000205783	ENSG00000108010	ENSG00000104973
ENSG00000241258	ENSG00000187456	ENSG00000198919
ENSG00000157540	ENSG00000179826	ENSG00000166351
ENSG00000112214	ENSG00000152642	ENSG00000180626
ENSG00000102580	ENSG00000183598	ENSG00000152086
ENSG00000068001	ENSG00000162398	ENSG00000146378
ENSG00000091317	ENSG00000186007	ENSG00000120280
ENSG00000025156	ENSG00000155755	ENSG00000105989
ENSG00000136379	ENSG00000214324	ENSG00000137310
ENSG00000166268	ENSG00000107949	ENSG00000169660
ENSG00000164185	ENSG00000162076	ENSG00000166311
ENSG00000113088	ENSG00000186090	ENSG00000111339
ENSG00000084072	ENSG00000184005	ENSG00000050405
ENSG00000142615	ENSG00000147869	ENSG00000067191
ENSG00000203985	ENSG00000131115	ENSG00000010671
ENSG00000205323	ENSG00000185739	ENSG00000146112
ENSG00000136560	ENSG00000156990	ENSG00000088682
ENSG00000224821	ENSG00000116661	ENSG00000105855
ENSG00000150045	ENSG00000179085	ENSG00000176903
ENSG00000181315	ENSG00000165863	ENSG00000131981
ENSG00000198590	ENSG00000188379	ENSG00000099810
ENSG00000108960	ENSG00000101104	ENSG00000161609
ENSG00000136731	ENSG00000178951	ENSG00000116117
ENSG00000148444	ENSG00000180875	ENSG00000183778
ENSG00000175606	ENSG00000102763	ENSG00000198759
ENSG00000157954	ENSG00000138172	ENSG00000198315
ENSG00000163155	ENSG00000188130	ENSG00000166704
ENSG00000179837	ENSG00000176946	ENSG00000077420
ENSG00000212670	ENSG00000130821	ENSG00000121895
ENSG00000185479	ENSG00000183479	ENSG00000170465
ENSG00000204316	ENSG00000100121	ENSG00000167566
ENSG00000158483	ENSG00000147862	ENSG00000186532
ENSG00000132600	ENSG00000177606	ENSG00000158816
ENSG00000214087	ENSG00000014641	ENSG00000110881
ENSG00000004059	ENSG00000184828	ENSG00000027697
ENSG00000162817	ENSG00000147180	ENSG00000079819
ENSG00000106305	ENSG00000120436	ENSG00000010278
ENSG00000149925	ENSG00000188277	ENSG00000141034
ENSG00000137770	ENSG00000066654	ENSG00000155495
ENSG00000265590	ENSG00000117461	ENSG00000114745
ENSG00000185379	ENSG00000081800	ENSG00000131732
ENSG00000269011	ENSG00000118046	ENSG00000100029
ENSG00000135702	ENSG00000105229	ENSG00000165269
ENSG00000124615	ENSG00000124299	ENSG00000172426
ENSG00000054148	ENSG00000100412	ENSG00000204687
ENSG00000206474	ENSG00000115421	ENSG00000243978
ENSG00000157184	ENSG00000158428	ENSG00000137413
ENSG00000166130	ENSG00000172466	ENSG00000169744
ENSG00000083642	ENSG00000184350	ENSG00000144278
ENSG00000135049	ENSG00000057252	ENSG00000083817
ENSG00000162409	ENSG00000047648	ENSG00000164548
ENSG00000164252	ENSG00000005100	ENSG00000197111
ENSG00000115875	ENSG00000143512	ENSG00000103423
ENSG00000121351	ENSG00000165078	ENSG00000188404
ENSG00000042493	ENSG00000138780	ENSG00000165995

ENSG00000253710	ENSG00000112306	ENSG00000103091
ENSG00000152348	ENSG00000186474	ENSG00000125354
ENSG00000206013	ENSG00000162972	ENSG00000175189
ENSG00000112406	ENSG00000154153	ENSG00000137203
ENSG00000138136	ENSG00000147642	ENSG00000187144
ENSG00000131469	ENSG00000167969	ENSG00000169641
ENSG00000135597	ENSG00000117601	ENSG00000256642
ENSG00000168517	ENSG00000162490	ENSG00000213088
ENSG00000117481	ENSG00000197061	ENSG00000151690
ENSG00000083828	ENSG00000087237	ENSG00000139722
ENSG00000170425	ENSG00000187498	ENSG00000154258
ENSG00000153132	ENSG00000221989	ENSG00000108352
ENSG00000010932	ENSG00000102780	ENSG00000145284
ENSG00000205060	ENSG00000128016	ENSG00000156925
ENSG00000149742	ENSG00000239388	ENSG00000188342
ENSG00000127989	ENSG00000067365	ENSG00000124575
ENSG00000197506	ENSG00000129654	ENSG00000065600
ENSG00000188529	ENSG00000084453	ENSG00000096384
ENSG00000187134	ENSG00000111679	ENSG00000140416
ENSG00000116560	ENSG00000122543	ENSG00000105894
ENSG00000168476	ENSG00000134240	ENSG00000138085
ENSG00000147606	ENSG00000155729	ENSG00000100600
ENSG00000187838	ENSG00000105792	ENSG00000168703
ENSG00000141456	ENSG00000146477	ENSG00000010810
ENSG00000197928	ENSG00000176399	ENSG00000143344
ENSG00000196350	ENSG00000132122	ENSG00000115607
ENSG00000159224	ENSG00000182898	ENSG00000079134
ENSG00000133874	ENSG00000105197	ENSG00000132471
ENSG00000146374	ENSG00000107518	ENSG00000119242
ENSG00000143603	ENSG00000182552	ENSG00000198561
ENSG00000184117	ENSG00000143756	ENSG00000240771
ENSG00000175984	ENSG00000106261	ENSG00000160781
ENSG00000188280	ENSG00000119231	ENSG00000064763
ENSG00000103599	ENSG00000140553	ENSG00000170608
ENSG00000156304	ENSG00000174109	ENSG00000140694
ENSG00000145014	ENSG00000215375	ENSG00000181555
ENSG00000170893	ENSG00000183647	ENSG00000144815
ENSG00000137824	ENSG00000157020	ENSG00000143867
ENSG00000104915	ENSG00000179407	ENSG00000179213
ENSG00000127377	ENSG00000258315	ENSG00000087274
ENSG00000215906	ENSG00000145642	ENSG00000168710
ENSG00000198356	ENSG00000184845	ENSG00000161040
ENSG00000104897	ENSG00000137080	ENSG00000231738
ENSG00000169900	ENSG00000269383	ENSG00000111245
ENSG00000183762	ENSG00000137975	ENSG00000176171
ENSG00000176239	ENSG00000215397	ENSG00000268950
ENSG00000169403	ENSG00000125434	ENSG00000104976
ENSG00000099942	ENSG00000196361	ENSG00000023902
ENSG00000177432	ENSG00000198093	ENSG00000167986
ENSG00000143815	ENSG00000122952	ENSG00000186675
ENSG00000175216	ENSG00000168907	ENSG00000229314
ENSG00000167272	ENSG00000052795	ENSG00000054690
ENSG00000140835	ENSG00000180957	ENSG00000134444
ENSG00000173660	ENSG00000067829	ENSG00000105072
ENSG00000158014	ENSG00000141012	ENSG00000062716
ENSG00000178773	ENSG00000133962	ENSG00000197150
ENSG00000160551	ENSG00000121680	ENSG00000139946
ENSG00000079462	ENSG00000147573	ENSG00000234829
ENSG00000197408	ENSG00000105398	ENSG00000114867
ENSG00000159348	ENSG00000184394	ENSG00000116127
ENSG00000120458	ENSG00000140022	ENSG00000075413
ENSG00000134986	ENSG00000182329	ENSG00000166317
ENSG00000156096	ENSG00000171119	ENSG00000154305
ENSG00000162981	ENSG00000188493	ENSG00000165182

ENSG00000196690	ENSG00000175895	ENSG00000153574
ENSG00000166881	ENSG00000078043	ENSG00000117222
ENSG00000173275	ENSG00000247626	ENSG00000135956
ENSG00000175110	ENSG00000068438	ENSG00000154277
ENSG00000101079	ENSG00000116288	ENSG00000158887
ENSG00000130758	ENSG00000119699	ENSG00000138658
ENSG00000180425	ENSG00000092978	ENSG00000154930
ENSG00000068354	ENSG00000164591	ENSG00000158901
ENSG00000150594	ENSG00000102265	ENSG00000204536
ENSG00000136810	ENSG00000187758	ENSG00000196814
ENSG00000198873	ENSG00000130726	ENSG00000176601
ENSG00000146039	ENSG00000120075	ENSG00000127990
ENSG00000239713	ENSG00000131355	ENSG00000144468
ENSG00000183891	ENSG00000135577	ENSG00000187672
ENSG00000160991	ENSG00000155719	ENSG00000203666
ENSG00000150977	ENSG00000120149	ENSG00000116830
ENSG00000168488	ENSG00000235631	ENSG00000004809
ENSG00000185818	ENSG00000172824	ENSG00000164047
ENSG00000168405	ENSG00000213714	ENSG00000166851
ENSG00000214274	ENSG00000106484	ENSG00000181355
ENSG00000086848	ENSG00000134602	ENSG00000138315
ENSG00000204564	ENSG00000127980	ENSG00000166508
ENSG00000243749	ENSG00000176230	ENSG00000136193
ENSG00000114978	ENSG00000119402	ENSG00000170777
ENSG00000130479	ENSG00000165030	ENSG00000077684
ENSG00000204388	ENSG00000258484	ENSG00000158258
ENSG00000166840	ENSG00000171681	ENSG00000181499
ENSG00000155961	ENSG00000133317	ENSG00000100422
ENSG00000171169	ENSG00000253305	ENSG00000061492
ENSG00000065911	ENSG00000106304	ENSG00000167595
ENSG00000197273	ENSG00000118276	ENSG00000125409
ENSG00000146047	ENSG00000164855	ENSG00000182261
ENSG00000110851	ENSG00000163346	ENSG00000162493
ENSG00000116489	ENSG00000115665	ENSG00000128408
ENSG00000085063	ENSG00000102802	ENSG00000163376
ENSG00000146285	ENSG00000110330	ENSG00000197622
ENSG00000170946	ENSG00000186350	ENSG00000152969
ENSG00000127074	ENSG00000161551	ENSG00000006377
ENSG00000106554	ENSG00000055118	ENSG00000117308
ENSG00000172932	ENSG00000085644	ENSG00000085377
ENSG00000146834	ENSG00000042304	ENSG00000111348
ENSG00000129991	ENSG00000184786	ENSG00000099783
ENSG00000144455	ENSG00000161973	ENSG00000102606
ENSG00000072778	ENSG00000188038	ENSG00000141644
ENSG00000144741	ENSG00000172154	ENSG00000139287
ENSG00000137675	ENSG00000078177	ENSG00000253159
ENSG00000101928	ENSG00000151693	ENSG00000189375
ENSG00000124602	ENSG00000236980	ENSG00000240764
ENSG00000257950	ENSG00000204657	ENSG00000139746
ENSG00000163694	ENSG00000065183	ENSG00000122121
ENSG00000231500	ENSG00000118308	ENSG00000121966
ENSG00000168286	ENSG00000125352	ENSG00000214078
ENSG00000189409	ENSG00000167074	ENSG00000173611
ENSG00000179294	ENSG00000165659	ENSG00000005379
ENSG00000242028	ENSG00000124507	ENSG00000136449
ENSG00000163220	ENSG00000164751	ENSG00000145730
ENSG00000214946	ENSG00000198055	ENSG00000145864
ENSG00000163884	ENSG00000167118	ENSG00000132950
ENSG00000187537	ENSG00000021300	ENSG00000173531
ENSG00000163072	ENSG00000153113	ENSG00000040341
ENSG00000123569	ENSG00000138100	ENSG00000259571
ENSG00000224130	ENSG00000186951	ENSG00000163877
ENSG00000157429	ENSG00000188937	ENSG00000183876
ENSG00000144834	ENSG00000160117	ENSG00000107242

ENSG00000117407	ENSG00000212710	ENSG00000187556
ENSG00000164983	ENSG00000139718	ENSG00000116266
ENSG00000214562	ENSG00000177182	ENSG00000123562
ENSG00000153933	ENSG00000164091	ENSG00000018699
ENSG00000141316	ENSG00000212124	ENSG00000122085
ENSG00000162521	ENSG00000160062	ENSG00000012061
ENSG00000183049	ENSG00000117543	ENSG00000215183
ENSG00000164128	ENSG00000169902	ENSG00000107796
ENSG00000185532	ENSG00000139044	ENSG00000143195
ENSG00000125968	ENSG00000101574	ENSG00000100441
ENSG00000184349	ENSG00000119703	ENSG00000156298
ENSG00000009844	ENSG00000125813	ENSG00000176371
ENSG00000115008	ENSG00000111231	ENSG00000071575
ENSG00000181773	ENSG00000213413	ENSG00000114770
ENSG00000163293	ENSG00000205363	ENSG00000104321
ENSG00000159111	ENSG00000073849	ENSG00000013275
ENSG00000008952	ENSG00000173442	ENSG00000139083
ENSG00000164167	ENSG00000125107	ENSG00000164007
ENSG00000186136	ENSG00000161791	ENSG00000148110
ENSG00000127022	ENSG00000185372	ENSG00000178171
ENSG00000164142	ENSG00000204287	ENSG00000186977
ENSG00000177868	ENSG00000175728	ENSG00000141720
ENSG00000140743	ENSG00000074966	ENSG00000171501
ENSG00000197363	ENSG00000088002	ENSG00000163012
ENSG00000168140	ENSG00000171611	ENSG00000100077
ENSG00000145247	ENSG00000100053	ENSG00000205022
ENSG00000176782	ENSG00000171812	ENSG00000177646
ENSG00000185774	ENSG00000010310	ENSG00000033170
ENSG00000204010	ENSG00000174799	ENSG00000163755
ENSG00000101446	ENSG00000115138	ENSG00000132434
ENSG00000120334	ENSG00000197604	ENSG00000162236
ENSG00000147316	ENSG00000184210	ENSG00000127472
ENSG00000120215	ENSG00000100599	ENSG00000137757
ENSG00000099899	ENSG00000138794	ENSG00000073756
ENSG00000155903	ENSG00000152782	ENSG00000165970
ENSG00000136695	ENSG00000160188	ENSG00000198862
ENSG00000225996	ENSG00000132155	ENSG00000149187
ENSG00000197584	ENSG00000129965	ENSG00000109472
ENSG00000141294	ENSG00000116237	ENSG00000249967
ENSG00000149089	ENSG00000130783	ENSG00000137936
ENSG00000129680	ENSG00000145888	ENSG00000128487
ENSG00000178445	ENSG00000204052	ENSG00000051620
ENSG00000165572	ENSG00000108262	ENSG00000109771
ENSG00000196418	ENSG00000180269	ENSG00000186868
ENSG00000152382	ENSG00000115226	ENSG00000140688
ENSG00000135406	ENSG00000163357	ENSG00000163705
ENSG00000160094	ENSG00000106077	ENSG00000102554
ENSG00000123983	ENSG00000159753	ENSG00000115446
ENSG00000168792	ENSG00000160183	ENSG00000101421
ENSG00000126821	ENSG00000136240	ENSG00000142192
ENSG00000155636	ENSG00000184698	ENSG00000068784
ENSG00000164244	ENSG00000152595	ENSG00000114200
ENSG00000261796	ENSG00000133121	ENSG00000185896
ENSG00000204345	ENSG00000137942	ENSG00000177479
ENSG00000130818	ENSG00000105550	ENSG00000119977
ENSG00000147121	ENSG00000159055	ENSG00000186377
ENSG00000092140	ENSG00000144488	ENSG00000155827
ENSG00000139970	ENSG00000184302	ENSG00000111728
ENSG00000168314	ENSG00000198502	ENSG00000114646
ENSG00000183718	ENSG00000166188	ENSG00000116016
ENSG00000147099	ENSG00000204524	ENSG00000116903
ENSG00000171720	ENSG00000254870	ENSG00000198689
ENSG00000165650	ENSG00000132478	ENSG00000136536
ENSG00000004468	ENSG00000109118	ENSG00000198885

ENSG00000180745	ENSG00000143546	ENSG00000110274
ENSG00000099377	ENSG00000181767	ENSG00000163214
ENSG00000160072	ENSG00000135222	ENSG00000171403
ENSG00000189325	ENSG00000171792	ENSG00000104299
ENSG00000047662	ENSG00000103342	ENSG00000177917
ENSG00000116251	ENSG00000137806	ENSG00000187837
ENSG00000197919	ENSG00000168566	ENSG00000072133
ENSG00000178927	ENSG00000100284	ENSG00000111783
ENSG00000138380	ENSG00000017260	ENSG00000102100
ENSG00000179755	ENSG00000156966	ENSG00000172774
ENSG00000165669	ENSG00000256269	ENSG00000196586
ENSG00000122859	ENSG00000124731	ENSG00000070756
ENSG00000144306	ENSG00000146232	ENSG00000182901
ENSG00000157005	ENSG00000077721	ENSG00000141446
ENSG00000251322	ENSG00000164485	ENSG00000265969
ENSG00000121594	ENSG00000166579	ENSG00000066468
ENSG00000268788	ENSG00000128322	ENSG00000114124
ENSG00000250510	ENSG00000115561	ENSG00000085982
ENSG00000139687	ENSG00000105290	ENSG00000177932
ENSG00000143222	ENSG00000172339	ENSG00000197992
ENSG00000177468	ENSG00000164695	ENSG00000012223
ENSG00000162891	ENSG00000137492	ENSG00000083223
ENSG00000121892	ENSG00000006695	ENSG00000183354
ENSG00000134594	ENSG00000189127	ENSG00000147548
ENSG00000141504	ENSG00000268178	ENSG00000164113
ENSG00000149809	ENSG00000134644	ENSG00000143107
ENSG00000163964	ENSG00000117400	ENSG00000174527
ENSG00000141959	ENSG00000126945	ENSG00000144233
ENSG00000131730	ENSG00000111665	ENSG00000124313
ENSG00000165192	ENSG00000242220	ENSG00000175073
ENSG00000159720	ENSG00000169242	ENSG00000147655
ENSG00000177191	ENSG00000114383	ENSG00000131400
ENSG00000168496	ENSG00000141076	ENSG00000181472
ENSG00000134551	ENSG00000186400	ENSG00000180104
ENSG00000159210	ENSG00000037474	ENSG00000140526
ENSG00000158711	ENSG00000112584	ENSG00000243137
ENSG00000169752	ENSG00000139890	ENSG00000166803
ENSG00000165762	ENSG00000121940	ENSG00000114315
ENSG00000180953	ENSG00000125975	ENSG00000147536
ENSG00000204928	ENSG00000230062	ENSG00000118495
ENSG00000138785	ENSG00000163687	ENSG00000140284
ENSG00000176472	ENSG00000214491	ENSG00000117525
ENSG00000235531	ENSG00000184047	ENSG00000111424
ENSG00000101347	ENSG00000222047	ENSG00000128886
ENSG00000244687	ENSG00000153002	ENSG00000103742
ENSG00000116209	ENSG00000051128	ENSG00000134324
ENSG00000188069	ENSG00000128285	ENSG00000073711
ENSG00000106603	ENSG00000182923	ENSG00000204246
ENSG00000119048	ENSG00000256574	ENSG00000145675
ENSG00000049656	ENSG00000100796	ENSG00000166924
ENSG00000186965	ENSG00000258588	ENSG00000171135
ENSG00000105523	ENSG00000139218	ENSG00000163659
ENSG00000139146	ENSG00000267596	ENSG00000142197
ENSG00000112031	ENSG00000101194	ENSG00000188987
ENSG00000141497	ENSG00000174684	ENSG00000067141
ENSG00000162783	ENSG00000158109	ENSG00000154760
ENSG00000241106	ENSG00000136044	ENSG00000108370
ENSG00000152818	ENSG00000163898	ENSG00000150938
ENSG00000169504	ENSG00000102057	ENSG00000177692
ENSG00000162069	ENSG00000113163	ENSG00000168939
ENSG00000175985	ENSG00000107404	ENSG00000239697
ENSG00000143353	ENSG00000221855	ENSG00000166634
ENSG00000138767	ENSG00000178567	ENSG00000133055
ENSG00000203756	ENSG00000131686	ENSG00000134812

ENSG00000215193	ENSG00000243649	ENSG00000107249
ENSG00000169062	ENSG00000169291	ENSG00000152583
ENSG00000117640	ENSG00000167553	ENSG00000170419
ENSG00000161850	ENSG00000082258	ENSG00000169777
ENSG00000172367	ENSG00000008710	ENSG00000079335
ENSG00000124374	ENSG00000082556	ENSG00000141698
ENSG00000214435	ENSG00000143217	ENSG00000128610
ENSG00000140307	ENSG00000185269	ENSG00000123144
ENSG00000102683	ENSG00000170214	ENSG00000076108
ENSG00000164508	ENSG00000184730	ENSG00000177409
ENSG00000008130	ENSG00000132275	ENSG00000165118
ENSG00000198612	ENSG00000115310	ENSG00000168386
ENSG00000140451	ENSG00000170876	ENSG00000154175
ENSG00000115170	ENSG00000241484	ENSG00000198513
ENSG00000175467	ENSG00000145861	ENSG00000144867
ENSG00000137331	ENSG00000129152	ENSG00000176774
ENSG00000100983	ENSG00000110719	ENSG00000091137
ENSG00000110931	ENSG00000149328	ENSG00000136488
ENSG00000167751	ENSG00000175718	ENSG00000243811
ENSG00000132589	ENSG00000132470	ENSG00000124357
ENSG00000266967	ENSG00000198939	ENSG00000162877
ENSG00000054796	ENSG00000136834	ENSG00000151967
ENSG00000146411	ENSG00000169951	ENSG00000153347
ENSG00000142538	ENSG00000164600	ENSG00000182346
ENSG00000162302	ENSG00000140650	ENSG00000198646
ENSG00000103175	ENSG00000167104	ENSG00000168675
ENSG00000010270	ENSG00000173826	ENSG00000065357
ENSG00000139209	ENSG00000107566	ENSG00000132259
ENSG00000253110	ENSG00000184792	ENSG00000162601
ENSG00000182742	ENSG00000151746	ENSG00000175203
ENSG00000204576	ENSG00000141084	ENSG00000159399
ENSG00000079246	ENSG00000144045	ENSG00000197969
ENSG00000179431	ENSG00000066135	ENSG00000182575
ENSG00000177879	ENSG00000151718	ENSG00000095637
ENSG00000123685	ENSG00000186153	ENSG00000184361
ENSG00000169194	ENSG00000011143	ENSG00000196155
ENSG00000052802	ENSG00000244165	ENSG00000102174
ENSG00000170852	ENSG00000168918	ENSG00000175130
ENSG00000220256	ENSG00000040933	ENSG00000115486
ENSG00000152778	ENSG00000132703	ENSG00000140455
ENSG00000157216	ENSG00000166664	ENSG00000136161
ENSG00000114654	ENSG00000132026	ENSG00000085265
ENSG00000196458	ENSG00000138802	ENSG00000151136
ENSG00000133135	ENSG00000198797	ENSG00000186628
ENSG00000178372	ENSG00000104853	ENSG00000173039
ENSG00000164794	ENSG00000111652	ENSG00000254709
ENSG00000168564	ENSG00000166710	ENSG00000165516
ENSG00000086102	ENSG00000164066	ENSG00000121898
ENSG00000136159	ENSG00000014216	ENSG00000148300
ENSG00000189420	ENSG00000152049	ENSG00000168676
ENSG00000251246	ENSG00000154188	ENSG00000156675
ENSG00000162006	ENSG00000196453	ENSG00000173545
ENSG00000183625	ENSG00000135090	ENSG00000186191
ENSG00000050628	ENSG00000203722	ENSG00000184384
ENSG00000185615	ENSG00000138434	ENSG00000166435
ENSG00000241563	ENSG00000165891	ENSG00000114859
ENSG00000065154	ENSG00000157350	ENSG00000132406
ENSG00000178826	ENSG00000155629	ENSG00000185926
ENSG00000177098	ENSG00000197312	ENSG00000078053
ENSG00000115963	ENSG00000059915	ENSG00000184478
ENSG00000141378	ENSG00000185559	ENSG00000133065
ENSG00000106993	ENSG00000084110	ENSG00000137449
ENSG00000174130	ENSG00000244694	ENSG00000162526
ENSG00000145439	ENSG00000103546	ENSG00000172765

ENSG00000258847	ENSG00000105376	ENSG00000070961
ENSG00000205707	ENSG00000125746	ENSG00000236398
ENSG00000116863	ENSG00000137124	ENSG00000131165
ENSG00000117533	ENSG00000109466	ENSG00000011426
ENSG00000104327	ENSG00000135776	ENSG00000188672
ENSG00000128881	ENSG00000196917	ENSG00000203832
ENSG00000164291	ENSG00000076356	ENSG00000047579
ENSG00000267824	ENSG00000117519	ENSG00000143882
ENSG00000118245	ENSG00000162482	ENSG00000120051
ENSG00000187766	ENSG00000139697	ENSG00000169344
ENSG00000089041	ENSG00000140511	ENSG00000171724
ENSG00000251201	ENSG00000143333	ENSG00000093167
ENSG00000196900	ENSG00000153779	ENSG00000137338
ENSG00000180979	ENSG00000165219	ENSG00000203734
ENSG00000168671	ENSG00000091844	ENSG00000164168
ENSG00000182783	ENSG00000170482	ENSG00000250151
ENSG00000117862	ENSG00000139572	ENSG00000108839
ENSG00000119919	ENSG00000182255	ENSG00000198837
ENSG00000141696	ENSG00000242441	ENSG00000198756
ENSG00000196987	ENSG00000182583	ENSG00000179295
ENSG00000165861	ENSG00000187288	ENSG00000006837
ENSG00000159200	ENSG00000198650	ENSG00000147206
ENSG00000149657	ENSG00000171291	ENSG00000241685
ENSG00000112499	ENSG00000163736	ENSG00000170044
ENSG00000116459	ENSG00000080819	ENSG00000167748
ENSG00000162894	ENSG00000135114	ENSG00000183560
ENSG00000126233	ENSG00000204531	ENSG00000164087
ENSG00000105835	ENSG00000164619	ENSG00000143294
ENSG00000204850	ENSG00000086548	ENSG00000107864
ENSG00000205867	ENSG00000105467	ENSG00000101000
ENSG00000147256	ENSG00000196123	ENSG00000241119
ENSG00000166143	ENSG00000184481	ENSG00000143226
ENSG00000175906	ENSG00000135454	ENSG00000177383
ENSG00000110680	ENSG00000108759	ENSG00000197454
ENSG00000122406	ENSG00000170619	ENSG00000120262
ENSG00000258873	ENSG00000151632	ENSG00000203757
ENSG00000168228	ENSG00000175928	ENSG00000171862
ENSG00000204683	ENSG00000184677	ENSG00000127804
ENSG00000204308	ENSG00000136824	ENSG00000121578
ENSG00000119616	ENSG00000255604	ENSG00000130997
ENSG00000134186	ENSG00000121101	ENSG00000167578
ENSG00000167094	ENSG00000111605	ENSG00000240694
ENSG00000069493	ENSG00000066279	ENSG00000197930
ENSG00000189430	ENSG00000185897	ENSG00000148680
ENSG00000184752	ENSG00000164535	ENSG00000106636
ENSG00000180316	ENSG00000197591	ENSG00000196510
ENSG00000162873	ENSG00000185686	ENSG00000125375
ENSG00000186787	ENSG00000163362	ENSG00000163812
ENSG00000167656	ENSG00000104848	ENSG00000138029
ENSG00000013297	ENSG00000144227	ENSG00000184708
ENSG00000182896	ENSG00000116981	ENSG00000182944
ENSG00000162066	ENSG00000109063	ENSG00000100395
ENSG00000137692	ENSG00000145349	ENSG00000170681
ENSG00000254445	ENSG00000137573	ENSG00000149489
ENSG00000178982	ENSG00000130054	ENSG00000166090
ENSG00000141505	ENSG00000160961	ENSG00000132323
ENSG00000132446	ENSG00000063244	ENSG00000163703
ENSG00000153721	ENSG00000132376	ENSG00000171517
ENSG00000239672	ENSG00000100012	ENSG00000213920
ENSG00000159110	ENSG00000091513	ENSG00000100625
ENSG00000152558	ENSG00000186792	ENSG00000121807
ENSG00000258436	ENSG00000141279	ENSG00000105419
ENSG00000112237	ENSG00000125207	ENSG00000140030
ENSG00000178966	ENSG00000136205	ENSG00000182141

ENSG00000172568	ENSG00000120658	ENSG00000129682
ENSG00000189057	ENSG00000170929	ENSG00000038210
ENSG00000178222	ENSG00000172037	ENSG00000116285
ENSG00000197191	ENSG00000005194	ENSG00000105323
ENSG00000095794	ENSG00000182405	ENSG00000258838
ENSG00000189157	ENSG00000139626	ENSG00000100448
ENSG00000095321	ENSG00000164761	ENSG00000186318
ENSG00000081923	ENSG00000172179	ENSG00000105388
ENSG00000174151	ENSG00000118729	ENSG00000130638
ENSG00000165417	ENSG00000100994	ENSG00000132254
ENSG00000164985	ENSG00000205221	ENSG00000118292
ENSG00000174276	ENSG00000198131	ENSG00000186472
ENSG00000134308	ENSG00000250641	ENSG00000174339
ENSG00000170439	ENSG00000241360	ENSG00000106330
ENSG00000166797	ENSG00000162944	ENSG00000159231
ENSG00000076685	ENSG00000034152	ENSG00000203989
ENSG00000198738	ENSG00000101670	ENSG00000149972
ENSG00000112183	ENSG00000017373	ENSG00000250349
ENSG00000111785	ENSG00000240583	ENSG00000178607
ENSG00000145741	ENSG00000166592	ENSG00000116741
ENSG00000145248	ENSG00000162390	ENSG00000141499
ENSG00000139637	ENSG00000166246	ENSG00000132464
ENSG00000106355	ENSG00000173269	ENSG00000172867
ENSG00000165644	ENSG00000165807	ENSG00000139842
ENSG00000125844	ENSG00000178467	ENSG00000180479
ENSG00000139330	ENSG00000198718	ENSG00000137225
ENSG00000242612	ENSG00000020577	ENSG00000197603
ENSG00000131370	ENSG00000166734	ENSG00000120693
ENSG00000149300	ENSG00000186049	ENSG00000170866
ENSG00000146802	ENSG00000167654	ENSG00000157693
ENSG00000117906	ENSG00000111241	ENSG00000164398
ENSG00000150764	ENSG00000104133	ENSG00000176787
ENSG00000071203	ENSG00000165914	ENSG00000214655
ENSG00000160055	ENSG00000101017	ENSG00000170683
ENSG00000138483	ENSG00000134697	ENSG00000066629
ENSG00000197532	ENSG00000156042	ENSG00000168152
ENSG00000168884	ENSG00000165646	ENSG00000188162
ENSG00000062194	ENSG00000134183	ENSG00000136861
ENSG00000170502	ENSG00000187037	ENSG00000125895
ENSG00000146731	ENSG00000162664	ENSG00000153896
ENSG00000211452	ENSG00000259753	ENSG00000179988
ENSG00000106086	ENSG00000065325	ENSG00000204293
ENSG00000259243	ENSG00000266302	ENSG00000164038
ENSG00000055950	ENSG00000025800	ENSG00000070950
ENSG00000268657	ENSG00000250588	ENSG00000075415
ENSG00000135213	ENSG00000205937	ENSG00000112983
ENSG00000162441	ENSG00000196199	ENSG00000166377
ENSG00000085514	ENSG00000101493	ENSG00000158669
ENSG00000196923	ENSG00000114353	ENSG00000175048
ENSG00000165355	ENSG00000172500	ENSG00000167193
ENSG00000111319	ENSG00000128510	ENSG00000179603
ENSG00000106245	ENSG00000145384	ENSG00000197467
ENSG00000168952	ENSG00000129518	ENSG00000124490
ENSG00000164124	ENSG00000145982	ENSG00000112294
ENSG00000169249	ENSG00000138356	ENSG00000163638
ENSG00000166598	ENSG00000163291	ENSG00000213204
ENSG00000116670	ENSG00000185437	ENSG00000174776
ENSG00000110900	ENSG00000167286	ENSG00000132635
ENSG00000214954	ENSG00000152580	ENSG00000214021
ENSG00000198624	ENSG00000162753	ENSG00000114439
ENSG00000112715	ENSG00000161649	ENSG00000158104
ENSG00000110955	ENSG00000204518	ENSG00000213780
ENSG00000159248	ENSG00000175137	ENSG00000163545
ENSG00000135974	ENSG00000196230	ENSG00000161204

ENSG00000120314	ENSG00000182606	ENSG00000167098
ENSG00000180245	ENSG00000172977	ENSG00000139880
ENSG00000006607	ENSG00000055130	ENSG00000112624
ENSG00000116793	ENSG00000188818	ENSG00000151883
ENSG00000170085	ENSG00000148396	ENSG00000122718
ENSG00000150477	ENSG00000160271	ENSG00000171903
ENSG00000134265	ENSG00000151117	ENSG00000111799
ENSG00000185674	ENSG00000103326	ENSG00000155666
ENSG00000099284	ENSG00000196466	ENSG00000136244
ENSG00000185507	ENSG00000187513	ENSG00000131626
ENSG00000166049	ENSG00000162063	ENSG00000132024
ENSG00000168936	ENSG00000106511	ENSG00000127831
ENSG00000137818	ENSG00000152620	ENSG00000185633
ENSG00000185097	ENSG00000148215	ENSG00000123352
ENSG00000011600	ENSG00000188828	ENSG00000187240
ENSG00000132716	ENSG00000117676	ENSG00000198734
ENSG00000258210	ENSG00000016391	ENSG00000182179
ENSG00000125485	ENSG00000107959	ENSG00000183024
ENSG00000049541	ENSG00000165996	ENSG00000128604
ENSG00000183386	ENSG00000161265	ENSG00000148204
ENSG00000223510	ENSG00000169554	ENSG00000064651
ENSG00000042753	ENSG00000204392	ENSG00000164068
ENSG00000198336	ENSG00000129932	ENSG00000137760
ENSG00000174899	ENSG00000110713	ENSG00000180354
ENSG00000105677	ENSG00000008083	ENSG00000197006
ENSG00000131143	ENSG00000169129	ENSG00000082996
ENSG00000084073	ENSG00000188155	ENSG00000115593
ENSG00000141013	ENSG00000174353	ENSG00000145545
ENSG00000243725	ENSG00000131914	ENSG00000244731
ENSG00000160633	ENSG00000121957	ENSG00000112249
ENSG00000215883	ENSG00000185664	ENSG00000116539
ENSG00000269026	ENSG00000171004	ENSG00000138185
ENSG00000140326	ENSG00000067992	ENSG00000139624
ENSG00000178917	ENSG00000143845	ENSG00000096717
ENSG00000013374	ENSG00000102854	ENSG00000112782
ENSG00000127578	ENSG00000120341	ENSG00000170909
ENSG00000168005	ENSG00000150054	ENSG00000134258
ENSG00000119596	ENSG00000087191	ENSG00000077097
ENSG00000167202	ENSG00000143442	ENSG00000180182
ENSG00000135317	ENSG00000083814	ENSG00000102104
ENSG00000124733	ENSG00000152154	ENSG00000137801
ENSG00000238243	ENSG00000130311	ENSG00000109171
ENSG00000229183	ENSG00000197977	ENSG00000182240
ENSG00000171530	ENSG00000082293	ENSG00000166938
ENSG00000160014	ENSG00000119414	ENSG00000159216
ENSG00000143033	ENSG00000096395	ENSG00000182326
ENSG00000076248	ENSG00000170807	ENSG00000169621
ENSG00000127362	ENSG00000223865	ENSG00000165359
ENSG00000205643	ENSG00000175455	ENSG00000196267
ENSG00000165688	ENSG00000162572	ENSG00000003987
ENSG00000080166	ENSG00000020129	ENSG00000204394
ENSG00000073670	ENSG00000143552	ENSG00000149532
ENSG00000119760	ENSG00000198877	ENSG00000196116
ENSG00000157330	ENSG00000129910	ENSG00000197816
ENSG00000166847	ENSG00000178084	ENSG00000161010
ENSG00000182591	ENSG00000178234	ENSG00000007171
ENSG00000138036	ENSG00000092929	ENSG00000107816
ENSG00000166106	ENSG00000146383	ENSG00000112414
ENSG00000256870	ENSG00000140563	ENSG00000174705
ENSG00000127399	ENSG00000181785	ENSG00000100023
ENSG00000196591	ENSG00000104341	ENSG00000125484
ENSG00000185177	ENSG00000185963	ENSG00000196659
ENSG00000204147	ENSG00000133895	ENSG00000180785
ENSG00000198668	ENSG00000148541	ENSG00000102893

ENSG00000176909	ENSG00000123485	ENSG00000142039
ENSG00000067167	ENSG00000109184	ENSG00000078589
ENSG00000146463	ENSG00000155893	ENSG00000131069
ENSG00000154165	ENSG00000166126	ENSG00000180573
ENSG00000163840	ENSG00000091664	ENSG00000128944
ENSG00000132591	ENSG00000159346	ENSG00000136167
ENSG00000205358	ENSG00000105993	ENSG00000051382
ENSG00000141526	ENSG00000215045	ENSG00000184083
ENSG00000147669	ENSG00000109790	ENSG00000165617
ENSG00000144891	ENSG00000080618	ENSG00000165805
ENSG00000104671	ENSG00000026950	ENSG00000106628
ENSG00000188322	ENSG00000064703	ENSG00000104472
ENSG00000203618	ENSG00000144579	ENSG00000101224
ENSG00000142025	ENSG00000177721	ENSG00000253797
ENSG00000129116	ENSG00000221938	ENSG00000101367
ENSG00000185627	ENSG00000172828	ENSG00000213160
ENSG00000165506	ENSG00000262246	ENSG00000130413
ENSG00000100075	ENSG00000176907	ENSG00000115042
ENSG00000129696	ENSG00000141252	ENSG00000255292
ENSG00000258465	ENSG00000103522	ENSG00000166743
ENSG00000064692	ENSG00000140557	ENSG00000138378
ENSG00000151151	ENSG00000104808	ENSG00000203993
ENSG00000170369	ENSG00000143627	ENSG00000137648
ENSG00000143727	ENSG00000163814	ENSG00000133812
ENSG00000180539	ENSG00000167508	ENSG00000172465
ENSG00000183943	ENSG00000120903	ENSG00000144331
ENSG00000196482	ENSG00000213096	ENSG00000142937
ENSG00000173041	ENSG00000162727	ENSG00000100968
ENSG00000099849	ENSG00000243489	ENSG00000198945
ENSG00000185946	ENSG00000096093	ENSG00000159374
ENSG00000182415	ENSG00000140265	ENSG00000137171
ENSG00000133101	ENSG00000139370	ENSG00000164082
ENSG00000188732	ENSG00000158113	ENSG00000205327
ENSG00000152454	ENSG00000162552	ENSG00000011422
ENSG00000128218	ENSG00000136527	ENSG00000093217
ENSG00000114120	ENSG00000171864	ENSG00000145491
ENSG00000164010	ENSG00000006704	ENSG00000112186
ENSG00000112514	ENSG00000143125	ENSG00000076662
ENSG00000162616	ENSG00000111696	ENSG00000140749
ENSG00000258231	ENSG00000126368	ENSG00000118307
ENSG00000137804	ENSG00000172869	ENSG00000198964
ENSG00000116096	ENSG00000145029	ENSG00000164465
ENSG00000186409	ENSG00000142619	ENSG00000171446
ENSG00000145708	ENSG00000139648	ENSG00000163017
ENSG00000204038	ENSG00000105127	ENSG00000162522
ENSG00000130045	ENSG00000221900	ENSG00000117385
ENSG00000177889	ENSG00000143228	ENSG00000167985
ENSG00000179163	ENSG00000044446	ENSG00000185722
ENSG00000153165	ENSG00000089012	ENSG00000176540
ENSG00000087299	ENSG00000154229	ENSG00000171051
ENSG00000184811	ENSG00000253831	ENSG00000176473
ENSG00000166250	ENSG00000154099	ENSG00000221931
ENSG00000248933	ENSG00000130558	ENSG00000109436
ENSG00000204271	ENSG00000089847	ENSG00000185002
ENSG00000182687	ENSG00000186185	ENSG00000106366
ENSG00000134917	ENSG00000115919	ENSG00000165202
ENSG00000124786	ENSG00000165312	ENSG00000133884
ENSG00000119147	ENSG00000169035	ENSG00000198518
ENSG00000175197	ENSG00000187054	ENSG00000104413
ENSG00000151532	ENSG00000136144	ENSG00000111790
ENSG00000177275	ENSG00000111331	ENSG00000144061
ENSG00000153563	ENSG00000186866	ENSG00000149090
ENSG00000186971	ENSG00000142208	ENSG00000103051
ENSG00000198909	ENSG00000133961	ENSG00000122861

ENSG00000184903	ENSG00000198785	ENSG00000185610
ENSG00000235718	ENSG00000182636	ENSG00000198399
ENSG00000214513	ENSG00000122482	ENSG00000196622
ENSG00000128311	ENSG00000155833	ENSG00000131203
ENSG00000183569	ENSG00000198753	ENSG00000102225
ENSG00000167700	ENSG00000083307	ENSG00000114573
ENSG00000178149	ENSG00000100403	ENSG00000142166
ENSG00000100138	ENSG00000187260	ENSG00000060971
ENSG00000189298	ENSG00000111641	ENSG00000141738
ENSG00000177058	ENSG00000262576	ENSG00000177885
ENSG00000196081	ENSG00000205978	ENSG00000167615
ENSG00000254997	ENSG00000243251	ENSG00000132554
ENSG00000156869	ENSG00000185652	ENSG00000143486
ENSG00000112855	ENSG00000115556	ENSG00000083290
ENSG00000171124	ENSG00000126777	ENSG00000153498
ENSG00000147601	ENSG00000109851	ENSG00000004478
ENSG00000227059	ENSG00000171450	ENSG00000143452
ENSG00000162998	ENSG00000173852	ENSG00000197376
ENSG00000120833	ENSG00000008441	ENSG00000168938
ENSG00000167173	ENSG00000112679	ENSG00000145220
ENSG00000163352	ENSG00000182685	ENSG00000178950
ENSG00000131951	ENSG00000142751	ENSG00000117598
ENSG00000162688	ENSG00000070413	ENSG00000089199
ENSG00000171956	ENSG00000123080	ENSG00000179300
ENSG00000157191	ENSG00000179546	ENSG00000004534
ENSG00000148377	ENSG00000149527	ENSG00000133110
ENSG00000149781	ENSG00000105538	ENSG00000119686
ENSG00000162980	ENSG00000153558	ENSG00000205863
ENSG00000152484	ENSG00000197362	ENSG00000205726
ENSG00000135047	ENSG00000019186	ENSG00000184226
ENSG00000126067	ENSG00000070159	ENSG00000179241
ENSG00000106400	ENSG00000108813	ENSG00000137709
ENSG00000120860	ENSG00000089692	ENSG00000198062
ENSG00000189308	ENSG00000119403	ENSG00000152193
ENSG00000186480	ENSG00000197594	ENSG00000178498
ENSG00000136720	ENSG00000120885	ENSG00000087269
ENSG00000229292	ENSG00000100603	ENSG00000130202
ENSG00000185554	ENSG00000170430	ENSG00000136485
ENSG00000100532	ENSG00000103042	ENSG00000250709
ENSG00000133193	ENSG00000137876	ENSG00000250232
ENSG00000172086	ENSG00000163312	ENSG00000112137
ENSG00000117791	ENSG00000186442	ENSG00000170255
ENSG00000173452	ENSG00000064655	ENSG00000116745
ENSG00000090104	ENSG00000135569	ENSG00000100299
ENSG00000068745	ENSG00000162511	ENSG00000185298
ENSG00000183463	ENSG00000058056	ENSG00000168778
ENSG00000149480	ENSG00000180398	ENSG00000108828
ENSG00000254636	ENSG00000124721	ENSG00000111261
ENSG00000075399	ENSG00000187715	ENSG00000165511
ENSG00000174672	ENSG00000067836	ENSG00000040731
ENSG00000135502	ENSG00000176895	ENSG00000215262
ENSG00000132514	ENSG00000172006	ENSG00000108349
ENSG00000108587	ENSG00000137133	ENSG00000057294
ENSG00000136425	ENSG00000171428	ENSG00000117143
ENSG00000162073	ENSG00000172819	ENSG00000197971
ENSG00000185875	ENSG00000171747	ENSG00000110888
ENSG00000116711	ENSG00000118705	ENSG00000065150
ENSG00000213699	ENSG00000113368	ENSG00000157064
ENSG00000180828	ENSG00000126461	ENSG00000174016
ENSG00000135617	ENSG00000175535	ENSG00000155252
ENSG00000131979	ENSG00000173517	ENSG00000153250
ENSG00000240432	ENSG00000134765	ENSG00000142230
ENSG00000197223	ENSG00000092529	ENSG00000143740
ENSG00000109705	ENSG00000101052	ENSG00000140950

ENSG00000150907	ENSG00000156345	ENSG00000008256
ENSG00000173786	ENSG00000196734	ENSG00000130538
ENSG00000051009	ENSG00000204183	ENSG00000144681
ENSG00000166856	ENSG00000172296	ENSG00000153832
ENSG00000068097	ENSG00000136451	ENSG00000144730
ENSG00000170516	ENSG00000183035	ENSG00000170248
ENSG00000134970	ENSG00000171617	ENSG00000134827
ENSG00000167863	ENSG00000203924	ENSG00000196378
ENSG00000175414	ENSG00000102221	ENSG00000095539
ENSG00000034713	ENSG00000064787	ENSG00000117360
ENSG00000152465	ENSG00000042088	ENSG00000100344
ENSG00000101199	ENSG00000105085	ENSG00000170925
ENSG00000167112	ENSG00000159788	ENSG00000197147
ENSG00000138083	ENSG00000005486	ENSG00000138316
ENSG00000216490	ENSG00000135355	ENSG00000135312
ENSG00000127952	ENSG00000113140	ENSG00000135913
ENSG00000203872	ENSG00000080224	ENSG00000008128
ENSG00000173253	ENSG00000143569	ENSG00000151445
ENSG00000186008	ENSG00000104324	ENSG00000214787
ENSG00000128965	ENSG00000010318	ENSG00000142623
ENSG00000100865	ENSG00000110107	ENSG00000168291
ENSG00000123728	ENSG00000156603	ENSG00000128849
ENSG00000132840	ENSG00000178401	ENSG00000153294
ENSG00000101115	ENSG00000100462	ENSG00000049769
ENSG00000159713	ENSG00000185753	ENSG00000146233
ENSG00000119185	ENSG00000131264	ENSG00000139117
ENSG00000156735	ENSG00000150093	ENSG00000071537
ENSG00000183643	ENSG00000175084	ENSG00000197217
ENSG00000162929	ENSG00000168610	ENSG00000119401
ENSG00000167220	ENSG00000183269	ENSG00000001497
ENSG00000184216	ENSG00000102145	ENSG00000163322
ENSG00000104129	ENSG00000126746	ENSG00000158525
ENSG00000144847	ENSG00000078487	ENSG00000196344
ENSG00000143942	ENSG00000086570	ENSG00000145246
ENSG00000214128	ENSG00000160870	ENSG00000134538
ENSG00000136936	ENSG00000105176	ENSG00000147654
ENSG00000219159	ENSG00000119608	ENSG00000175619
ENSG00000086506	ENSG00000160445	ENSG00000186517
ENSG00000172167	ENSG00000182631	ENSG00000176893
ENSG00000144736	ENSG00000198099	ENSG00000089057
ENSG00000110871	ENSG00000146166	ENSG00000165633
ENSG00000115073	ENSG00000100884	ENSG00000166450
ENSG00000189050	ENSG00000124782	ENSG00000174942
ENSG00000141905	ENSG00000082684	ENSG00000165632
ENSG00000141858	ENSG00000197674	ENSG00000105708
ENSG00000161055	ENSG00000129925	ENSG00000036565
ENSG00000178110	ENSG00000123165	ENSG00000120868
ENSG00000125850	ENSG00000154096	ENSG00000179934
ENSG00000184402	ENSG00000108296	ENSG00000114268
ENSG00000182484	ENSG00000143079	ENSG00000177455
ENSG00000102967	ENSG00000154059	ENSG00000165953
ENSG00000204511	ENSG00000128710	ENSG00000107186
ENSG00000110717	ENSG00000176531	ENSG00000152213
ENSG00000125888	ENSG00000124614	ENSG00000196104
ENSG00000131558	ENSG00000221838	ENSG00000120889
ENSG00000185594	ENSG00000083635	ENSG00000236104
ENSG00000183470	ENSG00000228567	ENSG00000103404
ENSG00000165246	ENSG00000166340	ENSG00000171307
ENSG00000198799	ENSG00000103534	ENSG00000107537
ENSG00000167741	ENSG00000205126	ENSG00000144029
ENSG00000160959	ENSG00000172728	ENSG00000139629
ENSG00000125871	ENSG00000172362	ENSG00000176783
ENSG00000198538	ENSG00000159658	ENSG00000135506
ENSG00000131236	ENSG00000143061	ENSG00000111732

ENSG00000146859	ENSG00000118503	ENSG00000130035
ENSG00000123179	ENSG00000137198	ENSG00000248871
ENSG00000007264	ENSG00000084710	ENSG00000158467
ENSG00000258083	ENSG00000203837	ENSG00000064419
ENSG00000187325	ENSG00000065675	ENSG00000242689
ENSG00000101188	ENSG00000168310	ENSG00000050767
ENSG00000169410	ENSG00000094631	ENSG00000196248
ENSG00000153443	ENSG00000112116	ENSG00000146909
ENSG00000183615	ENSG00000071859	ENSG00000156103
ENSG00000186222	ENSG00000125826	ENSG00000196277
ENSG00000136574	ENSG00000152527	ENSG00000114739
ENSG00000147400	ENSG00000138834	ENSG00000100490
ENSG00000069764	ENSG00000151552	ENSG00000007923
ENSG00000266956	ENSG00000140092	ENSG00000125903
ENSG00000162373	ENSG00000070770	ENSG00000186272
ENSG00000135587	ENSG00000128536	ENSG00000264545
ENSG00000174010	ENSG00000158747	ENSG00000132972
ENSG00000116833	ENSG00000121210	ENSG00000253953
ENSG00000163576	ENSG00000101327	ENSG00000081803
ENSG00000128165	ENSG00000176014	ENSG00000157542
ENSG00000187164	ENSG00000145832	ENSG00000002933
ENSG00000163378	ENSG00000103319	ENSG00000155463
ENSG00000198792	ENSG00000099204	ENSG00000117528
ENSG00000134056	ENSG00000196581	ENSG00000105821
ENSG00000121361	ENSG00000132671	ENSG00000122574
ENSG00000137821	ENSG00000120937	ENSG00000109674
ENSG00000100033	ENSG00000111537	ENSG00000172840
ENSG00000204220	ENSG00000066697	ENSG00000165471
ENSG00000146399	ENSG00000188158	ENSG00000166073
ENSG00000014824	ENSG00000154978	ENSG00000115828
ENSG00000164002	ENSG00000090889	ENSG00000105251
ENSG00000124217	ENSG00000123342	ENSG00000102984
ENSG00000206127	ENSG00000130540	ENSG00000162607
ENSG00000221859	ENSG00000099834	ENSG00000146147
ENSG00000171631	ENSG00000182223	ENSG00000169760
ENSG00000092377	ENSG00000168014	ENSG00000100239
ENSG00000182854	ENSG00000139132	ENSG00000204310
ENSG00000166669	ENSG00000244474	ENSG00000198553
ENSG00000058091	ENSG00000148090	ENSG00000136688
ENSG00000148335	ENSG00000163040	ENSG00000106344
ENSG00000188215	ENSG00000164850	ENSG00000136854
ENSG00000204264	ENSG00000130338	ENSG00000139514
ENSG00000179886	ENSG00000204389	ENSG00000123575
ENSG00000129355	ENSG00000204021	ENSG00000153093
ENSG00000163257	ENSG00000151726	ENSG00000133794
ENSG00000166510	ENSG00000169548	ENSG00000087076
ENSG00000129028	ENSG00000086991	ENSG00000136940
ENSG00000187713	ENSG00000087365	ENSG00000100368
ENSG00000148835	ENSG00000261115	ENSG00000169860
ENSG00000196436	ENSG00000147650	ENSG00000066044
ENSG00000178201	ENSG00000049089	ENSG00000159128
ENSG00000084112	ENSG00000151025	ENSG00000153823
ENSG00000145020	ENSG00000188981	ENSG00000160190
ENSG00000197162	ENSG00000101282	ENSG00000089177
ENSG00000197360	ENSG00000050327	ENSG00000154997
ENSG00000258713	ENSG00000095383	ENSG00000067177
ENSG00000113448	ENSG00000102445	ENSG00000100078
ENSG00000163938	ENSG00000164867	ENSG00000001036
ENSG00000104825	ENSG00000183748	ENSG00000127325
ENSG00000131779	ENSG00000078081	ENSG00000003096
ENSG00000048545	ENSG00000175215	ENSG00000164211
ENSG00000187714	ENSG00000130695	ENSG00000124120
ENSG00000255468	ENSG00000136099	ENSG00000173276
ENSG00000204619	ENSG00000166828	ENSG00000167642

ENSG00000175643	ENSG00000143367	ENSG00000162989
ENSG00000108395	ENSG00000163624	ENSG00000213023
ENSG00000156531	ENSG00000239590	ENSG00000197713
ENSG00000152942	ENSG00000157483	ENSG00000106410
ENSG00000119906	ENSG00000126705	ENSG00000103512
ENSG00000269709	ENSG00000176788	ENSG00000073464
ENSG00000175183	ENSG00000140945	ENSG00000164161
ENSG00000198189	ENSG00000070047	ENSG00000067064
ENSG00000128309	ENSG00000107281	ENSG00000205683
ENSG00000215595	ENSG00000122359	ENSG00000167612
ENSG00000197044	ENSG00000102974	ENSG00000205629
ENSG00000132297	ENSG00000198203	ENSG00000117305
ENSG00000169756	ENSG00000099812	ENSG00000116691
ENSG00000104756	ENSG00000008513	ENSG00000114861
ENSG00000156508	ENSG00000138075	ENSG00000114316
ENSG00000167800	ENSG00000151892	ENSG00000129173
ENSG00000233488	ENSG00000185787	ENSG00000128815
ENSG00000137392	ENSG00000103150	ENSG00000179168
ENSG00000163386	ENSG00000164100	ENSG00000172817
ENSG00000198374	ENSG00000176742	ENSG00000160410
ENSG00000132664	ENSG00000159455	ENSG00000164532
ENSG00000163686	ENSG00000142515	ENSG00000012504
ENSG00000147113	ENSG00000004660	ENSG00000143507
ENSG00000219438	ENSG00000225697	ENSG00000120669
ENSG00000016864	ENSG00000147457	ENSG00000156009
ENSG00000088833	ENSG00000165568	ENSG00000064490
ENSG00000163684	ENSG00000178921	ENSG00000171872
ENSG00000103811	ENSG00000138468	ENSG00000164077
ENSG00000204060	ENSG00000164080	ENSG00000101842
ENSG00000146469	ENSG00000198366	ENSG00000088888
ENSG00000251369	ENSG00000162763	ENSG00000128253
ENSG00000114248	ENSG00000258724	ENSG00000087086
ENSG00000162039	ENSG00000138617	ENSG00000134809
ENSG00000163106	ENSG00000025434	ENSG00000163704
ENSG00000112964	ENSG00000141736	ENSG00000154556
ENSG00000186393	ENSG00000106078	ENSG00000196712
ENSG00000065665	ENSG00000135069	ENSG00000165338
ENSG00000174885	ENSG00000255800	ENSG00000109424
ENSG00000164414	ENSG00000242498	ENSG00000180096
ENSG00000100030	ENSG00000172772	ENSG00000165282
ENSG00000141376	ENSG00000157601	ENSG00000119725
ENSG00000204410	ENSG00000133243	ENSG00000147697
ENSG00000112110	ENSG00000136950	ENSG00000146414
ENSG00000180881	ENSG00000169372	ENSG00000140534
ENSG00000140450	ENSG00000056998	ENSG00000129538
ENSG00000160113	ENSG00000182611	ENSG00000138592
ENSG00000121380	ENSG00000102081	ENSG00000139998
ENSG00000182827	ENSG00000078898	ENSG00000115808
ENSG00000164221	ENSG00000100201	ENSG00000106605
ENSG00000125245	ENSG00000100228	ENSG00000185825
ENSG00000175416	ENSG00000196177	ENSG00000010404
ENSG00000149357	ENSG00000131016	ENSG00000094914
ENSG00000185306	ENSG00000166578	ENSG00000204361
ENSG00000177971	ENSG00000103642	ENSG00000166862
ENSG00000158417	ENSG00000176567	ENSG00000115207
ENSG00000188612	ENSG00000148948	ENSG00000065883
ENSG00000071655	ENSG00000179218	ENSG00000007202
ENSG00000215474	ENSG00000198429	ENSG00000088756
ENSG00000173621	ENSG00000142632	ENSG00000135929
ENSG00000157326	ENSG00000101152	ENSG00000170396
ENSG00000161813	ENSG00000131738	ENSG00000204711
ENSG00000152404	ENSG00000075151	ENSG00000169379
ENSG00000155087	ENSG00000112697	ENSG00000204278
ENSG00000105737	ENSG00000100307	ENSG00000112936

ENSG00000162623	ENSG00000179262	ENSG00000120162
ENSG00000105171	ENSG00000176393	ENSG00000198932
ENSG00000166532	ENSG00000113851	ENSG00000137073
ENSG00000269049	ENSG00000204195	ENSG00000134259
ENSG00000123407	ENSG00000181991	ENSG00000158062
ENSG00000168131	ENSG00000188176	ENSG00000188886
ENSG00000139053	ENSG00000140876	ENSG00000196475
ENSG00000115998	ENSG00000151229	ENSG00000132463
ENSG00000170310	ENSG00000011376	ENSG00000178177
ENSG00000240972	ENSG00000103174	ENSG00000177628
ENSG00000164713	ENSG00000148057	ENSG00000072210
ENSG00000196754	ENSG00000128203	ENSG00000174059
ENSG00000137575	ENSG00000102904	ENSG00000113734
ENSG00000121211	ENSG00000142675	ENSG00000151360
ENSG00000181350	ENSG00000029363	ENSG00000108823
ENSG00000148175	ENSG00000146476	ENSG00000167981
ENSG00000133731	ENSG00000143258	ENSG00000075234
ENSG00000106404	ENSG00000180090	ENSG00000067066
ENSG00000254901	ENSG00000130254	ENSG00000241794
ENSG00000174225	ENSG00000012822	ENSG00000111321
ENSG00000140993	ENSG00000137265	ENSG00000146013
ENSG00000237353	ENSG00000070985	ENSG00000102349
ENSG00000122694	ENSG00000100982	ENSG00000138696
ENSG00000100316	ENSG00000243279	ENSG00000100583
ENSG00000164970	ENSG00000105479	ENSG00000198028
ENSG00000198807	ENSG00000198822	ENSG00000166451
ENSG00000189377	ENSG00000163394	ENSG00000197417
ENSG00000120137	ENSG00000101210	ENSG00000159763
ENSG00000109065	ENSG00000176198	ENSG00000084734
ENSG00000166086	ENSG00000166762	ENSG00000128394
ENSG00000176595	ENSG00000145192	ENSG00000114423
ENSG00000169155	ENSG00000213218	ENSG00000095587
ENSG00000149635	ENSG00000166471	ENSG00000137812
ENSG00000181038	ENSG00000256591	ENSG00000258529
ENSG00000244274	ENSG00000171115	ENSG00000187695
ENSG00000173991	ENSG00000196678	ENSG00000136045
ENSG00000204257	ENSG00000067208	ENSG00000167460
ENSG00000204790	ENSG00000177302	ENSG00000161021
ENSG00000148288	ENSG00000155980	ENSG00000198176
ENSG00000147873	ENSG00000065135	ENSG00000171604
ENSG00000197576	ENSG00000134070	ENSG00000156639
ENSG00000125538	ENSG00000144381	ENSG00000135063
ENSG00000074317	ENSG00000134987	ENSG00000085465
ENSG00000158691	ENSG00000153230	ENSG00000165868
ENSG00000188991	ENSG00000120160	ENSG00000078098
ENSG00000115514	ENSG00000119927	ENSG00000150347
ENSG00000085999	ENSG00000113645	ENSG00000204510
ENSG00000102359	ENSG00000105369	ENSG00000136939
ENSG00000164743	ENSG00000166046	ENSG00000187607
ENSG00000100330	ENSG00000168056	ENSG00000123838
ENSG00000134748	ENSG00000197177	ENSG00000054611
ENSG00000205971	ENSG00000169131	ENSG00000255104
ENSG00000258818	ENSG00000009765	ENSG00000003756
ENSG00000186994	ENSG00000100558	ENSG00000179088
ENSG00000104221	ENSG00000165643	ENSG00000131269
ENSG00000119801	ENSG00000174485	ENSG00000185513
ENSG00000132128	ENSG00000174963	ENSG00000066923
ENSG00000163541	ENSG00000188508	ENSG00000223547
ENSG00000204007	ENSG00000102302	ENSG00000081051
ENSG00000163961	ENSG00000166091	ENSG00000187118
ENSG00000040531	ENSG00000174473	ENSG00000102239
ENSG00000186094	ENSG00000122420	ENSG00000197721
ENSG00000175911	ENSG00000129990	ENSG00000173926
ENSG00000149948	ENSG00000214102	ENSG00000183747

ENSG00000179630	ENSG00000181001	ENSG00000186298
ENSG00000134109	ENSG00000137726	ENSG00000125498
ENSG00000173218	ENSG00000152457	ENSG00000068120
ENSG00000185432	ENSG00000144554	ENSG00000244255
ENSG00000142599	ENSG00000099958	ENSG00000156011
ENSG00000113578	ENSG00000163171	ENSG00000176406
ENSG00000196683	ENSG00000187726	ENSG00000121579
ENSG00000172116	ENSG00000163798	ENSG00000008311
ENSG00000100162	ENSG00000132321	ENSG00000249481
ENSG00000106399	ENSG00000090905	ENSG00000167851
ENSG00000085274	ENSG00000112159	ENSG00000224586
ENSG00000135175	ENSG00000064225	ENSG00000100626
ENSG00000243678	ENSG00000170370	ENSG00000118965
ENSG00000258388	ENSG00000182983	ENSG00000205642
ENSG00000196937	ENSG00000149257	ENSG00000102103
ENSG00000112146	ENSG00000221813	ENSG00000160862
ENSG00000088926	ENSG00000167380	ENSG00000135976
ENSG00000178932	ENSG00000107954	ENSG00000075188
ENSG00000205544	ENSG00000117394	ENSG00000134627
ENSG00000180934	ENSG00000172519	ENSG00000147133
ENSG00000157036	ENSG00000250741	ENSG00000033050
ENSG00000008438	ENSG00000182872	ENSG00000168070
ENSG00000164816	ENSG00000165702	ENSG00000101911
ENSG00000105255	ENSG00000221937	ENSG00000105929
ENSG00000165475	ENSG00000243709	ENSG00000105723
ENSG00000151224	ENSG00000116824	ENSG00000113916
ENSG00000105270	ENSG00000175322	ENSG00000166313
ENSG00000155367	ENSG00000164663	ENSG00000129214
ENSG00000179909	ENSG00000170128	ENSG00000105559
ENSG00000087510	ENSG00000122824	ENSG00000123612
ENSG00000179855	ENSG00000005381	ENSG00000159882
ENSG00000204140	ENSG00000124570	ENSG00000170790
ENSG00000131094	ENSG00000156261	ENSG00000142065
ENSG00000111247	ENSG00000111666	ENSG00000120318
ENSG00000178502	ENSG00000119285	ENSG00000171133
ENSG00000143252	ENSG00000085872	ENSG00000135917
ENSG00000126010	ENSG00000155287	ENSG00000147381
ENSG00000164120	ENSG00000196966	ENSG00000154162
ENSG00000170373	ENSG00000184060	ENSG00000186967
ENSG00000124383	ENSG00000136247	ENSG00000033627
ENSG00000163738	ENSG00000135740	ENSG00000143303
ENSG00000205302	ENSG00000256294	ENSG00000254772
ENSG00000113583	ENSG00000124449	ENSG00000179954
ENSG00000204642	ENSG00000181291	ENSG00000168621
ENSG00000132313	ENSG00000174004	ENSG00000171570
ENSG00000101935	ENSG00000108753	ENSG00000162368
ENSG00000167531	ENSG00000122971	ENSG00000010379
ENSG00000196405	ENSG00000130175	ENSG00000072849
ENSG00000197747	ENSG00000173992	ENSG00000184254
ENSG00000104131	ENSG00000132199	ENSG00000176571
ENSG00000196290	ENSG00000066336	ENSG00000136237
ENSG00000116329	ENSG00000169896	ENSG00000180787
ENSG00000215788	ENSG00000179889	ENSG00000171049
ENSG00000157227	ENSG00000160683	ENSG00000080503
ENSG00000066405	ENSG00000213401	ENSG00000139223
ENSG00000124678	ENSG00000125124	ENSG00000197651
ENSG00000112308	ENSG00000170242	ENSG00000185219
ENSG00000139517	ENSG00000183206	ENSG00000137204
ENSG00000138772	ENSG00000164877	ENSG00000075643
ENSG00000197785	ENSG00000181541	ENSG00000112303
ENSG00000139410	ENSG00000116721	ENSG00000094880
ENSG00000237388	ENSG00000123240	ENSG00000171855
ENSG00000187080	ENSG00000138587	ENSG00000113389
ENSG00000170638	ENSG00000123307	ENSG00000105697

ENSG00000143436	ENSG00000138670	ENSG00000139625
ENSG00000121289	ENSG00000178184	ENSG00000228144
ENSG00000124827	ENSG00000134480	ENSG00000164334
ENSG00000239900	ENSG00000079277	ENSG00000215568
ENSG00000160446	ENSG00000186943	ENSG00000133048
ENSG00000166484	ENSG00000013619	ENSG00000125952
ENSG00000239605	ENSG00000095906	ENSG00000113272
ENSG00000120656	ENSG00000165349	ENSG00000186970
ENSG00000196661	ENSG00000125864	ENSG00000121903
ENSG00000255730	ENSG00000164283	ENSG00000072864
ENSG00000118454	ENSG00000167910	ENSG00000140332
ENSG00000100650	ENSG00000225830	ENSG00000065328
ENSG00000222038	ENSG00000121236	ENSG00000138798
ENSG00000213402	ENSG00000122735	ENSG00000048162
ENSG00000221909	ENSG00000088386	ENSG00000168995
ENSG00000215012	ENSG00000186009	ENSG00000145860
ENSG00000166135	ENSG00000107789	ENSG00000164430
ENSG00000259141	ENSG00000023171	ENSG00000011275
ENSG00000100401	ENSG00000110536	ENSG00000142207
ENSG00000177548	ENSG00000018408	ENSG00000161574
ENSG00000150361	ENSG00000172487	ENSG00000204149
ENSG00000164197	ENSG00000125834	ENSG00000112234
ENSG00000106686	ENSG00000171777	ENSG00000178852
ENSG00000196335	ENSG00000022567	ENSG00000187180
ENSG00000167491	ENSG00000162391	ENSG00000132677
ENSG00000124208	ENSG00000154914	ENSG00000089123
ENSG00000205084	ENSG00000072315	ENSG00000177791
ENSG00000179921	ENSG00000111701	ENSG00000188112
ENSG00000137713	ENSG00000171056	ENSG00000140199
ENSG00000204540	ENSG00000100060	ENSG00000187800
ENSG00000168522	ENSG00000127586	ENSG00000152377
ENSG00000187902	ENSG00000170961	ENSG00000177675
ENSG00000112701	ENSG00000179456	ENSG00000101162
ENSG00000152760	ENSG00000167554	ENSG00000168830
ENSG00000160298	ENSG00000117425	ENSG00000107036
ENSG00000167755	ENSG00000115841	ENSG00000189195
ENSG00000206107	ENSG00000088035	ENSG00000184838
ENSG00000176978	ENSG00000124091	ENSG00000005889
ENSG00000179938	ENSG00000118520	ENSG00000036473
ENSG00000212935	ENSG00000093010	ENSG00000089053
ENSG00000113971	ENSG00000152779	ENSG00000132781
ENSG00000174529	ENSG00000154114	ENSG00000168930
ENSG00000167635	ENSG00000185010	ENSG00000164466
ENSG00000101247	ENSG00000111181	ENSG00000174460
ENSG00000198930	ENSG00000171459	ENSG00000147394
ENSG00000233539	ENSG00000104419	ENSG00000135823
ENSG00000136104	ENSG00000095397	ENSG00000147509
ENSG00000203965	ENSG00000105146	ENSG00000242732
ENSG00000125144	ENSG00000136839	ENSG00000133313
ENSG00000183793	ENSG00000156150	ENSG00000166266
ENSG00000161944	ENSG00000151148	ENSG00000130595
ENSG00000197933	ENSG00000169918	ENSG00000144136
ENSG00000187658	ENSG00000121905	ENSG00000134318
ENSG00000187823	ENSG00000019505	ENSG00000131791
ENSG00000165672	ENSG00000181027	ENSG00000105371
ENSG00000115325	ENSG00000198851	ENSG00000185666
ENSG00000205002	ENSG00000176732	ENSG00000140093
ENSG00000102886	ENSG00000162885	ENSG00000177156
ENSG00000101892	ENSG00000186190	ENSG00000112118
ENSG00000123609	ENSG00000142453	ENSG00000070759
ENSG00000255874	ENSG00000169297	ENSG00000183323
ENSG00000197128	ENSG00000147813	ENSG00000121570
ENSG00000140285	ENSG00000166159	ENSG00000204217
ENSG00000131080	ENSG00000161654	ENSG00000113360

ENSG00000163528	ENSG00000100055	ENSG00000138074
ENSG00000090989	ENSG00000132970	ENSG00000123700
ENSG00000143811	ENSG00000175727	ENSG00000203663
ENSG00000183423	ENSG00000140859	ENSG00000116996
ENSG00000196990	ENSG00000146535	ENSG00000111981
ENSG00000102996	ENSG00000174562	ENSG00000198815
ENSG00000181007	ENSG00000170160	ENSG00000160219
ENSG00000182974	ENSG00000175497	ENSG00000137103
ENSG00000165606	ENSG00000140519	ENSG00000187492
ENSG00000105982	ENSG00000100146	ENSG00000162769
ENSG00000255524	ENSG00000137267	ENSG00000205869
ENSG00000173083	ENSG00000197706	ENSG00000152359
ENSG00000186509	ENSG00000116218	ENSG00000143437
ENSG00000077254	ENSG00000152705	ENSG00000135409
ENSG00000006652	ENSG00000070061	ENSG00000163510
ENSG00000172155	ENSG00000001626	ENSG00000158481
ENSG00000120709	ENSG00000125798	ENSG00000069702
ENSG00000176358	ENSG00000100426	ENSG00000152104
ENSG00000157368	ENSG00000182718	ENSG00000120696
ENSG00000176124	ENSG00000261949	ENSG00000128829
ENSG00000198042	ENSG00000117625	ENSG00000106069
ENSG00000122707	ENSG00000142459	ENSG00000183389
ENSG00000173960	ENSG00000138796	ENSG00000114670
ENSG00000224916	ENSG00000166681	ENSG00000183313
ENSG00000051180	ENSG00000170633	ENSG00000139973
ENSG00000216921	ENSG00000157379	ENSG00000206561
ENSG00000113318	ENSG00000119408	ENSG00000197157
ENSG00000106443	ENSG00000049239	ENSG00000167207
ENSG00000107859	ENSG00000144837	ENSG00000050748
ENSG00000177994	ENSG00000100038	ENSG00000116726
ENSG00000160051	ENSG00000187173	ENSG00000135773
ENSG00000169989	ENSG00000127928	ENSG00000150637
ENSG00000186111	ENSG00000115594	ENSG00000188786
ENSG00000213614	ENSG00000091436	ENSG00000221818
ENSG00000118680	ENSG00000139445	ENSG00000174428
ENSG00000129455	ENSG00000169594	ENSG00000133106
ENSG00000122122	ENSG00000109099	ENSG00000049883
ENSG00000184909	ENSG00000186300	ENSG00000166477
ENSG00000164306	ENSG00000198833	ENSG00000145198
ENSG00000104064	ENSG00000107937	ENSG00000166685
ENSG00000204172	ENSG00000183955	ENSG00000149646
ENSG00000168061	ENSG00000150991	ENSG00000080200
ENSG00000119326	ENSG00000196565	ENSG00000141404
ENSG00000184344	ENSG00000119723	ENSG00000164967
ENSG00000134716	ENSG00000198585	ENSG00000180043
ENSG00000166233	ENSG00000123836	ENSG00000084092
ENSG00000117036	ENSG00000134278	ENSG00000108883
ENSG00000126261	ENSG00000141437	ENSG00000086758
ENSG00000177511	ENSG00000065609	ENSG00000175329
ENSG00000173612	ENSG00000138135	ENSG00000188687
ENSG00000241241	ENSG00000197976	ENSG00000162819
ENSG00000140600	ENSG00000132326	ENSG00000143554
ENSG00000138472	ENSG00000068323	ENSG00000022976
ENSG00000112039	ENSG00000100116	ENSG00000110195
ENSG00000158815	ENSG00000214711	ENSG00000163606
ENSG00000123999	ENSG00000111110	ENSG00000106536
ENSG00000225968	ENSG00000107890	ENSG00000137752
ENSG00000111912	ENSG00000119599	ENSG00000163788
ENSG00000115267	ENSG00000186364	ENSG00000205045
ENSG00000256861	ENSG00000142961	ENSG00000186867
ENSG00000104529	ENSG00000085491	ENSG00000087842
ENSG00000256087	ENSG00000186583	ENSG00000254656
ENSG00000186458	ENSG00000169154	ENSG00000185985
ENSG00000168546	ENSG00000164107	ENSG00000157653

ENSG00000112245	ENSG00000173258	ENSG00000184886
ENSG00000077274	ENSG00000099795	ENSG00000115677
ENSG00000166923	ENSG00000137275	ENSG00000100938
ENSG00000198522	ENSG00000206432	ENSG00000186854
ENSG00000162129	ENSG00000168781	ENSG00000112511
ENSG00000187994	ENSG00000165059	ENSG00000116885
ENSG00000196440	ENSG00000101290	ENSG00000198821
ENSG00000100353	ENSG00000054598	ENSG00000132670
ENSG00000264058	ENSG00000189001	ENSG00000144504
ENSG00000137259	ENSG00000197746	ENSG00000143318
ENSG00000258659	ENSG00000029153	ENSG00000144339
ENSG00000213523	ENSG00000007372	ENSG00000165678
ENSG00000254087	ENSG00000185917	ENSG00000127955
ENSG00000090339	ENSG00000177947	ENSG00000134283
ENSG00000139620	ENSG00000101868	ENSG00000149507
ENSG00000130830	ENSG00000070785	ENSG00000262355
ENSG00000214013	ENSG00000112337	ENSG00000186487
ENSG00000171858	ENSG00000135052	ENSG00000068796
ENSG00000197263	ENSG00000109775	ENSG00000028277
ENSG00000184979	ENSG00000109654	ENSG00000110975
ENSG00000185619	ENSG00000160753	ENSG00000143105
ENSG00000184209	ENSG00000135443	ENSG00000136573
ENSG00000168256	ENSG00000140987	ENSG00000129450
ENSG00000167261	ENSG00000101812	ENSG00000089597
ENSG00000138449	ENSG00000148704	ENSG00000095203
ENSG00000135378	ENSG00000183077	ENSG00000169432
ENSG00000130592	ENSG00000115806	ENSG00000137486
ENSG00000089220	ENSG00000244607	ENSG00000138686
ENSG00000232859	ENSG00000089351	ENSG00000159921
ENSG00000131669	ENSG00000177663	ENSG00000180071
ENSG00000168904	ENSG00000144410	ENSG00000158270
ENSG00000178573	ENSG00000177283	ENSG00000133226
ENSG00000174744	ENSG00000163297	ENSG00000152910
ENSG00000205155	ENSG00000104973	ENSG00000186017
ENSG00000196859	ENSG00000006025	ENSG00000198108
ENSG00000133131	ENSG00000125872	ENSG00000142698
ENSG00000103549	ENSG00000198919	ENSG00000118200
ENSG00000205269	ENSG00000251493	ENSG00000146385
ENSG00000205497	ENSG00000176092	ENSG00000031698
ENSG00000148343	ENSG00000166351	ENSG00000080608
ENSG00000185105	ENSG00000180626	ENSG00000169679
ENSG00000180389	ENSG00000152086	ENSG00000198445
ENSG00000186710	ENSG00000185974	ENSG00000166140
ENSG00000157212	ENSG00000146378	ENSG00000130150
ENSG00000104687	ENSG00000120280	ENSG00000242715
ENSG00000105048	ENSG00000130193	ENSG00000172689
ENSG00000186020	ENSG00000105989	ENSG00000125772
ENSG00000110077	ENSG00000137310	ENSG00000166979
ENSG00000010244	ENSG00000169660	ENSG00000145649
ENSG00000108061	ENSG00000166311	ENSG00000100225
ENSG00000135632	ENSG00000157833	ENSG00000064601
ENSG00000115137	ENSG00000119973	ENSG00000143257
ENSG00000116032	ENSG00000111339	ENSG00000196407
ENSG00000186910	ENSG00000050405	ENSG00000100099
ENSG00000125508	ENSG00000067191	ENSG00000049167
ENSG00000111215	ENSG00000010671	ENSG00000138382
ENSG00000119772	ENSG00000122136	ENSG00000106052
ENSG00000078902	ENSG00000146112	ENSG00000110848
ENSG00000198835	ENSG00000088682	ENSG00000135912
ENSG00000182446	ENSG00000105855	ENSG00000114209
ENSG00000178125	ENSG00000176903	ENSG00000170153
ENSG00000035928	ENSG00000131981	ENSG00000133816
ENSG00000116455	ENSG00000099810	ENSG00000188372
ENSG00000146410	ENSG00000161609	ENSG00000188760

ENSG00000111684	ENSG00000162738	ENSG00000131473
ENSG00000121454	ENSG00000116117	ENSG00000177294
ENSG00000164406	ENSG00000183778	ENSG00000111012
ENSG00000161267	ENSG00000120093	ENSG00000175548
ENSG00000105819	ENSG00000198759	ENSG00000142687
ENSG00000188408	ENSG00000198315	ENSG00000261934
ENSG00000094755	ENSG00000166704	ENSG00000154240
ENSG00000240230	ENSG00000077420	ENSG00000149806
ENSG00000172789	ENSG00000121895	ENSG00000108509
ENSG00000185453	ENSG00000170465	ENSG00000160294
ENSG00000149716	ENSG00000126266	ENSG00000164930
ENSG00000100243	ENSG00000167566	ENSG00000029559
ENSG00000060982	ENSG00000186532	ENSG00000167822
ENSG00000107833	ENSG00000158816	ENSG00000166341
ENSG00000112984	ENSG00000110881	ENSG00000184933
ENSG00000167281	ENSG00000027697	ENSG00000183150
ENSG00000103569	ENSG00000011451	ENSG00000178187
ENSG00000180370	ENSG00000081692	ENSG00000181788
ENSG00000184557	ENSG00000079819	ENSG00000111863
ENSG00000105711	ENSG00000010278	ENSG00000163913
ENSG00000145996	ENSG00000044012	ENSG00000138642
ENSG00000111850	ENSG00000141034	ENSG00000254505
ENSG00000096092	ENSG00000155495	ENSG00000134042
ENSG00000168028	ENSG00000113719	ENSG00000152207
ENSG00000162910	ENSG00000114745	ENSG00000182247
ENSG00000117215	ENSG00000131732	ENSG00000109061
ENSG00000174876	ENSG00000131037	ENSG00000006740
ENSG00000177706	ENSG00000100029	ENSG00000203668
ENSG00000215217	ENSG00000165269	ENSG00000234127
ENSG00000175105	ENSG00000172426	ENSG00000054392
ENSG00000185942	ENSG00000204687	ENSG00000160191
ENSG00000133114	ENSG00000243978	ENSG00000095787
ENSG00000143194	ENSG00000128340	ENSG00000144645
ENSG00000126861	ENSG00000135083	ENSG00000177689
ENSG00000115234	ENSG00000137413	ENSG00000140575
ENSG00000145016	ENSG00000100292	ENSG00000243896
ENSG00000129534	ENSG00000169744	ENSG00000113282
ENSG00000164611	ENSG00000105664	ENSG00000103035
ENSG00000253350	ENSG00000144278	ENSG00000111271
ENSG00000155096	ENSG00000083817	ENSG00000167525
ENSG00000116771	ENSG00000164548	ENSG00000120254
ENSG00000161281	ENSG00000197111	ENSG00000174405
ENSG00000106609	ENSG00000103423	ENSG00000141338
ENSG00000186187	ENSG00000188404	ENSG00000060237
ENSG00000188523	ENSG00000165995	ENSG00000164045
ENSG00000163558	ENSG00000103091	ENSG00000172113
ENSG00000133639	ENSG00000125354	ENSG00000174450
ENSG00000198040	ENSG00000175189	ENSG00000146021
ENSG00000109846	ENSG00000137203	ENSG00000084652
ENSG00000008324	ENSG00000187144	ENSG00000172073
ENSG00000173638	ENSG00000169641	ENSG00000175426
ENSG00000143443	ENSG00000256642	ENSG00000140067
ENSG00000173715	ENSG00000110786	ENSG00000243543
ENSG00000125818	ENSG00000213088	ENSG00000113108
ENSG00000106803	ENSG00000168267	ENSG00000104490
ENSG00000088298	ENSG00000151690	ENSG00000132631
ENSG00000204991	ENSG00000111412	ENSG00000114993
ENSG00000145214	ENSG00000139722	ENSG00000163374
ENSG00000176979	ENSG00000154258	ENSG00000007350
ENSG00000107738	ENSG00000108352	ENSG00000169925
ENSG00000134138	ENSG00000145284	ENSG00000111644
ENSG00000135334	ENSG00000156925	ENSG00000179979
ENSG00000181418	ENSG00000188342	ENSG00000156232
ENSG00000104859	ENSG00000124575	ENSG00000166845

ENSG00000243480	ENSG00000065600	ENSG00000075223
ENSG00000087301	ENSG00000096384	ENSG00000188554
ENSG00000203910	ENSG00000140416	ENSG00000188282
ENSG00000186860	ENSG00000105894	ENSG00000124812
ENSG00000047410	ENSG00000138085	ENSG00000066855
ENSG00000196570	ENSG00000105699	ENSG00000140795
ENSG00000184544	ENSG00000100600	ENSG00000034533
ENSG00000102781	ENSG00000168703	ENSG00000034693
ENSG00000169884	ENSG00000182968	ENSG00000172289
ENSG00000134330	ENSG00000010810	ENSG00000130957
ENSG00000147050	ENSG00000042062	ENSG00000147604
ENSG00000204920	ENSG00000153885	ENSG00000111254
ENSG00000151353	ENSG00000143344	ENSG00000198771
ENSG00000214642	ENSG00000188522	ENSG00000070087
ENSG00000135525	ENSG00000115607	ENSG00000197077
ENSG00000150048	ENSG00000079134	ENSG00000213782
ENSG00000153406	ENSG00000132471	ENSG00000167535
ENSG00000177106	ENSG00000119242	ENSG00000185482
ENSG00000117280	ENSG00000198561	ENSG00000254206
ENSG00000225921	ENSG00000104960	ENSG00000049768
ENSG00000172081	ENSG00000240771	ENSG00000122585
ENSG00000125861	ENSG00000160781	ENSG00000169347
ENSG00000154262	ENSG00000064763	ENSG00000135622
ENSG00000166136	ENSG00000170608	ENSG00000167815
ENSG00000111291	ENSG00000168878	ENSG00000138439
ENSG00000103266	ENSG00000140694	ENSG00000106328
ENSG00000181518	ENSG00000181555	ENSG00000147155
ENSG00000104921	ENSG00000144815	ENSG00000010803
ENSG00000115211	ENSG00000129968	ENSG00000259207
ENSG00000173250	ENSG00000143867	ENSG00000107779
ENSG00000166260	ENSG00000179213	ENSG00000182957
ENSG00000093000	ENSG00000159314	ENSG00000073578
ENSG00000173451	ENSG00000160838	ENSG00000019549
ENSG00000203876	ENSG00000087274	ENSG00000242419
ENSG00000105443	ENSG00000168710	ENSG00000170832
ENSG00000147885	ENSG00000161040	ENSG00000180818
ENSG00000100336	ENSG00000172482	ENSG00000072518
ENSG00000174173	ENSG00000231738	ENSG00000080603
ENSG00000104537	ENSG00000111245	ENSG00000118514
ENSG00000188580	ENSG00000107957	ENSG00000096696
ENSG00000183087	ENSG00000176171	ENSG00000204104
ENSG00000181873	ENSG00000268950	ENSG00000133246
ENSG00000166323	ENSG00000104976	ENSG00000155849
ENSG00000164253	ENSG00000023902	ENSG00000183090
ENSG00000158480	ENSG00000128011	ENSG00000114933
ENSG00000163534	ENSG00000125657	ENSG00000187554
ENSG00000181552	ENSG00000011485	ENSG00000149516
ENSG00000158457	ENSG00000167986	ENSG00000148634
ENSG00000197226	ENSG00000105329	ENSG00000155640
ENSG00000172324	ENSG00000186675	ENSG00000198732
ENSG00000187689	ENSG00000229314	ENSG00000136504
ENSG00000205592	ENSG00000054690	ENSG00000180773
ENSG00000134343	ENSG00000134444	ENSG00000176925
ENSG00000130313	ENSG00000162746	ENSG00000141161
ENSG00000084093	ENSG00000105072	ENSG00000124092
ENSG00000167765	ENSG00000062716	ENSG00000170967
ENSG00000132964	ENSG00000197150	ENSG00000115602
ENSG00000086730	ENSG00000139946	ENSG00000198121
ENSG00000213398	ENSG00000126522	ENSG00000103460
ENSG00000189362	ENSG00000234829	ENSG00000138279
ENSG00000139428	ENSG00000114867	ENSG00000182158
ENSG00000070610	ENSG00000140839	ENSG00000163635
ENSG00000167216	ENSG00000116127	ENSG00000173848
ENSG00000248672	ENSG00000063854	ENSG00000112182

ENSG00000133105	ENSG00000075413	ENSG00000131725
ENSG00000131019	ENSG00000166317	ENSG00000119509
ENSG00000164500	ENSG00000154305	ENSG00000109819
ENSG00000162734	ENSG00000157637	ENSG00000143376
ENSG00000120896	ENSG00000165182	ENSG00000112812
ENSG00000205307	ENSG00000153574	ENSG00000145103
ENSG00000112212	ENSG00000117222	ENSG00000100568
ENSG00000112787	ENSG00000135956	ENSG00000132669
ENSG00000161647	ENSG00000154277	ENSG00000139211
ENSG00000110243	ENSG00000158887	ENSG00000164330
ENSG00000128917	ENSG00000138658	ENSG00000105053
ENSG00000111716	ENSG00000154930	ENSG00000132139
ENSG00000166750	ENSG00000158901	ENSG00000044115
ENSG00000113575	ENSG00000204536	ENSG00000143921
ENSG00000145391	ENSG00000196814	ENSG00000182359
ENSG00000173401	ENSG00000176601	ENSG00000115484
ENSG00000204561	ENSG00000157335	ENSG00000187003
ENSG00000268975	ENSG00000127990	ENSG00000165959
ENSG00000025796	ENSG00000144468	ENSG00000096006
ENSG00000157734	ENSG00000187672	ENSG00000204764
ENSG00000057757	ENSG00000203666	ENSG00000134588
ENSG00000050393	ENSG00000116830	ENSG00000168646
ENSG00000100749	ENSG00000004809	ENSG00000073754
ENSG00000131759	ENSG00000164047	ENSG00000135476
ENSG00000186599	ENSG00000166851	ENSG00000122966
ENSG00000196470	ENSG00000181355	ENSG00000125355
ENSG00000162694	ENSG00000138315	ENSG00000155886
ENSG00000151572	ENSG00000166508	ENSG00000113580
ENSG00000162723	ENSG00000136193	ENSG00000146826
ENSG00000119782	ENSG00000170777	ENSG00000079689
ENSG00000106012	ENSG00000077684	ENSG00000071054
ENSG00000268334	ENSG00000158258	ENSG00000197168
ENSG00000167711	ENSG00000158955	ENSG00000005471
ENSG00000110628	ENSG00000181499	ENSG00000129353
ENSG00000131808	ENSG00000100422	ENSG00000161807
ENSG00000120664	ENSG00000061492	ENSG00000139546
ENSG00000115816	ENSG00000167595	ENSG00000154975
ENSG00000082497	ENSG00000125409	ENSG00000006282
ENSG00000163930	ENSG00000172818	ENSG00000146070
ENSG00000155959	ENSG00000182261	ENSG00000167632
ENSG00000014138	ENSG00000185386	ENSG00000188993
ENSG00000104805	ENSG00000162493	ENSG00000164329
ENSG00000084733	ENSG00000128408	ENSG00000070371
ENSG00000163825	ENSG00000130511	ENSG00000038945
ENSG00000163389	ENSG00000133315	ENSG00000177239
ENSG00000183878	ENSG00000163376	ENSG00000013573
ENSG00000154719	ENSG00000197622	ENSG00000139197
ENSG00000185133	ENSG00000197183	ENSG00000121691
ENSG00000151164	ENSG00000173894	ENSG00000115616
ENSG00000136110	ENSG00000152969	ENSG00000204681
ENSG00000136146	ENSG00000006377	ENSG00000174343
ENSG00000005448	ENSG00000117308	ENSG00000163380
ENSG00000105607	ENSG00000085377	ENSG00000093072
ENSG00000175283	ENSG00000111348	ENSG00000005955
ENSG00000180176	ENSG00000099783	ENSG00000105383
ENSG00000023608	ENSG00000007968	ENSG00000134256
ENSG00000136758	ENSG00000102606	ENSG00000182902
ENSG00000185885	ENSG00000141644	ENSG00000144583
ENSG00000105974	ENSG00000135631	ENSG00000175262
ENSG00000221963	ENSG00000139287	ENSG00000124215
ENSG00000132906	ENSG00000253159	ENSG00000065882
ENSG00000160075	ENSG00000189375	ENSG00000134198
ENSG00000214891	ENSG00000240764	ENSG00000159082
ENSG00000149823	ENSG00000139746	ENSG00000146276

ENSG00000215298	ENSG00000122121	ENSG00000131941
ENSG00000240038	ENSG00000121966	ENSG00000167601
ENSG00000163959	ENSG00000164976	ENSG00000221829
ENSG00000181264	ENSG00000214078	ENSG00000101452
ENSG00000058729	ENSG00000173611	ENSG00000168872
ENSG00000144485	ENSG00000005379	ENSG00000258366
ENSG00000187272	ENSG00000004848	ENSG00000063015
ENSG00000218739	ENSG00000136449	ENSG00000154310
ENSG00000187581	ENSG00000145730	ENSG00000040199
ENSG00000255819	ENSG00000145864	ENSG00000174948
ENSG00000135390	ENSG00000132950	ENSG00000106123
ENSG00000168172	ENSG00000173531	ENSG00000147255
ENSG00000184995	ENSG00000040341	ENSG00000108950
ENSG00000129473	ENSG00000259571	ENSG00000188385
ENSG00000267855	ENSG00000163877	ENSG00000205029
ENSG00000166889	ENSG00000183876	ENSG00000091129
ENSG00000185112	ENSG00000099960	ENSG00000147432
ENSG00000165887	ENSG00000161547	ENSG00000106927
ENSG00000121064	ENSG00000107242	ENSG00000204442
ENSG00000163646	ENSG00000187556	ENSG00000184368
ENSG00000109536	ENSG00000116266	ENSG00000144642
ENSG00000171014	ENSG00000123562	ENSG00000125900
ENSG00000169629	ENSG00000075426	ENSG00000150269
ENSG00000137561	ENSG00000018699	ENSG00000169247
ENSG00000069696	ENSG00000127191	ENSG00000115657
ENSG00000130643	ENSG00000115488	ENSG00000135720
ENSG00000113621	ENSG00000122085	ENSG00000197283
ENSG00000123349	ENSG00000012061	ENSG00000197324
ENSG00000137312	ENSG00000099822	ENSG00000061936
ENSG00000119041	ENSG00000215183	ENSG00000092036
ENSG00000150275	ENSG00000107796	ENSG00000164307
ENSG00000181039	ENSG00000143195	ENSG00000177398
ENSG00000183292	ENSG00000100441	ENSG00000143641
ENSG00000147003	ENSG00000156298	ENSG00000144868
ENSG00000161958	ENSG00000176371	ENSG00000167483
ENSG00000162643	ENSG00000071575	ENSG00000120500
ENSG00000156873	ENSG00000114770	ENSG00000188603
ENSG00000248874	ENSG00000104321	ENSG00000110887
ENSG00000204033	ENSG00000171790	ENSG00000137177
ENSG00000154814	ENSG00000076924	ENSG00000136114
ENSG00000223501	ENSG00000013275	ENSG00000074527
ENSG00000152133	ENSG00000177169	ENSG00000162641
ENSG00000164897	ENSG00000139083	ENSG00000263155
ENSG00000097046	ENSG00000164007	ENSG00000038219
ENSG00000236981	ENSG00000148110	ENSG00000165124
ENSG00000146910	ENSG00000178171	ENSG00000157617
ENSG00000171314	ENSG00000186977	ENSG00000184313
ENSG00000182645	ENSG00000141720	ENSG00000087258
ENSG00000170605	ENSG00000171501	ENSG00000133687
ENSG00000156535	ENSG00000163012	ENSG00000166946
ENSG00000158470	ENSG00000100077	ENSG00000135372
ENSG00000189326	ENSG00000205022	ENSG00000189181
ENSG00000189042	ENSG00000177646	ENSG00000101977
ENSG00000196167	ENSG00000033170	ENSG00000155363
ENSG00000171861	ENSG00000163755	ENSG00000048828
ENSG00000108528	ENSG00000132434	ENSG00000119707
ENSG00000106588	ENSG00000162236	ENSG00000112218
ENSG00000099968	ENSG00000127472	ENSG00000047365
ENSG00000188042	ENSG00000137757	ENSG00000148672
ENSG00000154945	ENSG00000073756	ENSG00000253873
ENSG00000187475	ENSG00000165970	ENSG00000174990
ENSG00000185745	ENSG00000198862	ENSG00000198758
ENSG00000138698	ENSG00000149187	ENSG00000171401
ENSG00000125534	ENSG00000109472	ENSG00000101096

ENSG00000125046	ENSG00000249967	ENSG00000170959
ENSG00000117408	ENSG00000137936	ENSG00000171505
ENSG00000188394	ENSG00000128487	ENSG00000101343
ENSG00000117410	ENSG00000179588	ENSG00000116194
ENSG00000089050	ENSG00000051620	ENSG00000186973
ENSG00000069482	ENSG00000109771	ENSG00000100504
ENSG00000159147	ENSG00000104941	ENSG00000106608
ENSG00000188738	ENSG00000186868	ENSG00000086717
ENSG00000157890	ENSG00000140688	ENSG00000149380
ENSG00000198663	ENSG00000163705	ENSG00000089639
ENSG00000108799	ENSG00000102554	ENSG00000102837
ENSG00000066185	ENSG00000115446	ENSG00000174292
ENSG00000156504	ENSG00000101421	ENSG00000132153
ENSG00000197057	ENSG00000157303	ENSG00000124608
ENSG00000203813	ENSG00000142192	ENSG00000177453
ENSG00000135346	ENSG00000068784	ENSG00000155465
ENSG00000125910	ENSG00000114200	ENSG00000204842
ENSG00000139329	ENSG00000185896	ENSG00000003393
ENSG00000122565	ENSG00000177479	ENSG00000172578
ENSG00000163159	ENSG00000119977	ENSG00000007237
ENSG00000161981	ENSG00000115363	ENSG00000197993
ENSG00000104889	ENSG00000258832	ENSG00000163681
ENSG00000164893	ENSG00000186377	ENSG00000079385
ENSG00000125845	ENSG00000155827	ENSG00000173210
ENSG00000204653	ENSG00000111728	ENSG00000176136
ENSG00000124875	ENSG00000114646	ENSG00000185420
ENSG00000205930	ENSG00000251692	ENSG00000088808
ENSG00000163382	ENSG00000116016	ENSG00000183258
ENSG00000133028	ENSG00000116903	ENSG00000136813
ENSG00000134108	ENSG00000106538	ENSG00000091106
ENSG00000185883	ENSG00000198689	ENSG00000138614
ENSG00000168569	ENSG00000185352	ENSG00000164318
ENSG00000203697	ENSG00000136536	ENSG00000197565
ENSG00000188315	ENSG00000158050	ENSG00000164509
ENSG00000119636	ENSG00000198885	ENSG00000151461
ENSG00000138381	ENSG00000110274	ENSG00000136828
ENSG00000214160	ENSG00000163214	ENSG00000136010
ENSG00000127423	ENSG00000171403	ENSG00000166603
ENSG00000186141	ENSG00000140983	ENSG00000221888
ENSG00000111640	ENSG00000104299	ENSG00000180988
ENSG00000130347	ENSG00000130640	ENSG00000155897
ENSG00000158604	ENSG00000177917	ENSG00000214215
ENSG00000176018	ENSG00000187837	ENSG00000104415
ENSG00000126775	ENSG00000011132	ENSG00000122012
ENSG00000127314	ENSG00000072133	ENSG00000104689
ENSG00000197826	ENSG00000111783	ENSG00000133019
ENSG00000204356	ENSG00000102100	ENSG00000114948
ENSG00000221858	ENSG00000172774	ENSG00000132205
ENSG00000115946	ENSG00000196586	ENSG00000196663
ENSG00000067082	ENSG00000070756	ENSG00000172175
ENSG00000214262	ENSG00000161682	ENSG00000164659
ENSG00000168374	ENSG00000255974	ENSG00000166349
ENSG00000172717	ENSG00000182901	ENSG00000186976
ENSG00000163472	ENSG00000141446	ENSG00000122367
ENSG00000142655	ENSG00000265969	ENSG00000122483
ENSG00000147262	ENSG00000103034	ENSG00000110075
ENSG00000180535	ENSG00000066468	ENSG00000118900
ENSG00000120942	ENSG00000114124	ENSG00000261832
ENSG00000106819	ENSG00000178726	ENSG00000178764
ENSG00000135736	ENSG00000085982	ENSG00000071991
ENSG00000140319	ENSG00000170412	ENSG00000085276
ENSG00000071243	ENSG00000177932	ENSG00000198883
ENSG00000115596	ENSG00000197992	ENSG00000146197
ENSG00000229377	ENSG00000197859	ENSG00000099995

ENSG00000254402	ENSG00000012223	ENSG00000174429
ENSG00000183579	ENSG00000083223	ENSG00000119650
ENSG00000103196	ENSG00000183354	ENSG00000165730
ENSG00000121274	ENSG00000147548	ENSG00000127080
ENSG00000184436	ENSG00000123358	ENSG00000141431
ENSG00000114450	ENSG00000164113	ENSG00000090863
ENSG00000136206	ENSG00000143107	ENSG00000112837
ENSG00000137040	ENSG00000174527	ENSG00000196967
ENSG00000137364	ENSG00000072071	ENSG00000204580
ENSG00000198178	ENSG00000144233	ENSG00000134389
ENSG00000164442	ENSG00000120899	ENSG00000233701
ENSG00000214842	ENSG00000124313	ENSG00000121690
ENSG00000155984	ENSG00000175073	ENSG00000105722
ENSG00000168591	ENSG00000147655	ENSG00000144290
ENSG00000116918	ENSG00000131400	ENSG00000255346
ENSG00000085871	ENSG00000181472	ENSG00000136986
ENSG00000122435	ENSG00000180104	ENSG00000156453
ENSG00000105438	ENSG00000169856	ENSG00000203805
ENSG00000176261	ENSG00000140526	ENSG00000135407
ENSG00000196550	ENSG00000243137	ENSG00000067057
ENSG00000099889	ENSG00000166803	ENSG00000188283
ENSG00000129460	ENSG00000167889	ENSG00000242110
ENSG00000196214	ENSG00000114315	ENSG00000110171
ENSG00000149761	ENSG00000147536	ENSG00000185737
ENSG00000007545	ENSG00000118495	ENSG00000153207
ENSG00000106733	ENSG00000186815	ENSG00000169836
ENSG00000196812	ENSG00000180340	ENSG00000133067
ENSG00000172493	ENSG00000140284	ENSG00000152128
ENSG00000185864	ENSG00000175264	ENSG00000133704
ENSG00000177764	ENSG00000117525	ENSG00000108292
ENSG00000152944	ENSG00000141098	ENSG00000163075
ENSG00000115252	ENSG00000111424	ENSG00000180638
ENSG00000168395	ENSG00000128886	ENSG00000167768
ENSG00000187135	ENSG00000103742	ENSG00000156475
ENSG00000116001	ENSG00000134324	ENSG00000166961
ENSG00000100605	ENSG00000183018	ENSG00000158859
ENSG00000128313	ENSG00000073711	ENSG00000052749
ENSG00000170820	ENSG00000204246	ENSG00000196767
ENSG00000227500	ENSG00000145675	ENSG00000072682
ENSG00000205106	ENSG00000166924	ENSG00000100106
ENSG00000163507	ENSG00000171135	ENSG00000162909
ENSG00000214029	ENSG00000142002	ENSG00000133116
ENSG00000139832	ENSG00000163659	ENSG00000070882
ENSG00000101608	ENSG00000142197	ENSG00000141503
ENSG00000198900	ENSG00000188987	ENSG00000066933
ENSG00000108107	ENSG00000067141	ENSG00000015479
ENSG00000140932	ENSG00000154760	ENSG00000133056
ENSG00000167996	ENSG00000167614	ENSG00000007129
ENSG00000126003	ENSG00000108370	ENSG00000169855
ENSG00000005882	ENSG00000150938	ENSG00000165548
ENSG00000121039	ENSG00000177692	ENSG00000100433
ENSG00000243646	ENSG00000168939	ENSG00000141665
ENSG00000068697	ENSG00000158286	ENSG00000145451
ENSG00000178397	ENSG00000239697	ENSG00000176177
ENSG00000142634	ENSG00000166634	ENSG00000204463
ENSG00000173198	ENSG00000133055	ENSG00000141194
ENSG00000243943	ENSG00000134812	ENSG00000173930
ENSG00000198870	ENSG00000107249	ENSG00000143178
ENSG00000204960	ENSG00000152583	ENSG00000130544
ENSG00000089127	ENSG00000170419	ENSG00000243232
ENSG00000112195	ENSG00000169777	ENSG00000140279
ENSG00000123416	ENSG00000079335	ENSG00000112333
ENSG00000203930	ENSG00000141698	ENSG00000111049
ENSG00000088448	ENSG00000128610	ENSG00000160767

ENSG00000166557	ENSG00000123144	ENSG00000182197
ENSG00000117602	ENSG00000076108	ENSG00000129465
ENSG00000165752	ENSG00000177409	ENSG00000255398
ENSG00000162600	ENSG00000127526	ENSG00000185069
ENSG00000110801	ENSG00000100242	ENSG00000088882
ENSG00000137135	ENSG00000037757	ENSG00000254585
ENSG00000114547	ENSG00000165118	ENSG00000087589
ENSG00000166426	ENSG00000168386	ENSG00000198589
ENSG00000133265	ENSG00000154175	ENSG00000079435
ENSG00000267552	ENSG00000198513	ENSG00000177990
ENSG00000206077	ENSG00000144867	ENSG00000013588
ENSG00000140905	ENSG00000176774	ENSG00000133107
ENSG00000254469	ENSG00000157933	ENSG00000149541
ENSG00000187446	ENSG00000091137	ENSG00000204962
ENSG00000177854	ENSG00000136488	ENSG00000166947
ENSG00000187268	ENSG00000110047	ENSG00000008197
ENSG00000059728	ENSG00000243811	ENSG00000089169
ENSG00000171234	ENSG00000126262	ENSG00000166278
ENSG00000262874	ENSG00000124357	ENSG00000154864
ENSG00000150051	ENSG00000162877	ENSG00000122965
ENSG00000177425	ENSG00000188859	ENSG00000108671
ENSG00000177034	ENSG00000164690	ENSG00000025039
ENSG00000149443	ENSG00000151967	ENSG00000183765
ENSG00000164326	ENSG00000153347	ENSG00000132437
ENSG00000106591	ENSG00000182346	ENSG00000102230
ENSG00000138814	ENSG00000198646	ENSG00000088827
ENSG00000198327	ENSG00000168675	ENSG00000139767
ENSG00000166407	ENSG00000065357	ENSG00000170920
ENSG00000183153	ENSG00000132259	ENSG00000100505
ENSG00000181826	ENSG00000162601	ENSG00000144674
ENSG00000059122	ENSG00000175203	ENSG00000239264
ENSG00000186431	ENSG00000159399	ENSG00000058668
ENSG00000170634	ENSG00000197969	ENSG00000164830
ENSG00000203690	ENSG00000182575	ENSG00000086967
ENSG00000188820	ENSG00000095637	ENSG00000130985
ENSG00000170231	ENSG00000184361	ENSG00000136827
ENSG00000196693	ENSG00000196155	ENSG00000254996
ENSG00000165716	ENSG00000102174	ENSG00000188037
ENSG00000142686	ENSG00000175130	ENSG00000091986
ENSG00000205808	ENSG00000142539	ENSG00000116574
ENSG00000124107	ENSG00000115486	ENSG00000136235
ENSG00000102786	ENSG00000140455	ENSG00000173193
ENSG00000090097	ENSG00000136161	ENSG00000153066
ENSG00000106785	ENSG00000170561	ENSG00000151948
ENSG00000113456	ENSG00000085265	ENSG00000092068
ENSG00000125868	ENSG00000100379	ENSG00000159459
ENSG00000031823	ENSG00000151136	ENSG00000137944
ENSG00000160207	ENSG00000186628	ENSG00000123411
ENSG00000134248	ENSG00000173039	ENSG00000107159
ENSG00000173465	ENSG00000077463	ENSG00000147481
ENSG00000171574	ENSG00000254709	ENSG00000086015
ENSG00000149926	ENSG00000165516	ENSG00000184155
ENSG00000104267	ENSG00000121898	ENSG00000204897
ENSG00000135503	ENSG00000005073	ENSG00000124198
ENSG00000065621	ENSG00000058453	ENSG00000120705
ENSG00000147416	ENSG00000163283	ENSG00000133561
ENSG00000204694	ENSG00000148300	ENSG00000102287
ENSG00000155275	ENSG00000105618	ENSG00000167080
ENSG00000198863	ENSG00000168676	ENSG00000260238
ENSG00000178093	ENSG00000156675	ENSG00000095002
ENSG00000221823	ENSG00000173545	ENSG00000118322
ENSG00000180628	ENSG00000151079	ENSG00000079393
ENSG00000007341	ENSG00000186191	ENSG00000155659
ENSG00000168875	ENSG00000184384	ENSG00000164117

ENSG00000163249	ENSG00000167619	ENSG00000126856
ENSG00000132423	ENSG00000183134	ENSG00000112041
ENSG00000172738	ENSG00000166435	ENSG00000059691
ENSG00000153044	ENSG00000114859	ENSG00000174502
ENSG00000160999	ENSG00000132406	ENSG00000092820
ENSG00000189334	ENSG00000185926	ENSG00000073803
ENSG00000179626	ENSG00000078053	ENSG00000164946
ENSG00000198105	ENSG00000171680	ENSG00000125648
ENSG00000243414	ENSG00000184478	ENSG00000130518
ENSG00000004866	ENSG00000133065	ENSG00000072840
ENSG00000176723	ENSG00000137449	ENSG00000183706
ENSG00000113716	ENSG00000162526	ENSG00000161640
ENSG00000169599	ENSG00000172765	ENSG00000122218
ENSG00000179526	ENSG00000070961	ENSG00000130164
ENSG00000102804	ENSG00000236398	ENSG00000144057
ENSG00000134851	ENSG00000174080	ENSG00000146151
ENSG00000173077	ENSG00000131165	ENSG00000109929
ENSG00000258289	ENSG00000011426	ENSG00000124813
ENSG00000204740	ENSG00000133612	ENSG00000198074
ENSG00000196428	ENSG00000188672	ENSG00000154124
ENSG00000198841	ENSG00000203832	ENSG00000103723
ENSG00000104522	ENSG00000047579	ENSG00000115318
ENSG00000163808	ENSG00000143882	ENSG00000140678
ENSG00000173933	ENSG00000197951	ENSG00000163702
ENSG00000135605	ENSG00000120051	ENSG00000186081
ENSG00000163348	ENSG00000169344	ENSG00000221986
ENSG00000114735	ENSG00000110697	ENSG00000006116
ENSG00000128739	ENSG00000171724	ENSG00000163719
ENSG00000142945	ENSG00000093167	ENSG00000197497
ENSG00000137941	ENSG00000137338	ENSG00000162992
ENSG00000158122	ENSG00000203734	ENSG00000131379
ENSG00000183207	ENSG00000164168	ENSG00000156515
ENSG00000197016	ENSG00000250151	ENSG00000215009
ENSG00000113504	ENSG00000108839	ENSG00000008405
ENSG00000204305	ENSG00000198837	ENSG00000073598
ENSG00000135018	ENSG00000182379	ENSG00000102078
ENSG00000142252	ENSG00000198756	ENSG00000139926
ENSG00000108651	ENSG00000179295	ENSG00000198848
ENSG00000171199	ENSG00000006837	ENSG00000197381
ENSG00000186790	ENSG00000147206	ENSG00000030304
ENSG00000164879	ENSG00000241685	ENSG00000159387
ENSG00000086712	ENSG00000170044	ENSG00000198223
ENSG00000064545	ENSG00000167748	ENSG00000166265
ENSG00000066739	ENSG00000183560	ENSG00000259741
ENSG00000170322	ENSG00000164087	ENSG00000174282
ENSG00000109471	ENSG00000143294	ENSG00000184937
ENSG00000196758	ENSG00000107864	ENSG00000072062
ENSG00000187922	ENSG00000101000	ENSG00000165280
ENSG00000108424	ENSG00000241119	ENSG00000011021
ENSG00000222001	ENSG00000143226	ENSG00000153246
ENSG00000184939	ENSG00000177383	ENSG00000060749
ENSG00000139826	ENSG00000197454	ENSG00000132763
ENSG00000267673	ENSG00000120262	ENSG00000144063
ENSG00000130948	ENSG00000203757	ENSG00000243772
ENSG00000100647	ENSG00000112273	ENSG00000059573
ENSG00000196549	ENSG00000171862	ENSG00000167785
ENSG00000159692	ENSG00000127804	ENSG00000197959
ENSG00000143878	ENSG00000121578	ENSG00000143630
ENSG00000198183	ENSG00000168065	ENSG00000092758
ENSG00000198162	ENSG00000130997	ENSG00000141441
ENSG00000076351	ENSG00000121068	ENSG00000124256
ENSG00000063127	ENSG00000167578	ENSG00000166402
ENSG00000262484	ENSG00000240694	ENSG00000142556
ENSG00000180083	ENSG00000197930	ENSG00000185467

ENSG00000109107	ENSG00000148680	ENSG00000005156
ENSG00000053702	ENSG00000106636	ENSG00000090861
ENSG00000026652	ENSG00000196510	ENSG00000189280
ENSG00000120742	ENSG00000125375	ENSG00000065485
ENSG00000108176	ENSG00000163812	ENSG00000158473
ENSG00000188624	ENSG00000138029	ENSG00000110025
ENSG00000221819	ENSG00000188229	ENSG00000165630
ENSG00000181704	ENSG00000159674	ENSG00000121577
ENSG00000163013	ENSG00000184708	ENSG00000155974
ENSG00000172653	ENSG00000182944	ENSG00000164399
ENSG00000178974	ENSG00000100395	ENSG00000157510
ENSG00000156017	ENSG00000170681	ENSG00000006432
ENSG00000136826	ENSG00000149489	ENSG00000108387
ENSG00000221995	ENSG00000166090	ENSG00000258555
ENSG00000165533	ENSG00000132323	ENSG00000198373
ENSG00000204390	ENSG00000163703	ENSG00000180251
ENSG00000143198	ENSG00000103496	ENSG00000165675
ENSG00000206532	ENSG00000171517	ENSG00000203710
ENSG00000204472	ENSG00000071894	ENSG00000102195
ENSG00000139351	ENSG00000213920	ENSG00000077044
ENSG00000219626	ENSG00000100625	ENSG00000109323
ENSG00000175324	ENSG00000121807	ENSG00000147912
ENSG00000173273	ENSG00000105419	ENSG00000205339
ENSG00000248712	ENSG00000140030	ENSG00000144668
ENSG00000214860	ENSG00000182141	ENSG00000181698
ENSG00000128266	ENSG00000129682	ENSG00000144369
ENSG00000182083	ENSG00000038210	ENSG00000173757
ENSG00000075711	ENSG00000116285	ENSG00000117322
ENSG00000232629	ENSG00000105323	ENSG00000102466
ENSG00000088881	ENSG00000099956	ENSG00000172752
ENSG00000130656	ENSG00000198471	ENSG00000141027
ENSG00000025772	ENSG00000258838	ENSG00000124243
ENSG00000128567	ENSG00000091704	ENSG00000197892
ENSG00000117399	ENSG00000100448	ENSG00000120549
ENSG00000139174	ENSG00000186318	ENSG00000175387
ENSG00000112208	ENSG00000105388	ENSG00000101230
ENSG00000181163	ENSG00000108405	ENSG00000160886
ENSG00000126759	ENSG00000130638	ENSG00000148082
ENSG00000143786	ENSG00000132254	ENSG00000145833
ENSG00000022277	ENSG00000118292	ENSG00000081189
ENSG00000165832	ENSG00000103202	ENSG00000187792
ENSG00000125249	ENSG00000186472	ENSG00000130559
ENSG00000028116	ENSG00000174339	ENSG00000120659
ENSG00000103043	ENSG00000106330	ENSG00000179409
ENSG00000244094	ENSG00000159231	ENSG00000106105
ENSG00000213853	ENSG00000203989	ENSG00000019991
ENSG00000186226	ENSG00000182747	ENSG00000188779
ENSG00000179094	ENSG00000149972	ENSG00000105767
ENSG00000114115	ENSG00000170889	ENSG00000196954
ENSG00000151292	ENSG00000250349	ENSG00000107862
ENSG00000188992	ENSG00000178607	ENSG00000122557
ENSG00000221883	ENSG00000116741	ENSG00000205268
ENSG00000197536	ENSG00000141499	ENSG00000095627
ENSG00000197170	ENSG00000132464	ENSG00000100714
ENSG00000126453	ENSG00000149577	ENSG00000131778
ENSG00000152409	ENSG00000172867	ENSG00000186038
ENSG00000185129	ENSG00000139842	ENSG00000102243
ENSG00000157017	ENSG00000180479	ENSG00000143643
ENSG00000077549	ENSG00000137225	ENSG00000100084
ENSG00000108342	ENSG00000197603	ENSG00000080839
ENSG00000182534	ENSG00000120693	ENSG00000131373
ENSG00000171368	ENSG00000170866	ENSG00000165509
ENSG00000142856	ENSG00000157693	ENSG00000011638
ENSG00000213638	ENSG00000187624	ENSG00000171132

ENSG00000136878	ENSG00000164398	ENSG00000152256
ENSG00000184640	ENSG00000176787	ENSG00000171988
ENSG00000255112	ENSG00000214655	ENSG00000066117
ENSG00000039123	ENSG00000170683	ENSG00000091039
ENSG00000169683	ENSG00000066629	ENSG00000076650
ENSG00000164975	ENSG00000168152	ENSG00000108582
ENSG00000188959	ENSG00000188162	ENSG00000166446
ENSG00000159337	ENSG00000181449	ENSG00000135108
ENSG00000086619	ENSG00000136861	ENSG00000102226
ENSG00000064489	ENSG00000125895	ENSG00000204967
ENSG00000187791	ENSG00000153896	ENSG00000183023
ENSG00000115271	ENSG00000179988	ENSG00000069020
ENSG00000136811	ENSG00000204293	ENSG00000023318
ENSG00000197153	ENSG00000164038	ENSG00000147065
ENSG00000100764	ENSG00000070950	ENSG00000017483
ENSG00000130227	ENSG00000075415	ENSG00000196242
ENSG00000101843	ENSG00000112983	ENSG00000065029
ENSG00000151917	ENSG00000166377	ENSG00000103994
ENSG00000132639	ENSG00000158669	ENSG00000144567
ENSG00000154545	ENSG00000175048	ENSG00000181963
ENSG00000133138	ENSG00000167193	ENSG00000102158
ENSG00000182585	ENSG00000179603	ENSG00000172602
ENSG00000145721	ENSG00000197467	ENSG00000173698
ENSG00000069011	ENSG00000124490	ENSG00000133321
ENSG00000172538	ENSG00000112294	ENSG00000177303
ENSG00000113845	ENSG00000103381	ENSG00000105549
ENSG00000169087	ENSG00000163638	ENSG00000065615
ENSG00000213213	ENSG00000161618	ENSG00000092607
ENSG00000176845	ENSG00000213204	ENSG00000166479
ENSG00000182183	ENSG00000174776	ENSG00000143776
ENSG00000178233	ENSG00000132635	ENSG00000100105
ENSG00000166405	ENSG00000214021	ENSG00000184672
ENSG00000177370	ENSG00000171282	ENSG00000173402
ENSG00000070495	ENSG00000114439	ENSG00000109576
ENSG00000158710	ENSG00000203485	ENSG00000131067
ENSG00000103005	ENSG00000090932	ENSG00000176281
ENSG00000058673	ENSG00000160539	ENSG00000170145
ENSG00000152939	ENSG00000158104	ENSG00000126947
ENSG00000251380	ENSG00000213780	ENSG00000170743
ENSG00000256061	ENSG00000105248	ENSG00000153046
ENSG00000173436	ENSG00000163545	ENSG00000185811
ENSG00000046647	ENSG00000161204	ENSG00000213390
ENSG00000182851	ENSG00000167098	ENSG00000183671
ENSG00000120235	ENSG00000139880	ENSG00000138308
ENSG00000169906	ENSG00000112624	ENSG00000162722
ENSG00000196664	ENSG00000100263	ENSG00000138131
ENSG00000135148	ENSG00000204610	ENSG00000169258
ENSG00000001630	ENSG00000151883	ENSG00000188676
ENSG00000121067	ENSG00000122718	ENSG00000116679
ENSG00000214279	ENSG00000171903	ENSG00000004777
ENSG00000236446	ENSG00000111799	ENSG00000169327
ENSG00000181323	ENSG00000155666	ENSG00000184108
ENSG00000164109	ENSG00000136244	ENSG00000120210
ENSG00000062582	ENSG00000131626	ENSG00000183853
ENSG00000140564	ENSG00000132024	ENSG00000150867
ENSG00000003249	ENSG00000127831	ENSG00000113296
ENSG00000123636	ENSG00000185633	ENSG00000170786
ENSG00000171054	ENSG00000123352	ENSG00000253148
ENSG00000197279	ENSG00000186642	ENSG00000100813
ENSG00000080815	ENSG00000187240	ENSG00000242019
ENSG00000138758	ENSG00000198734	ENSG00000269699
ENSG00000198157	ENSG00000182179	ENSG00000141568
ENSG00000182308	ENSG00000183024	ENSG00000136021
ENSG00000196539	ENSG00000232434	ENSG00000175294

ENSG00000196419	ENSG00000128604	ENSG00000163599
ENSG00000125505	ENSG00000148204	ENSG00000135387
ENSG00000176274	ENSG00000064651	ENSG00000182022
ENSG00000111639	ENSG00000164068	ENSG00000142484
ENSG00000196511	ENSG00000137760	ENSG00000113209
ENSG00000174953	ENSG00000180354	ENSG00000166387
ENSG00000155926	ENSG00000197006	ENSG00000244411
ENSG00000189046	ENSG00000082996	ENSG00000089006
ENSG00000145337	ENSG00000115593	ENSG00000175787
ENSG00000183837	ENSG00000145545	ENSG00000154767
ENSG00000123600	ENSG00000165175	ENSG00000204420
ENSG00000187175	ENSG00000244731	ENSG00000169550
ENSG00000064999	ENSG00000112249	ENSG00000153446
ENSG00000096080	ENSG00000143819	ENSG00000171603
ENSG00000188050	ENSG00000116539	ENSG00000116748
ENSG00000196326	ENSG00000011347	ENSG00000241635
ENSG00000108100	ENSG00000138185	ENSG00000185775
ENSG00000213762	ENSG00000139624	ENSG00000129691
ENSG00000110203	ENSG00000096717	ENSG00000188906
ENSG00000158553	ENSG00000112782	ENSG00000163449
ENSG00000140832	ENSG00000157782	ENSG00000171496
ENSG00000155256	ENSG00000170909	ENSG00000130714
ENSG00000174137	ENSG00000134258	ENSG00000140829
ENSG00000166411	ENSG00000077097	ENSG00000081138
ENSG00000157060	ENSG00000180182	ENSG00000142784
ENSG00000196757	ENSG00000179388	ENSG00000204424
ENSG00000041880	ENSG00000102104	ENSG00000169946
ENSG00000118620	ENSG00000137801	ENSG00000135966
ENSG00000204767	ENSG00000109171	ENSG00000101181
ENSG00000134760	ENSG00000140986	ENSG00000183251
ENSG00000126768	ENSG00000182240	ENSG00000198205
ENSG00000176715	ENSG00000173264	ENSG00000115282
ENSG00000089289	ENSG00000166938	ENSG00000197912
ENSG00000170426	ENSG00000083444	ENSG00000177212
ENSG00000108439	ENSG00000159216	ENSG00000113838
ENSG00000088256	ENSG00000151650	ENSG00000170340
ENSG00000137273	ENSG00000168071	ENSG00000135624
ENSG00000184678	ENSG00000182326	ENSG00000175809
ENSG00000120784	ENSG00000169621	ENSG00000139915
ENSG00000164136	ENSG00000130675	ENSG00000175691
ENSG00000087250	ENSG00000181656	ENSG00000140943
ENSG00000164440	ENSG00000165359	ENSG00000136305
ENSG00000176555	ENSG00000196267	ENSG00000101265
ENSG00000055332	ENSG00000003987	ENSG00000165152
ENSG00000073350	ENSG00000204394	ENSG00000179055
ENSG00000004487	ENSG00000149532	ENSG00000175634
ENSG00000184378	ENSG00000196116	ENSG00000169919
ENSG00000148358	ENSG00000197816	ENSG00000118113
ENSG00000172375	ENSG00000177469	ENSG00000136630
ENSG00000204001	ENSG00000161010	ENSG00000165494
ENSG00000149634	ENSG00000007171	ENSG00000168454
ENSG00000177732	ENSG00000107816	ENSG00000165661
ENSG00000166869	ENSG00000112414	ENSG00000158571
ENSG00000168004	ENSG00000174705	ENSG00000101974
ENSG00000118242	ENSG00000174514	ENSG00000198740
ENSG00000175595	ENSG00000176438	ENSG00000105991
ENSG00000172461	ENSG00000100023	ENSG00000105392
ENSG00000021355	ENSG00000109625	ENSG00000121871
ENSG00000119537	ENSG00000125484	ENSG00000145194
ENSG00000108604	ENSG00000196659	ENSG00000204963
ENSG00000178636	ENSG00000180785	ENSG00000104714
ENSG00000169217	ENSG00000102893	ENSG00000131503
ENSG00000134419	ENSG00000142039	ENSG00000112280
ENSG00000148925	ENSG00000078589	ENSG00000185803

ENSG00000125870	ENSG00000131069	ENSG00000160752
ENSG00000136261	ENSG00000168040	ENSG00000154134
ENSG00000141101	ENSG00000180573	ENSG00000156398
ENSG00000204438	ENSG00000128944	ENSG00000139173
ENSG00000169488	ENSG00000136167	ENSG00000044090
ENSG00000203859	ENSG00000106266	ENSG00000174807
ENSG00000164403	ENSG00000051382	ENSG00000109684
ENSG00000146755	ENSG00000184083	ENSG00000080298
ENSG00000174238	ENSG00000165617	ENSG00000138311
ENSG00000185758	ENSG00000165805	ENSG00000249139
ENSG00000181631	ENSG00000106628	ENSG00000168787
ENSG00000119979	ENSG00000104472	ENSG00000124201
ENSG00000139163	ENSG00000181444	ENSG00000180116
ENSG00000138138	ENSG00000101224	ENSG00000213983
ENSG00000242265	ENSG00000253797	ENSG00000166716
ENSG00000139292	ENSG00000101367	ENSG00000127946
ENSG00000106852	ENSG00000213160	ENSG00000221840
ENSG00000253767	ENSG00000130413	ENSG00000249158
ENSG00000131482	ENSG00000115042	ENSG00000178338
ENSG00000108423	ENSG00000255292	ENSG00000182352
ENSG00000079950	ENSG00000166743	ENSG00000135365
ENSG00000105219	ENSG00000138378	ENSG00000196588
ENSG00000071994	ENSG00000203993	ENSG00000158008
ENSG00000168734	ENSG00000137648	ENSG00000137634
ENSG00000110917	ENSG00000133812	ENSG00000117054
ENSG00000115365	ENSG00000172465	ENSG00000225190
ENSG00000114767	ENSG00000144331	ENSG00000096070
ENSG00000164434	ENSG00000142937	ENSG00000256223
ENSG00000173862	ENSG00000187773	ENSG00000109991
ENSG00000214456	ENSG00000100968	ENSG00000100354
ENSG00000122025	ENSG00000198945	ENSG00000141434
ENSG00000146215	ENSG00000159374	ENSG00000182578
ENSG00000105281	ENSG00000137171	ENSG00000166831
ENSG00000110013	ENSG00000164082	ENSG00000126787
ENSG00000255307	ENSG00000205327	ENSG00000179046
ENSG00000240682	ENSG00000011422	ENSG00000165092
ENSG00000204099	ENSG00000093217	ENSG00000181761
ENSG00000204193	ENSG00000104967	ENSG00000090054
ENSG00000167701	ENSG00000145491	ENSG00000130560
ENSG00000164488	ENSG00000156959	ENSG00000188558
ENSG00000167925	ENSG00000112186	ENSG00000100220
ENSG00000164953	ENSG00000076662	ENSG00000104408
ENSG00000204673	ENSG00000140749	ENSG00000177030
ENSG00000205209	ENSG00000172638	ENSG00000164116
ENSG00000155438	ENSG00000118307	ENSG00000074590
ENSG00000184432	ENSG00000198964	ENSG00000105825
ENSG00000111669	ENSG00000164465	ENSG00000197887
ENSG00000134291	ENSG00000171446	ENSG00000187889
ENSG00000178700	ENSG00000163017	ENSG00000124212
ENSG00000103495	ENSG00000162522	ENSG00000104517
ENSG00000166228	ENSG00000125734	ENSG00000165794
ENSG00000158717	ENSG00000117385	ENSG00000104938
ENSG00000173812	ENSG00000167985	ENSG00000164574
ENSG00000171757	ENSG00000185722	ENSG00000183273
ENSG00000169609	ENSG00000116133	ENSG00000178078
ENSG00000119922	ENSG00000176540	ENSG00000095319
ENSG00000115239	ENSG00000171051	ENSG00000184675
ENSG00000164258	ENSG00000176473	ENSG00000173805
ENSG00000176871	ENSG00000221931	ENSG00000145526
ENSG00000187109	ENSG00000100321	ENSG00000197140
ENSG00000172878	ENSG00000109436	ENSG00000124749
ENSG00000125831	ENSG00000198003	ENSG00000173876
ENSG00000114529	ENSG00000185002	ENSG00000149136
ENSG00000254667	ENSG00000106366	ENSG00000119446

ENSG00000135441	ENSG00000165202	ENSG00000035664
ENSG00000116337	ENSG00000133884	ENSG00000125571
ENSG00000168614	ENSG00000131095	ENSG00000115380
ENSG00000106089	ENSG00000198518	ENSG00000158077
ENSG00000023909	ENSG00000171798	ENSG00000149131
ENSG00000088992	ENSG00000104413	ENSG00000169047
ENSG00000184221	ENSG00000142303	ENSG00000100014
ENSG00000144231	ENSG00000159842	ENSG00000173226
ENSG00000173581	ENSG00000111790	ENSG00000083807
ENSG00000198300	ENSG00000144061	ENSG00000133083
ENSG00000170854	ENSG00000197114	ENSG00000204941
ENSG00000145476	ENSG00000149090	ENSG00000138095
ENSG00000121897	ENSG00000103051	ENSG00000123104
ENSG00000114126	ENSG00000122861	ENSG00000085662
ENSG00000111364	ENSG00000178623	ENSG00000061273
ENSG00000153201	ENSG00000185610	ENSG00000015171
ENSG00000166478	ENSG00000181240	ENSG00000103274
ENSG00000166289	ENSG00000198399	ENSG00000011201
ENSG00000198521	ENSG00000196622	ENSG00000135747
ENSG00000253485	ENSG00000131203	ENSG00000197991
ENSG00000102858	ENSG00000102225	ENSG00000155366
ENSG00000198443	ENSG00000114573	ENSG00000177200
ENSG00000226650	ENSG00000142166	ENSG00000198917
ENSG00000203772	ENSG00000060971	ENSG00000177807
ENSG00000101311	ENSG00000141738	ENSG00000026036
ENSG00000081721	ENSG00000177885	ENSG00000105668
ENSG00000119396	ENSG00000177192	ENSG00000127838
ENSG00000162300	ENSG00000167615	ENSG00000165821
ENSG00000125347	ENSG00000132554	ENSG00000135423
ENSG00000175221	ENSG00000168672	ENSG00000187398
ENSG00000156463	ENSG00000143486	ENSG00000144820
ENSG00000143653	ENSG00000083290	ENSG00000136634
ENSG00000127415	ENSG00000171206	ENSG00000151005
ENSG00000241598	ENSG00000153498	ENSG00000164920
ENSG00000172775	ENSG00000205441	ENSG00000147202
ENSG00000144959	ENSG00000004478	ENSG00000174652
ENSG00000234516	ENSG00000137843	ENSG00000075856
ENSG00000123595	ENSG00000143452	ENSG00000108840
ENSG00000164366	ENSG00000197376	ENSG00000189108
ENSG00000010539	ENSG00000168938	ENSG00000109572
ENSG00000165521	ENSG00000145220	ENSG00000173080
ENSG00000184517	ENSG00000178950	ENSG00000100519
ENSG00000162620	ENSG00000101098	ENSG00000187210
ENSG00000138448	ENSG00000117598	ENSG00000181072
ENSG00000113048	ENSG00000089199	ENSG00000156802
ENSG00000108788	ENSG00000166016	ENSG00000241945
ENSG00000108932	ENSG00000179300	ENSG00000197555
ENSG00000185614	ENSG00000004534	ENSG00000144935
ENSG00000135298	ENSG00000133110	ENSG00000054793
ENSG00000189058	ENSG00000119686	ENSG00000129467
ENSG00000155918	ENSG00000205863	ENSG00000139344
ENSG00000167528	ENSG00000205726	ENSG00000181666
ENSG00000187682	ENSG00000184226	ENSG00000149150
ENSG00000203943	ENSG00000179241	ENSG00000148814
ENSG00000171202	ENSG00000137709	ENSG00000104356
ENSG00000171502	ENSG00000198062	ENSG00000008056
ENSG00000258653	ENSG00000152193	ENSG00000130803
ENSG00000171621	ENSG00000178498	ENSG00000144962
ENSG00000163377	ENSG00000076984	ENSG00000054967
ENSG00000246922	ENSG00000087269	ENSG00000141349
ENSG00000183542	ENSG00000130202	ENSG00000153575
ENSG00000147533	ENSG00000136485	ENSG00000179833
ENSG00000107929	ENSG00000000938	ENSG00000033122
ENSG00000269881	ENSG00000250709	ENSG00000080007

ENSG00000159166	ENSG00000170577	ENSG00000179918
ENSG00000198746	ENSG00000183260	ENSG00000005436
ENSG00000186452	ENSG00000250232	ENSG00000132510
ENSG00000167543	ENSG00000112137	ENSG00000147576
ENSG00000185591	ENSG00000170255	ENSG00000104365
ENSG00000181009	ENSG00000116745	ENSG00000125945
ENSG00000176387	ENSG00000100299	ENSG00000077279
ENSG00000215547	ENSG00000185298	ENSG00000151062
ENSG00000132613	ENSG00000111325	ENSG00000196497
ENSG00000180992	ENSG00000080845	ENSG00000136404
ENSG00000138769	ENSG00000168778	ENSG00000164175
ENSG00000184293	ENSG00000108828	ENSG00000163518
ENSG00000175779	ENSG00000111261	ENSG00000100629
ENSG00000113643	ENSG00000165511	ENSG00000182256
ENSG00000167183	ENSG00000040731	ENSG00000103426
ENSG00000179840	ENSG00000215262	ENSG00000185483
ENSG00000135924	ENSG00000108349	ENSG00000172543
ENSG00000055070	ENSG00000057294	ENSG00000018625
ENSG00000161583	ENSG00000117143	ENSG00000112706
ENSG00000151090	ENSG00000197971	ENSG00000157353
ENSG00000132274	ENSG00000110888	ENSG00000073910
ENSG00000110375	ENSG00000065150	ENSG00000182389
ENSG00000143164	ENSG00000157064	ENSG00000113119
ENSG00000074660	ENSG00000064195	ENSG00000147118
ENSG00000174748	ENSG00000174016	ENSG00000088038
ENSG00000135482	ENSG00000155252	ENSG00000164744
ENSG00000125447	ENSG00000153250	ENSG00000157654
ENSG00000269171	ENSG00000133256	ENSG00000158865
ENSG00000215274	ENSG00000142230	ENSG00000168916
ENSG00000153714	ENSG00000143740	ENSG00000184507
ENSG00000176454	ENSG00000140950	ENSG00000160710
ENSG00000104412	ENSG00000165553	ENSG00000064607
ENSG00000224132	ENSG00000008256	ENSG00000153317
ENSG00000197580	ENSG00000176619	ENSG00000183309
ENSG00000133115	ENSG00000130538	ENSG00000075539
ENSG00000175336	ENSG00000101222	ENSG00000011405
ENSG00000070269	ENSG00000132563	ENSG00000116406
ENSG00000120805	ENSG00000144681	ENSG00000189184
ENSG00000176009	ENSG00000153832	ENSG00000118690
ENSG00000178922	ENSG00000144730	ENSG00000178802
ENSG00000185238	ENSG00000170248	ENSG00000225614
ENSG00000137674	ENSG00000134827	ENSG00000168497
ENSG00000102312	ENSG00000196378	ENSG00000102034
ENSG00000188389	ENSG00000095539	ENSG00000145626
ENSG00000069943	ENSG00000117360	ENSG00000163581
ENSG00000070366	ENSG00000100344	ENSG00000113946
ENSG00000180909	ENSG00000170925	ENSG00000176834
ENSG00000110958	ENSG00000197147	ENSG00000137878
ENSG00000117594	ENSG00000138316	ENSG00000257008
ENSG00000185982	ENSG00000169871	ENSG00000143147
ENSG00000102387	ENSG00000135312	ENSG00000164151
ENSG00000268313	ENSG00000135913	ENSG00000109794
ENSG00000109917	ENSG00000269404	ENSG00000164050
ENSG00000162460	ENSG00000008128	ENSG00000062370
ENSG00000187778	ENSG00000151445	ENSG00000153930
ENSG00000197430	ENSG00000214787	ENSG00000128596
ENSG00000125356	ENSG00000125816	ENSG00000146038
ENSG00000140374	ENSG00000142623	ENSG00000163462
ENSG00000145826	ENSG00000168291	ENSG00000095564
ENSG00000156222	ENSG00000128849	ENSG00000168754
ENSG00000145041	ENSG00000188064	ENSG00000168661
ENSG00000183011	ENSG00000153294	ENSG00000179873
ENSG00000129197	ENSG00000049769	ENSG00000176246
ENSG00000130775	ENSG00000146233	ENSG00000006125

ENSG00000158156	ENSG00000139117	ENSG00000108312
ENSG00000163435	ENSG00000071537	ENSG00000185621
ENSG00000170788	ENSG00000197217	ENSG00000134243
ENSG00000006757	ENSG00000119401	ENSG00000092621
ENSG00000116473	ENSG00000001497	ENSG00000100191
ENSG00000083454	ENSG00000163322	ENSG00000119698
ENSG00000165733	ENSG00000158525	ENSG00000145348
ENSG00000151689	ENSG00000196344	ENSG00000137766
ENSG00000157870	ENSG00000145246	ENSG00000250423
ENSG00000150779	ENSG00000134538	ENSG00000008196
ENSG00000155506	ENSG00000147654	ENSG00000239998
ENSG00000107295	ENSG00000175619	ENSG00000167306
ENSG00000203972	ENSG00000186517	ENSG00000205359
ENSG00000130055	ENSG00000176893	ENSG00000162836
ENSG00000151882	ENSG00000089057	ENSG00000049540
ENSG00000163421	ENSG00000121853	ENSG00000226288
ENSG00000205667	ENSG00000182175	ENSG00000256660
ENSG00000129451	ENSG00000103227	ENSG00000105135
ENSG00000002330	ENSG00000165633	ENSG00000125255
ENSG00000197364	ENSG00000167664	ENSG00000088812
ENSG00000135537	ENSG00000160396	ENSG00000256349
ENSG00000266208	ENSG00000106236	ENSG00000104722
ENSG00000150787	ENSG00000074964	ENSG00000105122
ENSG00000099715	ENSG00000166450	ENSG00000117091
ENSG00000174891	ENSG00000174942	ENSG00000162148
ENSG00000131068	ENSG00000165632	ENSG00000113263
ENSG00000115128	ENSG00000182040	ENSG00000143379
ENSG00000181744	ENSG00000105708	ENSG00000196132
ENSG00000198625	ENSG00000036565	ENSG00000143970
ENSG00000179256	ENSG00000120868	ENSG00000101445
ENSG00000260970	ENSG00000147465	ENSG00000149564
ENSG00000166927	ENSG00000179934	ENSG00000185972
ENSG00000125810	ENSG00000114268	ENSG00000118473
ENSG00000110090	ENSG00000177455	ENSG00000105289
ENSG00000130382	ENSG00000165953	ENSG00000054938
ENSG00000173372	ENSG00000107186	ENSG00000185477
ENSG00000174371	ENSG00000152213	ENSG00000164627
ENSG00000176746	ENSG00000196104	ENSG00000013725
ENSG00000137996	ENSG00000120889	ENSG00000127124
ENSG00000180776	ENSG00000236104	ENSG00000075429
ENSG00000117748	ENSG00000103404	ENSG00000128594
ENSG00000171227	ENSG00000171307	ENSG00000163354
ENSG00000164620	ENSG00000107537	ENSG00000108846
ENSG00000152380	ENSG00000130584	ENSG00000133878
ENSG00000163464	ENSG00000144029	ENSG00000084693
ENSG00000081320	ENSG00000139629	ENSG00000095951
ENSG00000136273	ENSG00000176783	ENSG00000149476
ENSG00000134339	ENSG00000161558	ENSG00000108641
ENSG00000146530	ENSG00000135506	ENSG00000172159
ENSG00000184454	ENSG00000111732	ENSG00000106128
ENSG00000206171	ENSG00000090661	ENSG00000173166
ENSG00000111540	ENSG00000130035	ENSG00000119203
ENSG00000172915	ENSG00000248871	ENSG00000162736
ENSG00000139192	ENSG00000158467	ENSG00000180353
ENSG00000136710	ENSG00000064419	ENSG00000112149
ENSG00000171540	ENSG00000242689	ENSG00000196932
ENSG00000164270	ENSG00000050767	ENSG00000180318
ENSG00000140006	ENSG00000196248	ENSG00000153789
ENSG00000182334	ENSG00000146909	ENSG00000120322
ENSG00000140995	ENSG00000142102	ENSG00000132330
ENSG00000196072	ENSG00000156103	ENSG00000241978
ENSG00000113240	ENSG00000196277	ENSG00000166886
ENSG00000174827	ENSG00000114739	ENSG00000116871
ENSG00000255526	ENSG00000100490	ENSG00000065457

ENSG00000198034	ENSG00000007923	ENSG00000168237
ENSG00000122203	ENSG00000125903	ENSG00000187742
ENSG00000166575	ENSG00000186272	ENSG00000188340
ENSG00000196636	ENSG00000264545	ENSG00000078269
ENSG00000182551	ENSG00000132972	ENSG00000118402
ENSG00000118971	ENSG00000253953	ENSG00000165125
ENSG00000197769	ENSG00000112561	ENSG00000196739
ENSG00000205057	ENSG00000081803	ENSG00000149930
ENSG00000148690	ENSG00000157542	ENSG00000184867
ENSG00000075785	ENSG00000075240	ENSG00000100889
ENSG00000168301	ENSG00000002933	ENSG00000179715
ENSG00000162571	ENSG00000110651	ENSG00000176928
ENSG00000257093	ENSG00000155463	ENSG00000163982
ENSG00000081087	ENSG00000196368	ENSG00000157703
ENSG00000179639	ENSG00000117528	ENSG00000122733
ENSG00000136918	ENSG00000184697	ENSG00000101425
ENSG00000169246	ENSG00000105821	ENSG00000157557
ENSG00000145908	ENSG00000122574	ENSG00000188559
ENSG00000141560	ENSG00000109674	ENSG00000005844
ENSG00000145901	ENSG00000172840	ENSG00000119042
ENSG00000149196	ENSG00000105221	ENSG00000087303
ENSG00000170955	ENSG00000165471	ENSG00000125703
ENSG00000187676	ENSG00000166073	ENSG00000175198
ENSG00000256391	ENSG00000106631	ENSG00000165695
ENSG00000139508	ENSG00000115828	ENSG00000151834
ENSG00000145494	ENSG00000139797	ENSG00000186638
ENSG00000120437	ENSG00000105251	ENSG00000197705
ENSG00000137221	ENSG00000102984	ENSG00000166986
ENSG00000135679	ENSG00000162607	ENSG00000152592
ENSG00000154122	ENSG00000146147	ENSG00000179520
ENSG00000122376	ENSG00000239857	ENSG00000130703
ENSG00000213186	ENSG00000169760	ENSG00000181752
ENSG00000100151	ENSG00000100239	ENSG00000174564
ENSG00000198090	ENSG00000204310	ENSG00000039068
ENSG00000039319	ENSG00000198553	ENSG00000185261
ENSG00000244693	ENSG00000136688	ENSG00000074771
ENSG00000167862	ENSG00000106344	ENSG00000197748
ENSG00000135111	ENSG00000136854	ENSG00000144821
ENSG00000112175	ENSG00000124249	ENSG00000134775
ENSG00000114638	ENSG00000139514	ENSG00000149115
ENSG00000142684	ENSG00000123575	ENSG00000132182
ENSG00000090520	ENSG00000153093	ENSG00000170775
ENSG00000165434	ENSG00000133794	ENSG00000095303
ENSG00000167005	ENSG00000087076	ENSG00000174038
ENSG00000070423	ENSG00000136802	ENSG00000165091
ENSG00000142657	ENSG00000136940	ENSG00000173212
ENSG00000055732	ENSG00000100368	ENSG00000005961
ENSG00000204481	ENSG00000169860	ENSG00000170454
ENSG00000103248	ENSG00000066044	ENSG00000203780
ENSG00000137509	ENSG00000159128	ENSG00000156194
ENSG00000123219	ENSG00000153823	ENSG00000185085
ENSG00000159352	ENSG00000114251	ENSG00000145242
ENSG00000128310	ENSG00000141639	ENSG00000161217
ENSG00000179363	ENSG00000160190	ENSG00000181090
ENSG00000125971	ENSG00000089177	ENSG00000167383
ENSG00000152475	ENSG00000154997	ENSG00000172548
ENSG00000155714	ENSG00000067177	ENSG00000137261
ENSG00000132965	ENSG00000163050	ENSG00000148187
ENSG00000125827	ENSG00000100078	ENSG00000122674
ENSG00000100298	ENSG00000001036	ENSG00000105499
ENSG00000074842	ENSG00000127325	ENSG00000198798
ENSG00000129282	ENSG00000003096	ENSG00000197587
ENSG00000213921	ENSG00000106624	ENSG00000174898
ENSG00000185056	ENSG00000164211	ENSG00000150510

ENSG00000181016	ENSG00000124120	ENSG00000107829
ENSG00000117228	ENSG00000173276	ENSG00000082641
ENSG00000162438	ENSG00000167642	ENSG00000134007
ENSG00000159176	ENSG00000162989	ENSG00000163810
ENSG00000136319	ENSG00000213023	ENSG00000148600
ENSG00000227507	ENSG00000197713	ENSG00000198089
ENSG00000060140	ENSG00000106410	ENSG00000131864
ENSG00000100804	ENSG00000103512	ENSG00000138375
ENSG00000108255	ENSG00000073464	ENSG00000198743
ENSG00000146409	ENSG00000164161	ENSG00000196136
ENSG00000108829	ENSG00000067064	ENSG00000134954
ENSG00000147471	ENSG00000205683	ENSG00000160208
ENSG00000262481	ENSG00000167612	ENSG00000180871
ENSG00000166170	ENSG00000205629	ENSG00000049192
ENSG00000104635	ENSG00000117305	ENSG00000114166
ENSG00000011677	ENSG00000122490	ENSG00000139597
ENSG00000179387	ENSG00000116691	ENSG00000136267
ENSG00000182050	ENSG00000114861	ENSG00000197102
ENSG00000167434	ENSG00000114316	ENSG00000150961
ENSG00000080824	ENSG00000064666	ENSG00000169188
ENSG00000137168	ENSG00000129173	ENSG00000166866
ENSG00000172551	ENSG00000128815	ENSG00000134249
ENSG00000140391	ENSG00000179168	ENSG00000122678
ENSG00000019582	ENSG00000172817	ENSG00000159708
ENSG00000128791	ENSG00000178772	ENSG00000043039
ENSG00000237289	ENSG00000160410	ENSG00000137252
ENSG00000212659	ENSG00000164532	ENSG00000151812
ENSG00000126952	ENSG00000012504	ENSG00000105880
ENSG00000172489	ENSG00000143507	ENSG00000131467
ENSG00000212916	ENSG00000120669	ENSG00000158966
ENSG00000253958	ENSG00000156009	ENSG00000149133
ENSG00000170279	ENSG00000077080	ENSG00000105953
ENSG00000143891	ENSG00000064490	ENSG00000103365
ENSG00000177025	ENSG00000171872	ENSG00000167613
ENSG00000197595	ENSG00000164077	ENSG00000129103
ENSG00000166523	ENSG00000101842	ENSG00000182077
ENSG00000267022	ENSG00000088888	ENSG00000100393
ENSG00000135905	ENSG00000128253	ENSG00000170745
ENSG00000100416	ENSG00000087086	ENSG00000144283
ENSG00000143314	ENSG00000134809	ENSG00000139144
ENSG00000120057	ENSG00000163704	ENSG00000144036
ENSG00000183654	ENSG00000154556	ENSG00000160818
ENSG00000157110	ENSG00000196712	ENSG00000130119
ENSG00000163737	ENSG00000186919	ENSG00000145681
ENSG00000164056	ENSG00000165338	ENSG00000204574
ENSG00000154485	ENSG00000109424	ENSG00000154928
ENSG00000186471	ENSG00000144711	ENSG00000197948
ENSG00000157992	ENSG00000180096	ENSG00000159063
ENSG00000094963	ENSG00000165282	ENSG00000196998
ENSG00000173905	ENSG00000119725	ENSG00000187323
ENSG00000180998	ENSG00000147697	ENSG00000198752
ENSG00000173239	ENSG00000141448	ENSG00000187908
ENSG00000186806	ENSG00000146414	ENSG00000135643
ENSG00000138669	ENSG00000140534	ENSG00000133030
ENSG00000118939	ENSG00000129538	ENSG00000157827
ENSG00000169169	ENSG00000060491	ENSG00000138646
ENSG00000132837	ENSG00000138592	ENSG00000101004
ENSG00000184166	ENSG00000139998	ENSG00000077235
ENSG00000138190	ENSG00000115808	ENSG00000135324
ENSG00000123810	ENSG00000106605	ENSG00000090686
ENSG00000166428	ENSG00000185825	ENSG00000116584
ENSG00000124207	ENSG00000170442	ENSG00000065989
ENSG00000099866	ENSG00000187848	ENSG00000003400
ENSG00000070190	ENSG00000010404	ENSG00000119681

ENSG00000241837	ENSG00000094914	ENSG00000113302
ENSG00000100503	ENSG00000204361	ENSG00000125247
ENSG00000105784	ENSG00000166862	ENSG00000139178
ENSG00000156381	ENSG00000115207	ENSG00000074800
ENSG00000123684	ENSG00000065883	ENSG00000138835
ENSG00000068489	ENSG00000115694	ENSG00000101746
ENSG00000128524	ENSG00000007202	ENSG00000136048
ENSG00000111674	ENSG00000088756	ENSG00000134532
ENSG00000144635	ENSG00000135929	ENSG00000204396
ENSG00000111670	ENSG00000170396	ENSG00000183576
ENSG00000133808	ENSG00000204711	ENSG00000102468
ENSG00000185880	ENSG00000169379	ENSG00000171109
ENSG00000188295	ENSG00000105401	ENSG00000117501
ENSG00000241128	ENSG00000204278	ENSG00000009413
ENSG00000152795	ENSG00000112936	ENSG00000112685
ENSG00000100528	ENSG00000182782	ENSG00000059378
ENSG00000131116	ENSG00000198570	ENSG00000141219
ENSG00000119655	ENSG00000120162	ENSG00000132704
ENSG00000120071	ENSG00000198932	ENSG00000110876
ENSG00000131142	ENSG00000137073	ENSG00000103375
ENSG00000170162	ENSG00000130309	ENSG00000180357
ENSG00000255152	ENSG00000241878	ENSG00000181958
ENSG00000170271	ENSG00000134259	ENSG00000254737
ENSG00000183250	ENSG00000158062	ENSG00000171346
ENSG00000214216	ENSG00000188886	ENSG00000196724
ENSG00000175582	ENSG00000196475	ENSG00000185052
ENSG00000152034	ENSG00000132463	ENSG00000018189
ENSG00000070526	ENSG00000178177	ENSG00000105366
ENSG00000189339	ENSG00000177628	ENSG00000153157
ENSG00000135549	ENSG00000072210	ENSG00000111664
ENSG00000176108	ENSG00000174059	ENSG00000140057
ENSG00000047634	ENSG00000113734	ENSG00000131831
ENSG00000106080	ENSG00000151360	ENSG00000196263
ENSG00000255302	ENSG00000108823	ENSG00000160783
ENSG00000092108	ENSG00000167981	ENSG00000010292
ENSG00000088205	ENSG00000075234	ENSG00000025293
ENSG00000133169	ENSG00000067066	ENSG00000102699
ENSG00000104818	ENSG00000174177	ENSG00000198844
ENSG00000183034	ENSG00000241794	ENSG00000096968
ENSG00000132329	ENSG00000111321	ENSG00000186204
ENSG00000136051	ENSG00000146013	ENSG00000100767
ENSG00000173726	ENSG00000102349	ENSG00000072121
ENSG00000104983	ENSG00000138696	ENSG00000058404
ENSG00000041802	ENSG00000100583	ENSG00000074410
ENSG00000101333	ENSG00000198028	ENSG00000182963
ENSG00000166347	ENSG00000166451	ENSG00000049618
ENSG00000228459	ENSG00000172009	ENSG00000006611
ENSG00000240542	ENSG00000197417	ENSG00000196411
ENSG00000080910	ENSG00000159763	ENSG00000167258
ENSG00000258984	ENSG00000084734	ENSG00000174938
ENSG00000164821	ENSG00000128394	ENSG00000044574
ENSG00000214026	ENSG00000114423	ENSG00000119899
ENSG00000140350	ENSG00000095587	ENSG00000174837
ENSG00000137992	ENSG00000137812	ENSG00000197566
ENSG00000139352	ENSG00000101187	ENSG00000112494
ENSG00000135226	ENSG00000258529	ENSG00000123815
ENSG00000139269	ENSG00000123901	ENSG00000182489
ENSG00000180332	ENSG00000187695	ENSG00000172771
ENSG00000138495	ENSG00000136045	ENSG00000171481
ENSG00000198856	ENSG00000196154	ENSG00000116353
ENSG00000056586	ENSG00000167460	ENSG00000158290
ENSG00000187189	ENSG00000161021	ENSG00000167995
ENSG00000166257	ENSG00000143869	ENSG00000109685
ENSG00000169592	ENSG00000167657	ENSG00000215041

ENSG00000053770	ENSG00000198176	ENSG00000165370
ENSG00000107560	ENSG00000171604	ENSG00000066827
ENSG00000180386	ENSG00000156639	ENSG00000183230
ENSG00000107745	ENSG00000135063	ENSG00000160712
ENSG00000162298	ENSG00000167487	ENSG00000169231
ENSG00000100591	ENSG00000136848	ENSG00000168334
ENSG00000153774	ENSG00000112033	ENSG00000185019
ENSG00000163596	ENSG00000085465	ENSG00000132842
ENSG00000160087	ENSG00000165478	ENSG00000101605
ENSG00000112343	ENSG00000165868	ENSG00000196834
ENSG00000140274	ENSG00000078098	ENSG00000184735
ENSG00000188910	ENSG00000150347	ENSG00000197579
ENSG00000175646	ENSG00000204510	ENSG00000151490
ENSG00000140264	ENSG00000136939	ENSG00000112562
ENSG00000170113	ENSG00000131653	ENSG00000111218
ENSG00000147614	ENSG00000187607	ENSG00000005884
ENSG00000070501	ENSG00000123838	ENSG00000176566
ENSG00000124196	ENSG00000054611	ENSG00000145868
ENSG00000175938	ENSG00000164086	ENSG00000137501
ENSG00000196642	ENSG00000255104	ENSG00000130856
ENSG00000107902	ENSG00000135519	ENSG00000128683
ENSG00000170703	ENSG00000003756	ENSG00000128573
ENSG00000250733	ENSG00000179088	ENSG00000196600
ENSG00000105617	ENSG00000131269	ENSG00000011566
ENSG00000204568	ENSG00000185513	ENSG00000125319
ENSG00000148158	ENSG00000066923	ENSG00000101255
ENSG00000214022	ENSG00000223547	ENSG00000170289
ENSG00000100288	ENSG00000081051	ENSG00000204120
ENSG00000167395	ENSG00000187118	ENSG00000110148
ENSG00000174804	ENSG00000015520	ENSG00000149633
ENSG00000137691	ENSG00000102239	ENSG00000196098
ENSG00000150337	ENSG00000197721	ENSG00000197496
ENSG00000136930	ENSG00000173926	ENSG00000160883
ENSG00000198454	ENSG00000117115	ENSG00000146918
ENSG00000126970	ENSG00000183747	ENSG00000143190
ENSG00000256566	ENSG00000186298	ENSG00000215305
ENSG00000204618	ENSG00000125498	ENSG00000138193
ENSG00000163482	ENSG00000068120	ENSG00000182871
ENSG00000125378	ENSG00000244255	ENSG00000166507
ENSG00000167799	ENSG00000156011	ENSG00000167110
ENSG00000166164	ENSG00000180596	ENSG00000040633
ENSG00000005801	ENSG00000176406	ENSG00000162896
ENSG00000106346	ENSG00000121579	ENSG00000254536
ENSG00000257949	ENSG00000036530	ENSG00000169330
ENSG00000181029	ENSG00000067606	ENSG00000182348
ENSG00000162227	ENSG00000149043	ENSG00000121879
ENSG00000105516	ENSG00000008311	ENSG00000204560
ENSG00000186119	ENSG00000249481	ENSG00000105486
ENSG00000203740	ENSG00000167851	ENSG00000187566
ENSG00000112541	ENSG00000176076	ENSG00000070778
ENSG00000141485	ENSG00000224586	ENSG00000140522
ENSG00000184731	ENSG00000182054	ENSG00000115935
ENSG00000204869	ENSG00000100626	ENSG00000143674
ENSG00000172410	ENSG00000118965	ENSG00000112539
ENSG00000143995	ENSG00000205642	ENSG00000115896
ENSG00000132603	ENSG00000102103	ENSG00000136040
ENSG00000064652	ENSG00000160862	ENSG00000184194
ENSG00000155621	ENSG00000135976	ENSG00000135426
ENSG00000064886	ENSG00000075188	ENSG00000167825
ENSG00000166848	ENSG00000185033	ENSG00000064393
ENSG00000162244	ENSG00000134627	ENSG00000084070
ENSG00000168209	ENSG00000130402	ENSG00000153234
ENSG00000198960	ENSG00000147133	ENSG00000166359
ENSG00000115661	ENSG00000033050	ENSG00000188868

ENSG00000139180	ENSG00000168070	ENSG00000169439
ENSG00000168827	ENSG00000101911	ENSG00000072364
ENSG00000172023	ENSG00000105929	ENSG00000152223
ENSG00000165899	ENSG00000105723	ENSG00000120800
ENSG00000174775	ENSG00000113916	ENSG00000095777
ENSG00000139793	ENSG00000166313	ENSG00000104972
ENSG00000127954	ENSG00000129214	ENSG00000152767
ENSG00000186952	ENSG00000105559	ENSG00000165097
ENSG00000185164	ENSG00000139914	ENSG00000117834
ENSG00000131470	ENSG00000023892	ENSG00000143858
ENSG00000138385	ENSG00000123612	ENSG00000198952
ENSG00000112378	ENSG00000159882	ENSG00000064218
ENSG00000136448	ENSG00000185115	ENSG00000106772
ENSG00000197045	ENSG00000143067	ENSG00000112379
ENSG00000168260	ENSG00000170790	ENSG00000128833
ENSG00000077348	ENSG00000025708	ENSG00000134313
ENSG00000184860	ENSG00000142065	ENSG00000111962
ENSG00000198794	ENSG00000120318	ENSG00000144218
ENSG00000183324	ENSG00000171133	ENSG00000204859
ENSG00000107672	ENSG00000135917	ENSG00000108018
ENSG00000198492	ENSG00000105397	ENSG00000108852
ENSG00000143110	ENSG00000147381	ENSG00000167333
ENSG00000007952	ENSG00000154162	ENSG00000184588
ENSG00000119523	ENSG00000186967	ENSG00000118007
ENSG00000108947	ENSG00000033627	ENSG00000130816
ENSG00000126814	ENSG00000143303	ENSG00000134253
ENSG00000132507	ENSG00000004399	ENSG00000197818
ENSG00000102021	ENSG00000254772	ENSG00000179583
ENSG00000197790	ENSG00000125089	ENSG00000032219
ENSG00000076864	ENSG00000130812	ENSG00000205420
ENSG00000069966	ENSG00000179954	ENSG00000128802
ENSG00000168242	ENSG00000168621	ENSG00000117000
ENSG00000121058	ENSG00000196365	ENSG00000164715
ENSG00000157778	ENSG00000171570	ENSG00000101916
ENSG00000159648	ENSG00000197110	ENSG00000090402
ENSG00000152284	ENSG00000162368	ENSG00000009335
ENSG00000183733	ENSG00000010379	ENSG00000124574
ENSG00000163029	ENSG00000072849	ENSG00000116580
ENSG00000083937	ENSG00000148180	ENSG00000172534
ENSG00000018610	ENSG00000118369	ENSG00000146282
ENSG00000183346	ENSG00000143816	ENSG00000134262
ENSG00000128989	ENSG00000184254	ENSG00000101448
ENSG00000184465	ENSG00000176571	ENSG00000163071
ENSG00000204519	ENSG00000136237	ENSG00000157741
ENSG00000119938	ENSG00000142583	ENSG00000101447
ENSG00000175536	ENSG00000180787	ENSG00000183434
ENSG00000164708	ENSG00000171823	ENSG00000123191
ENSG00000128564	ENSG00000171049	ENSG00000127527
ENSG00000235608	ENSG00000080503	ENSG00000187555
ENSG00000134905	ENSG00000139223	ENSG00000187764
ENSG00000134152	ENSG00000197651	ENSG00000198934
ENSG00000136944	ENSG00000185219	ENSG00000176945
ENSG00000170262	ENSG00000136859	ENSG00000135439
ENSG00000188060	ENSG00000137204	ENSG00000241839
ENSG00000125995	ENSG00000075643	ENSG00000133460
ENSG00000154478	ENSG00000112303	ENSG00000130244
ENSG00000106038	ENSG00000094880	ENSG00000160310
ENSG00000139405	ENSG00000171855	ENSG00000101596
ENSG00000248405	ENSG00000113389	ENSG00000016602
ENSG00000135336	ENSG00000105697	ENSG00000101958
ENSG00000156509	ENSG00000139625	ENSG00000175175
ENSG00000128891	ENSG00000228144	ENSG00000072501
ENSG00000186732	ENSG00000164334	ENSG00000105976
ENSG00000166796	ENSG00000215568	ENSG00000131389

ENSG00000174718	ENSG00000157119	ENSG00000107099
ENSG00000090266	ENSG00000089159	ENSG00000172943
ENSG00000115649	ENSG00000133048	ENSG00000185046
ENSG00000077984	ENSG00000125952	ENSG00000120008
ENSG00000198780	ENSG00000113272	ENSG00000123338
ENSG00000114857	ENSG00000186970	ENSG00000132692
ENSG00000152684	ENSG00000121903	ENSG00000159403
ENSG00000163081	ENSG00000112309	ENSG00000134452
ENSG00000203877	ENSG00000169562	ENSG00000141298
ENSG00000235711	ENSG00000072864	ENSG00000013503
ENSG00000247077	ENSG00000197846	ENSG00000185149
ENSG00000123545	ENSG00000140332	ENSG00000198901
ENSG00000167941	ENSG00000065328	ENSG00000016082
ENSG00000170848	ENSG00000138798	ENSG00000187527
ENSG00000124713	ENSG00000048140	ENSG00000179455
ENSG00000168348	ENSG00000140955	ENSG00000115295
ENSG00000184515	ENSG00000048162	ENSG00000111335
ENSG00000172671	ENSG00000168995	ENSG00000129292
ENSG00000133250	ENSG00000145860	ENSG00000145819
ENSG00000182628	ENSG00000164430	ENSG00000177119
ENSG00000167014	ENSG00000011275	ENSG00000145244
ENSG00000183255	ENSG00000142207	ENSG00000106804
ENSG00000124172	ENSG00000161574	ENSG00000117713
ENSG00000124134	ENSG00000090020	ENSG00000137872
ENSG00000197238	ENSG00000204149	ENSG00000123066
ENSG00000160570	ENSG00000112234	ENSG00000183303
ENSG00000140386	ENSG00000178852	ENSG00000116128
ENSG00000118702	ENSG00000187180	ENSG00000197081
ENSG00000176937	ENSG00000132677	ENSG00000196653
ENSG00000134590	ENSG00000089123	ENSG00000143119
ENSG00000111726	ENSG00000177791	ENSG00000062096
ENSG00000178878	ENSG00000188112	ENSG00000085719
ENSG00000111229	ENSG00000140199	ENSG00000163902
ENSG00000198783	ENSG00000187800	ENSG00000175471
ENSG00000186591	ENSG00000152377	ENSG00000010165
ENSG00000213366	ENSG00000091622	ENSG00000173809
ENSG00000143498	ENSG00000177675	ENSG00000136068
ENSG00000153130	ENSG00000101162	ENSG00000186513
ENSG00000183770	ENSG00000168830	ENSG00000155158
ENSG00000063245	ENSG00000107036	ENSG00000188827
ENSG00000204352	ENSG00000189195	ENSG00000171316
ENSG00000017427	ENSG00000184838	ENSG00000138756
ENSG00000074695	ENSG00000005889	ENSG00000165795
ENSG00000205835	ENSG00000100399	ENSG00000172260
ENSG00000055211	ENSG00000036473	ENSG00000104067
ENSG00000267041	ENSG00000105137	ENSG00000173120
ENSG00000213799	ENSG00000089053	ENSG00000134201
ENSG00000184897	ENSG00000132781	ENSG00000142511
ENSG00000257315	ENSG00000168930	ENSG00000163217
ENSG00000134824	ENSG00000170486	ENSG00000083168
ENSG00000166192	ENSG00000164466	ENSG00000204634
ENSG00000157211	ENSG00000174460	ENSG00000135164
ENSG00000114544	ENSG00000147394	ENSG00000171772
ENSG00000228804	ENSG00000135823	ENSG00000126870
ENSG00000119138	ENSG00000196415	ENSG00000094661
ENSG00000167332	ENSG00000147509	ENSG00000162714
ENSG00000138080	ENSG00000242732	ENSG00000135338
ENSG00000106333	ENSG00000133313	ENSG00000162687
ENSG00000151364	ENSG00000166266	ENSG00000198173
ENSG00000256407	ENSG00000100031	ENSG00000140459
ENSG00000221837	ENSG00000104885	ENSG00000028310
ENSG00000177694	ENSG00000130595	ENSG00000110497
ENSG00000174928	ENSG00000144136	ENSG00000136383
ENSG00000101294	ENSG00000134318	ENSG00000170579

ENSG00000163832	ENSG00000131791	ENSG00000183337
ENSG00000154553	ENSG00000105371	ENSG00000147408
ENSG00000136492	ENSG00000185666	ENSG00000198369
ENSG00000153094	ENSG00000140093	ENSG00000179399
ENSG00000197745	ENSG00000008382	ENSG00000166006
ENSG00000137502	ENSG00000160349	ENSG00000143324
ENSG00000143297	ENSG00000177156	ENSG00000156886
ENSG00000176890	ENSG00000112118	ENSG00000053747
ENSG00000044459	ENSG00000070759	ENSG00000091136
ENSG00000237541	ENSG00000166825	ENSG00000041515
ENSG00000177105	ENSG00000183323	ENSG00000160321
ENSG00000134901	ENSG00000121570	ENSG00000170953
ENSG00000213988	ENSG00000204217	ENSG00000153956
ENSG00000234776	ENSG00000113360	ENSG00000169515
ENSG00000226894	ENSG00000138074	ENSG00000107815
ENSG00000197046	ENSG00000123700	ENSG00000135899
ENSG00000184182	ENSG00000203663	ENSG00000107263
ENSG00000136643	ENSG00000116996	ENSG00000006453
ENSG00000153936	ENSG00000111981	ENSG00000204301
ENSG00000162999	ENSG00000198815	ENSG00000095713
ENSG00000125869	ENSG00000164093	ENSG00000163395
ENSG00000127366	ENSG00000160219	ENSG00000154783
ENSG00000185049	ENSG00000137103	ENSG00000077264
ENSG00000143001	ENSG00000187492	ENSG00000156787
ENSG00000103490	ENSG00000162769	ENSG00000197479
ENSG00000118640	ENSG00000205869	ENSG00000113492
ENSG00000188474	ENSG00000170423	ENSG00000174914
ENSG00000126264	ENSG00000152359	ENSG00000174667
ENSG00000111837	ENSG00000143437	ENSG00000138771
ENSG00000171097	ENSG00000135409	ENSG00000182674
ENSG00000186462	ENSG00000141551	ENSG00000181333
ENSG00000008282	ENSG00000163510	ENSG00000111879
ENSG00000100325	ENSG00000158481	ENSG00000184470
ENSG00000162188	ENSG00000174996	ENSG00000134398
ENSG00000160131	ENSG00000069702	ENSG00000171714
ENSG00000185813	ENSG00000171017	ENSG00000162946
ENSG00000176396	ENSG00000152104	ENSG00000162598
ENSG00000122223	ENSG00000087266	ENSG00000126001
ENSG00000102010	ENSG00000183779	ENSG00000055483
ENSG00000099256	ENSG00000099203	ENSG00000182199
ENSG00000151553	ENSG00000120696	ENSG00000101442
ENSG00000197442	ENSG00000128829	ENSG00000138435
ENSG00000170006	ENSG00000106069	ENSG00000167634
ENSG00000198736	ENSG00000117298	ENSG00000089558
ENSG00000188324	ENSG00000138892	ENSG00000124253
ENSG00000126458	ENSG00000183389	ENSG00000170484
ENSG00000136111	ENSG00000114670	ENSG00000143850
ENSG00000147402	ENSG00000183313	ENSG00000152495
ENSG00000188735	ENSG00000108878	ENSG00000151503
ENSG00000118096	ENSG00000139973	ENSG00000147082
ENSG00000182287	ENSG00000206561	ENSG00000174231
ENSG00000226593	ENSG00000173653	ENSG00000029364
ENSG00000165934	ENSG00000131183	ENSG00000183690
ENSG00000102970	ENSG00000197157	ENSG00000147246
ENSG00000128045	ENSG00000167207	ENSG00000072818
ENSG00000198586	ENSG00000050748	ENSG00000160584
ENSG00000160318	ENSG00000196187	ENSG00000117597
ENSG00000232856	ENSG00000116726	ENSG00000167210
ENSG00000153048	ENSG00000135773	ENSG00000022556
ENSG00000131059	ENSG00000150637	ENSG00000168434
ENSG00000204449	ENSG00000122254	ENSG00000130844
ENSG00000170290	ENSG00000188786	ENSG00000176095
ENSG00000172361	ENSG00000221818	ENSG00000135074
ENSG00000143774	ENSG00000171786	ENSG00000131697

ENSG00000179820	ENSG00000174428	ENSG00000136750
ENSG00000189292	ENSG00000133106	ENSG00000023330
ENSG00000137767	ENSG00000049883	ENSG00000157625
ENSG00000255835	ENSG00000166477	ENSG00000160868
ENSG00000256713	ENSG00000145198	ENSG00000105996
ENSG00000124275	ENSG00000166685	ENSG00000169340
ENSG00000165055	ENSG00000149646	ENSG00000181322
ENSG00000197982	ENSG00000080200	ENSG00000142512
ENSG00000181513	ENSG00000141404	ENSG00000152953
ENSG00000067225	ENSG00000164967	ENSG00000163399
ENSG00000138028	ENSG00000180043	ENSG00000187862
ENSG00000164023	ENSG00000084092	ENSG00000181722
ENSG00000189030	ENSG00000186716	ENSG00000135127
ENSG00000129744	ENSG00000108883	ENSG00000239961
ENSG00000103269	ENSG00000086758	ENSG00000127529
ENSG00000037965	ENSG00000175329	ENSG00000188089
ENSG00000147872	ENSG00000188687	ENSG00000134757
ENSG00000112357	ENSG00000162819	ENSG00000173572
ENSG00000105131	ENSG00000143554	ENSG00000134516
ENSG00000099957	ENSG00000022976	ENSG00000150403
ENSG00000138152	ENSG00000206503	ENSG00000078725
ENSG00000105717	ENSG00000187166	ENSG00000008086
ENSG00000267796	ENSG00000110195	ENSG00000074803
ENSG00000091583	ENSG00000184908	ENSG00000073146
ENSG00000188263	ENSG00000167114	ENSG00000196152
ENSG00000185821	ENSG00000163606	ENSG00000148942
ENSG00000150783	ENSG00000066735	ENSG00000145850
ENSG00000172987	ENSG00000180475	ENSG00000157657
ENSG00000155026	ENSG00000106536	ENSG00000170921
ENSG00000171944	ENSG00000137752	ENSG00000143365
ENSG00000157869	ENSG00000167470	ENSG00000066032
ENSG00000123395	ENSG00000163788	ENSG00000165671
ENSG00000101473	ENSG00000205045	ENSG00000198912
ENSG00000206538	ENSG00000186867	ENSG00000145781
ENSG00000047849	ENSG00000087842	ENSG00000198033
ENSG00000173662	ENSG00000254656	ENSG00000115053
ENSG00000132004	ENSG00000185985	ENSG00000153395
ENSG00000166333	ENSG00000157653	ENSG00000173705
ENSG00000185933	ENSG00000184886	ENSG00000143179
ENSG00000143368	ENSG00000115677	ENSG00000035681
ENSG00000012211	ENSG00000160161	ENSG00000125386
ENSG00000167524	ENSG00000100938	ENSG00000154227
ENSG00000148832	ENSG00000186854	ENSG00000146433
ENSG00000231068	ENSG00000112511	ENSG00000182634
ENSG00000167645	ENSG00000116885	ENSG00000081913
ENSG00000171503	ENSG00000198821	ENSG00000135596
ENSG00000107018	ENSG00000132670	ENSG00000114757
ENSG00000185873	ENSG00000144504	ENSG00000118432
ENSG00000181215	ENSG00000143318	ENSG00000127666
ENSG00000152422	ENSG00000144339	ENSG00000144724
ENSG00000172301	ENSG00000165678	ENSG00000160185
ENSG00000146054	ENSG00000127955	ENSG00000151789
ENSG00000136697	ENSG00000134283	ENSG00000052126
ENSG00000213999	ENSG00000149507	ENSG00000185013
ENSG00000213892	ENSG00000128626	ENSG00000138792
ENSG00000172354	ENSG00000262355	ENSG00000008277
ENSG00000185905	ENSG00000186487	ENSG00000106351
ENSG00000186660	ENSG00000068796	ENSG00000145623
ENSG00000141141	ENSG00000147799	ENSG00000249853
ENSG00000100731	ENSG00000028277	ENSG00000103544
ENSG00000184990	ENSG00000185915	ENSG00000143669
ENSG00000148572	ENSG00000110975	ENSG00000146453
ENSG00000116731	ENSG00000143105	ENSG00000168826
ENSG00000023191	ENSG00000136573	ENSG00000138674

ENSG00000105327	ENSG00000172005	ENSG00000149177
ENSG00000243501	ENSG00000129450	ENSG00000163564
ENSG00000188596	ENSG00000089597	ENSG00000145794
ENSG00000171722	ENSG00000095203	ENSG00000157593
ENSG00000112320	ENSG00000116983	ENSG00000122257
ENSG00000156171	ENSG00000169432	ENSG00000137076
ENSG00000153904	ENSG00000100068	ENSG00000164309
ENSG00000221880	ENSG00000137486	ENSG00000132359
ENSG00000150676	ENSG00000138686	ENSG00000132549
ENSG00000104870	ENSG00000159921	ENSG00000150457
ENSG00000101440	ENSG00000180071	ENSG00000134247
ENSG00000198898	ENSG00000158270	ENSG00000144460
ENSG00000224940	ENSG00000133226	ENSG00000187957
ENSG00000198082	ENSG00000152910	ENSG00000255408
ENSG00000164900	ENSG00000186017	ENSG00000054277
ENSG00000109208	ENSG00000198108	ENSG00000142677
ENSG00000141198	ENSG00000142698	ENSG00000113739
ENSG00000181626	ENSG00000117148	ENSG00000185231
ENSG00000113196	ENSG00000118200	ENSG00000108576
ENSG00000177627	ENSG00000146385	ENSG00000162771
ENSG00000203995	ENSG00000031698	ENSG00000204701
ENSG00000187223	ENSG00000080608	ENSG00000198963
ENSG00000119392	ENSG00000169679	ENSG00000183668
ENSG00000139899	ENSG00000254726	ENSG00000136002
ENSG00000198339	ENSG00000198445	ENSG00000166197
ENSG00000138134	ENSG00000166140	ENSG00000151475
ENSG00000171885	ENSG00000141524	ENSG00000137460
ENSG00000163113	ENSG00000130150	ENSG00000179270
ENSG00000189120	ENSG00000171246	ENSG00000162761
ENSG00000125733	ENSG00000242715	ENSG00000159167
ENSG00000139437	ENSG00000172689	ENSG00000173585
ENSG00000049247	ENSG00000198855	ENSG00000169418
ENSG00000197451	ENSG00000184428	ENSG00000140506
ENSG00000078403	ENSG00000125772	ENSG00000076382
ENSG00000167658	ENSG00000166979	ENSG00000174607
ENSG00000154640	ENSG00000145649	ENSG00000150261
ENSG00000167916	ENSG00000105428	ENSG00000129422
ENSG00000164299	ENSG00000100225	ENSG00000157680
ENSG00000261794	ENSG00000108784	ENSG00000135838
ENSG00000169981	ENSG00000064601	ENSG00000123329
ENSG00000184163	ENSG00000179930	ENSG00000146592
ENSG00000135637	ENSG00000143257	ENSG00000120251
ENSG00000156853	ENSG00000196407	ENSG00000139985
ENSG00000255221	ENSG00000196557	ENSG00000131374
ENSG00000154027	ENSG00000100099	ENSG00000178021
ENSG00000106031	ENSG00000049167	ENSG00000140090
ENSG00000259431	ENSG00000138382	ENSG00000170485
ENSG00000227471	ENSG00000106052	ENSG00000101938
ENSG00000162341	ENSG00000064932	ENSG00000111859
ENSG00000148484	ENSG00000110848	ENSG00000099381
ENSG00000065518	ENSG00000135912	ENSG00000137497
ENSG00000179148	ENSG00000114209	ENSG00000160179
ENSG00000182472	ENSG00000170153	ENSG00000204983
ENSG00000165997	ENSG00000133816	ENSG00000196266
ENSG00000168701	ENSG00000148339	ENSG00000221836
ENSG00000110172	ENSG00000188372	ENSG00000164691
ENSG00000175785	ENSG00000188760	ENSG00000124900
ENSG00000156689	ENSG00000131473	ENSG00000177000
ENSG00000170684	ENSG00000177294	ENSG00000124228
ENSG00000100170	ENSG00000111012	ENSG00000091262
ENSG00000144550	ENSG00000175548	ENSG00000148737
ENSG00000248458	ENSG00000142687	ENSG00000141449
ENSG00000169499	ENSG00000204335	ENSG00000159915
ENSG00000148344	ENSG00000261934	ENSG00000163872

ENSG00000141744	ENSG00000154240	ENSG00000112773
ENSG00000075975	ENSG00000149806	ENSG00000140481
ENSG00000064012	ENSG00000108509	ENSG00000134874
ENSG00000184345	ENSG00000160294	ENSG00000204186
ENSG00000164144	ENSG00000164930	ENSG00000133943
ENSG00000087157	ENSG00000029559	ENSG00000204970
ENSG00000180205	ENSG00000167822	ENSG00000174255
ENSG00000069424	ENSG00000166341	ENSG00000187612
ENSG00000164615	ENSG00000089820	ENSG00000154646
ENSG00000172232	ENSG00000184933	ENSG00000121053
ENSG00000154529	ENSG00000183150	ENSG00000169398
ENSG00000196735	ENSG00000178187	ENSG00000088280
ENSG00000251655	ENSG00000181788	ENSG00000120156
ENSG00000164180	ENSG00000111863	ENSG00000163629
ENSG00000162897	ENSG00000163913	ENSG00000062038
ENSG00000222009	ENSG00000141314	ENSG00000088826
ENSG00000176720	ENSG00000138642	ENSG00000129566
ENSG00000068912	ENSG00000167967	ENSG00000135835
ENSG00000181222	ENSG00000254505	ENSG00000170037
ENSG00000198393	ENSG00000170525	ENSG00000213047
ENSG00000137764	ENSG00000134042	ENSG00000158485
ENSG00000153707	ENSG00000152207	ENSG00000122728
ENSG00000135457	ENSG00000182247	ENSG00000198948
ENSG00000100453	ENSG00000109061	ENSG00000106479
ENSG00000214050	ENSG00000120913	ENSG00000170054
ENSG00000130513	ENSG00000006740	ENSG00000127507
ENSG00000204889	ENSG00000203668	ENSG00000108797
ENSG00000115468	ENSG00000234127	ENSG00000138639
ENSG00000203685	ENSG00000054392	ENSG00000171954
ENSG00000162851	ENSG00000160191	ENSG00000254245
ENSG00000180658	ENSG00000095787	ENSG00000122870
ENSG00000070214	ENSG00000144645	ENSG00000038532
ENSG00000253537	ENSG00000250506	ENSG00000172365
ENSG00000269700	ENSG00000101074	ENSG00000151702
ENSG00000189366	ENSG00000177689	ENSG00000171940
ENSG00000161203	ENSG00000140575	ENSG00000132773
ENSG00000163453	ENSG00000243896	ENSG00000144028
ENSG00000243452	ENSG00000113282	ENSG00000204469
ENSG00000212938	ENSG00000103035	ENSG00000129219
ENSG00000255529	ENSG00000185189	ENSG00000100934
ENSG00000112365	ENSG00000111271	ENSG00000151150
ENSG00000130489	ENSG00000167525	ENSG00000133574
ENSG00000075407	ENSG00000183484	ENSG00000151376
ENSG00000108064	ENSG00000120254	ENSG00000137809
ENSG00000268182	ENSG00000174405	ENSG00000132694
ENSG00000162385	ENSG00000141338	ENSG00000162434
ENSG00000132467	ENSG00000060237	ENSG00000168959
ENSG00000068137	ENSG00000164045	ENSG00000174483
ENSG00000179292	ENSG00000141756	ENSG00000112062
ENSG00000104946	ENSG00000172113	ENSG00000148843
ENSG00000167186	ENSG00000114626	ENSG00000137802
ENSG00000197961	ENSG00000174450	ENSG00000158163
ENSG00000100079	ENSG00000146021	ENSG00000162949
ENSG00000188386	ENSG00000084652	ENSG00000100665
ENSG00000148459	ENSG00000172073	ENSG00000174482
ENSG00000187626	ENSG00000126243	ENSG00000126767
ENSG00000166965	ENSG00000186810	ENSG00000152208
ENSG00000172428	ENSG00000175426	ENSG00000106789
ENSG00000140365	ENSG00000159307	ENSG00000108001
ENSG00000154803	ENSG00000152315	ENSG00000165186
ENSG00000130590	ENSG00000140067	ENSG00000148606
ENSG00000091879	ENSG00000131149	ENSG00000198482
ENSG00000111358	ENSG00000158246	ENSG00000078687
ENSG00000112559	ENSG00000111432	ENSG00000097007

ENSG00000175505	ENSG00000243543	ENSG00000163645
ENSG00000176148	ENSG00000113108	ENSG00000004948
ENSG00000180433	ENSG00000104490	ENSG00000187021
ENSG00000174937	ENSG00000129667	ENSG00000162374
ENSG00000174325	ENSG00000132631	ENSG00000132854
ENSG00000198918	ENSG00000114993	ENSG00000065534
ENSG00000184954	ENSG00000174015	ENSG00000112902
ENSG00000086062	ENSG00000135119	ENSG00000178904
ENSG00000214700	ENSG00000163374	ENSG00000108389
ENSG00000152076	ENSG00000185163	ENSG00000178568
ENSG00000232423	ENSG00000007350	ENSG00000117009
ENSG00000175854	ENSG00000169925	ENSG00000087008
ENSG00000126709	ENSG00000111644	ENSG00000155660
ENSG00000204538	ENSG00000179979	ENSG00000009954
ENSG00000156052	ENSG00000156232	ENSG00000107951
ENSG00000155545	ENSG00000166845	ENSG00000102241
ENSG00000136682	ENSG00000105662	ENSG00000172572
ENSG00000189253	ENSG00000075223	ENSG00000183780
ENSG00000165487	ENSG00000188554	ENSG00000039560
ENSG00000168876	ENSG00000188282	ENSG00000204965
ENSG00000251258	ENSG00000124812	ENSG00000108244
ENSG00000156973	ENSG00000185215	ENSG00000148824
ENSG00000109113	ENSG00000066855	ENSG00000082805
ENSG00000169717	ENSG00000140795	ENSG00000107077
ENSG00000109255	ENSG00000034533	ENSG00000038358
ENSG00000170458	ENSG00000034693	ENSG00000133026
ENSG00000180488	ENSG00000172289	ENSG00000148773
ENSG00000108825	ENSG00000130957	ENSG00000054118
ENSG00000104695	ENSG00000147604	ENSG00000163995
ENSG00000178997	ENSG00000111254	ENSG00000148019
ENSG00000156482	ENSG00000039987	ENSG00000123576
ENSG00000198774	ENSG00000198771	ENSG00000137411
ENSG00000167034	ENSG00000173578	ENSG00000101546
ENSG00000065320	ENSG00000070087	ENSG00000175764
ENSG00000166482	ENSG00000197077	ENSG00000141458
ENSG00000059758	ENSG00000213782	ENSG00000134160
ENSG00000154007	ENSG00000167535	ENSG00000145147
ENSG00000215271	ENSG00000185482	ENSG00000164828
ENSG00000115827	ENSG00000168539	ENSG00000124181
ENSG00000172057	ENSG00000254206	ENSG00000183715
ENSG00000182749	ENSG00000049768	ENSG00000151322
ENSG00000136153	ENSG00000122585	ENSG00000137098
ENSG00000085231	ENSG00000169347	ENSG00000056972
ENSG00000157502	ENSG00000126062	ENSG00000243135
ENSG00000160339	ENSG00000135622	ENSG00000088053
ENSG00000116497	ENSG00000167815	ENSG00000113721
ENSG00000187860	ENSG00000138439	ENSG00000072858
ENSG00000146540	ENSG00000106328	ENSG00000115464
ENSG00000120053	ENSG00000147155	ENSG00000048342
ENSG00000055917	ENSG00000170190	ENSG00000135903
ENSG00000141994	ENSG00000010803	ENSG00000175946
ENSG00000169059	ENSG00000259207	ENSG00000100347
ENSG00000197782	ENSG00000107779	ENSG00000204542
ENSG00000188177	ENSG00000186283	ENSG00000214944
ENSG00000167774	ENSG00000182957	ENSG00000184144
ENSG00000164162	ENSG00000073578	ENSG00000129204
ENSG00000197353	ENSG00000019549	ENSG00000182732
ENSG00000125788	ENSG00000242419	ENSG00000104738
ENSG00000165275	ENSG00000170832	ENSG00000159495
ENSG00000205502	ENSG00000180818	ENSG00000165271
ENSG00000143889	ENSG00000072518	ENSG00000165801
ENSG00000104219	ENSG00000163207	ENSG00000157766
ENSG00000070193	ENSG00000080603	ENSG00000144229
ENSG00000136457	ENSG00000136535	ENSG00000102290

ENSG00000268163	ENSG00000118514	ENSG00000196338
ENSG00000130517	ENSG00000096696	ENSG00000088305
ENSG00000167653	ENSG00000204104	ENSG00000134909
ENSG00000198218	ENSG00000133246	ENSG00000182134
ENSG00000152214	ENSG00000180900	ENSG00000169180
ENSG00000132825	ENSG00000155849	ENSG00000120949
ENSG00000124260	ENSG00000183090	ENSG00000060709
ENSG00000161179	ENSG00000114933	ENSG00000148840
ENSG00000163202	ENSG00000187554	ENSG00000136444
ENSG00000171357	ENSG00000149516	ENSG00000116183
ENSG00000170379	ENSG00000148634	ENSG00000134569
ENSG00000137710	ENSG00000188730	ENSG00000180347
ENSG00000104611	ENSG00000155640	ENSG00000134365
ENSG00000147382	ENSG00000165238	ENSG00000167378
ENSG00000136297	ENSG00000198732	ENSG00000151276
ENSG00000136699	ENSG00000027847	ENSG00000074755
ENSG00000105849	ENSG00000136504	ENSG00000182013
ENSG00000255359	ENSG00000178919	ENSG00000196604
ENSG00000235878	ENSG00000180773	ENSG00000171451
ENSG00000224389	ENSG00000107719	ENSG00000180815
ENSG00000005108	ENSG00000176925	ENSG00000158560
ENSG00000164323	ENSG00000049283	ENSG00000137251
ENSG00000176410	ENSG00000173557	ENSG00000164078
ENSG00000113073	ENSG00000141161	ENSG00000197943
ENSG00000186230	ENSG00000124092	ENSG00000184156
ENSG00000165105	ENSG00000170967	ENSG00000143799
ENSG00000163807	ENSG00000115602	ENSG00000258417
ENSG00000111678	ENSG00000185838	ENSG00000170175
ENSG00000119431	ENSG00000198121	ENSG00000078142
ENSG00000151327	ENSG00000103460	ENSG00000070601
ENSG00000213965	ENSG00000164626	ENSG00000204947
ENSG00000117020	ENSG00000138279	ENSG00000167992
ENSG00000189007	ENSG00000182158	ENSG00000173262
ENSG00000136875	ENSG00000163635	ENSG00000087087
ENSG00000114698	ENSG00000173848	ENSG00000164076
ENSG00000213463	ENSG00000112182	ENSG00000196526
ENSG00000106689	ENSG00000131725	ENSG00000169851
ENSG00000187257	ENSG00000119509	ENSG00000105339
ENSG00000152926	ENSG00000109819	ENSG00000160218
ENSG00000154645	ENSG00000143376	ENSG00000241973
ENSG00000121390	ENSG00000112812	ENSG00000115183
ENSG00000182632	ENSG00000145103	ENSG00000169126
ENSG00000023697	ENSG00000100568	ENSG00000158683
ENSG00000197586	ENSG00000155760	ENSG00000138002
ENSG00000235268	ENSG00000132669	ENSG00000114487
ENSG00000198546	ENSG00000139211	ENSG00000197915
ENSG00000165113	ENSG00000164330	ENSG00000173114
ENSG00000213022	ENSG00000105053	ENSG00000073282
ENSG00000145692	ENSG00000132139	ENSG00000215421
ENSG00000215717	ENSG00000044115	ENSG00000082482
ENSG00000187005	ENSG00000143921	ENSG00000163701
ENSG00000107362	ENSG00000182359	ENSG00000183638
ENSG00000175664	ENSG00000007384	ENSG00000122194
ENSG00000183828	ENSG00000137699	ENSG00000075651
ENSG00000124334	ENSG00000115484	ENSG00000154678
ENSG00000165886	ENSG00000187003	ENSG00000176927
ENSG00000182315	ENSG00000165959	ENSG00000148053
ENSG00000182220	ENSG00000096006	ENSG00000005700
ENSG00000173597	ENSG00000204764	ENSG00000139641
ENSG00000138182	ENSG00000134588	ENSG00000143772
ENSG00000243927	ENSG00000167994	ENSG00000036828
ENSG00000186998	ENSG00000168646	ENSG00000068400
ENSG00000204595	ENSG00000073754	ENSG00000118058
ENSG00000006555	ENSG00000135476	ENSG00000189182

ENSG00000148411	ENSG00000122966	ENSG00000186529
ENSG00000164219	ENSG00000125355	ENSG00000116260
ENSG00000002016	ENSG00000188986	ENSG00000147081
ENSG00000234734	ENSG00000155886	ENSG00000130377
ENSG00000233041	ENSG00000115266	ENSG00000164741
ENSG00000123977	ENSG00000113580	ENSG00000213079
ENSG00000132334	ENSG00000146826	ENSG00000151338
ENSG00000108278	ENSG00000079689	ENSG00000122756
ENSG00000176659	ENSG00000092096	ENSG00000196547
ENSG00000168955	ENSG00000071054	ENSG00000164199
ENSG00000147684	ENSG00000121653	ENSG00000120327
ENSG00000169895	ENSG00000130734	ENSG00000105227
ENSG00000196704	ENSG00000197168	ENSG00000143473
ENSG00000159173	ENSG00000005471	ENSG00000188994
ENSG00000213123	ENSG00000129353	ENSG00000007933
ENSG00000063978	ENSG00000161807	ENSG00000115414
ENSG00000133985	ENSG00000139546	ENSG00000171843
ENSG00000197712	ENSG00000154975	ENSG00000162745
ENSG00000169220	ENSG00000006282	ENSG00000146950
ENSG00000149534	ENSG00000146070	ENSG00000145703
ENSG00000213639	ENSG00000182272	ENSG00000188039
ENSG00000099290	ENSG00000222040	ENSG00000105663
ENSG00000140299	ENSG00000167632	ENSG00000133454
ENSG00000178074	ENSG00000188993	ENSG00000148602
ENSG00000147100	ENSG00000164329	ENSG00000157445
ENSG00000123992	ENSG00000070371	ENSG00000147459
ENSG00000151033	ENSG00000038945	ENSG00000154736
ENSG00000126524	ENSG00000177239	ENSG00000173406
ENSG00000122188	ENSG00000013573	ENSG00000159899
ENSG00000197165	ENSG00000139197	ENSG00000136160
ENSG00000124882	ENSG00000121691	ENSG00000132561
ENSG00000100167	ENSG00000115616	ENSG00000148219
ENSG00000147144	ENSG00000204681	ENSG00000113212
ENSG00000163568	ENSG00000174343	ENSG00000120054
ENSG00000163864	ENSG00000163380	ENSG00000089472
ENSG00000128973	ENSG00000093072	ENSG00000138039
ENSG00000116990	ENSG00000005955	ENSG00000254521
ENSG00000166295	ENSG00000105383	ENSG00000127249
ENSG00000090432	ENSG00000134256	ENSG00000124486
ENSG00000151962	ENSG00000182902	ENSG00000085552
ENSG00000078668	ENSG00000144583	ENSG00000226372
ENSG00000090470	ENSG00000175262	ENSG00000231924
ENSG00000165682	ENSG00000124215	ENSG00000145740
ENSG00000197776	ENSG00000065882	ENSG00000173546
ENSG00000118961	ENSG00000134198	ENSG00000157765
ENSG00000204065	ENSG00000164916	ENSG00000115423
ENSG00000197813	ENSG00000159082	ENSG00000104728
ENSG00000157538	ENSG00000146276	ENSG00000085511
ENSG00000109072	ENSG00000131941	ENSG00000160856
ENSG00000258223	ENSG00000167601	ENSG00000153162
ENSG00000159556	ENSG00000221829	ENSG00000144452
ENSG00000164674	ENSG00000101452	ENSG00000130226
ENSG00000140044	ENSG00000168872	ENSG00000117600
ENSG00000189299	ENSG00000020633	ENSG00000055163
ENSG00000137094	ENSG00000106991	ENSG00000079432
ENSG00000151929	ENSG00000111344	ENSG00000107104
ENSG00000179044	ENSG00000258366	ENSG00000161031
ENSG00000163827	ENSG00000078900	ENSG00000138162
ENSG00000180919	ENSG00000063015	ENSG00000144749
ENSG00000172901	ENSG00000154310	ENSG00000135749
ENSG00000134375	ENSG00000040199	ENSG00000133863
ENSG00000145431	ENSG00000174948	ENSG00000205403
ENSG00000104331	ENSG00000106123	ENSG00000172320
ENSG00000166455	ENSG00000147255	ENSG00000065361

ENSG00000137404	ENSG00000108950	ENSG00000171811
ENSG00000081177	ENSG00000188385	ENSG00000172673
ENSG00000105669	ENSG00000071051	ENSG00000122512
ENSG00000118420	ENSG00000205029	ENSG00000131386
ENSG00000181961	ENSG00000146242	ENSG00000162105
ENSG00000143748	ENSG00000091129	ENSG00000196235
ENSG00000041988	ENSG00000147432	ENSG00000134668
ENSG00000063660	ENSG00000106927	ENSG00000102043
ENSG00000240871	ENSG00000204442	ENSG00000254692
ENSG00000164284	ENSG00000161664	ENSG00000112293
ENSG00000112115	ENSG00000184368	ENSG00000166963
ENSG00000198673	ENSG00000144642	ENSG00000102755
ENSG00000125846	ENSG00000107623	ENSG00000087460
ENSG00000125675	ENSG00000125900	ENSG00000160145
ENSG00000142188	ENSG00000150269	ENSG00000173040
ENSG00000182572	ENSG00000169247	ENSG00000129250
ENSG00000183291	ENSG00000115657	ENSG00000116299
ENSG00000113430	ENSG00000135720	ENSG00000205250
ENSG00000104432	ENSG00000112164	ENSG00000075702
ENSG00000263020	ENSG00000197283	ENSG00000143514
ENSG00000136158	ENSG00000197324	ENSG00000148842
ENSG00000159433	ENSG00000061936	ENSG00000140015
ENSG00000032389	ENSG00000092036	ENSG00000010327
ENSG00000170948	ENSG00000164307	ENSG00000103313
ENSG00000184986	ENSG00000177398	ENSG00000162512
ENSG00000128383	ENSG00000146618	ENSG00000151474
ENSG00000204414	ENSG00000143641	ENSG00000120733
ENSG00000103245	ENSG00000144868	ENSG00000111642
ENSG00000124251	ENSG00000167483	ENSG00000112246
ENSG00000198965	ENSG00000120500	ENSG00000019169
ENSG00000158526	ENSG00000188603	ENSG00000171365
ENSG00000188015	ENSG00000141985	ENSG00000082781
ENSG00000157111	ENSG00000110887	ENSG00000065618
ENSG00000125741	ENSG00000137177	ENSG00000132681
ENSG00000187569	ENSG00000134640	ENSG00000179796
ENSG00000198231	ENSG00000105613	ENSG00000120594
ENSG00000165704	ENSG00000136114	ENSG00000141200
ENSG00000145685	ENSG00000074527	ENSG00000155093
ENSG00000163254	ENSG00000162641	ENSG00000147231
ENSG00000173137	ENSG00000263155	ENSG00000144893
ENSG00000171425	ENSG00000170748	ENSG00000164171
ENSG00000115548	ENSG00000038219	ENSG00000151914
ENSG00000163900	ENSG00000165124	ENSG00000116641
ENSG00000181495	ENSG00000157617	ENSG00000183486
ENSG00000168394	ENSG00000184313	ENSG00000135333
ENSG00000153339	ENSG00000087258	ENSG00000101940
ENSG00000168060	ENSG00000133687	ENSG00000151320
ENSG00000213993	ENSG00000166946	ENSG00000081052
ENSG00000107614	ENSG00000135372	ENSG00000054523
ENSG00000103550	ENSG00000189181	ENSG00000132938
ENSG00000111341	ENSG00000101977	ENSG00000073614
ENSG00000083896	ENSG00000127903	ENSG00000110427
ENSG00000162510	ENSG00000155363	ENSG00000146005
ENSG00000130005	ENSG00000048828	ENSG00000128512
ENSG00000141646	ENSG00000119707	ENSG00000166444
ENSG00000145817	ENSG00000112218	ENSG00000128159
ENSG00000138078	ENSG00000135144	ENSG00000188782
ENSG00000256162	ENSG00000047365	ENSG00000130749
ENSG00000104951	ENSG00000148672	ENSG00000204577
ENSG00000182916	ENSG00000253873	ENSG00000132793
ENSG00000218537	ENSG00000174990	ENSG00000205517
ENSG00000084623	ENSG00000172985	ENSG00000142661
ENSG00000185900	ENSG00000198758	ENSG00000132801
ENSG00000183784	ENSG00000171401	ENSG00000177599

ENSG00000260007	ENSG00000101096	ENSG00000124177
ENSG00000172239	ENSG00000170959	ENSG00000196422
ENSG00000125991	ENSG00000103154	ENSG00000141384
ENSG00000171466	ENSG00000171505	ENSG00000185518
ENSG00000048462	ENSG00000100092	ENSG00000182866
ENSG00000116704	ENSG00000157985	ENSG00000181804
ENSG00000170074	ENSG00000101343	ENSG00000149506
ENSG00000048991	ENSG00000116194	ENSG00000187098
ENSG00000102317	ENSG00000186973	ENSG00000182447
ENSG00000162702	ENSG00000100504	ENSG00000183856
ENSG00000184178	ENSG00000106608	ENSG00000162706
ENSG00000148950	ENSG00000086717	ENSG00000102313
ENSG00000090776	ENSG00000177733	ENSG00000103310
ENSG00000184489	ENSG00000149380	ENSG00000142609
ENSG00000147403	ENSG00000089639	ENSG00000141543
ENSG00000086475	ENSG00000102837	ENSG00000198795
ENSG00000144848	ENSG00000174292	ENSG00000204248
ENSG00000161638	ENSG00000138623	ENSG00000118257
ENSG00000164176	ENSG00000132153	ENSG00000188785
ENSG00000144843	ENSG00000124608	ENSG00000184634
ENSG00000161920	ENSG00000168264	ENSG00000136943
ENSG00000161016	ENSG00000177453	ENSG00000131323
ENSG00000165623	ENSG00000155465	ENSG00000057657
ENSG00000109332	ENSG00000204842	ENSG00000117707
ENSG00000150667	ENSG00000003393	ENSG00000102385
ENSG00000111731	ENSG00000172578	ENSG00000134802
ENSG00000185198	ENSG00000007237	ENSG00000144857
ENSG00000171223	ENSG00000197993	ENSG00000125337
ENSG00000140612	ENSG00000163681	ENSG00000113231
ENSG00000124613	ENSG00000079385	ENSG00000177324
ENSG00000163875	ENSG00000173210	ENSG00000154734
ENSG00000136866	ENSG00000176136	ENSG00000090615
ENSG00000170542	ENSG00000185420	ENSG00000176049
ENSG00000188739	ENSG00000167106	ENSG00000127334
ENSG00000213593	ENSG00000088808	ENSG00000146966
ENSG00000217340	ENSG00000183258	ENSG00000244038
ENSG00000160360	ENSG00000159339	ENSG00000066248
ENSG00000215910	ENSG00000136813	ENSG00000004846
ENSG00000104524	ENSG00000060558	ENSG00000038382
ENSG00000147127	ENSG00000091106	ENSG00000004975
ENSG00000169371	ENSG00000138614	ENSG00000171759
ENSG00000269067	ENSG00000164318	ENSG00000204613
ENSG00000269624	ENSG00000197565	ENSG00000146221
ENSG00000134545	ENSG00000112139	ENSG00000269323
ENSG00000168116	ENSG00000172247	ENSG00000113555
ENSG00000213462	ENSG00000164509	ENSG00000133958
ENSG00000122912	ENSG00000151461	ENSG00000176769
ENSG00000151247	ENSG00000171962	ENSG00000166748
ENSG00000163046	ENSG00000136828	ENSG00000184445
ENSG00000203778	ENSG00000136010	ENSG00000166206
ENSG00000181915	ENSG00000166603	ENSG00000104998
ENSG00000119227	ENSG00000245848	ENSG00000204406
ENSG00000107187	ENSG00000221888	ENSG00000160007
ENSG00000168036	ENSG00000180988	ENSG00000126838
ENSG00000123739	ENSG00000155897	ENSG00000149311
ENSG00000157873	ENSG00000214215	ENSG00000104043
ENSG00000147378	ENSG00000099331	ENSG00000141337
ENSG00000164823	ENSG00000104415	ENSG00000184408
ENSG00000185155	ENSG00000122012	ENSG00000091128
ENSG00000014919	ENSG00000104689	ENSG00000182319
ENSG00000146386	ENSG00000133019	ENSG00000104369
ENSG00000170142	ENSG00000114948	ENSG00000170477
ENSG00000258834	ENSG00000132205	ENSG00000066056
ENSG00000267140	ENSG00000150990	ENSG00000198633

ENSG00000140465	ENSG00000196663	ENSG00000204969
ENSG00000124343	ENSG00000172175	ENSG00000143375
ENSG00000128908	ENSG00000164659	ENSG00000196914
ENSG00000205439	ENSG00000166349	ENSG00000089225
ENSG00000159212	ENSG00000186976	ENSG00000171992
ENSG00000147421	ENSG00000122367	ENSG00000107863
ENSG00000163273	ENSG00000122483	ENSG00000253117
ENSG00000205279	ENSG00000118160	ENSG00000021826
ENSG00000186010	ENSG00000110075	ENSG00000099991
ENSG00000139990	ENSG00000118900	ENSG00000182667
ENSG00000166949	ENSG00000002834	ENSG00000069248
ENSG00000128242	ENSG00000261832	ENSG00000143126
ENSG00000180481	ENSG00000178764	ENSG00000163492
ENSG00000100246	ENSG00000071991	ENSG00000144655
ENSG00000104499	ENSG00000085276	ENSG00000159450
ENSG00000165233	ENSG00000198883	ENSG00000116141
ENSG00000196228	ENSG00000146197	ENSG00000162733
ENSG00000148362	ENSG00000099995	ENSG00000181693
ENSG00000141086	ENSG00000174429	ENSG00000113319
ENSG00000114395	ENSG00000119650	ENSG00000163637
ENSG00000170779	ENSG00000165730	ENSG00000068793
ENSG00000114902	ENSG00000127080	ENSG00000118777
ENSG00000163463	ENSG00000141431	ENSG00000177614
ENSG00000145309	ENSG00000090863	ENSG00000104237
ENSG00000105650	ENSG00000112837	ENSG00000189091
ENSG00000204866	ENSG00000196967	ENSG00000173567
ENSG00000169570	ENSG00000102076	ENSG00000178235
ENSG00000086300	ENSG00000099910	ENSG00000198670
ENSG00000196260	ENSG00000204580	ENSG00000070748
ENSG00000092345	ENSG00000134389	ENSG00000124496
ENSG00000237240	ENSG00000233701	ENSG00000147130
ENSG00000161572	ENSG00000107742	ENSG00000084676
ENSG00000197461	ENSG00000121690	ENSG00000121270
ENSG00000198791	ENSG00000105722	ENSG00000140538
ENSG00000135930	ENSG00000144290	ENSG00000082397
ENSG00000203795	ENSG00000255346	ENSG00000141946
ENSG00000138678	ENSG00000136986	ENSG00000072201
ENSG00000259112	ENSG00000156453	ENSG00000196535
ENSG00000102910	ENSG00000108591	ENSG00000186918
ENSG00000162377	ENSG00000203805	ENSG00000163053
ENSG00000174233	ENSG00000135407	ENSG00000122778
ENSG00000177054	ENSG00000165383	ENSG00000175029
ENSG00000111816	ENSG00000189433	ENSG00000130052
ENSG00000183520	ENSG00000067057	ENSG00000127481
ENSG00000176994	ENSG00000105726	ENSG00000124571
ENSG00000187486	ENSG00000188283	ENSG00000168575
ENSG00000142168	ENSG00000242110	ENSG00000171860
ENSG00000024862	ENSG00000010295	ENSG00000197093
ENSG00000157315	ENSG00000110171	ENSG00000060718
ENSG00000066322	ENSG00000185737	ENSG00000140798
ENSG00000188917	ENSG00000153207	ENSG00000165966
ENSG00000164776	ENSG00000169836	ENSG00000104447
ENSG00000171634	ENSG00000198400	ENSG00000134115
ENSG00000112651	ENSG00000133067	ENSG00000112769
ENSG00000064835	ENSG00000227184	ENSG00000100926
ENSG00000000971	ENSG00000152128	ENSG00000146250
ENSG00000162065	ENSG00000133704	ENSG00000078114
ENSG00000065923	ENSG00000108292	ENSG00000114480
ENSG00000151418	ENSG00000163075	ENSG00000143199
ENSG00000175514	ENSG00000180638	ENSG00000050438
ENSG00000226364	ENSG00000167768	ENSG00000172995
ENSG00000161652	ENSG00000156475	ENSG00000044524
ENSG00000165526	ENSG00000166961	ENSG00000047644
ENSG00000153561	ENSG00000158859	ENSG00000038295

ENSG00000187642	ENSG00000052749	ENSG00000069431
ENSG00000089022	ENSG00000196767	ENSG00000081760
ENSG00000146963	ENSG00000072682	ENSG00000182752
	ENSG00000160202	ENSG00000171444
	ENSG00000100106	ENSG00000116783
	ENSG00000162909	ENSG00000132932
	ENSG00000133116	ENSG00000158445
	ENSG00000070882	ENSG00000042980
	ENSG00000141503	ENSG00000253731
	ENSG00000066933	ENSG00000197724
	ENSG00000015479	ENSG00000054356
	ENSG00000133056	ENSG00000171560
	ENSG00000179142	ENSG00000039537
	ENSG00000007129	ENSG00000165185
	ENSG00000169855	ENSG00000117724
	ENSG00000187664	ENSG00000132688
	ENSG00000165548	ENSG00000083457
	ENSG00000100433	ENSG00000157426
	ENSG00000141665	ENSG00000176225
	ENSG00000145451	ENSG00000101306
	ENSG00000176165	ENSG00000139182
	ENSG00000177051	ENSG00000196963
	ENSG00000176177	ENSG00000185070
	ENSG00000204463	ENSG00000091592
	ENSG00000141194	ENSG00000251664
	ENSG00000173930	ENSG00000072832
	ENSG00000143178	ENSG00000122145
	ENSG00000130544	ENSG00000132915
	ENSG00000243232	ENSG00000176771
	ENSG00000140279	ENSG00000144406
	ENSG00000112333	ENSG00000149294
	ENSG00000111049	ENSG00000140521
	ENSG00000160767	ENSG00000152785
	ENSG00000182197	ENSG00000187048
	ENSG00000129465	ENSG00000253846
	ENSG00000255398	ENSG00000108821
	ENSG00000185069	ENSG00000177992
	ENSG00000186862	ENSG00000145216
	ENSG00000136830	ENSG00000160299
	ENSG00000088882	ENSG00000133020
	ENSG00000254585	ENSG00000164694
	ENSG00000087589	ENSG00000081842
	ENSG00000149575	ENSG00000179222
	ENSG00000198589	ENSG00000156709
	ENSG00000079435	ENSG00000166839
	ENSG00000177990	ENSG00000112659
	ENSG00000013588	ENSG00000111961
	ENSG00000105738	ENSG00000148513
	ENSG00000133107	ENSG00000175267
	ENSG00000149541	ENSG00000111087
	ENSG00000212721	ENSG00000198793
	ENSG00000143006	ENSG00000127412
	ENSG00000198768	ENSG00000119522
	ENSG00000204962	ENSG00000068650
	ENSG00000166947	ENSG00000158089
	ENSG00000008197	ENSG00000147010
	ENSG00000099864	ENSG00000136715
	ENSG00000167965	ENSG00000163531
	ENSG00000167191	ENSG00000160505
	ENSG00000089169	ENSG00000057149
	ENSG00000166278	ENSG00000138759
	ENSG00000154864	ENSG00000112038
	ENSG00000164742	ENSG00000184571
	ENSG00000122965	ENSG00000159164

ENSG00000108671	ENSG00000154237
ENSG00000025039	ENSG00000048052
ENSG00000183765	ENSG00000157388
ENSG00000132437	ENSG00000111452
ENSG00000102230	ENSG00000198722
ENSG00000088827	ENSG00000143127
ENSG00000070614	ENSG00000077782
ENSG00000173175	ENSG00000111046
ENSG00000139767	ENSG00000156414
ENSG00000160882	ENSG00000156920
ENSG00000170920	ENSG00000152591
ENSG00000100505	ENSG00000138161
ENSG00000144674	ENSG00000048707
ENSG00000239264	ENSG00000155966
ENSG00000058668	ENSG00000113361
ENSG00000164830	ENSG00000259030
ENSG00000086967	ENSG00000172046
ENSG00000130985	ENSG00000134207
ENSG00000136827	ENSG00000163092
ENSG00000254996	ENSG00000075035
ENSG00000111249	ENSG00000186635
ENSG00000100364	ENSG00000130943
ENSG00000188037	ENSG00000039139
ENSG00000110328	ENSG00000243156
ENSG00000091986	ENSG00000171105
ENSG00000116574	ENSG00000077943
ENSG00000136235	ENSG00000153071
ENSG00000173193	ENSG00000122515
ENSG00000153066	ENSG00000143322
ENSG00000151948	ENSG00000168769
ENSG00000092068	ENSG00000030582
ENSG00000159459	ENSG00000121933
ENSG00000137944	ENSG00000189350
ENSG00000123411	ENSG00000147044
ENSG00000107159	ENSG00000099139
ENSG00000147481	ENSG00000239389
ENSG00000086015	ENSG00000101825
ENSG00000184155	ENSG00000130287
ENSG00000204897	ENSG00000168843
ENSG00000124198	ENSG00000101191
ENSG00000120705	ENSG00000168280
ENSG00000167769	ENSG00000136928
ENSG00000133561	ENSG00000130396
ENSG00000102287	ENSG00000206579
ENSG00000167080	ENSG00000166342
ENSG00000260238	ENSG00000129646
ENSG00000095002	ENSG00000109944
ENSG00000118322	ENSG00000160469
ENSG00000079393	ENSG00000120328
ENSG00000155659	ENSG00000099954
ENSG00000164117	ENSG00000110514
ENSG00000126856	ENSG00000184647
ENSG00000112041	ENSG00000147724
ENSG00000059691	ENSG00000172349
ENSG00000174502	ENSG00000118515
ENSG00000092820	ENSG00000079112
ENSG00000073803	ENSG00000172403
ENSG00000164946	ENSG00000170382
ENSG00000125648	ENSG00000163611
ENSG00000130518	ENSG00000073331
ENSG00000072840	ENSG00000166535
ENSG00000168412	ENSG00000153253
ENSG00000183706	ENSG00000125851
ENSG00000161640	ENSG00000158321

ENSG00000122218	ENSG00000206181
ENSG00000130164	ENSG00000184305
ENSG00000121753	ENSG00000087245
ENSG00000144057	ENSG00000244482
ENSG00000146151	ENSG00000112992
ENSG00000109929	ENSG00000101040
ENSG00000176248	ENSG00000101349
ENSG00000204946	ENSG00000167676
ENSG00000124813	ENSG00000166833
ENSG00000198074	ENSG00000175356
ENSG00000154124	ENSG00000154133
ENSG00000103723	ENSG00000135424
ENSG00000115318	ENSG00000171189
ENSG00000140678	ENSG00000013293
ENSG00000163702	ENSG00000074181
ENSG00000186081	ENSG00000149182
ENSG00000221986	ENSG00000160796
ENSG00000243207	ENSG00000131873
ENSG00000006116	ENSG00000118004
ENSG00000198719	ENSG00000135945
ENSG00000123453	ENSG00000164588
ENSG00000094796	ENSG00000171456
ENSG00000163719	ENSG00000129351
ENSG00000197497	ENSG00000165379
ENSG00000162992	ENSG00000162267
ENSG00000146360	ENSG00000141968
ENSG00000131379	ENSG00000146426
ENSG00000156515	ENSG00000156113
ENSG00000215009	ENSG00000076555
ENSG00000008405	ENSG00000171735
ENSG00000073598	ENSG00000071242
ENSG00000100311	ENSG00000165194
ENSG00000102078	ENSG00000142405
ENSG00000139926	ENSG00000145536
ENSG00000198848	ENSG00000114805
ENSG00000178175	ENSG00000089154
ENSG00000197381	ENSG00000012124
ENSG00000030304	ENSG00000155052
ENSG00000159387	ENSG00000187391
ENSG00000198223	ENSG00000170011
ENSG00000166265	ENSG00000218336
ENSG00000259741	ENSG00000122641
ENSG00000165917	ENSG00000151208
ENSG00000174282	ENSG00000148143
ENSG00000184937	ENSG00000165699
ENSG00000072062	ENSG00000153292
ENSG00000165280	ENSG00000156395
ENSG00000011021	ENSG00000077522
ENSG00000153246	ENSG00000134376
ENSG00000101400	ENSG00000140443
ENSG00000060749	ENSG00000204131
ENSG00000146090	ENSG00000107593
ENSG00000132763	ENSG00000089101
ENSG00000142606	ENSG00000105877
ENSG00000144063	ENSG00000140836
ENSG00000075043	ENSG00000078018
ENSG00000243772	ENSG00000134250
ENSG00000059573	ENSG00000124788
ENSG00000167785	ENSG00000132164
ENSG00000197959	ENSG00000084774
ENSG00000143630	ENSG00000148848
ENSG00000178209	ENSG00000161270
ENSG00000092758	ENSG00000047617
ENSG00000008735	ENSG00000124140

ENSG00000141441	ENSG00000113211
ENSG00000105641	ENSG00000170004
ENSG00000141258	ENSG00000115850
ENSG00000124256	ENSG00000197694
ENSG00000166402	ENSG00000253729
ENSG00000182492	ENSG00000203786
ENSG00000142556	ENSG00000178031
ENSG00000185467	ENSG00000118407
ENSG00000005156	ENSG00000250120
ENSG00000090861	ENSG00000162670
ENSG00000189280	ENSG00000112852
ENSG00000065485	ENSG00000198842
ENSG00000158473	ENSG00000198026
ENSG00000110025	ENSG00000080573
ENSG00000165630	ENSG00000074657
ENSG00000121577	ENSG00000166689
ENSG00000155974	ENSG00000148468
ENSG00000164399	ENSG00000185313
ENSG00000128591	ENSG00000198879
ENSG00000157510	ENSG00000181192
ENSG00000091010	ENSG00000168356
ENSG00000006432	ENSG00000109667
ENSG00000183773	ENSG00000177084
ENSG00000108387	ENSG00000264424
ENSG00000258555	ENSG00000157856
ENSG00000198373	ENSG00000005339
ENSG00000180251	ENSG00000173064
ENSG00000010322	ENSG00000079841
ENSG00000165675	ENSG00000055609
ENSG00000203710	ENSG00000167548
ENSG00000184574	ENSG00000144481
ENSG00000102195	ENSG00000085185
ENSG00000077044	ENSG00000047936
ENSG00000109323	ENSG00000135636
ENSG00000147912	ENSG00000171495
ENSG00000205339	ENSG00000172716
ENSG00000144668	ENSG00000107554
ENSG00000173548	ENSG00000168016
ENSG00000181698	ENSG00000132874
ENSG00000130720	ENSG00000197893
ENSG00000144369	ENSG00000083067
ENSG00000173757	ENSG00000087495
ENSG00000117322	ENSG00000122786
ENSG00000102466	ENSG00000197386
ENSG00000172752	ENSG00000156510
ENSG00000141027	ENSG00000142449
ENSG00000024422	ENSG00000144191
ENSG00000124243	ENSG00000138119
ENSG00000197892	ENSG00000079102
ENSG00000120549	ENSG00000138741
ENSG00000175387	ENSG00000146938
ENSG00000101230	ENSG00000163673
ENSG00000160886	ENSG00000130779
ENSG00000148082	ENSG00000166147
ENSG00000145833	ENSG00000163618
ENSG00000081189	ENSG00000179709
ENSG00000104783	ENSG00000182463
ENSG00000187792	ENSG00000140853
ENSG00000130559	ENSG00000140937
ENSG00000120659	ENSG00000065526
ENSG00000179409	ENSG00000204961
ENSG00000106105	ENSG00000160111
ENSG00000019991	ENSG00000119866
ENSG00000188779	ENSG00000185823

ENSG00000105767	ENSG00000113205
ENSG00000130182	ENSG00000007062
ENSG00000196954	ENSG00000142973
ENSG00000107862	ENSG00000171094
ENSG00000122557	ENSG00000205038
ENSG00000205268	ENSG00000171815
ENSG00000095627	ENSG00000171435
ENSG00000100714	ENSG00000113100
ENSG00000140545	ENSG00000160791
ENSG00000131778	ENSG00000156299
ENSG00000111252	ENSG00000248383
ENSG00000128422	ENSG00000185448
ENSG00000186038	ENSG00000134853
ENSG00000102243	ENSG00000141837
ENSG00000143643	ENSG00000178104
ENSG00000100084	ENSG00000144908
ENSG00000080839	ENSG00000169282
ENSG00000131373	ENSG00000126562
ENSG00000165509	ENSG00000036448
ENSG00000011638	ENSG00000175065
ENSG00000171132	ENSG00000049323
ENSG00000152256	ENSG00000070729
ENSG00000171988	ENSG00000138650
ENSG00000079805	ENSG00000106290
ENSG00000066117	ENSG00000198796
ENSG00000091039	ENSG00000179869
ENSG00000076650	ENSG00000171487
ENSG00000108582	ENSG00000138411
ENSG00000166446	ENSG00000153820
ENSG00000167680	ENSG00000184304
ENSG00000135108	ENSG00000165029
ENSG00000102226	ENSG00000116962
ENSG00000204967	ENSG00000157404
ENSG00000183023	ENSG00000088538
ENSG00000069020	ENSG00000102001
ENSG00000023318	ENSG00000150995
ENSG00000147065	ENSG00000145362
ENSG00000017483	ENSG00000101542
ENSG00000196242	ENSG00000149292
ENSG00000065029	ENSG00000050030
ENSG00000179115	ENSG00000068654
ENSG00000103994	ENSG00000152217
ENSG00000058600	ENSG00000088367
ENSG00000099341	ENSG00000147485
ENSG00000144567	ENSG00000128052
ENSG00000114349	ENSG00000084636
ENSG00000181963	ENSG00000100678
ENSG00000232748	ENSG00000120324
ENSG00000102158	ENSG00000139364
ENSG00000172602	ENSG00000151655
ENSG00000173698	ENSG00000185274
ENSG00000133321	ENSG00000012232
ENSG00000177303	ENSG00000072952
ENSG00000105549	ENSG00000172554
ENSG00000065615	ENSG00000163219
ENSG00000152192	ENSG00000151240
ENSG00000092607	ENSG00000133401
ENSG00000166479	ENSG00000164692
ENSG00000204624	ENSG00000109265
ENSG00000143776	ENSG00000198947
ENSG00000100105	ENSG00000156650
ENSG00000184672	ENSG00000171587
ENSG00000173402	ENSG00000134369
ENSG00000099949	ENSG00000257335

ENSG00000075461	ENSG00000137727
ENSG00000109576	ENSG00000165300
ENSG00000131067	ENSG00000178971
ENSG00000169598	ENSG00000169083
ENSG00000176281	ENSG00000187372
ENSG00000170145	ENSG00000031081
ENSG00000126947	ENSG00000069018
ENSG00000170743	ENSG00000104774
ENSG00000153046	ENSG00000169436
ENSG00000185811	ENSG00000214814
ENSG00000213390	ENSG00000189132
ENSG00000144230	ENSG00000047578
ENSG00000183671	ENSG00000127603
ENSG00000138308	ENSG00000145555
ENSG00000162722	ENSG00000079308
ENSG00000138131	ENSG00000155511
ENSG00000090316	ENSG00000144285
ENSG00000169258	ENSG00000118689
ENSG00000188676	ENSG00000150086
ENSG00000116679	ENSG00000125730
ENSG00000004777	ENSG00000165757
ENSG00000169327	ENSG00000067798
ENSG00000161914	ENSG00000124942
ENSG00000184108	ENSG00000196367
ENSG00000120210	ENSG00000008226
ENSG00000183853	ENSG00000075340
ENSG00000034053	ENSG00000177354
ENSG00000150867	ENSG00000105851
ENSG00000113296	ENSG00000127241
ENSG00000164362	ENSG00000113248
ENSG00000104888	ENSG00000158125
ENSG00000126337	ENSG00000156218
ENSG00000198933	ENSG00000002746
ENSG00000170786	ENSG00000204956
ENSG00000253148	ENSG00000010818
ENSG00000100813	ENSG00000102452
ENSG00000242019	ENSG00000020256
ENSG00000269699	ENSG00000198198
ENSG00000141568	ENSG00000081818
ENSG00000136021	ENSG00000113327
ENSG00000175294	ENSG00000169933
ENSG00000163599	ENSG00000150394
ENSG00000135387	ENSG00000196876
ENSG00000182022	ENSG00000055957
ENSG00000142484	ENSG00000166501
ENSG00000113209	ENSG00000138615
ENSG00000166387	ENSG00000125414
ENSG00000148308	ENSG00000029534
ENSG00000244411	ENSG00000164061
ENSG00000089006	ENSG00000081479
ENSG00000175787	ENSG00000085563
ENSG00000154767	ENSG00000133124
ENSG00000204420	ENSG00000155816
ENSG00000169550	ENSG00000146648
ENSG00000153446	ENSG00000106571
ENSG00000171603	ENSG00000116147
ENSG00000116748	ENSG00000081853
ENSG00000241635	ENSG00000196730
ENSG00000185775	ENSG00000206384
ENSG00000129691	ENSG00000196569
ENSG00000188906	ENSG00000183495
ENSG00000163449	ENSG00000184956
ENSG00000171496	ENSG00000196090
ENSG00000130714	ENSG00000103449

ENSG00000140829	ENSG00000120332
ENSG00000081138	ENSG00000075673
ENSG00000099337	ENSG00000214338
ENSG00000142784	ENSG00000100095
ENSG00000204424	ENSG00000105429
ENSG00000142235	ENSG00000115306
ENSG00000169946	ENSG00000151388
ENSG00000135966	ENSG00000173821
ENSG00000135898	ENSG00000183454
ENSG00000101181	ENSG00000168477
ENSG00000104880	ENSG00000206190
ENSG00000183251	ENSG00000140873
ENSG00000123146	ENSG00000089250
ENSG00000198205	ENSG00000255330
ENSG00000115282	ENSG00000162711
ENSG00000197912	ENSG00000154654
ENSG00000177212	ENSG00000058335
ENSG00000113838	ENSG00000183914
ENSG00000170340	ENSG00000114270
ENSG00000135624	ENSG00000124203
ENSG00000175809	ENSG00000136531
ENSG00000139915	ENSG00000164796
ENSG00000175691	ENSG00000128731
ENSG00000140943	ENSG00000150893
ENSG00000171310	ENSG00000152822
ENSG00000175866	ENSG00000130508
ENSG00000136305	ENSG00000023839
ENSG00000101265	ENSG00000121297
ENSG00000165152	ENSG00000054654
ENSG00000179055	ENSG00000170927
ENSG00000175634	ENSG00000187955
ENSG00000128271	ENSG00000141510
ENSG00000169919	ENSG00000185920
ENSG00000118113	ENSG00000198691
ENSG00000136630	ENSG00000109927
ENSG00000165494	ENSG00000204291
ENSG00000168454	ENSG00000185567
ENSG00000165661	ENSG00000150760
ENSG00000158571	ENSG00000171914
ENSG00000134817	ENSG00000143341
ENSG00000101974	ENSG00000174469
ENSG00000198740	ENSG00000078295
ENSG00000105991	ENSG00000118946
ENSG00000105392	ENSG00000136011
ENSG00000121871	ENSG00000008300
ENSG00000145194	ENSG00000158486
ENSG00000204963	ENSG00000152092
ENSG00000104714	ENSG00000041982
ENSG00000131503	ENSG00000083857
ENSG00000112280	ENSG00000167522
ENSG00000185803	ENSG00000143631
ENSG00000160752	ENSG00000196208
ENSG00000101298	ENSG00000168702
ENSG00000076928	ENSG00000169876
ENSG00000154134	ENSG00000151067
ENSG00000156398	ENSG00000115705
ENSG00000083812	ENSG00000123243
ENSG00000139173	ENSG00000138829
ENSG00000044090	ENSG00000021645
ENSG00000103249	ENSG00000006788
ENSG00000174807	ENSG00000169862
ENSG00000104892	ENSG00000130635
ENSG00000109684	ENSG00000107611
ENSG00000080298	ENSG00000196159

ENSG00000138311	ENSG00000145113
ENSG00000249139	ENSG00000165323
ENSG00000168787	ENSG00000149256
ENSG00000124201	ENSG00000146555
ENSG00000180116	ENSG00000131711
ENSG00000213983	ENSG00000198626
ENSG00000166716	ENSG00000091656
ENSG00000127946	ENSG00000179915
ENSG00000157570	ENSG00000110799
ENSG00000183160	ENSG00000198216
ENSG00000160211	ENSG00000145934
ENSG00000188305	ENSG00000197410
ENSG00000221840	ENSG00000042832
ENSG00000249158	ENSG00000157423
ENSG00000178338	ENSG00000151952
ENSG00000182352	ENSG00000131018
ENSG00000135365	ENSG00000197653
ENSG00000133466	ENSG00000084674
ENSG00000167549	ENSG00000101680
ENSG00000196588	ENSG00000163359
ENSG00000158008	ENSG00000198838
ENSG00000147324	ENSG00000007174
ENSG00000137634	ENSG00000090920
ENSG00000117054	ENSG00000121904
ENSG00000225190	ENSG00000042781
ENSG00000096070	ENSG00000183091
ENSG00000256223	ENSG00000183117
ENSG00000168487	ENSG00000181143
ENSG00000109991	ENSG00000155657
ENSG00000100354	
ENSG00000126778	
ENSG00000166813	
ENSG00000141434	
ENSG00000182578	
ENSG00000166831	
ENSG00000126787	
ENSG00000179046	
ENSG00000176887	
ENSG00000165092	
ENSG00000181761	
ENSG00000090054	
ENSG00000130560	
ENSG00000188558	
ENSG00000100220	
ENSG00000104408	
ENSG00000177030	
ENSG00000156466	
ENSG00000164116	
ENSG00000074590	
ENSG00000105825	
ENSG00000197887	
ENSG00000169223	
ENSG00000187889	
ENSG00000124212	
ENSG00000154917	
ENSG00000104517	
ENSG00000165794	
ENSG00000104938	
ENSG00000164574	
ENSG00000142627	
ENSG00000124772	
ENSG00000183273	
ENSG00000163975	
ENSG00000178078	

ENSG00000095319
ENSG00000184675
ENSG00000173805
ENSG00000145526
ENSG00000197140
ENSG00000124749
ENSG00000173876
ENSG00000146205
ENSG00000149136
ENSG00000119446
ENSG00000035664
ENSG00000125571
ENSG00000115380
ENSG00000158077
ENSG00000149131
ENSG00000169047
ENSG00000185252
ENSG00000100014
ENSG00000173226
ENSG00000197901
ENSG00000106006
ENSG00000083807
ENSG00000133083
ENSG00000204941
ENSG00000138095
ENSG00000123104
ENSG00000112658
ENSG00000173599
ENSG00000174348
ENSG00000085662
ENSG00000061273
ENSG00000015171
ENSG00000103274
ENSG00000011201
ENSG00000135747
ENSG00000197991
ENSG00000155366
ENSG00000177200
ENSG00000198917
ENSG00000177807
ENSG00000128274
ENSG00000026036
ENSG00000105668
ENSG00000127838
ENSG00000188716
ENSG00000165821
ENSG00000135423
ENSG00000187398
ENSG00000144820
ENSG00000090581
ENSG00000136634
ENSG00000151005
ENSG00000164920
ENSG00000147202
ENSG00000130669
ENSG00000174652
ENSG00000167723
ENSG00000075856
ENSG00000108840
ENSG00000130810
ENSG00000168135
ENSG00000164818
ENSG00000100304
ENSG00000189108

ENSG00000109572
ENSG00000100083
ENSG00000134780
ENSG00000173080
ENSG00000159069
ENSG00000075618
ENSG00000100519
ENSG00000187210
ENSG00000181072
ENSG00000156802
ENSG00000241945
ENSG00000197555
ENSG00000144935
ENSG00000054793
ENSG00000129467
ENSG00000139344
ENSG00000181666
ENSG00000149150
ENSG00000169218
ENSG00000148814
ENSG00000104356
ENSG00000008056
ENSG00000130803
ENSG00000144962
ENSG00000054967
ENSG00000141349
ENSG00000153575
ENSG00000179833
ENSG00000100241
ENSG00000033122
ENSG00000080007
ENSG00000149782
ENSG00000099326
ENSG00000179918
ENSG00000005436
ENSG00000132510
ENSG00000147576
ENSG00000104365
ENSG00000125945
ENSG00000077279
ENSG00000171161
ENSG00000151062
ENSG00000142156
ENSG00000196497
ENSG00000196689
ENSG00000136404
ENSG00000164175
ENSG00000163518
ENSG00000149260
ENSG00000175344
ENSG00000100629
ENSG00000182256
ENSG00000133069
ENSG00000206422
ENSG00000103426
ENSG00000185483
ENSG00000172543
ENSG00000018625
ENSG00000112706
ENSG00000157353
ENSG00000073910
ENSG00000182389
ENSG00000113119
ENSG00000116396

ENSG00000147118
ENSG00000184985
ENSG00000088038
ENSG00000164744
ENSG00000157654
ENSG00000158865
ENSG00000168916
ENSG00000184507
ENSG00000160710
ENSG00000064607
ENSG00000153317
ENSG00000183309
ENSG00000075539
ENSG00000130294
ENSG00000011405
ENSG00000154118
ENSG00000116406
ENSG00000053918
ENSG00000189184
ENSG00000118690
ENSG00000178802
ENSG00000225614
ENSG00000168497
ENSG00000102034
ENSG00000159409
ENSG00000145626
ENSG00000099308
ENSG00000163581
ENSG00000113946
ENSG00000176834
ENSG00000137878
ENSG00000257008
ENSG00000143147
ENSG00000164151
ENSG00000109794
ENSG00000130653
ENSG00000196182
ENSG00000164050
ENSG00000062370
ENSG00000153930
ENSG00000128596
ENSG00000146038
ENSG00000163462
ENSG00000095564
ENSG00000168754
ENSG00000124920
ENSG00000168661
ENSG00000120457
ENSG00000179873
ENSG00000176246
ENSG00000006125
ENSG00000108312
ENSG00000185621
ENSG00000134243
ENSG00000092621
ENSG00000100191
ENSG00000119698
ENSG00000145348
ENSG00000137766
ENSG00000250423
ENSG00000008196
ENSG00000239998
ENSG00000167306
ENSG00000125503

ENSG00000167394
ENSG00000144119
ENSG00000205359
ENSG00000106113
ENSG00000162836
ENSG00000049540
ENSG00000226288
ENSG00000256660
ENSG00000175745
ENSG00000105135
ENSG00000158458
ENSG00000128268
ENSG00000181786
ENSG00000125255
ENSG00000088812
ENSG00000256349
ENSG00000104722
ENSG00000105122
ENSG00000117091
ENSG00000162148
ENSG00000113263
ENSG00000143379
ENSG00000196132
ENSG00000143970
ENSG00000123143
ENSG00000101445
ENSG00000149564
ENSG00000185972
ENSG00000118473
ENSG00000117013
ENSG00000105289
ENSG00000054938
ENSG00000185477
ENSG00000164627
ENSG00000101276
ENSG00000127663
ENSG00000013725
ENSG00000127124
ENSG00000075429
ENSG00000187553
ENSG00000169184
ENSG00000128594
ENSG00000163354
ENSG00000108846
ENSG00000168481
ENSG00000133878
ENSG00000084693
ENSG00000095951
ENSG00000149476
ENSG00000108641
ENSG00000172159
ENSG00000073111
ENSG00000106128
ENSG00000114853
ENSG00000173166
ENSG00000119203
ENSG00000162736
ENSG00000167208
ENSG00000180353
ENSG00000112149
ENSG00000221968
ENSG00000196932
ENSG00000002822
ENSG00000185294

ENSG00000013288
ENSG00000180318
ENSG00000181867
ENSG00000159625
ENSG00000129951
ENSG00000153789
ENSG00000120322
ENSG00000132330
ENSG00000142319
ENSG00000241978
ENSG00000166886
ENSG00000116871
ENSG00000065457
ENSG00000168237
ENSG00000187742
ENSG00000188340
ENSG00000198723
ENSG00000078269
ENSG00000118402
ENSG00000135100
ENSG00000165125
ENSG00000196739
ENSG00000149930
ENSG00000107731
ENSG00000184867
ENSG00000100889
ENSG00000179715
ENSG00000176928
ENSG00000064300
ENSG00000163982
ENSG00000157703
ENSG00000122733
ENSG00000101425
ENSG00000156486
ENSG00000157557
ENSG00000188559
ENSG00000128805
ENSG00000005844
ENSG00000119042
ENSG00000087303
ENSG00000125703
ENSG00000175198
ENSG00000165695
ENSG00000151834
ENSG00000102870
ENSG00000186638
ENSG00000197705
ENSG00000166986
ENSG00000152592
ENSG00000182704
ENSG00000179520
ENSG00000130703
ENSG00000181752
ENSG00000174564
ENSG00000068971
ENSG00000039068
ENSG00000185261
ENSG00000074771
ENSG00000197748
ENSG00000144821
ENSG00000134775
ENSG00000185736
ENSG00000149115
ENSG00000177143

ENSG00000132182
ENSG00000170775
ENSG00000095303
ENSG00000132130
ENSG00000174038
ENSG00000165091
ENSG00000173212
ENSG00000005961
ENSG00000198569
ENSG00000170454
ENSG00000203780
ENSG00000157103
ENSG00000156194
ENSG00000185085
ENSG00000160224
ENSG00000145242
ENSG00000161217
ENSG00000105357
ENSG00000181090
ENSG00000167383
ENSG00000101945
ENSG00000172548
ENSG00000137261
ENSG00000159733
ENSG00000148187
ENSG00000122674
ENSG00000103241
ENSG00000105499
ENSG00000198798
ENSG00000197587
ENSG00000187855
ENSG00000174898
ENSG00000150510
ENSG00000107829
ENSG00000082641
ENSG00000101216
ENSG00000134007
ENSG00000163810
ENSG00000148600
ENSG00000198089
ENSG00000131864
ENSG00000138375
ENSG00000198743
ENSG00000196136
ENSG00000134954
ENSG00000160208
ENSG00000180871
ENSG00000049192
ENSG00000114166
ENSG00000139597
ENSG00000132518
ENSG00000106268
ENSG00000136267
ENSG00000197102
ENSG00000150961
ENSG00000169188
ENSG00000166866
ENSG00000134249
ENSG00000095917
ENSG00000122678
ENSG00000159708
ENSG00000043039
ENSG00000101292
ENSG00000137252

ENSG00000151812
ENSG00000105880
ENSG00000131467
ENSG00000158966
ENSG00000149133
ENSG00000105953
ENSG00000103365
ENSG00000167613
ENSG00000129103
ENSG00000057593
ENSG00000188157
ENSG00000182077
ENSG00000100393
ENSG00000170745
ENSG00000144283
ENSG00000139144
ENSG00000144036
ENSG00000160818
ENSG00000130119
ENSG00000151640
ENSG00000145681
ENSG00000204574
ENSG00000184916
ENSG00000154928
ENSG00000197948
ENSG00000159063
ENSG00000196998
ENSG00000187323
ENSG00000249471
ENSG00000198752
ENSG00000187908
ENSG00000135643
ENSG00000133030
ENSG00000157827
ENSG00000138646
ENSG00000101004
ENSG00000077235
ENSG00000135324
ENSG00000090686
ENSG00000116584
ENSG00000065989
ENSG00000003400
ENSG00000157219
ENSG00000119681
ENSG00000113302
ENSG00000125247
ENSG00000139178
ENSG00000074800
ENSG00000225485
ENSG00000138835
ENSG00000101746
ENSG00000136048
ENSG00000149091
ENSG00000143801
ENSG00000134532
ENSG00000204396
ENSG00000183576
ENSG00000107521
ENSG00000102468
ENSG00000171109
ENSG00000117501
ENSG00000018280
ENSG00000009413
ENSG00000103489

ENSG00000112685
ENSG00000168269
ENSG00000059378
ENSG00000141219
ENSG00000132704
ENSG00000174669
ENSG00000110876
ENSG00000171045
ENSG00000103375
ENSG00000180357
ENSG00000181958
ENSG00000180730
ENSG00000254737
ENSG00000171346
ENSG00000196724
ENSG00000063169
ENSG00000247596
ENSG00000185052
ENSG00000018189
ENSG00000169682
ENSG00000105366
ENSG00000153157
ENSG00000159618
ENSG00000103044
ENSG00000111664
ENSG00000140057
ENSG00000131831
ENSG00000119283
ENSG00000196263
ENSG00000160783
ENSG00000010292
ENSG00000025293
ENSG00000197446
ENSG00000102699
ENSG00000198844
ENSG00000096968
ENSG00000186204
ENSG00000153266
ENSG00000100767
ENSG00000072121
ENSG00000058404
ENSG00000087085
ENSG00000092051
ENSG00000074410
ENSG00000182963
ENSG00000049618
ENSG00000006611
ENSG00000196411
ENSG00000167258
ENSG00000174938
ENSG00000044574
ENSG00000119899
ENSG00000174837
ENSG00000197566
ENSG00000180509
ENSG00000112494
ENSG00000123815
ENSG00000182489
ENSG00000172771
ENSG00000167608
ENSG00000171481
ENSG00000116353
ENSG00000158290
ENSG00000167995

ENSG00000109685
ENSG00000215041
ENSG00000165370
ENSG00000066827
ENSG00000183230
ENSG00000160712
ENSG00000169231
ENSG00000168334
ENSG00000185019
ENSG00000182938
ENSG00000132842
ENSG00000101605
ENSG00000171385
ENSG00000196834
ENSG00000184735
ENSG00000197579
ENSG00000151490
ENSG00000104936
ENSG00000179981
ENSG00000112562
ENSG00000111218
ENSG00000005884
ENSG00000176566
ENSG00000183840
ENSG00000145868
ENSG00000137501
ENSG00000130856
ENSG00000128683
ENSG00000128573
ENSG00000196600
ENSG00000011566
ENSG00000139874
ENSG00000125319
ENSG00000101255
ENSG00000170289
ENSG00000165810
ENSG00000117154
ENSG00000204120
ENSG00000110148
ENSG00000149633
ENSG00000196098
ENSG00000197496
ENSG00000161243
ENSG00000160883
ENSG00000146918
ENSG00000185038
ENSG00000143190
ENSG00000215305
ENSG00000138193
ENSG00000182871
ENSG00000015133
ENSG00000166507
ENSG00000167110
ENSG00000040633
ENSG00000162896
ENSG00000254536
ENSG00000169330
ENSG00000021762
ENSG00000182348
ENSG00000121879
ENSG00000204560
ENSG00000105486
ENSG00000188011
ENSG00000187566

ENSG00000070778
ENSG00000185338
ENSG00000140522
ENSG00000115935
ENSG00000143674
ENSG00000112539
ENSG00000115896
ENSG00000163060
ENSG00000136040
ENSG00000184194
ENSG00000135426
ENSG00000167825
ENSG00000064393
ENSG00000084070
ENSG00000099994
ENSG00000153234
ENSG00000196517
ENSG00000166359
ENSG00000188868
ENSG00000169439
ENSG00000072364
ENSG00000152223
ENSG00000120800
ENSG00000095777
ENSG00000175894
ENSG00000104972
ENSG00000152767
ENSG00000165097
ENSG00000168418
ENSG00000117834
ENSG00000143858
ENSG00000198952
ENSG00000153060
ENSG00000138111
ENSG00000064218
ENSG00000106772
ENSG00000112379
ENSG00000084731
ENSG00000128833
ENSG00000134313
ENSG00000111962
ENSG00000144218
ENSG00000204859
ENSG00000108018
ENSG00000108852
ENSG00000149571
ENSG00000167333
ENSG00000184588
ENSG00000118007
ENSG00000130816
ENSG00000134253
ENSG00000197818
ENSG00000179583
ENSG00000188783
ENSG00000253293
ENSG00000136997
ENSG00000032219
ENSG00000205420
ENSG00000128802
ENSG00000117000
ENSG00000164715
ENSG00000101916
ENSG00000090402
ENSG00000142173

ENSG00000102890
ENSG00000105088
ENSG00000180921
ENSG00000009335
ENSG00000124574
ENSG00000002587
ENSG00000134955
ENSG00000116580
ENSG00000172534
ENSG00000146282
ENSG00000169181
ENSG00000134262
ENSG00000196576
ENSG00000197136
ENSG00000101448
ENSG00000163071
ENSG00000157741
ENSG00000132361
ENSG00000101447
ENSG00000183434
ENSG00000123191
ENSG00000115507
ENSG00000127527
ENSG00000114554
ENSG00000178996
ENSG00000187555
ENSG00000187764
ENSG00000198934
ENSG00000176945
ENSG00000119946
ENSG00000177272
ENSG00000135439
ENSG00000241839
ENSG00000133460
ENSG00000130244
ENSG00000141577
ENSG00000160310
ENSG00000101596
ENSG00000016602
ENSG00000101958
ENSG00000175175
ENSG00000072501
ENSG00000105976
ENSG00000131389
ENSG00000164889
ENSG00000196562
ENSG00000107099
ENSG00000172943
ENSG00000185046
ENSG00000120008
ENSG00000123338
ENSG00000132692
ENSG00000006283
ENSG00000159403
ENSG00000134452
ENSG00000141298
ENSG00000126500
ENSG00000142089
ENSG00000013503
ENSG00000185149
ENSG00000198901
ENSG00000016082
ENSG00000187527
ENSG00000179455

ENSG00000106397
ENSG00000115295
ENSG00000111335
ENSG00000129292
ENSG00000145819
ENSG00000177119
ENSG00000145244
ENSG00000106804
ENSG00000117713
ENSG00000137872
ENSG00000105568
ENSG00000123066
ENSG00000154764
ENSG00000183303
ENSG00000116128
ENSG00000035862
ENSG00000197081
ENSG00000196653
ENSG00000143119
ENSG00000062096
ENSG00000085719
ENSG00000163902
ENSG00000175471
ENSG00000010165
ENSG00000173809
ENSG00000136068
ENSG00000248919
ENSG00000186513
ENSG00000155158
ENSG00000141540
ENSG00000188827
ENSG00000171316
ENSG00000138756
ENSG00000165795
ENSG00000172260
ENSG00000104067
ENSG00000173120
ENSG00000134201
ENSG00000142511
ENSG00000126391
ENSG00000163217
ENSG00000083168
ENSG00000204634
ENSG00000135164
ENSG00000171772
ENSG00000126870
ENSG00000094661
ENSG00000162714
ENSG00000135338
ENSG00000162687
ENSG00000178395
ENSG00000198173
ENSG00000140459
ENSG00000028310
ENSG00000160447
ENSG00000110497
ENSG00000198910
ENSG00000136383
ENSG00000170579
ENSG00000183337
ENSG00000147408
ENSG00000198369
ENSG00000006071
ENSG00000179399

ENSG00000166006
ENSG00000143324
ENSG00000156886
ENSG00000053747
ENSG00000091136
ENSG00000041515
ENSG00000160321
ENSG00000170953
ENSG00000153956
ENSG00000169515
ENSG00000107815
ENSG00000135899
ENSG00000182885
ENSG00000107263
ENSG00000006453
ENSG00000204301
ENSG00000095713
ENSG00000163395
ENSG00000154783
ENSG00000077264
ENSG00000156787
ENSG00000197479
ENSG00000113492
ENSG00000178409
ENSG00000174914
ENSG00000174667
ENSG00000138771
ENSG00000161509
ENSG00000182674
ENSG00000181333
ENSG00000111879
ENSG00000184470
ENSG00000134398
ENSG00000171714
ENSG00000162946
ENSG00000162598
ENSG00000126001
ENSG00000055483
ENSG00000182199
ENSG00000101442
ENSG00000130429
ENSG00000138435
ENSG00000167634
ENSG00000104812
ENSG00000089558
ENSG00000124253
ENSG00000170484
ENSG00000143850
ENSG00000152495
ENSG00000151503
ENSG00000268173
ENSG00000147082
ENSG00000102128
ENSG00000060069
ENSG00000174231
ENSG00000029364
ENSG00000183690
ENSG00000147246
ENSG00000005007
ENSG00000124493
ENSG00000072818
ENSG00000160584
ENSG00000178038
ENSG00000117597

ENSG00000167210
ENSG00000022556
ENSG00000168434
ENSG00000114923
ENSG00000130844
ENSG00000176095
ENSG00000135074
ENSG00000131697
ENSG00000136750
ENSG00000023330
ENSG00000082014
ENSG00000099998
ENSG00000157625
ENSG00000160868
ENSG00000105996
ENSG00000169340
ENSG00000181322
ENSG00000142512
ENSG00000152953
ENSG00000105278
ENSG00000163399
ENSG00000187862
ENSG00000170523
ENSG00000181722
ENSG00000135127
ENSG00000239961
ENSG00000127529
ENSG00000188089
ENSG00000134757
ENSG00000173572
ENSG00000134516
ENSG00000150403
ENSG00000078725
ENSG00000105639
ENSG00000008086
ENSG00000074803
ENSG00000073146
ENSG00000196152
ENSG00000148942
ENSG00000145850
ENSG00000144712
ENSG00000157657
ENSG00000170921
ENSG00000143365
ENSG00000066032
ENSG00000165671
ENSG00000198912
ENSG00000206557
ENSG00000109047
ENSG00000145781
ENSG00000198033
ENSG00000115053
ENSG00000153395
ENSG00000173705
ENSG00000204176
ENSG00000143179
ENSG00000035681
ENSG00000140548
ENSG00000135625
ENSG00000182156
ENSG00000099814
ENSG00000162804
ENSG00000125386
ENSG00000154227

ENSG00000146433
ENSG00000182634
ENSG00000081913
ENSG00000012779
ENSG00000135596
ENSG00000114757
ENSG00000118432
ENSG00000127666
ENSG00000099992
ENSG00000155034
ENSG00000172061
ENSG00000061938
ENSG00000144724
ENSG00000160185
ENSG00000151789
ENSG00000052126
ENSG00000185013
ENSG00000101144
ENSG00000138792
ENSG00000008277
ENSG00000106351
ENSG00000145623
ENSG00000249853
ENSG00000103544
ENSG00000179314
ENSG00000143669
ENSG00000146453
ENSG00000168826
ENSG00000087116
ENSG00000138674
ENSG00000149177
ENSG00000163564
ENSG00000130876
ENSG00000145794
ENSG00000144647
ENSG00000157593
ENSG00000122257
ENSG00000137076
ENSG00000164309
ENSG00000132359
ENSG00000132549
ENSG00000150457
ENSG00000132481
ENSG00000134247
ENSG00000144460
ENSG00000187957
ENSG00000255408
ENSG00000054277
ENSG00000142677
ENSG00000113739
ENSG00000131196
ENSG00000185231
ENSG00000108576
ENSG00000162771
ENSG00000204701
ENSG00000198963
ENSG00000118162
ENSG00000183668
ENSG00000136002
ENSG00000166197
ENSG00000110046
ENSG00000151475
ENSG00000137460
ENSG00000179270

ENSG00000162761
ENSG00000100346
ENSG00000159167
ENSG00000173585
ENSG00000160293
ENSG00000108417
ENSG00000169418
ENSG00000140506
ENSG00000140848
ENSG00000076382
ENSG00000183908
ENSG00000174607
ENSG00000150261
ENSG00000173011
ENSG00000129422
ENSG00000182580
ENSG00000157680
ENSG00000106633
ENSG00000177731
ENSG00000135838
ENSG00000123329
ENSG00000146592
ENSG00000120251
ENSG00000139985
ENSG00000127616
ENSG00000130766
ENSG00000131374
ENSG00000103056
ENSG00000178021
ENSG00000140090
ENSG00000126218
ENSG00000170485
ENSG00000160867
ENSG00000101938
ENSG00000168502
ENSG00000111859
ENSG00000099381
ENSG00000166448
ENSG00000137497
ENSG00000160179
ENSG00000204983
ENSG00000196266
ENSG00000221836
ENSG00000164691
ENSG00000124900
ENSG00000177000
ENSG00000169071
ENSG00000124228
ENSG00000091262
ENSG00000148737
ENSG00000168447
ENSG00000141449
ENSG00000159915
ENSG00000163872
ENSG00000112773
ENSG00000140481
ENSG00000134874
ENSG00000204186
ENSG00000133943
ENSG00000125820
ENSG00000124006
ENSG00000204970
ENSG00000174255
ENSG00000187612

ENSG00000154646
ENSG00000121053
ENSG00000159363
ENSG00000159263
ENSG00000169398
ENSG00000088280
ENSG00000120156
ENSG00000163629
ENSG00000149639
ENSG00000066230
ENSG00000062038
ENSG00000088826
ENSG00000085741
ENSG00000165566
ENSG00000129566
ENSG00000135835
ENSG00000170037
ENSG00000213047
ENSG00000172350
ENSG00000157240
ENSG00000158485
ENSG00000122728
ENSG00000198948
ENSG00000106479
ENSG00000170054
ENSG00000127507
ENSG00000108797
ENSG00000138639
ENSG00000171954
ENSG00000109906
ENSG00000254245
ENSG00000122870
ENSG00000105647
ENSG00000038532
ENSG00000172365
ENSG00000151702
ENSG00000171940
ENSG00000262304
ENSG00000132773
ENSG00000144028
ENSG00000204469
ENSG00000129219
ENSG00000256463
ENSG00000101638
ENSG00000124089
ENSG00000100934
ENSG00000151150
ENSG00000133574
ENSG00000151376
ENSG00000137809
ENSG00000106246
ENSG00000132694
ENSG00000162434
ENSG00000056487
ENSG00000008838
ENSG00000204592
ENSG00000168959
ENSG00000169994
ENSG00000174483
ENSG00000112062
ENSG00000149654
ENSG00000148843
ENSG00000137802
ENSG00000158163

ENSG00000162949
ENSG00000100665
ENSG00000101438
ENSG00000174482
ENSG00000126767
ENSG00000152208
ENSG00000106789
ENSG00000108001
ENSG00000165186
ENSG00000148606
ENSG00000130158
ENSG00000198482
ENSG00000078687
ENSG00000097007
ENSG00000032444
ENSG00000163645
ENSG00000004948
ENSG00000065000
ENSG00000130827
ENSG00000137841
ENSG00000258947
ENSG00000187021
ENSG00000162374
ENSG00000132854
ENSG00000065534
ENSG00000171595
ENSG00000112902
ENSG00000178904
ENSG00000108389
ENSG00000178568
ENSG00000117009
ENSG00000087008
ENSG00000129933
ENSG00000167178
ENSG00000155660
ENSG00000009954
ENSG00000107951
ENSG00000168398
ENSG00000204632
ENSG00000102241
ENSG00000172572
ENSG00000183780
ENSG00000039560
ENSG00000204965
ENSG00000108244
ENSG00000148824
ENSG00000082805
ENSG00000107077
ENSG00000123064
ENSG00000038358
ENSG00000013364
ENSG00000133026
ENSG00000148773
ENSG00000054118
ENSG00000163995
ENSG00000148019
ENSG00000123576
ENSG00000137411
ENSG00000101546
ENSG00000130176
ENSG00000213694
ENSG00000175764
ENSG00000141458
ENSG00000158748

ENSG00000134160
ENSG00000149295
ENSG00000145147
ENSG00000102003
ENSG00000164828
ENSG00000124181
ENSG00000183715
ENSG00000151322
ENSG00000137098
ENSG00000056972
ENSG00000243135
ENSG00000169427
ENSG00000088053
ENSG00000113721
ENSG00000072858
ENSG00000115464
ENSG00000011028
ENSG00000103740
ENSG00000048342
ENSG00000135903
ENSG00000175946
ENSG00000100347
ENSG00000204542
ENSG00000214944
ENSG00000184144
ENSG00000129204
ENSG00000171608
ENSG00000182732
ENSG00000104738
ENSG00000159495
ENSG00000175398
ENSG00000165271
ENSG00000114812
ENSG00000165801
ENSG00000157766
ENSG00000115353
ENSG00000144229
ENSG00000102290
ENSG00000196338
ENSG00000088305
ENSG00000173227
ENSG00000134909
ENSG00000182134
ENSG00000169180
ENSG00000120949
ENSG00000060709
ENSG00000148840
ENSG00000130529
ENSG00000136444
ENSG00000116183
ENSG00000134569
ENSG00000104881
ENSG00000180347
ENSG00000105675
ENSG00000134365
ENSG00000167378
ENSG00000131409
ENSG00000151276
ENSG00000162849
ENSG00000074755
ENSG00000177558
ENSG00000204851
ENSG00000182013
ENSG00000196604

ENSG00000151615
ENSG00000171451
ENSG00000180815
ENSG00000158560
ENSG00000137251
ENSG00000164078
ENSG00000197943
ENSG00000184156
ENSG00000198211
ENSG00000143799
ENSG00000258417
ENSG00000170175
ENSG00000169122
ENSG00000078142
ENSG00000070601
ENSG00000204947
ENSG00000154342
ENSG00000167992
ENSG00000173262
ENSG00000087087
ENSG00000164076
ENSG00000196526
ENSG00000169851
ENSG00000105339
ENSG00000123454
ENSG00000169710
ENSG00000160218
ENSG00000183317
ENSG00000241973
ENSG00000115183
ENSG00000169126
ENSG00000158683
ENSG00000132005
ENSG00000138002
ENSG00000114487
ENSG00000197915
ENSG00000173114
ENSG00000073282
ENSG00000105464
ENSG00000144476
ENSG00000215421
ENSG00000082482
ENSG00000163701
ENSG00000183638
ENSG00000122194
ENSG00000075651
ENSG00000154678
ENSG00000149596
ENSG00000157613
ENSG00000111199
ENSG00000176927
ENSG00000187122
ENSG00000148053
ENSG00000005700
ENSG00000139641
ENSG00000143772
ENSG00000036828
ENSG00000125510
ENSG00000068400
ENSG00000118058
ENSG00000142733
ENSG00000189182
ENSG00000186529
ENSG00000127511

ENSG00000116260
ENSG00000147081
ENSG00000130377
ENSG00000135902
ENSG00000164741
ENSG00000213079
ENSG00000151338
ENSG00000122756
ENSG00000163914
ENSG00000196547
ENSG00000137868
ENSG00000164199
ENSG00000120327
ENSG00000105227
ENSG00000143473
ENSG00000062822
ENSG00000188994
ENSG00000007933
ENSG00000161681
ENSG00000153303
ENSG00000115414
ENSG00000171843
ENSG00000162745
ENSG00000146950
ENSG00000145703
ENSG00000188039
ENSG00000105663
ENSG00000133454
ENSG00000148602
ENSG00000157445
ENSG00000147459
ENSG00000154736
ENSG00000173406
ENSG00000159899
ENSG00000136160
ENSG00000132561
ENSG00000148219
ENSG00000113212
ENSG00000120054
ENSG00000089472
ENSG00000138039
ENSG00000196358
ENSG00000254521
ENSG00000141564
ENSG00000127249
ENSG00000128602
ENSG00000124486
ENSG00000143466
ENSG00000085552
ENSG00000101076
ENSG00000226372
ENSG00000130299
ENSG00000231924
ENSG00000145740
ENSG00000173546
ENSG00000157765
ENSG00000115423
ENSG00000104728
ENSG00000085511
ENSG00000160856
ENSG00000153162
ENSG00000144452
ENSG00000130226
ENSG00000182771

ENSG00000117600
ENSG00000055163
ENSG00000079432
ENSG00000204252
ENSG00000107104
ENSG00000161031
ENSG00000138162
ENSG00000173898
ENSG00000144749
ENSG00000135749
ENSG00000133863
ENSG00000135686
ENSG00000205403
ENSG00000172320
ENSG00000065361
ENSG00000171811
ENSG00000172673
ENSG00000122512
ENSG00000131386
ENSG00000162105
ENSG00000196235
ENSG00000134668
ENSG00000121440
ENSG00000102043
ENSG00000254692
ENSG00000165731
ENSG00000112293
ENSG00000166963
ENSG00000102755
ENSG00000087460
ENSG00000160145
ENSG00000173040
ENSG00000129250
ENSG00000116299
ENSG00000205250
ENSG00000075702
ENSG00000143514
ENSG00000148842
ENSG00000140015
ENSG00000010327
ENSG00000103313
ENSG00000162512
ENSG00000151474
ENSG00000120733
ENSG00000111642
ENSG00000112246
ENSG00000019169
ENSG00000140464
ENSG00000171365
ENSG00000082781
ENSG00000065618
ENSG00000132681
ENSG00000179796
ENSG00000196924
ENSG00000120594
ENSG00000171942
ENSG00000141200
ENSG00000155093
ENSG00000183580
ENSG00000183421
ENSG00000147231
ENSG00000144893
ENSG00000164171
ENSG00000197444

ENSG00000151914
ENSG00000116641
ENSG00000183486
ENSG00000135333
ENSG00000101940
ENSG00000151320
ENSG00000141956
ENSG00000081052
ENSG00000054523
ENSG00000132938
ENSG00000073614
ENSG00000158296
ENSG00000167693
ENSG00000110427
ENSG00000146005
ENSG00000128512
ENSG00000148604
ENSG00000166444
ENSG00000128159
ENSG00000188782
ENSG00000130749
ENSG00000204577
ENSG00000132793
ENSG00000100628
ENSG00000101489
ENSG00000155269
ENSG00000205517
ENSG00000142661
ENSG00000132801
ENSG00000116852
ENSG00000177599
ENSG00000124177
ENSG00000089094
ENSG00000196422
ENSG00000141384
ENSG00000126467
ENSG00000185518
ENSG00000182866
ENSG00000181804
ENSG00000149506
ENSG00000107618
ENSG00000141519
ENSG00000187098
ENSG00000197903
ENSG00000182447
ENSG00000183856
ENSG00000162706
ENSG00000099821
ENSG00000102313
ENSG00000103310
ENSG00000004939
ENSG00000013016
ENSG00000142609
ENSG00000141543
ENSG00000198795
ENSG00000204248
ENSG00000118257
ENSG00000105204
ENSG00000188785
ENSG00000197122
ENSG00000184634
ENSG00000081248
ENSG00000174279
ENSG00000136943

ENSG00000131323
ENSG00000057657
ENSG00000117707
ENSG00000102385
ENSG00000134802
ENSG00000144857
ENSG00000125337
ENSG00000113231
ENSG00000177324
ENSG00000154734
ENSG00000171219
ENSG00000090615
ENSG00000176049
ENSG00000127334
ENSG00000146966
ENSG00000198598
ENSG00000244038
ENSG00000066248
ENSG00000004846
ENSG00000164880
ENSG00000162951
ENSG00000197106
ENSG00000038382
ENSG00000004975
ENSG00000171759
ENSG00000204613
ENSG00000146221
ENSG00000269323
ENSG00000166948
ENSG00000113555
ENSG00000088836
ENSG00000133958
ENSG00000176769
ENSG00000166748
ENSG00000184445
ENSG00000166206
ENSG00000136378
ENSG00000104998
ENSG00000146828
ENSG00000204406
ENSG00000171551
ENSG00000160007
ENSG00000126838
ENSG00000149311
ENSG00000104043
ENSG00000050555
ENSG00000141337
ENSG00000184408
ENSG00000091128
ENSG00000182319
ENSG00000104369
ENSG00000170477
ENSG00000163885
ENSG00000066056
ENSG00000198633
ENSG00000204969
ENSG00000009709
ENSG00000143375
ENSG00000196914
ENSG00000089225
ENSG00000171992
ENSG00000110400
ENSG00000169676
ENSG00000178586

ENSG00000130147
ENSG00000107863
ENSG00000166897
ENSG00000253117
ENSG00000021826
ENSG00000099991
ENSG00000182667
ENSG00000069248
ENSG00000143126
ENSG00000186723
ENSG00000143028
ENSG00000163492
ENSG00000140323
ENSG00000144655
ENSG00000159450
ENSG00000116141
ENSG00000133488
ENSG00000160716
ENSG00000125965
ENSG00000130477
ENSG00000162733
ENSG00000181693
ENSG00000079999
ENSG00000068724
ENSG00000124067
ENSG00000113319
ENSG00000163637
ENSG00000068793
ENSG00000118777
ENSG00000177380
ENSG00000177614
ENSG00000104237
ENSG00000189091
ENSG00000138031
ENSG00000173567
ENSG00000178235
ENSG00000198670
ENSG00000070748
ENSG00000124496
ENSG00000147130
ENSG00000084676
ENSG00000121270
ENSG00000213923
ENSG00000140538
ENSG00000082397
ENSG00000141946
ENSG00000105509
ENSG00000132394
ENSG00000072201
ENSG00000196535
ENSG00000134013
ENSG00000186918
ENSG00000163053
ENSG00000111262
ENSG00000126217
ENSG00000122778
ENSG00000175029
ENSG00000130052
ENSG00000127481
ENSG00000124571
ENSG00000026559
ENSG00000095059
ENSG00000163817
ENSG00000168575

ENSG00000171860
ENSG00000197093
ENSG00000060718
ENSG00000140798
ENSG00000165966
ENSG00000104447
ENSG00000134115
ENSG00000116544
ENSG00000112769
ENSG00000100926
ENSG00000146250
ENSG00000136026
ENSG00000078114
ENSG00000114480
ENSG00000143199
ENSG00000179002
ENSG00000050438
ENSG00000172995
ENSG00000106665
ENSG00000044524
ENSG00000047644
ENSG00000038295
ENSG00000069431
ENSG00000081760
ENSG00000182752
ENSG00000171444
ENSG00000116783
ENSG00000132932
ENSG00000158445
ENSG00000042980
ENSG00000091536
ENSG00000253731
ENSG00000197724
ENSG00000054356
ENSG00000171560
ENSG00000039537
ENSG00000165185
ENSG00000117724
ENSG00000132688
ENSG00000083457
ENSG00000157426
ENSG00000140505
ENSG00000176225
ENSG00000080854
ENSG00000101306
ENSG00000060656
ENSG00000100985
ENSG00000178732
ENSG00000139182
ENSG00000196963
ENSG00000185070
ENSG00000091592
ENSG00000105695
ENSG00000142949
ENSG00000251664
ENSG00000072832
ENSG00000074276
ENSG00000122145
ENSG00000132915
ENSG00000176771
ENSG00000171044
ENSG00000142611
ENSG00000108175
ENSG00000144406

ENSG00000149294
ENSG00000140521
ENSG00000072786
ENSG00000152785
ENSG00000187048
ENSG00000127580
ENSG00000253846
ENSG00000108821
ENSG00000177992
ENSG00000145216
ENSG00000160299
ENSG00000133020
ENSG00000164694
ENSG00000130589
ENSG00000081842
ENSG00000179222
ENSG00000156709
ENSG00000166839
ENSG00000112659
ENSG00000155066
ENSG00000126583
ENSG00000111961
ENSG00000109066
ENSG00000148513
ENSG00000175267
ENSG00000111087
ENSG00000198793
ENSG00000127412
ENSG00000119522
ENSG00000068650
ENSG00000158089
ENSG00000147010
ENSG00000136715
ENSG00000163531
ENSG00000160505
ENSG00000057149
ENSG00000138759
ENSG00000112038
ENSG00000184571
ENSG00000159164
ENSG00000181790
ENSG00000184185
ENSG00000154237
ENSG00000048052
ENSG00000125755
ENSG00000157388
ENSG00000111452
ENSG00000121634
ENSG00000198722
ENSG00000143127
ENSG00000077782
ENSG00000072195
ENSG00000111046
ENSG00000156414
ENSG00000156920
ENSG00000100280
ENSG00000152591
ENSG00000074370
ENSG00000138161
ENSG00000167972
ENSG00000048707
ENSG00000155966
ENSG00000113361
ENSG00000259030

ENSG00000172046
ENSG00000134207
ENSG00000163092
ENSG00000075035
ENSG00000186635
ENSG00000130943
ENSG00000074211
ENSG00000039139
ENSG00000243156
ENSG00000171105
ENSG00000077943
ENSG00000153071
ENSG00000122515
ENSG00000143322
ENSG00000168769
ENSG00000162104
ENSG00000030582
ENSG00000121933
ENSG00000152977
ENSG00000189350
ENSG00000147044
ENSG00000099139
ENSG00000239389
ENSG00000198788
ENSG00000101825
ENSG00000169618
ENSG00000130287
ENSG00000163485
ENSG00000168843
ENSG00000101191
ENSG00000168280
ENSG00000109501
ENSG00000136928
ENSG00000130396
ENSG00000206579
ENSG00000120907
ENSG00000166342
ENSG00000129646
ENSG00000109944
ENSG00000173801
ENSG00000160469
ENSG00000070915
ENSG00000120328
ENSG00000137877
ENSG00000142347
ENSG00000099954
ENSG00000110514
ENSG00000184647
ENSG00000105426
ENSG00000147724
ENSG00000133424
ENSG00000070886
ENSG00000184922
ENSG00000172349
ENSG00000118515
ENSG00000079112
ENSG00000172403
ENSG00000002726
ENSG00000170382
ENSG00000163611
ENSG00000073331
ENSG00000166535
ENSG00000153253
ENSG00000125851

ENSG00000158321
ENSG00000090975
ENSG00000206181
ENSG00000184305
ENSG00000087245
ENSG00000244482
ENSG00000112992
ENSG00000101040
ENSG00000101349
ENSG00000167676
ENSG00000166833
ENSG00000175356
ENSG00000154133
ENSG00000135424
ENSG00000171189
ENSG00000124664
ENSG00000013293
ENSG00000074181
ENSG00000178394
ENSG00000149182
ENSG00000160796
ENSG00000130940
ENSG00000131873
ENSG00000118004
ENSG00000135945
ENSG00000164588
ENSG00000171456
ENSG00000167037
ENSG00000129351
ENSG00000107282
ENSG00000165379
ENSG00000162267
ENSG00000142185
ENSG00000141968
ENSG00000146426
ENSG00000156113
ENSG00000076555
ENSG00000171735
ENSG00000071242
ENSG00000165194
ENSG00000142405
ENSG00000145536
ENSG00000114805
ENSG00000089154
ENSG00000012124
ENSG00000155052
ENSG00000187391
ENSG00000170011
ENSG00000218336
ENSG00000122641
ENSG00000221866
ENSG00000151208
ENSG00000148143
ENSG00000165699
ENSG00000153292
ENSG00000156395
ENSG00000162337
ENSG00000077522
ENSG00000134376
ENSG00000140443
ENSG00000204131
ENSG00000107593
ENSG00000133216
ENSG00000089101

ENSG00000105877
ENSG00000140836
ENSG00000078018
ENSG00000134250
ENSG00000149403
ENSG00000124788
ENSG00000132164
ENSG00000084774
ENSG00000148848
ENSG00000161270
ENSG00000047617
ENSG00000124140
ENSG00000113211
ENSG00000170004
ENSG00000115850
ENSG00000197694
ENSG00000253729
ENSG00000203786
ENSG00000178031
ENSG00000118407
ENSG00000250120
ENSG00000162670
ENSG00000105287
ENSG00000112852
ENSG00000176463
ENSG00000198842
ENSG00000198026
ENSG00000198286
ENSG00000096433
ENSG00000080573
ENSG00000078814
ENSG00000074657
ENSG00000166689
ENSG00000148468
ENSG00000185313
ENSG00000198879
ENSG00000197616
ENSG00000181192
ENSG00000168356
ENSG00000129159
ENSG00000130037
ENSG00000109667
ENSG00000177084
ENSG00000264424
ENSG00000157856
ENSG00000160255
ENSG00000005339
ENSG00000141527
ENSG00000103222
ENSG00000114841
ENSG00000173064
ENSG00000079841
ENSG00000055609
ENSG00000167548
ENSG00000144481
ENSG00000085185
ENSG00000047936
ENSG00000135636
ENSG00000171495
ENSG00000172716
ENSG00000179593
ENSG00000107554
ENSG00000168016
ENSG00000132874

ENSG00000197893
ENSG00000083067
ENSG00000087495
ENSG00000122786
ENSG00000197386
ENSG00000156510
ENSG00000142449
ENSG00000144191
ENSG00000182095
ENSG00000196296
ENSG00000156564
ENSG00000153976
ENSG00000138119
ENSG00000079102
ENSG00000136717
ENSG00000138741
ENSG00000146938
ENSG00000148408
ENSG00000186174
ENSG00000163673
ENSG00000130779
ENSG00000148400
ENSG00000166147
ENSG00000181045
ENSG00000163618
ENSG00000185585
ENSG00000064687
ENSG00000179709
ENSG00000182463
ENSG00000131620
ENSG00000140853
ENSG00000140937
ENSG00000065526
ENSG00000204961
ENSG00000160111
ENSG00000119866
ENSG00000185823
ENSG00000105409
ENSG00000113205
ENSG00000007062
ENSG00000142973
ENSG00000171094
ENSG00000205038
ENSG00000171815
ENSG00000171435
ENSG00000113100
ENSG00000160460
ENSG00000160791
ENSG00000156299
ENSG00000248383
ENSG00000185448
ENSG00000134853
ENSG00000141837
ENSG00000178104
ENSG00000144908
ENSG00000169282
ENSG00000126562
ENSG00000107736
ENSG00000036448
ENSG00000175065
ENSG00000132382
ENSG00000049323
ENSG00000070729
ENSG00000138650

ENSG00000106290
ENSG00000198796
ENSG00000179869
ENSG00000171487
ENSG00000106976
ENSG00000138411
ENSG00000153820
ENSG00000184304
ENSG00000165029
ENSG00000116962
ENSG00000157404
ENSG00000088538
ENSG00000102001
ENSG00000150995
ENSG00000184347
ENSG00000170549
ENSG00000145362
ENSG00000101542
ENSG00000105143
ENSG00000149292
ENSG00000050030
ENSG00000068654
ENSG00000152217
ENSG00000088367
ENSG00000117245
ENSG00000147485
ENSG00000128052
ENSG00000084636
ENSG00000100678
ENSG00000120324
ENSG00000139364
ENSG00000151655
ENSG00000185274
ENSG00000075275
ENSG00000012232
ENSG00000072952
ENSG00000172554
ENSG00000198354
ENSG00000163219
ENSG00000069188
ENSG00000151240
ENSG00000133401
ENSG00000138622
ENSG00000164692
ENSG00000109265
ENSG00000198947
ENSG00000181378
ENSG00000156650
ENSG00000171587
ENSG00000134369
ENSG00000257335
ENSG00000137727
ENSG00000165300
ENSG00000178971
ENSG00000169083
ENSG00000187535
ENSG00000196498
ENSG00000187372
ENSG00000031081
ENSG00000069018
ENSG00000179242
ENSG00000104774
ENSG00000169436
ENSG00000214814

ENSG00000189132
ENSG00000047578
ENSG00000127603
ENSG00000145555
ENSG00000137216
ENSG00000079308
ENSG00000102935
ENSG00000155511
ENSG00000144285
ENSG00000116254
ENSG00000118689
ENSG00000150086
ENSG00000007314
ENSG00000125730
ENSG00000110076
ENSG00000165757
ENSG00000067798
ENSG00000124942
ENSG00000124126
ENSG00000196367
ENSG00000177103
ENSG00000008226
ENSG00000067842
ENSG00000075340
ENSG00000157087
ENSG00000177354
ENSG00000105851
ENSG00000127241
ENSG00000113248
ENSG00000158125
ENSG00000156218
ENSG00000002746
ENSG00000204956
ENSG00000010818
ENSG00000133392
ENSG00000102452
ENSG00000020256
ENSG00000198198
ENSG00000081818
ENSG00000113327
ENSG00000169933
ENSG00000150394
ENSG00000196876
ENSG00000055957
ENSG00000118898
ENSG00000166501
ENSG00000138615
ENSG00000125414
ENSG00000029534
ENSG00000164061
ENSG00000198959
ENSG00000081479
ENSG00000167880
ENSG00000163873
ENSG00000085563
ENSG00000187775
ENSG00000133124
ENSG00000155816
ENSG00000146648
ENSG00000106571
ENSG00000116147
ENSG00000081853
ENSG00000157927
ENSG00000196730

ENSG00000206384
ENSG00000196569
ENSG00000183495
ENSG00000184956
ENSG00000196090
ENSG00000103449
ENSG00000120332
ENSG00000075673
ENSG00000214338
ENSG00000100095
ENSG00000105429
ENSG00000115306
ENSG00000151388
ENSG00000173821
ENSG00000183454
ENSG00000168477
ENSG00000186340
ENSG00000206190
ENSG00000140873
ENSG00000089250
ENSG00000255330
ENSG00000162711
ENSG00000154654
ENSG00000074047
ENSG00000058335
ENSG00000183914
ENSG00000114270
ENSG00000124203
ENSG00000136531
ENSG00000164796
ENSG00000128731
ENSG00000150893
ENSG00000152822
ENSG00000070182
ENSG00000130508
ENSG00000023839
ENSG00000121297
ENSG00000054654
ENSG00000170927
ENSG00000187955
ENSG00000113262
ENSG00000141510
ENSG00000185920
ENSG00000103197
ENSG00000198691
ENSG00000109927
ENSG00000204291
ENSG00000185567
ENSG00000150760
ENSG00000100345
ENSG00000171914
ENSG00000143341
ENSG00000174469
ENSG00000078295
ENSG00000118946
ENSG00000136011
ENSG00000008300
ENSG00000198597
ENSG00000158486
ENSG00000152092
ENSG00000041982
ENSG00000083857
ENSG00000167522
ENSG00000198010

ENSG00000123384
ENSG00000143631
ENSG00000196208
ENSG00000168702
ENSG00000169876
ENSG00000151067
ENSG00000115705
ENSG00000183873
ENSG00000123243
ENSG00000138829
ENSG00000021645
ENSG00000006788
ENSG00000169862
ENSG00000130635
ENSG00000092054
ENSG00000107611
ENSG00000196159
ENSG00000142798
ENSG00000145113
ENSG00000165323
ENSG00000149256
ENSG00000146555
ENSG00000131711
ENSG00000198626
ENSG00000091656
ENSG00000179915
ENSG00000196218
ENSG00000110799
ENSG00000198216
ENSG00000145934
ENSG00000197410
ENSG00000042832
ENSG00000115155
ENSG00000157423
ENSG00000151952
ENSG00000131018
ENSG00000197653
ENSG00000117983
ENSG00000084674
ENSG00000101680
ENSG00000163359
ENSG00000154358
ENSG00000198838
ENSG00000007174
ENSG00000090920
ENSG00000121904
ENSG00000042781
ENSG00000183091
ENSG00000183117
ENSG00000181143
ENSG00000155657
