# Supplementary material for: Prevalent Accumulation of Non-Optimal Codons through Somatic Mutations in Human Cancers
Source: PLoS One. 2016 Aug 11;11(8):e0160463. doi: 10.1371/journal.pone.0160463 (PMC4981346; doi:10.1371/journal.pone.0160463)
Supplement: S12 Table — (PDF) [file pone.0160463.s014.pdf]

Proto-oncogenes	Tumor-repressors
ENSG00000182511	ENSG00000170632
ENSG00000168283	ENSG00000008710
ENSG00000141968	ENSG00000213341
ENSG00000126785	ENSG00000145022
ENSG00000145022	ENSG00000168646
ENSG00000101336	ENSG00000186153
ENSG00000186051	ENSG00000115266
ENSG00000041353	ENSG00000206075
ENSG00000143545	ENSG00000172531
ENSG00000129472	ENSG00000171735
ENSG00000077092	ENSG00000071242
ENSG00000184481	ENSG00000077092
ENSG00000171791	ENSG00000142867
ENSG00000165023	ENSG00000102054
ENSG00000157764	ENSG00000112096
ENSG00000111276	ENSG00000108518
ENSG00000178691	ENSG00000184481
ENSG00000167964	ENSG00000129071
ENSG00000138175	ENSG00000183814
ENSG00000119950	ENSG00000179361
ENSG00000166592	ENSG00000239306
ENSG00000167601	ENSG00000137142
ENSG00000167085	ENSG00000104723
ENSG00000111241	ENSG00000005955
ENSG00000170962	ENSG00000164825
ENSG00000168118	ENSG00000125841
ENSG00000182986	ENSG00000138193
ENSG00000174903	ENSG00000119950
ENSG00000170776	ENSG00000182871
ENSG00000184992	ENSG00000221829
ENSG00000121879	ENSG00000167085
ENSG00000197880	ENSG00000020633
ENSG00000170345	ENSG00000014123
ENSG00000167461	ENSG00000111344
ENSG00000110367	ENSG00000078900
ENSG00000073921	ENSG00000105856
ENSG00000071564	ENSG00000040199
ENSG00000157554	ENSG00000101955
ENSG00000141985	ENSG00000091129
ENSG00000101246	ENSG00000004838
ENSG00000123892	ENSG00000146674
ENSG00000075388	ENSG00000141753
ENSG00000169213	ENSG00000055130
ENSG00000171094	ENSG00000119383
ENSG00000101977	ENSG00000115461
ENSG00000104388	ENSG00000185338
ENSG00000122644	ENSG00000120738
ENSG00000072364	ENSG00000139263
ENSG00000050426	ENSG00000073282

ENSG00000154229	ENSG00000100387
ENSG00000182712	ENSG00000115317
ENSG00000087586	ENSG00000179271
ENSG00000142208	ENSG00000136040
ENSG00000186895	ENSG00000171444
ENSG00000007237	ENSG00000105928
ENSG00000143622	ENSG00000064393
ENSG00000102096	ENSG00000136653
ENSG00000118058	ENSG00000136114
ENSG00000167550	ENSG00000103264
ENSG00000072422	ENSG00000132932
ENSG00000079332	ENSG00000133895
ENSG00000108379	ENSG00000141252
ENSG00000104415	ENSG00000048828
ENSG00000123570	ENSG00000139278
ENSG00000136997	ENSG00000111269
ENSG00000134853	ENSG00000107551
ENSG00000085276	ENSG00000090924
ENSG00000118689	ENSG00000103479
ENSG00000077150	ENSG00000187054
ENSG00000126752	ENSG00000137707
ENSG00000080371	ENSG00000163932
ENSG00000222014	ENSG00000182636
ENSG00000198355	ENSG00000168556
ENSG00000214827	ENSG00000138294
ENSG00000171843	ENSG00000103126
ENSG00000119508	ENSG00000112679
ENSG00000134323	ENSG00000123080
ENSG00000050327	ENSG00000079385
ENSG00000237441	ENSG00000114021
ENSG00000166452	ENSG00000165699
ENSG00000198400	ENSG00000112761
ENSG00000108292	ENSG00000182511
ENSG00000130368	ENSG00000154493
ENSG00000176490	ENSG00000120885
ENSG00000124209	ENSG00000079257
ENSG00000100228	ENSG00000123095
ENSG00000206418	ENSG00000147883
ENSG00000100302	ENSG00000090238
ENSG00000108830	ENSG00000245848
ENSG00000139725	ENSG00000164741
ENSG00000181467	ENSG00000150093
ENSG00000144118	ENSG00000104689
ENSG00000105976	ENSG00000110002
ENSG00000143761	ENSG00000139618
ENSG00000070404	ENSG00000154096
ENSG00000172794	ENSG00000116254
ENSG00000179331	ENSG00000152894
ENSG00000120008	ENSG00000118689
ENSG00000112210	ENSG00000118503
ENSG00000111404	ENSG00000108231

ENSG00000165702	ENSG00000176485
ENSG00000165527	ENSG00000056661
ENSG00000158186	ENSG00000163939
ENSG00000110619	ENSG00000105722
ENSG00000118508	ENSG00000158747
ENSG00000118513	ENSG00000126581
ENSG00000107807	ENSG00000182168
ENSG00000167513	ENSG00000101412
ENSG00000066336	ENSG00000110171
ENSG00000145819	ENSG00000109452
ENSG00000085832	ENSG00000243477
ENSG00000105656	ENSG00000119508
ENSG00000103710	ENSG00000150593
ENSG00000116574	ENSG00000197635
ENSG00000107968	ENSG00000133454
ENSG00000143862	ENSG00000026297
ENSG00000018408	ENSG00000140945
ENSG00000105619	ENSG00000102974
ENSG00000116128	ENSG00000176490
ENSG00000111737	ENSG00000104814
ENSG00000067560	ENSG00000100003
ENSG00000144134	ENSG00000145632
ENSG00000144840	ENSG00000133116
ENSG00000137693	ENSG00000134531
ENSG00000169228	ENSG00000162892
ENSG00000197885	ENSG00000156804
ENSG00000123473	ENSG00000152661
ENSG00000141150	ENSG00000184281
ENSG00000162924	ENSG00000131016
ENSG00000068323	ENSG00000133216
ENSG00000113595	ENSG00000124762
ENSG00000137193	ENSG00000135439
ENSG00000083168	ENSG00000109320
ENSG00000100311	ENSG00000146094
ENSG00000172660	ENSG00000083093
ENSG00000250254	ENSG00000100290
ENSG00000089280	ENSG00000130254
ENSG00000172680	ENSG00000120910
ENSG00000168461	ENSG00000182197
ENSG00000172007	ENSG00000164938
ENSG00000164292	ENSG00000064933
ENSG00000133703	ENSG00000159840
ENSG00000152700	ENSG00000161048
ENSG00000165731	ENSG00000140836
ENSG00000146955	ENSG00000170498
ENSG00000128340	ENSG00000143761
ENSG00000128581	ENSG00000170367
ENSG00000106615	ENSG00000166091
ENSG00000138293	ENSG00000120008
ENSG00000087460	ENSG00000013588
ENSG00000176105	ENSG00000129993

ENSG00000185236	ENSG00000166619
ENSG00000163655	ENSG00000128602
ENSG00000010810	ENSG00000107954
ENSG00000105649	ENSG00000133134
ENSG00000124243	ENSG00000170296
ENSG00000006744	ENSG00000104332
ENSG00000166128	ENSG00000100296
ENSG00000100721	ENSG00000183765
ENSG00000111087	ENSG00000172201
ENSG00000103769	ENSG00000035403
ENSG00000213465	ENSG00000063438
ENSG00000132341	ENSG00000132382
ENSG00000162595	ENSG00000104213
ENSG00000110395	ENSG00000117713
ENSG00000132881	ENSG00000113083
ENSG00000099246	ENSG00000133328
ENSG00000069399	ENSG00000165474
ENSG00000157404	ENSG00000171056
ENSG00000104903	ENSG00000179456
ENSG00000133818	ENSG00000117425
ENSG00000204713	ENSG00000175315
ENSG00000108774	ENSG00000197081
ENSG00000140464	ENSG00000122877
ENSG00000143549	ENSG00000104419
ENSG00000124795	ENSG00000095002
ENSG00000151422	ENSG00000019505
ENSG00000102128	ENSG00000067560
ENSG00000124839	ENSG00000107104
ENSG00000139083	ENSG00000151348
ENSG00000172602	ENSG00000111653
ENSG00000213281	ENSG00000138650
ENSG00000214087	ENSG00000134508
ENSG00000004059	ENSG00000172530
ENSG00000168421	ENSG00000125798
ENSG00000128487	ENSG00000137693
ENSG00000100105	ENSG00000147889
ENSG00000140983	ENSG00000196730
ENSG00000135363	ENSG00000109854
ENSG00000066468	ENSG00000121753
ENSG00000155961	ENSG00000165795
ENSG00000147548	ENSG00000097033
ENSG00000139687	ENSG00000083857
ENSG00000134594	ENSG00000007306
ENSG00000164949	ENSG00000107937
ENSG00000078061	ENSG00000141150
ENSG00000185515	ENSG00000068323
ENSG00000157933	ENSG00000175793
ENSG00000006468	ENSG00000163217
ENSG00000115963	ENSG00000105971
ENSG00000175906	ENSG00000107984
ENSG00000105514	ENSG00000178175

ENSG00000128512	ENSG00000054598
ENSG00000165671	ENSG00000135164
ENSG00000179295	ENSG00000007372
ENSG00000143294	ENSG00000166200
ENSG00000167578	ENSG00000184937
ENSG00000169696	ENSG00000023287
ENSG00000182944	ENSG00000140968
ENSG00000175197	ENSG00000122512
ENSG00000104140	ENSG00000127947
ENSG00000162980	ENSG00000144677
ENSG00000196588	ENSG00000166033
ENSG00000067955	ENSG00000104765
ENSG00000150907	ENSG00000138376
ENSG00000162367	ENSG00000083799
ENSG00000175414	ENSG00000099810
ENSG00000123728	ENSG00000114388
ENSG00000182866	ENSG00000126456
ENSG00000187097	ENSG00000036257
ENSG00000126858	ENSG00000186532
ENSG00000182578	ENSG00000156076
ENSG00000167193	ENSG00000174469
ENSG00000173821	ENSG00000123094
ENSG00000108924	ENSG00000108387
ENSG00000189067	ENSG00000197299
ENSG00000154917	ENSG00000137203
ENSG00000134287	ENSG00000138293
ENSG00000069974	ENSG00000072609
ENSG00000153814	ENSG00000129521
ENSG00000100030	ENSG00000135249
ENSG00000141376	ENSG00000185024
ENSG00000138674	ENSG00000111961
ENSG00000159216	ENSG00000126561
ENSG00000174225	ENSG00000198561
ENSG00000198795	ENSG00000215301
ENSG00000172476	ENSG00000085382
ENSG00000149948	ENSG00000112530
ENSG00000197122	ENSG00000068028
ENSG00000133392	ENSG00000177374
ENSG00000155366	ENSG00000161040
ENSG00000008853	ENSG00000175387
ENSG00000102882	ENSG00000163191
ENSG00000132698	ENSG00000023902
ENSG00000254087	ENSG00000143514
ENSG00000168256	ENSG00000105329
ENSG00000134533	ENSG00000154655
ENSG00000178573	ENSG00000137218
ENSG00000070831	ENSG00000131910
ENSG00000121454	ENSG00000135956
ENSG00000164611	ENSG00000154277
ENSG00000163558	ENSG00000114204
ENSG00000133639	ENSG00000162595

ENSG00000047410	ENSG00000105767
ENSG00000117280	ENSG00000135828
ENSG00000000938	ENSG00000108384
ENSG00000172780	ENSG00000080839
ENSG00000141380	ENSG00000120733
ENSG00000131759	ENSG00000186908
ENSG00000084733	ENSG00000120708
ENSG00000078674	ENSG00000107882
ENSG00000119729	ENSG00000105373
ENSG00000047936	ENSG00000100234
ENSG00000188042	ENSG00000161298
ENSG00000134108	ENSG00000166573
ENSG00000127314	ENSG00000167797
ENSG00000168374	ENSG00000173531
ENSG00000152213	ENSG00000140464
ENSG00000172493	ENSG00000187091
ENSG00000139832	ENSG00000179796
ENSG00000198900	ENSG00000122133
ENSG00000102034	ENSG00000117118
ENSG00000166407	ENSG00000139083
ENSG00000144566	ENSG00000111328
ENSG00000130396	ENSG00000133321
ENSG00000105221	ENSG00000232119
ENSG00000171476	ENSG00000083642
ENSG00000109471	ENSG00000134086
ENSG00000143878	ENSG00000042493
ENSG00000122035	ENSG00000112406
ENSG00000213672	ENSG00000185499
ENSG00000181163	ENSG00000102554
ENSG00000125249	ENSG00000169031
ENSG00000114316	ENSG00000178773
ENSG00000152932	ENSG00000072133
ENSG00000205927	ENSG00000131435
ENSG00000139998	ENSG00000103034
ENSG00000119537	ENSG00000076242
ENSG00000169379	ENSG00000178726
ENSG00000122025	ENSG00000114978
ENSG00000141542	ENSG00000115107
ENSG00000009709	ENSG00000012223
ENSG00000109906	ENSG00000110851
ENSG00000138675	ENSG00000147548
ENSG00000119396	ENSG00000123358
ENSG00000114354	ENSG00000008226
ENSG00000123595	ENSG00000106331
ENSG00000196914	ENSG00000169856
ENSG00000151702	ENSG00000184349
ENSG00000187682	ENSG00000118495
ENSG00000120805	ENSG00000182253
ENSG00000126883	ENSG00000147316
ENSG00000116473	ENSG00000170549
ENSG00000145715	ENSG00000171720

ENSG00000162775	ENSG00000067141
ENSG00000130382	ENSG00000197919
ENSG00000111540	ENSG00000139687
ENSG00000113916	ENSG00000180953
ENSG00000075785	ENSG00000065559
ENSG00000135679	ENSG00000163590
ENSG00000060140	ENSG00000196159
ENSG00000157557	ENSG00000050165
ENSG00000175582	ENSG00000138443
ENSG00000078399	ENSG00000054523
ENSG00000097007	ENSG00000115963
ENSG00000153179	ENSG00000144791
ENSG00000153208	ENSG00000188257
ENSG00000110777	ENSG00000170873
ENSG00000174775	ENSG00000239672
ENSG00000188060	ENSG00000148481
ENSG00000163660	ENSG00000112237
ENSG00000134954	ENSG00000073150
ENSG00000100276	ENSG00000138413
ENSG00000137502	ENSG00000168389
ENSG00000079102	ENSG00000117906
ENSG00000177105	ENSG00000101265
ENSG00000126458	ENSG00000166444
ENSG00000128045	ENSG00000061337
ENSG00000196092	ENSG00000171862
ENSG00000084676	ENSG00000243725
ENSG00000186716	ENSG00000160633
ENSG00000157869	ENSG00000149554
ENSG00000108091	ENSG00000256870
ENSG00000078403	ENSG00000099953
ENSG00000113721	ENSG00000155090
ENSG00000187908	ENSG00000196628
ENSG00000165997	ENSG00000162616
ENSG00000126860	ENSG00000119147
ENSG00000135903	ENSG00000115306
ENSG00000043093	ENSG00000116285
ENSG00000166140	ENSG00000099956
ENSG00000213231	ENSG00000081913
ENSG00000129204	ENSG00000131023
ENSG00000185305	ENSG00000158008
ENSG00000172927	ENSG00000147324
ENSG00000126217	ENSG00000150907
ENSG00000185862	ENSG00000139842
ENSG00000006451	ENSG00000141510
ENSG00000109113	ENSG00000161055
ENSG00000181690	ENSG00000134982
ENSG00000177606	ENSG00000146904
ENSG00000152214	ENSG00000176887
ENSG00000165105	ENSG00000163638
ENSG00000185630	ENSG00000100033
ENSG00000096968	ENSG00000109670

ENSG00000048462	ENSG00000189067
ENSG00000132155	ENSG00000129355
ENSG00000147127	ENSG00000105825
ENSG00000109685	ENSG00000156531
ENSG00000139890	ENSG00000156508
ENSG00000182158	ENSG00000155666
ENSG00000173848	ENSG00000122779
	ENSG00000127831
	ENSG00000162706
	ENSG00000142178
	ENSG00000142082
	ENSG00000175354
	ENSG00000184675
	ENSG00000196754
	ENSG00000137801
	ENSG00000149177
	ENSG00000163564
	ENSG00000185565
	ENSG00000114127
	ENSG00000164120
	ENSG00000168040
	ENSG00000150457
	ENSG00000153071
	ENSG00000085662
	ENSG00000005339
	ENSG00000137713
	ENSG00000167755
	ENSG00000008853
	ENSG00000185920
	ENSG00000125144
	ENSG00000068903
	ENSG00000115325
	ENSG00000131080
	ENSG00000006712
	ENSG00000149679
	ENSG00000032742
	ENSG00000057657
	ENSG00000189283
	ENSG00000110711
	ENSG00000122707
	ENSG00000132698
	ENSG00000168769
	ENSG00000104413
	ENSG00000178573
	ENSG00000174744
	ENSG00000108094
	ENSG00000184557
	ENSG00000120370
	ENSG00000152977
	ENSG00000132196
	ENSG00000163558

ENSG00000004534
ENSG00000071794
ENSG00000168702
ENSG00000137709
ENSG00000170801
ENSG00000149782
ENSG00000185345
ENSG00000129422
ENSG00000104899
ENSG00000074319
ENSG00000161647
ENSG00000196470
ENSG00000162694
ENSG00000110628
ENSG00000130939
ENSG00000163930
ENSG00000144730
ENSG00000213859
ENSG00000105974
ENSG00000127616
ENSG00000204370
ENSG00000125845
ENSG00000067082
ENSG00000120868
ENSG00000012232
ENSG00000071243
ENSG00000152213
ENSG00000120889
ENSG00000204977
ENSG00000140873
ENSG00000072952
ENSG00000168395
ENSG00000148737
ENSG00000007923
ENSG00000101191
ENSG00000177425
ENSG00000186575
ENSG00000117289
ENSG00000102786
ENSG00000198553
ENSG00000004866
ENSG00000102804
ENSG00000173077
ENSG00000001631
ENSG00000128596
ENSG00000171476
ENSG00000114251
ENSG00000159692
ENSG00000143878
ENSG00000122035
ENSG00000160007

ENSG00000189079
ENSG00000136826
ENSG00000165025
ENSG00000167642
ENSG00000109062
ENSG00000075711
ENSG00000173801
ENSG00000124802
ENSG00000139174
ENSG00000156970
ENSG00000114861
ENSG00000149311
ENSG00000085741
ENSG00000196712
ENSG00000135269
ENSG00000146414
ENSG00000007168
ENSG00000055332
ENSG00000121769
ENSG00000106852
ENSG00000163513
ENSG00000173812
ENSG00000143570
ENSG00000067066
ENSG00000109906
ENSG00000182985
ENSG00000198300
ENSG00000169184
ENSG00000125347
ENSG00000091138
ENSG00000196914
ENSG00000012048
ENSG00000089225
ENSG00000138448
ENSG00000135298
ENSG00000167657
ENSG00000147257
ENSG00000136848
ENSG00000151090
ENSG00000135482
ENSG00000003756
ENSG00000064201
ENSG00000116473
ENSG00000139318
ENSG00000091483
ENSG00000129451
ENSG00000002822
ENSG00000170955
ENSG00000135046
ENSG00000087088
ENSG00000147854

ENSG00000107731
ENSG00000134371
ENSG00000253958
ENSG00000064300
ENSG00000186654
ENSG00000080503
ENSG00000120057
ENSG00000163737
ENSG00000067113
ENSG00000147443
ENSG00000140350
ENSG00000139352
ENSG00000039068
ENSG00000153487
ENSG00000161544
ENSG00000137962
ENSG00000166164
ENSG00000178568
ENSG00000104228
ENSG00000132781
ENSG00000113763
ENSG00000166266
ENSG00000076864
ENSG00000124713
ENSG00000134954
ENSG00000069702
ENSG00000100276
ENSG00000108963
ENSG00000103490
ENSG00000108946
ENSG00000137727
ENSG00000196092
ENSG00000186716
ENSG00000085117
ENSG00000184674
ENSG00000136717
ENSG00000145147
ENSG00000140009
ENSG00000106536
ENSG00000183715
ENSG00000116731
ENSG00000135424
ENSG00000082397
ENSG00000144339
ENSG00000139437
ENSG00000187323
ENSG00000187908
ENSG00000148400
ENSG00000100380
ENSG00000128918
ENSG00000172005

ENSG00000064012
ENSG00000153707
ENSG00000241697
ENSG00000159388
ENSG00000163453
ENSG00000135766
ENSG00000175054
ENSG00000154803
ENSG00000159023
ENSG00000103197
ENSG00000118523
ENSG00000100941
ENSG00000104695
ENSG00000167034
ENSG00000184828
ENSG00000118046
ENSG00000104219
ENSG00000179242
ENSG00000128016
ENSG00000111679
ENSG00000175029
ENSG00000171456
ENSG00000063169
ENSG00000163568
ENSG00000116288
ENSG00000140044
ENSG00000136158
ENSG00000049768
ENSG00000147274
ENSG00000141646
ENSG00000259207
ENSG00000182916
ENSG00000073578
ENSG00000196411
ENSG00000180900
ENSG00000147403
ENSG00000161638
ENSG00000144843
ENSG00000140937
ENSG00000165238
ENSG00000133121
ENSG00000166949
ENSG00000114395
ENSG00000111816
ENSG00000197579
ENSG00000169242
ENSG00000114383
ENSG00000138279
ENSG00000089022
