# Supplementary material for: Prevalent Accumulation of Non-Optimal Codons through Somatic Mutations in Human Cancers
Source: PLoS One. 2016 Aug 11;11(8):e0160463. doi: 10.1371/journal.pone.0160463 (PMC4981346; doi:10.1371/journal.pone.0160463)
Supplement: S14 Table — The p-values were estimated by Chi-square, two-tail test. (PDF) [file pone.0160463.s016.pdf]

	Dataset	O->O	O->N	%	p-value	N->N	N->O	%	p-value
**Non-Synonymous mutations**	**CosMic**	126,020	**13,272**	9.53	-	95,459	**3,211**	3.25	-
	**SNP-Poly**	9,846	**426**	4.15	2.20E-74	7,166	**341**	4.50	*2.19E-09*
	**Ortholog-Poly**	43,674	**1,856**	4.08	*4.48E-297*	28,749	**1,754**	5.75	*2.09E-87*
**Synonymous mutations**	**CosMic**	5,068	**43,791**	89.63	-	2,314	**9,893**	81.04	-
	**SNP-Poly**	1,397	**9,186**	86.80	2.43E-17	473	**6,434**	93.15	6.20E-115
	**Ortholog-Poly**	10,398	**45,212**	81.30	*0.00E+00*	3,576	**45,127**	92.66	*0.00E+00*
